# Feed-Forward Neural Network for Predicting Enantioselectivity of the Asymmetric Negishi Reaction

**DOI:** 10.1021/acscentsci.3c00512

**Published:** 2023-08-24

**Authors:** Abbigayle E. Cuomo, Sebastian Ibarraran, Sanil Sreekumar, Haote Li, Jungmin Eun, Jan Paul Menzel, Pengpeng Zhang, Frederic Buono, Jinhua J. Song, Robert H. Crabtree, Victor S. Batista, Timothy R. Newhouse

**Affiliations:** † Department of Chemistry, Yale University, New Haven, Connecticut 06511, United States; ‡ Chemical Development, Boehringer Ingelheim Pharmaceuticals Inc, 900 Ridgebury Road, Ridgefield, Connecticut 06877, United States

## Abstract

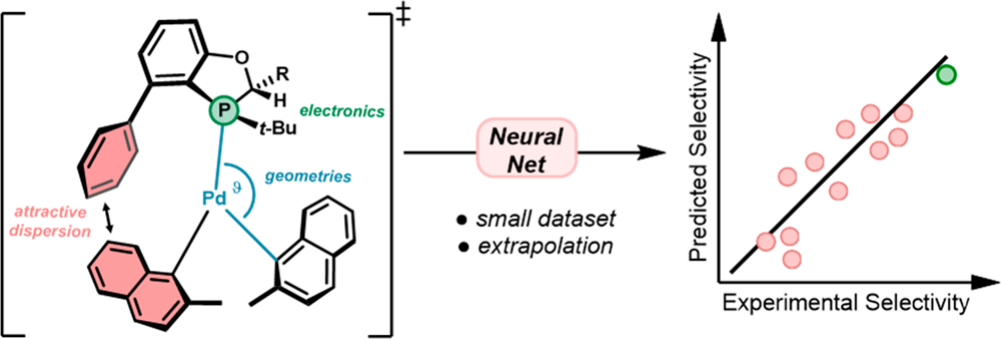

Density functional theory (DFT) is a powerful tool to model transition state (TS) energies to predict selectivity in chemical synthesis. However, a successful multistep synthesis campaign must navigate energetically narrow differences in pathways that create some limits to rapid and unambiguous application of DFT to these problems. While powerful data science techniques may provide a complementary approach to overcome this problem, doing so with the relatively small data sets that are widespread in organic synthesis presents a significant challenge. Herein, we show that a small data set can be labeled with features from DFT TS calculations to train a feed-forward neural network for predicting enantioselectivity of a Negishi cross-coupling reaction with *P*-chiral hindered phosphines. This approach to modeling enantioselectivity is compared with conventional approaches, including exclusive use of DFT energies and data science approaches, using features from ligands or ground states with neural network architectures.

## Abstract 2

We report a feed-forward neural network trained on a small dataset of DFT calculated transition states to predict the enantioselectivity of the asymmetric Negishi cross-coupling reaction.

## Introduction

**Figure 1:**
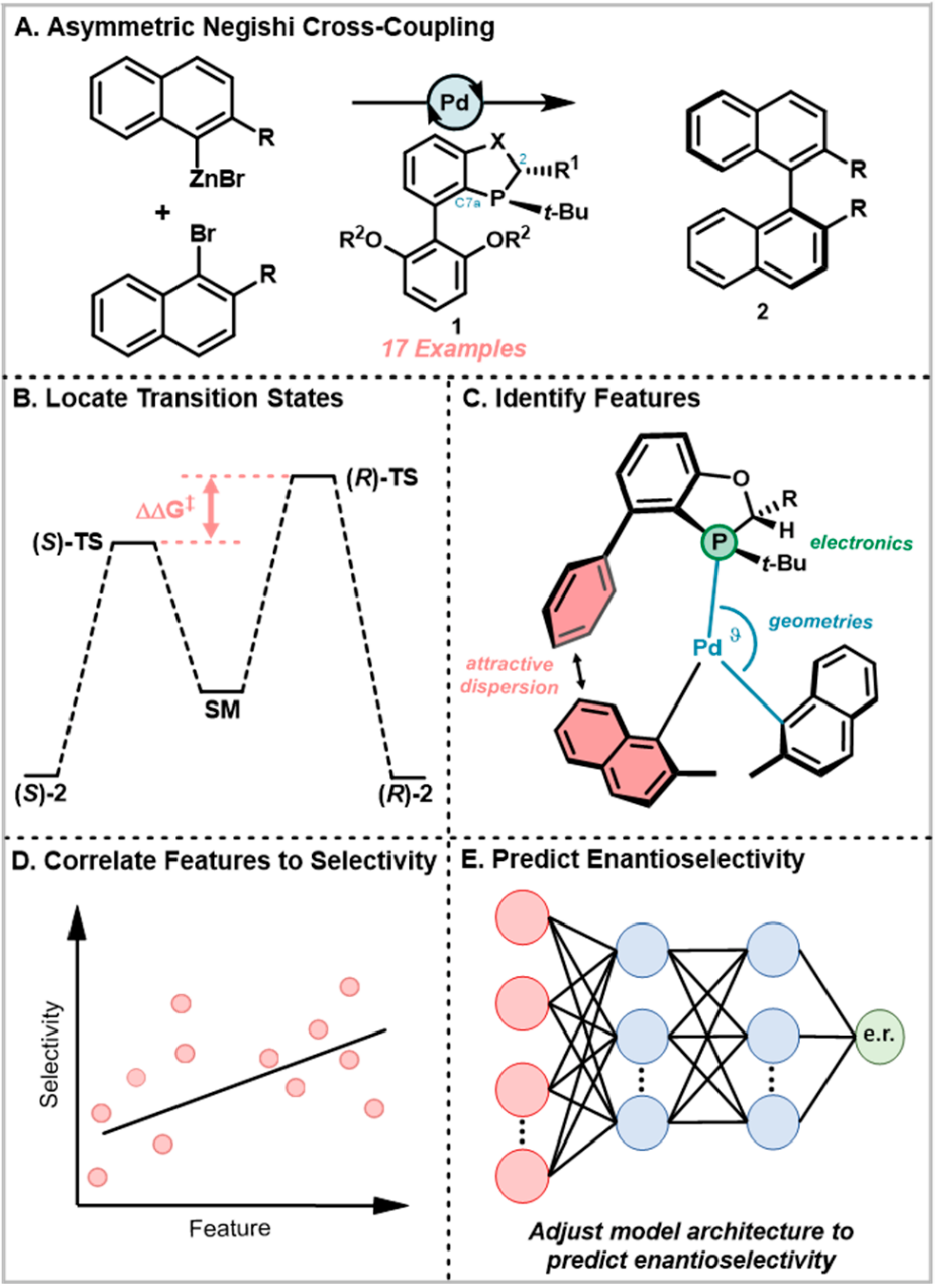
Process for model development of the asymmetric Negishi reaction catalyzed by BOP ligands.

Axially chiral biaryl compounds are prevalent in many biologically active natural products and pharmaceuticals, while also serving as the backbone for thousands of chiral catalysts and ligands.^1−3^ Transition-metal-catalyzed C(sp^2^)–C(sp^2^) cross-coupling reactions remain one of the most straightforward and versatile ways to achieve the synthesis of these motifs.^4−6^ One powerful method that has emerged is the Pd-catalyzed Negishi coupling using *P*-chiral dihydrobenzooxaphosphole (BOP) ligands^7^ (Figure 1A).^8^

Challenges in evaluating and predicting the efficiency of new ligands in these and other catalytic reactions are commonly encountered when using the one-variable-at-a-time optimization approach to isolate the effect of a single variable.^9^ This approach to screening ligands allows for rapid evaluation of hypotheses, but it is challenging to locate the global maximum in a multidimensional space, or otherwise is overly time- and resource-intensive to iteratively probe new ligands.^10^ Predictive approaches promise to overcome these limitations with density functional theory (DFT) being one of the most commonly implemented methods for modeling enantioselectivity.^11−13^ Not only do the differences in free energies of transition states (TSs) (ΔΔ*G*^‡^) provide physically meaningful results (Figure 1B), but the geometries obtained can provide stereomechanistic understanding and inspire synthetic innovation (Figure 1C).^12,14−18^ The principal limitation with exclusively using energy barriers from DFT calculations derives from small errors that lead to dramatically significant synthetic conclusions.^13,19^ For example, for a reaction with a 90:10 er, the ΔΔ*G*^‡^ for the diastereomeric transition states is 1.3 kcal/mol, and nuanced variation of that catalyst control can easily be obscured by errors in calculation (up to ∼1 kcal/mol).^20,21^ Although higher levels of theory could in principle resolve this challenge, these methods are prohibitively expensive for complex substrates and catalysts.^22^

Herein, we report a more accurate approach to predicting the ΔΔ*G*^‡^ by upgrading DFT transition state calculations with a feed-forward neural network (NN). This workflow is applied to the asymmetric Negishi reaction but, in principle, could be applied to a wide range of chemical reactions (Figure 1E). By extracting a maximum amount of information from each data point through the use of DFT transition state calculations, the training on larger data sets can be avoided. With this approach, our method succeeds with a deliberately small training set (17 data points). Notably, the vast majority of literature data sets of catalytic reactions is approximately this size.

Machine learning (ML) models benefit from maximally large data sets for training.^23,24^ This type of data is typically accessed by searching through chemistry databases for relevant reactions or obtaining data through high-throughput experimentation (HTE).^25,26^ While these data collection methods are valuable tools for creating large data sets, a few immediate challenges have become apparent: (1) data available on chemistry databases can be inconsistently catalogued,^27^ (2) training space for models is limited by bias toward high-performing examples,^28^ and (3) generating large data sets via HTE can be technically challenging particularly for reactions with conditions that necessitate additional sophisticated equipment. These problems are a common challenge faced by the modeling community, and while using smaller data sets has been a successful approach across various chemistries,^25,29^ success training neural networks with deliberately small data sets is limited. The potential advantage is the ability to work on problems where little data exists.

The choice of the ligand for the Negishi cross-coupling reaction is crucial for a highly enantioselective outcome.^30^ Patel and co-workers^7^ recently reported a method for the Pd-catalyzed asymmetric Negishi cross-coupling of tetra-*ortho*-substituted biaryls in good yield and enantioselectivity (Figure 1A). In that report, 17 ligands that shared a common backbone structure were applied to the Negishi cross-coupling reaction. Several qualitative and generalizable conclusions were drawn to inform the catalyst design. It was found that the use of bulkier substituents at C2 and electron-donating groups on the lower aryl ring resulted in higher enantioselectivity (Figure 1A). Although individual variables correlated to higher enantioselectivity, combining these structural variations linearly did not inform the optimal structure or properties of the ligand. To understand this phenomenon, Patel and co-workers performed DFT calculations for 2 of the 17 ligands and concluded that shorter bond distances between the Pd and O of the lower aryl ring (Figure 1A) could be associated with the lower barrier to achieving the *S* isomer. This study provides a fascinating mechanistic framework for considering further ligand design but did not lead to quantitative predictions.

## Computational Methods

**Figure 2:**
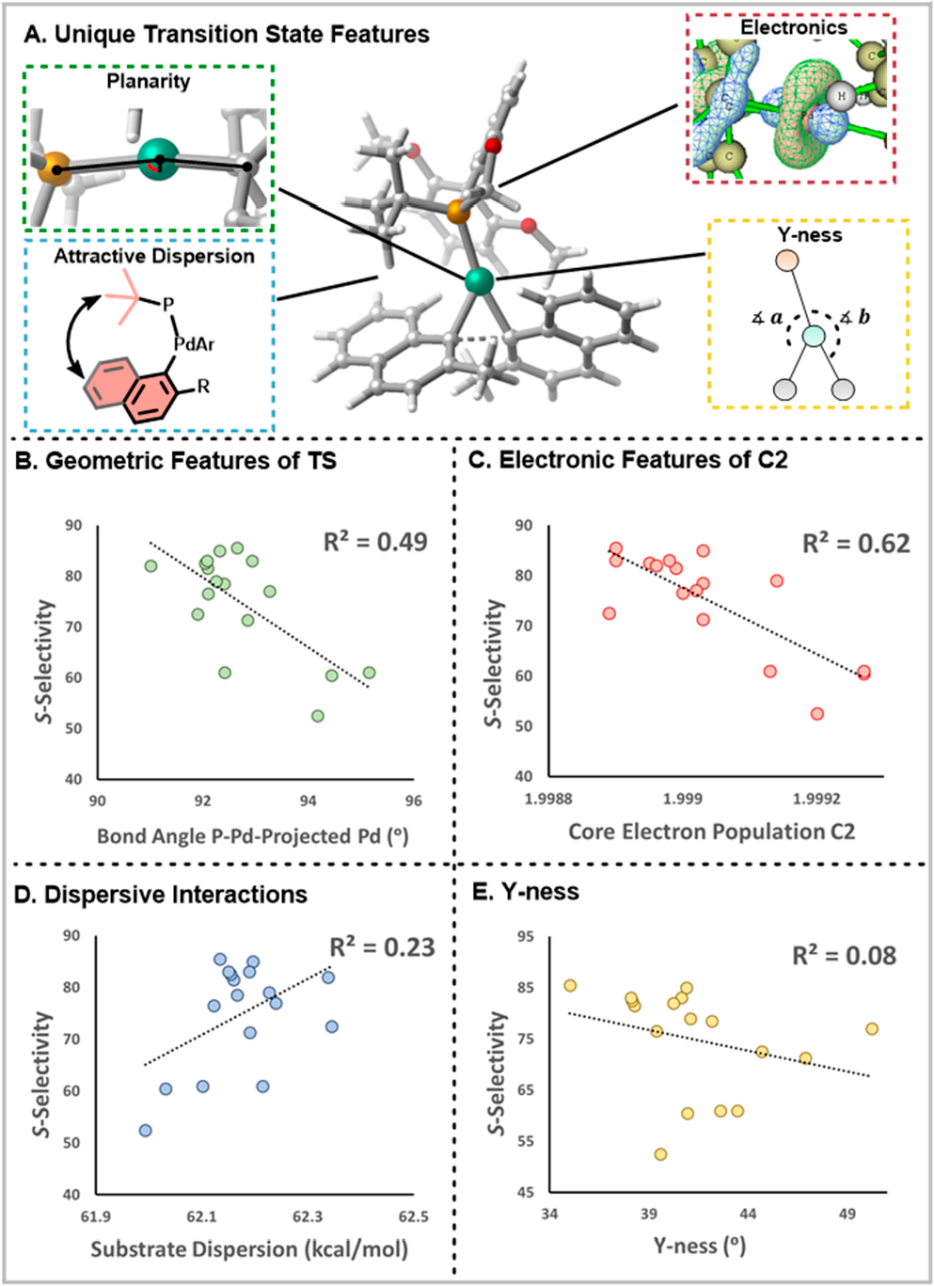
(a) Unique features to the asymmetric Negishi reaction. (b) Correlation of the bond angle between P, Pd, and the projected Pd to selectivity (c) Correlation of electronics of C2 to selectivity. (d) Correlation of dispersive interaction between substrates to selectivity. (e) Correlation of the deviation from a regular Y-shape to selectivity.

Pro-*S* and pro-*R* transition state geometries for these 17 ligands were calculated in the gas phase using DFT at the B3LYP/6-31G(d,p) level of theory^31−35^ and the LANL2DZ pseudopotential for Pd.^36^ To refine the calculations, single-point energy calculations were performed at the wB97x-D/6-3111++G(d,p) level of theory^37−39^ and the def2-TZVP pseudopotential for Pd.^40,41^ Manual inspection of these geometries was conducted to identify potentially significant features that could affect enantioselectivity. Geometries and other computed properties were correlated to efficiency. Figure 2A highlights some representative features, and a complete list can be found in the Supporting Information (Figure S4).

The features included geometric features that attempt to describe the extent to which the ligand impacts the spatial organization of the substrates. These descriptors included the extent to which Pd was projected out of the plane defined by its ligands (Figure 2A, Planarity), the extent to which the ligand is oriented toward one of the substrates as defined by deviation from a regular Y-shaped complex (Figure 2A, Y-ness), and other bond lengths and angles. The most significant geometric features were the bond length between C2 and P, how far Pd was puckered from the plane, and the bond angle between P–C4–C5.

In addition to geometric features of the TS, electronic features were also analyzed (Figure 2D). Analysis revealed that the atoms that experienced the strongest correlation to selectivity in terms of electronic features were P and C2 of the oxaphosphole or azaphosphole ring. This result is in good agreement with experimental observations, as C2 substitution was required for good selectivity.

Additionally, attractive dispersion was investigated by computing this feature by the method of Liu and co-workers.^42^ While dispersion has been found to be important in asymmetric catalysis,^43,44^ to the best of our knowledge it has not been used as a feature for a neural network to describe the efficiency of a chemical reaction. Multiple dispersive interactions between the ligand and substrates were identified and studied at the M06-D3/6-311+G(d,p) level of theory^45^ (Figure 2D). While a significant correlation was observed (*R* = 0.23), this was not one of the highest levels. Although dispersive interactions are evidently important based on the magnitude of these values (e.g., *R*^2^ = 0.32, 0.21), this feature did not vary in a useful way across the ligands in this data set.

## Results and Discussion

### Neural Network Architecture

With the features in hand, we sought to build a feed-forward neural network with this small data set. Our approach was to first develop a series of high-performing models and then evaluate those models with two additional experimentally obtained data sets. Because of the small size of the training set, all models were trained with the leave-one-out (LOO) cross-validation method, and input features were scaled from 0 to 1 using min–max normalization. Overfitting was evaluated through these plots, input of random data, and by evaluating how closely the slope approached the expected value (*m* = 1), as well as the *R*^2^, and the root mean square error (RMSE).

**Figure 3:**
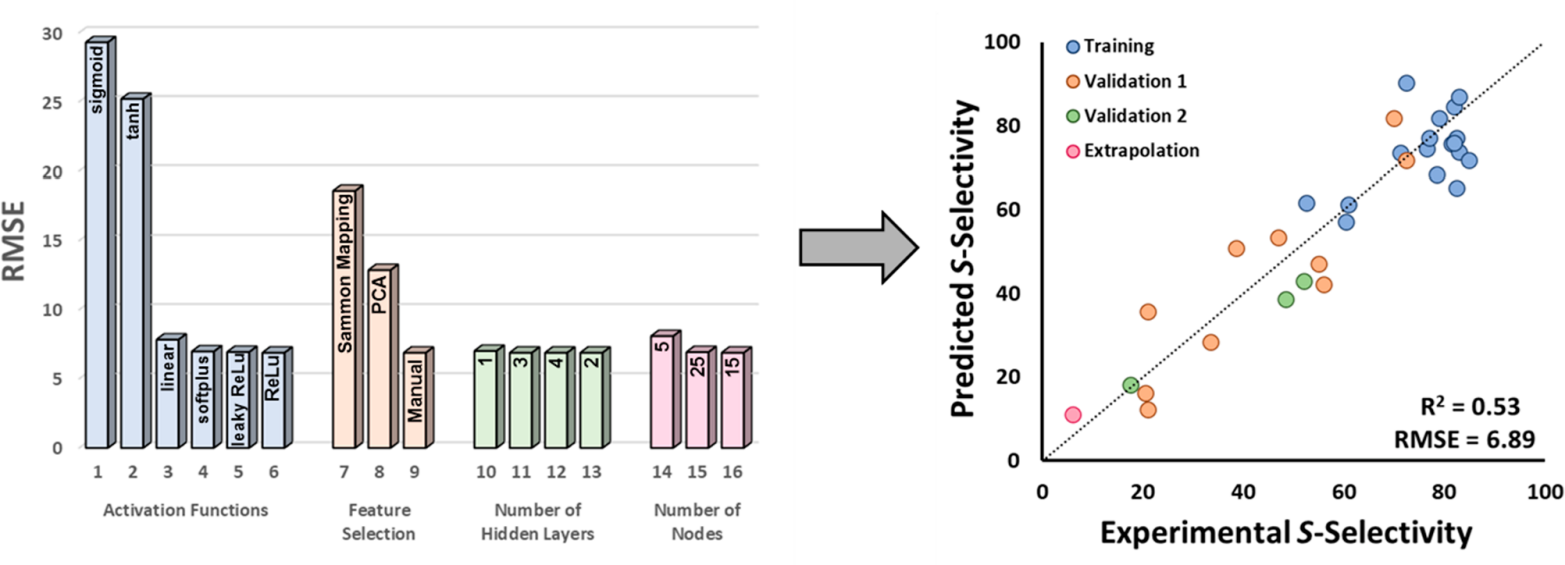
Evaluating the performance of the feed-forward neural network on the basis of fine-tuning of the activation function, feature selection, number of hidden layers, and number of nodes in the hidden layers. The final performance of the model on the training and validation sets is shown on the right.

We recognized that a larger number of features would result in overfitting, so our objective was to use a minimal number of features while maintaining the model performance. Faced with the challenge of paring down the input features to the number of literature examples (17 or less), a few methods were evaluated by Sammon mapping, principal component analysis (PCA), and manual selection (Figure 3). When the number of input features was reduced to 15, Sammon Mapping (RMSE = 18.6) and PCA (RMSE = 12.9) were less effective than manual selection (RMSE = 6.9) because manual selection involved human inspection of the correlation between the features and selectivity.

With the input features to the model determined, the hyperparameters of the model were fine-tuned.^46^ We began by comparing the performance of different activation functions in the hidden layers.^47^ The sigmoid and hyperbolic tangent functions gave the worst performance (Figure 3). Rectified linear units (ReLu), softplus, and Leaky ReLu all produced similar RMSEs, with ReLu having the lowest (6.9). The introduction of nonlinearity to the model proved to be beneficial because a completely linear model saw a higher RMSE (7.8) compared with using the ReLu activation function. Additionally, we examined the model performance by tuning the number of hidden layers and the number of neurons in the hidden layers.^48,49^ However, changing these variables did not result in significant performance changes of the model, so our final architecture utilized two hidden layers with 15 nodes in each of the layers (Figure 3).

### Model Validation

In addition to employing LOO cross-validation, a number of other tests were used to evaluate for overfitting. A series of randomization tests, including *Y*-shuffling (shuffling the labels) and *X*- and *Y*-randomization (generating random inputs and labels) afforded models with high RMSEs and no correlation (RMSE = 33.7, *R*^2^ = 0.0) (see the Supporting Information). These results suggested the model was capturing chemically meaningful information from the data.

With the final architecture of the NN defined, five versions of the model were created. Each of these five models is representative of a different metric to evaluate enantioselectivity. These metrics include enantiomeric excess (ee), enantiomeric ratio (er), the natural log of the er [ln(er)], the *S*-selectivity, and the *R*-selectivity. To determine which one of these models provided the best predictions, 10 validation ligands were chosen because of their ready availability and were used to evaluate the five models. The ligands were selected for their accessibility without any deliberate consideration made for how they may perform. While from a chemical standpoint all models should perform similarly, numerical distributions of data inputs may lead to differential outcomes: the model that predicted *S*-selectivity gave the best predictions (Figure 3). As the model uses features that are inherently not chiral, for example, the extent of electron density at one position, we would not expect equal and opposite results from pro-*R*- and pro-*S*-derived models. Because we fit the 10 validation points to the data when choosing our final model, we performed a second validation with three additional ligands as an additional experimental validation. The enantioselectivity of these ligands was also predicted by our model with good error (RMSE = 7.73; Figure 3).

The features that were important to the model’s performance were core and valence electron population at C2, geometric deviation from planarity by Pd, and the bond angle at the dihydrobenzooxaphosphole ring junction position adjacent to phosphorus. These comprehensible factors provide tunable parameters to impact selectivity.

**Figure 4:**
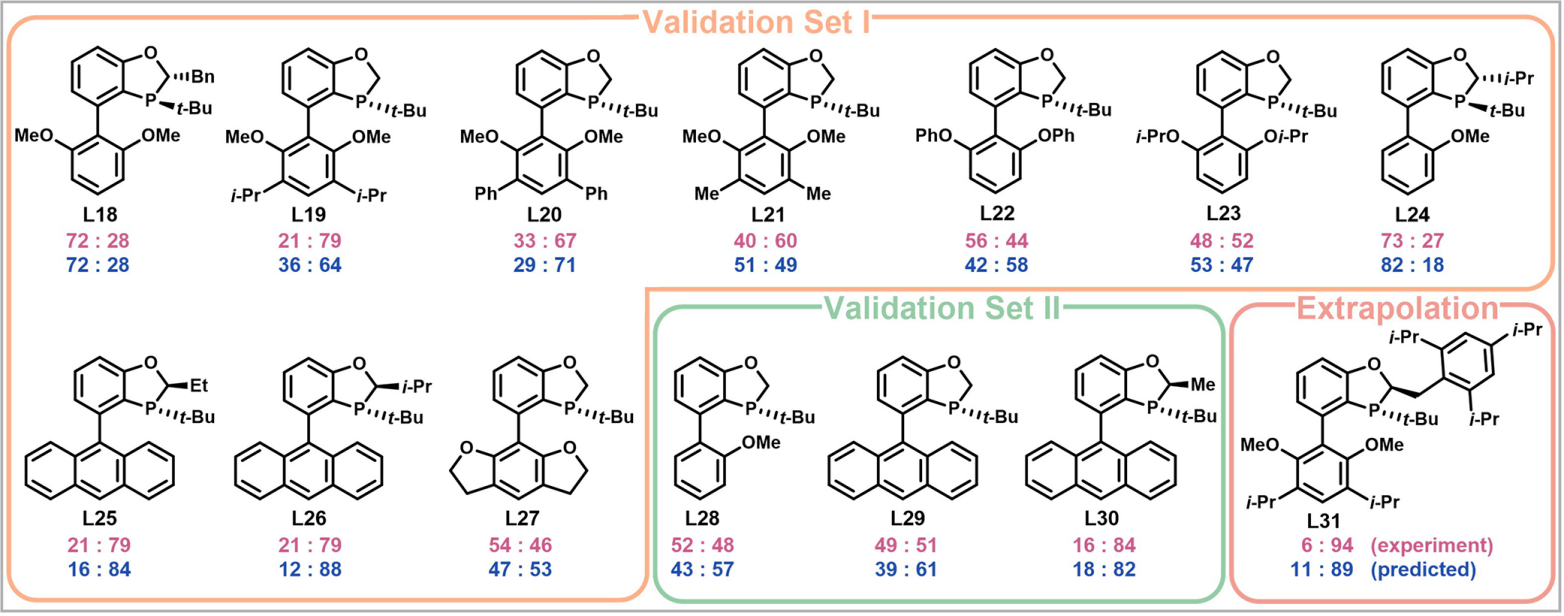
Comparison of experimental values (pink) to model predictions (blue) of validation sets I and II and extrapolation.

After modeling was complete, the 30 ligands of the training and validation sets were manually inspected in order to identify the key structural features of highly selective ligands. A series of ligands was designed, and their selectivity was computationally evaluated by our model. Of the ligands that were designed, L31 (Figure 4, Figure S11) was singly selected as a candidate for extrapolation to a higher selectivity regime. The predicted selectivity (11:89) was in good agreement with the experimentally determined value (6:94). Excitingly, this ligand achieved selectivity higher than that of the ligands in the training or validation sets.

### Comparing Different Approaches to Modeling

**Figure 5:**
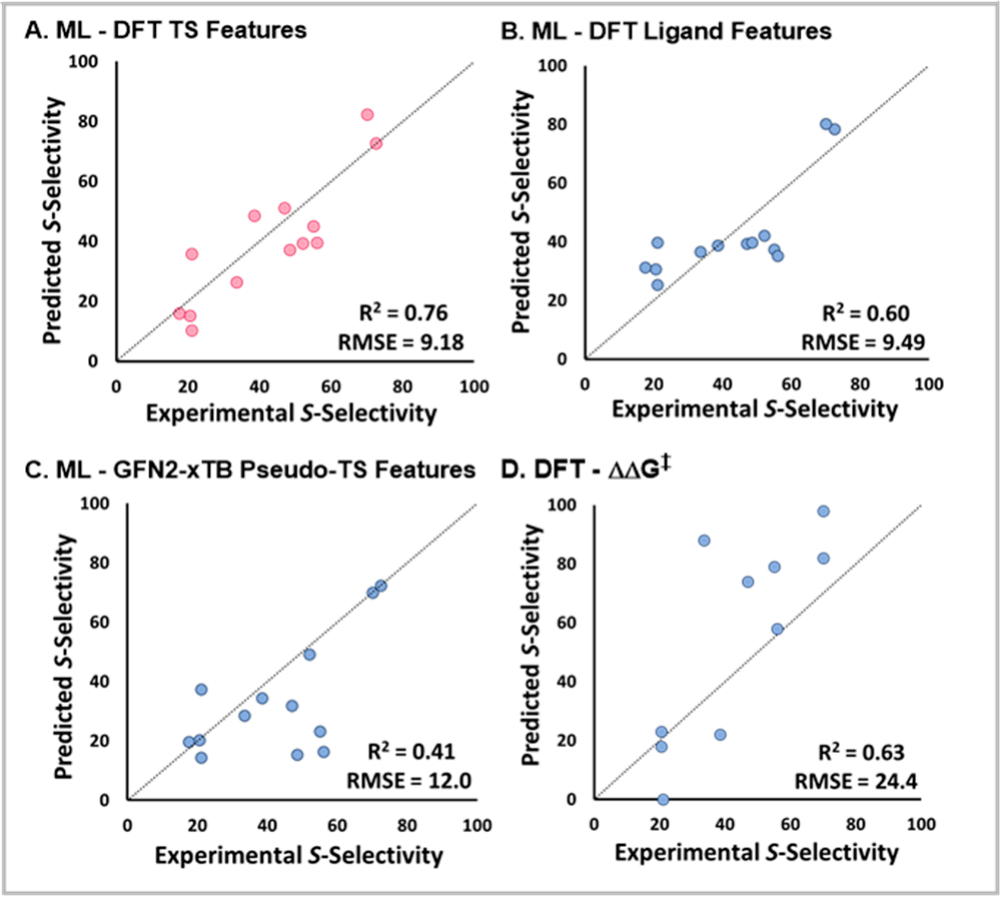
Various methods for modeling enantioselectivity. All points shown are the validation set 1 and 2 predictions. (a) Our ML-DFT TS approach, (b) NN based on DFT calculations of only the ligand, and (c) NN based on pseudo-TS calculations with GFN2-xTB. (d) Linear regression based on DFT-calculated ΔΔ*G*^‡^.

To highlight the efficacy of modeling by the approach described herein, we compared our approach with several commonly defined approaches that are successful with larger data sets. Figure 5A shows the correlations for the validation sets for our DFT-ML approach. This approach is compared with a model built by using features derived exclusively from the ligand.^22^ As illustrated by the representative plot in Figure 5B, this approach resulted only in regression to the mean.

A less resource-intensive semiempirical method (GFN2-xTB) was also applied to the model.^50−52^ As an approximation, pseudo-TSs were located by traversing along the reaction coordinate by locating the highest energy point as the distance between coupling carbons was decreased. Modeling with these geometries and features calculated by DFT was met with more limited success (RMSE = 12.0, Figure 5C). For example, experimental results revealed that the C2 substitution was required for good selectivity. While our DFT-ML model captured this trend, the model built from GFN2-xTB could not: for L29 our calculated er (39:61) was in good agreement with the experimentally observed value (49:51), whereas the GFN2-xTB model was not (15:85). This may indicate that the accuracy of the DFT geometries is essential for estimating the electronic properties imparted by the ligand.

We also compared our DFT-ML method with the conventional DFT approach of calculating er by relating ΔΔ*G*^‡^ to equilibrium. As shown in Figure 5D, the limited extent of correlation observed highlights the challenge of correlating enantioselectivity to purely DFT-calculated energies [computed as wB97x-D/6-311++G(d,p)-def2-TZVP(Pd)//B3LYP/6-31G(d)-LANL2DZ(Pd); see the Supporting Information for additional levels of theory). Like the GFN2-xTB model, the purely DFT approach was unable to capture the importance of substitution at C2. A ligand that lacks a substituent at C2, L27, is predicted to have high selectivity by the purely DFT approach (79:21), yet a relatively low level of selectivity is experimentally observed (54:46). In contrast, the DFT-ML model identifies this counterintuitive selectivity with a predicted er of 47:53.

## Conclusion

Using molecular features from a small number of DFT-calculated TSs as inputs for a neural network allows for the accurate prediction of ligand efficiency for a Negishi cross-coupling reaction and extrapolation to the design of a more selective ligand. The DFT TSs revealed new geometric, electronic, and attractive dispersive interactions whose individual moderate correlations could be productively utilized for predicting enantioselectivity. Moreover, our results identify specific and readily comprehensible factors that influence the final outcomes. We conjecture that the input features indicate how early or late the TS is along the C–C coupling coordinate, which, in turn, predicts the extent of selectivity more precisely than simply relying on TS energies.

It is anticipated that this could be a useful approach for model development of mechanistically understood transforms, especially when experimental data on structurally relevant molecules is limited. This circumstance necessarily arises as research is directed toward novel chemical space in the search for new types of matter with unexplored properties.

## Supporting Information Available

Experimental procedures, HPLC traces and transition state coordinates, full model, and computational details (PDF)
