# Supplementary material for: Feed-Forward Neural Network for Predicting Enantioselectivity of the Asymmetric Negishi Reaction
Source: ACS Cent Sci. 2023 Aug 24;9(9):1768–74. doi: 10.1021/acscentsci.3c00512 (PMC10540279; doi:10.1021/acscentsci.3c00512)
Supplement: Supplementary file 1 — oc3c00512_si_001.pdf [file oc3c00512_si_001.pdf]

Feed-Forward Neural Network for Predicting Enantioselectivity of the Asymmetric Negishi Reaction

Abbigayle E. Cuomo[1], Sebastian Ibarraran[1], Sanil Sreekumar[2], Haote Li[1], Jungmin Eun[1], Jan Paul Menzel[1], Pengpeng Zhang[1], Frederic Buono[2], Jinhua J. Song[2], Robert H. Crabtree[1], Victor S. Batista*[,1], Timothy R. Newhouse*[,1]

[1]Department of Chemistry, Yale University, New Haven, CT 06511, United States
[2]Chemical Development, Boehringer Ingelheim Pharmaceuticals Inc, Inc, 900 Ridgebury Road, Ridgefield, Connecticut 06877, United States

*Email: timothy.newhouse@yale.edu, victor.batista@yale.edu

Table of Contents

1. Computational Details **S2**
1.1 Transition State Search S2
1.2 Dispersion Calculations S4
1.3 Feature Extraction S5
2. Machine Learning Models **S6**
2.1 Feature Reduction S6
2.2 Neural Network Architectures S7
2.3 Tests for Overfitting S9
2.3 Validation Set Predictions S10
2.4 Other Approaches to Modeling S14
2.4.1 Ligand Model S14
2.4.2 GFN2-xTB S15
2.4.3 $\Delta\Delta G^{\ddagger}$ S17
2.4.3 Other ML Models S17
2.5 Feature Space S18
3. Experimental Details **S19**
3.1 General Experimental Procedure S19
3.2 Characterization Data for Validation Set S20
4. Computational Coordinates **S38**
5. References **S149**

1. Computational Details

1.1 Transition State Search

All density functional theory (DFT) calculations were performed using the Gaussian 16 suite of programs (revision A3).[1] The B3LYP functional was used to investigate the reaction pathways.[2] Geometry optimizations for the ground states, transition states and products were performed with the LANL2DZ pseudopotential for palladium (Pd)[3] and the 6-31G(d)[4] basis set for all other atoms. The stationary points are characterized via calculations of the analytical gradients and Hessians. Intermediates and transition states were identified by the observation of the correct number of imaginary eigenvalues in the Hessian matrix: zero (0) and one (1) respectively. To refine the results, single point energy calculations were performed on the gas-phase optimized structures. This was performed using four different methods (1) M06 functional[5] with the LANL2DZ pseudopotential for Pd and the 6-311+g(d,p) basis set[6] for all other atoms (2) B3LYP functional[2] with Grimme's GD3-BJ dispersion correction[7] with the def2-TZVP pseudopotential for Pd[8] and the 6-311++G(d,p) basis set for all other atoms (3) M06-2X functional[5] with Grimme's GD3 dispersion correction[9] with the def2-TZVP pseudopotential for Pd and the 6-311++G(d,p) basis set for all other atoms and (4) wB97x-D functional[10] the def2-TZVP pseudopotential for Pd and the 6-311++G(d,p) basis set for all other atoms. All quoted free energies are reported at 298.15 K in THF and were calculated via the SMD[11] continuum solvation model. To evaluate the effect of electronic properties on selectivity, a full Natural Bond Orbital (NBO) analysis was performed in Gaussian using version 3.1 of the NBO program at the B3LYP/6-31G(d,p) level of theory.[12]

These calculations were performed for 30 ligands; 17 training, 10 validation I, and 3 validation II ligands. Structures of the ligands used in this study are depicted in Table S1.

Table S1. Structures used in this study. L1-L17 were used as the training set, L18-L27 served as validation set I, and L28-L31 served was validation set II.

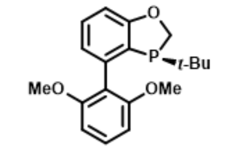 L1 (47.5 : 52.5)	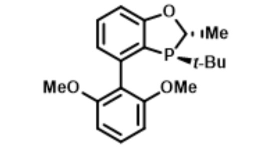 L2 (21.5 : 78.5)	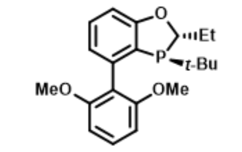 L3 (18.5 : 81.5)	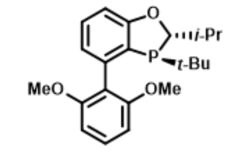 L4 (17.5 : 82.5)
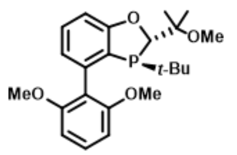 L5 (27.5 : 72.5)	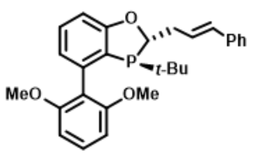 L6 (23.5 : 76.5)	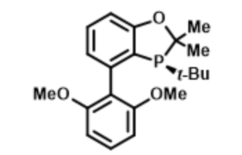 L8 (14.5 : 85.5)	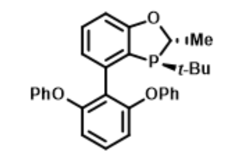 L9 (28.7 : 71.3)
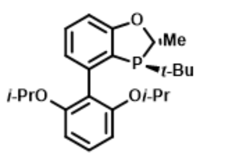 L10 (15.0 : 85.5)	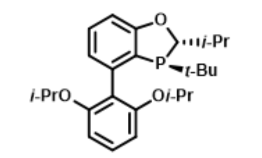 L11 (18.0 : 82.0)	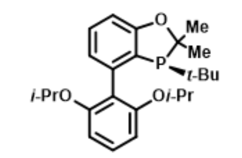 L12 (17.0 : 83.0)	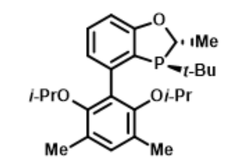 L13 (23.0 : 77.0)
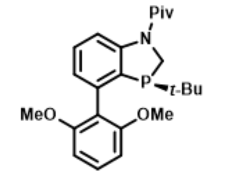 L14 (39.5 : 60.5)	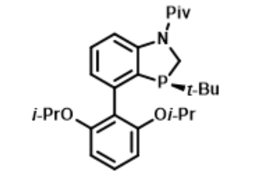 L15 (39.0 : 61.0)	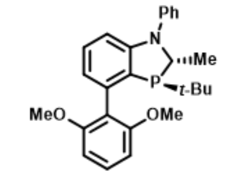 L16 (17.5 : 82.5)	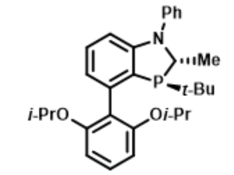 L17 (21.0 : 79.0)
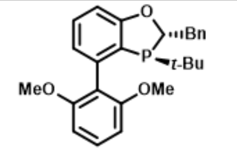 L18 (72.0 : 28.0)	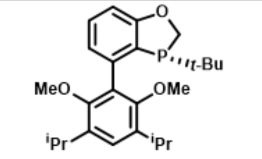 L19 (21.0 : 79.0)	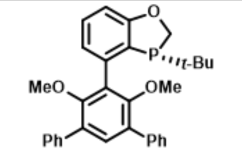 L20 (33.0 : 67.0)	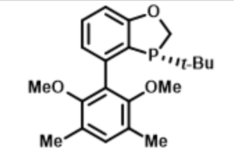 L21 (40.0 : 60.0)
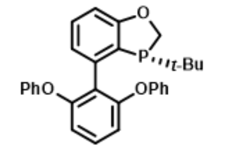 L22 (56.0 : 44.0)	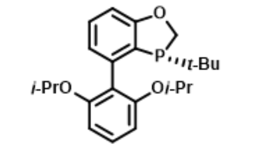 L23 (48.0 : 52.0)	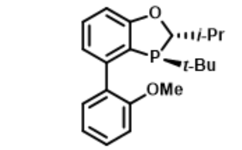 L24 (73.0 : 27.0)	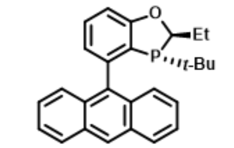 L25 (21.0 : 79.0)
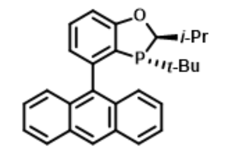 L26 (21.0 : 79.0)	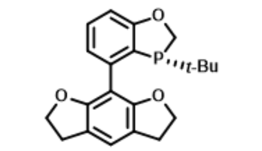 L27 (54.0 : 46.0)	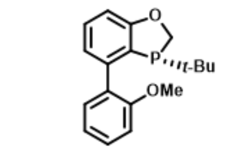 L28 (52.0 : 48.0)	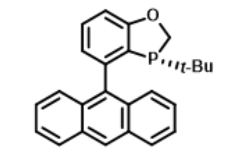 L29 (49.0 : 51.0)
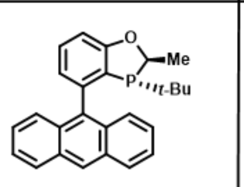 L30 (16.0 : 84.0)	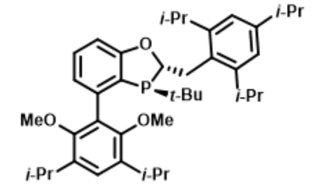 L31 (6.0 : 94.0)	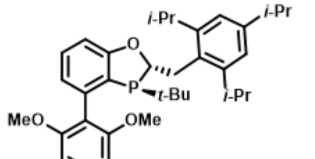 L7 (83.0 : 17.0)	

1.2 Dispersion Calculations

To study the origin of the dispersion interactions between the ligands and the substrate, separate dispersion calculations were performed for groups that were informed to experience such interactions via visual inspection. Grimme's GD3 was used to evaluate empirical dispersion. Dispersion interactions were evaluated between the substrates, the lower aryl ring and the substrates, and the *t*-butyl group and the substrates. The geometries for each of these dispersion calculations were taken from the optimized transition state structures. To isolate the structures of interest to evaluate dispersion, per Peng Liu's method[13], C-C and C-Pd bonds were cleaved and atoms that were not of interest were deleted. Hydrogen atoms were added to dangling carbon atoms at a distance of 1.07Å in the same direction as the cleaved bonds (Figure S1).[13]

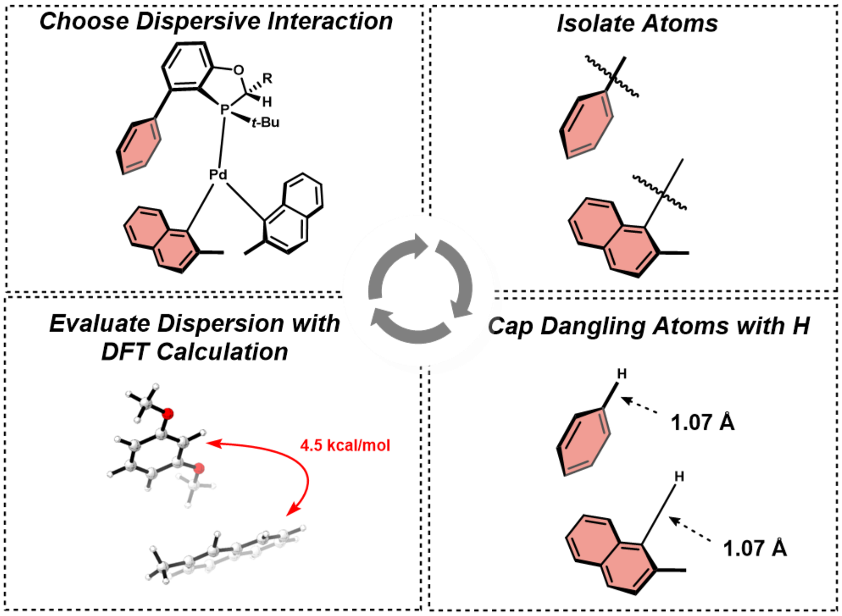

Figure S1. General procedure for calculating dispersion interactions. Isolation of atoms of interested were followed by hydrogen capping and DFT calculations using Grimme's GD3 dispersion correction.

Calculations of the dispersive interaction energies were based on density functional theory (DFT) calculations using the Gaussian 16 suite of programs (revision A3). Single point energy calculations were performed using the M06 functional and the 6-311+G(d,p) basis set with Grimme's GD3 dispersion correction[10]. Figure S2 details the dispersive interactions evaluated for the model.

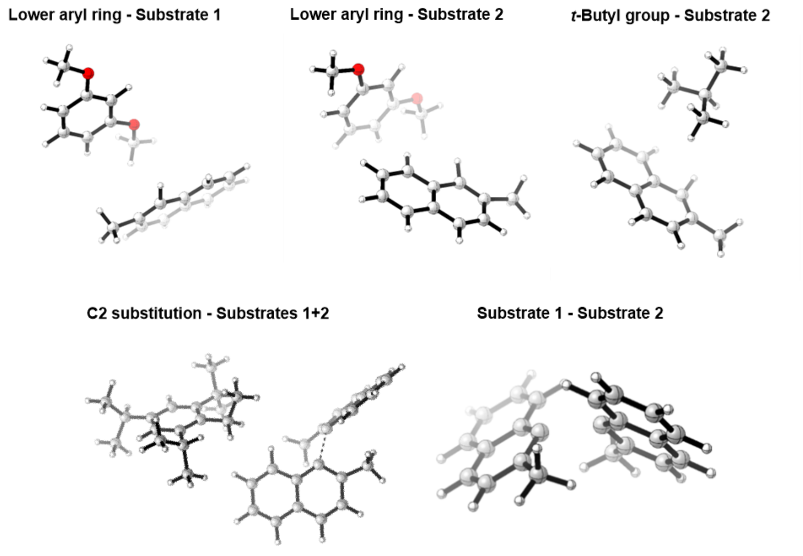

Figure S2. Dispersive interactions analyzed in this study. Molecular representations were generated with CYLview20.

1.3 Feature Extraction

Before features could be extracted for the model, atoms from the DFT calculations were labeled manually. Due to the small size of the dataset and the consistent backbone structure of the ligand, a standard numbering scheme was used for each ligand as depicted in Scheme S1. A python script was used to extract molecular, atomic, and electronic features from Gaussian output files, and all features are listed in Table S2 (source code is available at: https://github.com/Newhouse-Group/6-Endo-Radical-Cyclization).

Scheme S1. Numbering scheme used to extract atomistic and molecular features.

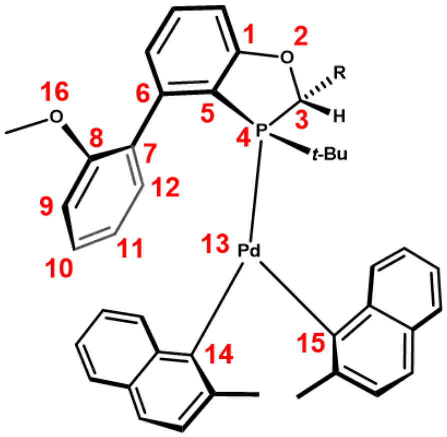

Table S2. Categories of atomic, molecular, and electronic features extracted to use as input for the model.

Dipole Moment	Dihedral Angles	Core Electron Population	NMR Shifts
Bond Lengths	Total Charge	Valence Electron Population	Y-ness
Bond Angles	Charge	Rydberg Population	Puckered Deviation 360°
Imaginary Frequency	ΔG	HOMO/LUMO	Dispersion
Electronegativity	Hardness	Electrophilicity	Sterimol

2. Machine Learning Models

Dimension reduction and machine learning models were created in Google Collaboratory using Python version 3.8. Neural networks (NNs) were generated with PyTorch version 1.13.1+c116. All NNs were generated via the leave-one-out (LOO) cross-validation method due to the small size of the dataset.

2.1 Feature Reduction

Because the training set comprised of only 17 ligands and there were >150 features extracted from the DFT calculations, feature reduction techniques needed to be used to avoid overfitting. Principal Component Analysis (PCA)[14] was first attempted to reduce the ~150 features to 15 (Figure S3A). Additionally, Sammon Mapping[15], was utilized (Figure S3B). Whereas PCA aims to highlight the most descriptive components of a given dataset, Sammon Mapping maps higher dimensional data to lower dimensional data by preserving the inner point distances of the original data. Both models showed moderate performance, however we still sought to find another technique to improve the predictions.

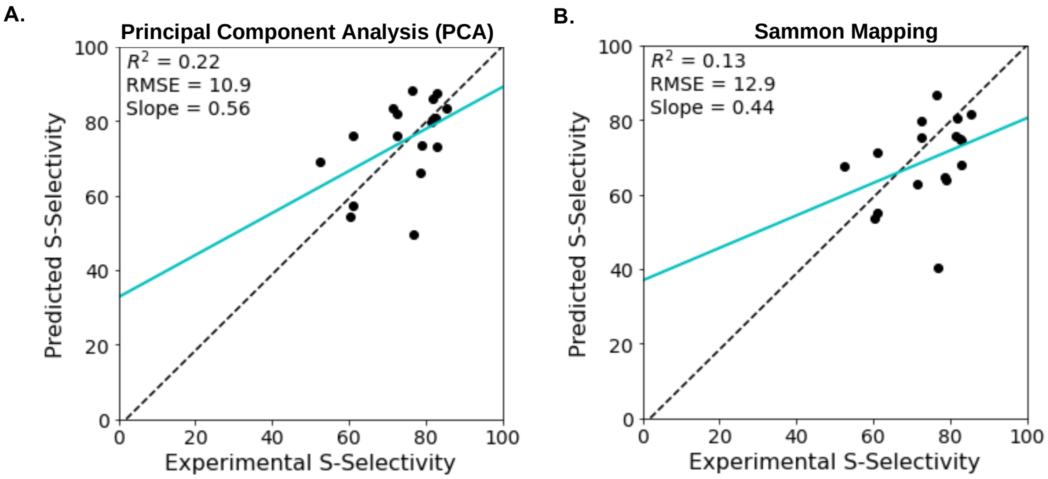

Figure S3. (A) NN performance after reducing ~150 features down to 15 using PCA. (B) NN performance after reducing ~150 features down to 15 using Sammon Mapping.

The third method we attempted to use was correlating the features to the selectivity of the reaction. The fifteen features with the highest R^2 value were then selected as the input features for the model. Electronic features, especially about the C2 position on the oxaphosphole ring, appeared to be dominating factors for determining selectivity. Geometric features, including bond lengths and angles involving the C2 and P were also important features for determining selectivity. Additionally, geometries about the transition state were also important and are highlighted in Figure S4. Creating a NN with this method of feature selection showed to be the most effective method. The model was created according to the procedure outlined above.

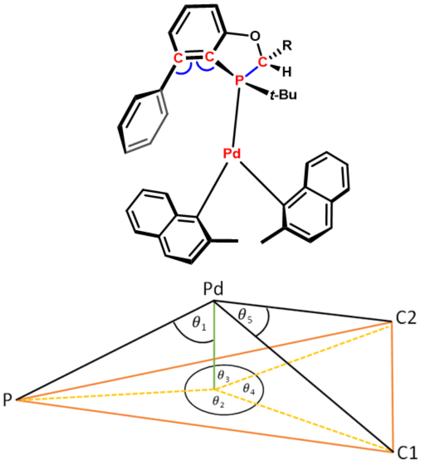

Rydberg 4 (0.69)	NMR 3 (0.51)	Puckered Deviation 360° (0.49)
Valence 3 (0.63)	Rydberg 5 (0.51)	BA 4,5,6 (0.47)
Total 3 (0.63)	BL 3-4 (0.31)	Core 13 (0.40)
Core 3 (0.62)	NMR 4 (0.50)	Core 4 (0.37)
Rydberg 3 (0.57)	P-Pd-Flat Pd (0.49)	BA 5,6,7 (0.22)

Figure S4. (left) Highlighted in red are atoms with features that experienced the highest correlation to selectivity. Highlighted in blue are bond lengths/angles that were also highly correlated. The bottom diagram depicts interesting geometries about the transition state involving Pd. (right) Top 15 features used as inputs for the NN model. In parenthesis are the R^2 value to selectivity.

2.2. Neural Network Architectures

With the method of feature selection finalized, the hyperparameters of the NN were adjusted to evaluate for optimal performance. The first round of optimizations involved changing the activation function in the hidden layers (Table S3). The Rectified Linear Unit (ReLu) had the best overall performance, while the leaky ReLu and softplus performed only marginally worse. Because the feature for the models were chosen based on how high their R^2 value was to the enantioselectivity, the model built with only linear layers also performed relatively well compared to the other activation functions.

In addition to the activation functions, the number of hidden layers and the number of nodes in those hidden layers were also varied (Table S3).

Table S3. Comparative performance of different NN architectures. The best overall performance used the ReLu activation function, 2 hidden layers and 15 nodes in each layer.

Category	Change	R2	RMSE	Slope
Activation Function	Sigmoid	0.02	29.25	-0.01
	Hyperbolic Tangent	0.01	28.58	-0.01
	Linear	0.43	7.84	0.65
	Softplus	0.52	6.94	0.67
	Leaky ReLu	0.52	6.89	0.67
	ReLu	0.52	6.85	0.69
Number of Hidden Layers	1	0.52	7.02	0.70
	3	0.52	6.85	0.69
	4	0.52	6.85	0.69
	2	0.52	6.85	0.69
Number of Nodes	5	0.29	8.09	0.36
	25	0.52	6.89	0.66
	15	0.52	6.85	0.69

A graphical representation of the model's performance as well as a comparison of reported and predicted selectivities are outlined in Figure S5 and Table S4 respectively.

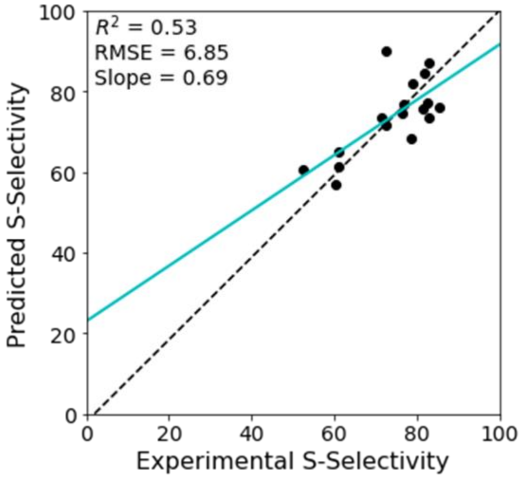

Figure S5. Final model performance on the training data set. Plotted points are LOO predictions.

Table S4. Comparison of reported and predicted values for selectivity of the training set with the final NN architecture.

Ligand	Experimental	Predicted	Ligand	Experimental	Predicted	Ligand	Experimental	Predicted
**L1**	52.5	60.7	**L7**	83.0	73.7	**L13**	77.0	77.0
**L2**	78.5	68.3	**L8**	85.5	75.9	**L14**	60.5	57.1
**L3**	81.5	76.0	**L9**	71.3	73.6	**L15**	61.0	61.3
**L4**	82.5	77.1	**L10**	85.0	71.7	**L16**	61.0	65.2
**L5**	72.5	90.2	**L11**	82.0	84.5	**L17**	79.0	81.8
**L6**	76.5	74.6	**L12**	83.0	87.0			

This final architecture was compared to a version in which $\Delta\Delta G^{\ddagger}$ was used as an input feature in the NN using the single-point energy calculations at the wB97x-D/6-311++G(d,p)-def2-TZVP(Pd) level of theory. Table S5 shows a direct comparison between the two models.

Table S5. Comparison of the final NN architecture with the top 15 features compared to including the $\Delta\Delta G^{\ddagger}$ as an input feature.

Model	R2	RMSE	Slope
Top 15 Features	0.52	6.85	0.69
Top 14 Features + ΔΔG‡	0.47	7.47	0.65

2.3 Tests for Overfitting

Although we took preventative steps to avoid overfitting, it is still necessary to perform the necessary checks to ensure that this is not the case. Various randomization tests were performed to evaluate for overfitting. If this model performs well with this random data, then that indicates that the model was not finding chemically meaningful information amongst the training data and cannot extrapolate to unseen validation data.

The randomization tests used for this study were (1) X-randomization, (2) Y-shuffling, and (3) Y-randomization. X-randomization involves assigning random values to be used as input features to the model (Figure S6A). For Y-shuffling, the enantioselectivity labels of the training data are randomly assigned to each of the training points (Figure S6B). Y-randomization involves assigning random labels (values of enantioselectivity) to the training data (Figure S6C). The low levels of correlation observed by using any of these methods (0.17, 0.00, and 0.00) as well as the slopes being significantly closer to 0 than to 1 (0.01, 0.00, and 0.03) indicates that this NN is not describing the random error of the training data rather than relationships between the variables. Confident that the model was not overfit with these tests, we could continue forward with predicting the enantioselectivity of ligands that were not included in the training set.

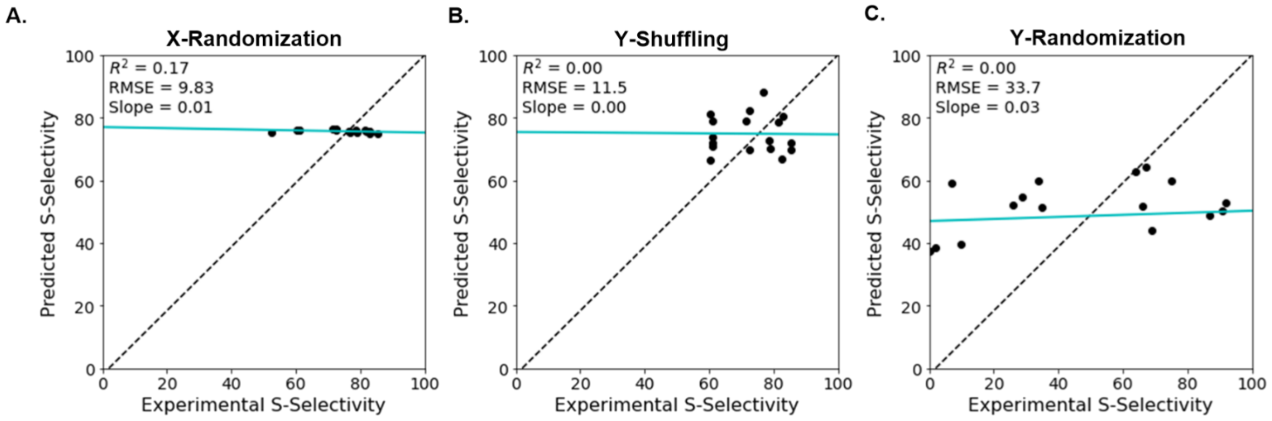

Figure S6. (A) Model created with random x-data as the input features. (B) Model created by randomly assigning each training point with a selectivity label. (C) Model created by randomly generating selectivity labels for each of the training points.

2.5 Validation Set Predictions

When creating these models, there were multiple options for selectivity labels to use for the training. These include; enantiomeric excess (ee), enantiomeric ratio (er), the natural logarithm of the er (ln(er)), *S*-selectivity, and *R*-selectivity. Figure S7 shows the training performance for each of these measures of selectivity using the LOO method for L1-L17.

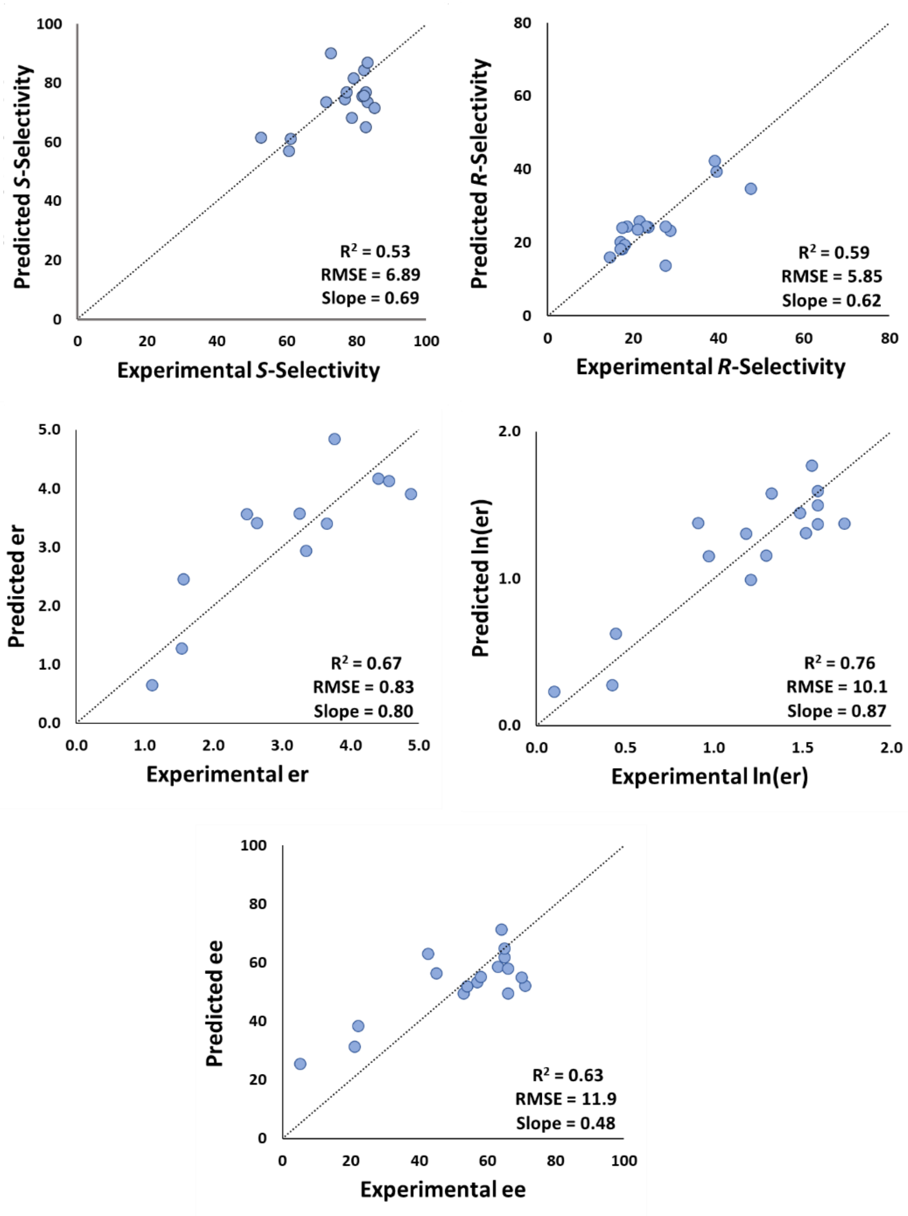

Figure S7. Models built using *S*-selectivity, *R*-selectivity, ee, er, and ln(er) as labels. Model performance was evaluated with the R^2, RMSE, and slope shown in the bottom right corner of each of the plots.

It was evident from these plots that any of these measures of selectivity could provide promising predictions for the validation set. To test these models, 10 of the 13 validation points (L18-L27) were evaluated on each of the models to determine which would be the best model to move forward with. Ligands for the validation sets were strictly chosen based on availability. Figure S8 shows the performance of each of these five models with the first validation set.

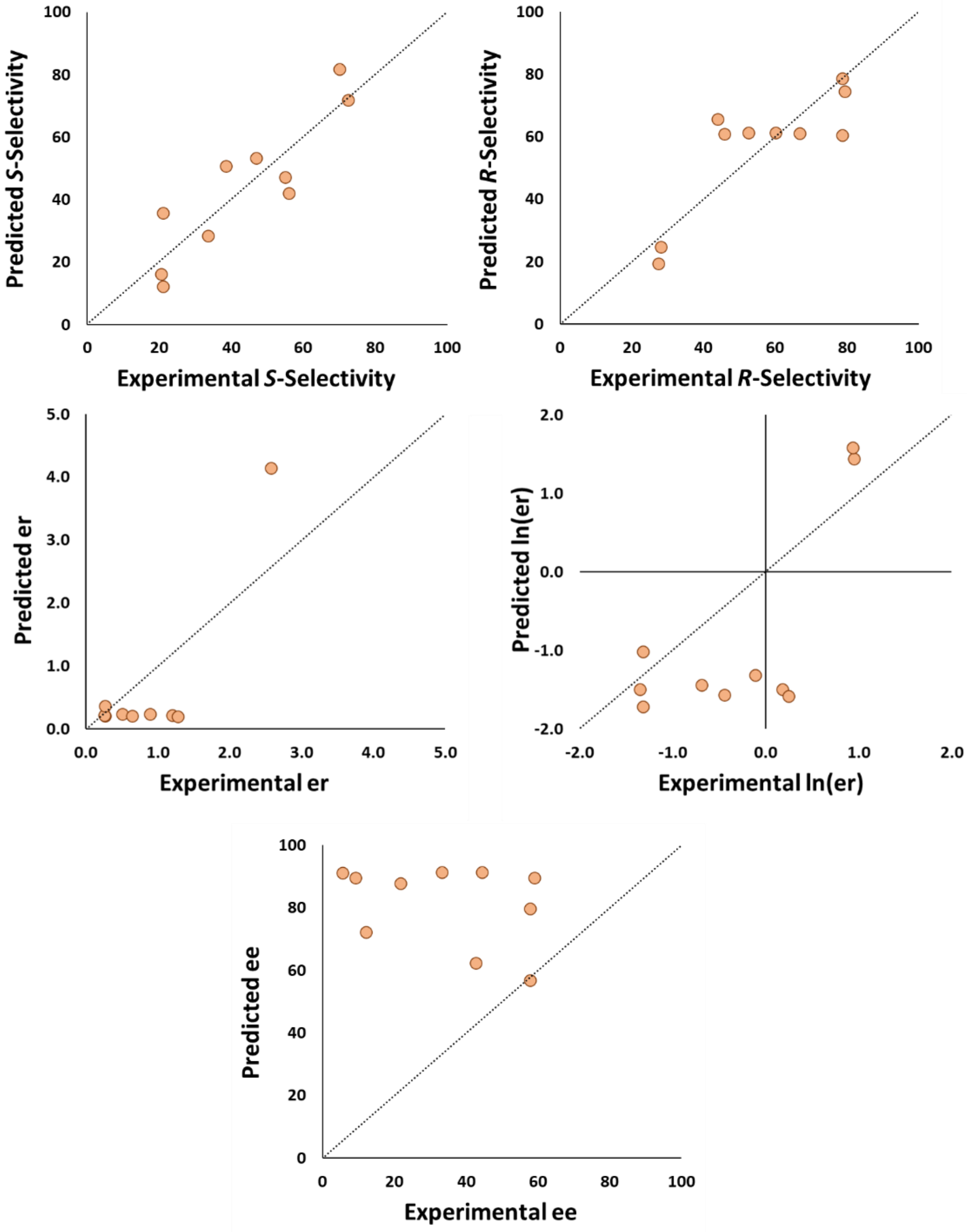

Figure S8. Models built using *S*-selectivity, *R*-selectivity, ee, er, and ln(er) as labels. Performance of the trained models with a validation set of ten ligands (L18-L27).

Evident from these plots, the model trained with *S*-selectivity as the label outperformed the other models. The other four models appear to regress to the mean rather than make meaningful predictions from the chemical data.

Because the validation data was essentially fit to five different models when choosing the final one, an additional validation set of three ligands (L28-L30) was tested with the *S*-selectivity model. Results of validation set I (orange points) and validation set II (green points) for *S*-selectivity are shown in Figure S9.

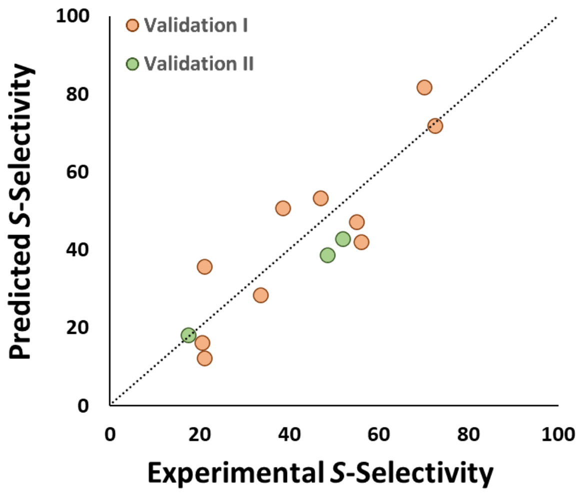

Figure S9. Performance of the final model on validation set I and II.

Figure S10 details the predictions for each of the ligands.

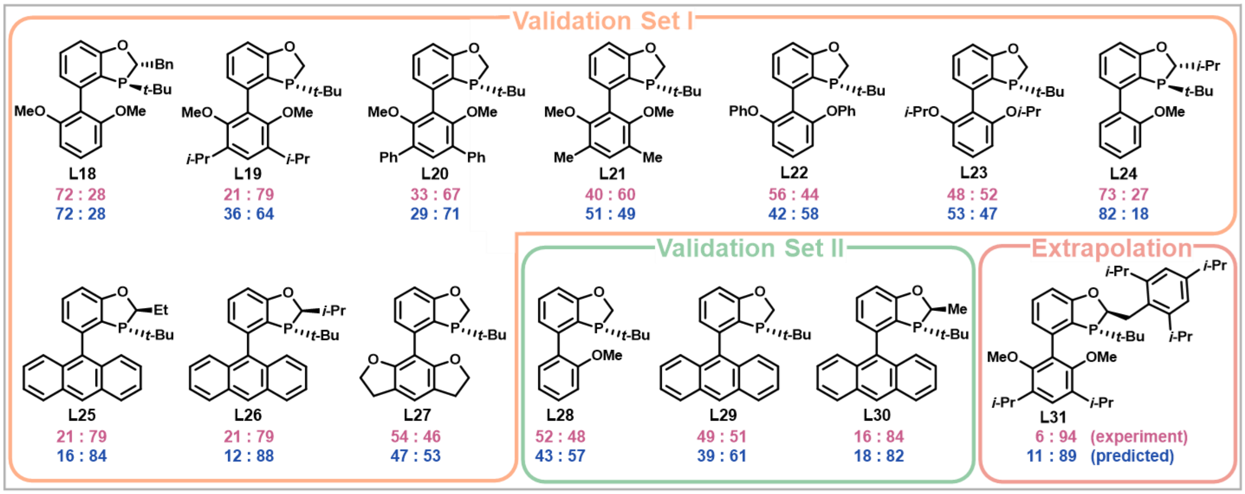

Figure S10. Comparison of experimental and predicted values for *S*-selectivity of validation set I and II.

A series of extrapolation ligands were designed by manual inspection of the training and test sets. Five structures were designed based on trends that, seemingly, positively influenced ligands to be more selective. The design of and subsequent DFT calculation and prediction generation of these proposed structures was complete in about a day's time. The structure of these five proposed structures and their predicted selectivity is shown in Figure S11. L31 was ultimately chosen as the sole ligand for experimental extrapolation due to it yielding the best predicted selectivity.

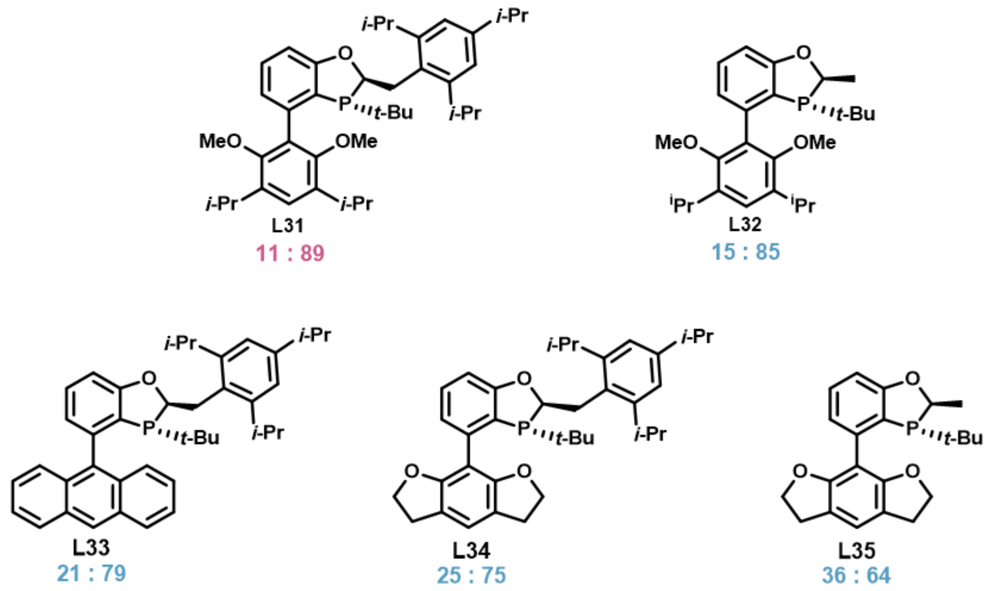

Figure S11. Proposed extrapolation ligands and their predicted selectivity.

2.4 Other Approaches to Modeling

2.4.1 Ligand-Only Model

For this particular model of the Negishi reaction, the structure of the substrate and the conditions of the reaction remained constant. To simplify the model from requiring transition state calculations, we created a model based on features from calculations of only the ligand. Calculations were performed for these structures in the same manner as outlined in section 1.1.

The same features were used for this model as the transition state model. The transition states had additional features that were not present in the calculations of only the ligand (puckered deviation from 360° and P-Pd-Flat Pd). To replace these two features in the ligand-only model, features of the ligand-only model were again correlated to selectivity. The two highest correlated features were then used in their place (Core 5 and Rydberg 2). The performance of the training set (pink points) and validation set (green points) are shown in in Figure S12.

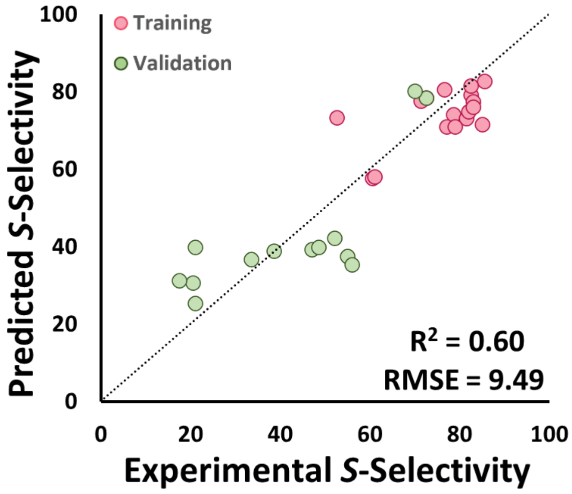

Figure S12. Performance of ligand-only model on training set, validation set I and II. Depicted R^2 and RMSE are in reference to the validation sets.

2.4.2 GFN2-xTB Model

To resemble the transition state DFT calculations as much as possible, the highest energy point along the C-C reaction coordinate was determined with GFN2-xTB[16], from here on called pseudo transition state (pseudo-TS). Pseudo-TSs were located via traversing across the reaction coordinate of the potential energy surface by gradually decreasing the distance between C14 and C15 from 3.0 to 1.0 Å in 200 evenly spaced increments. When the energy reached a maximum, the structure was isolated. To obtain the same features as the DFT TS calculations, subsequent single-point energy and NBO calculations were performed on these optimized structures using Gaussian. Unlike the ligand-only model, all features present in the DFT TS calculations were also present in the GFN2-xTB pseudo-TSs. Therefore, the same features used to construct the DFT TS model were used to create the GFN2-xTB pseudo-TS model. Performance of this model is shown in Figure S13.

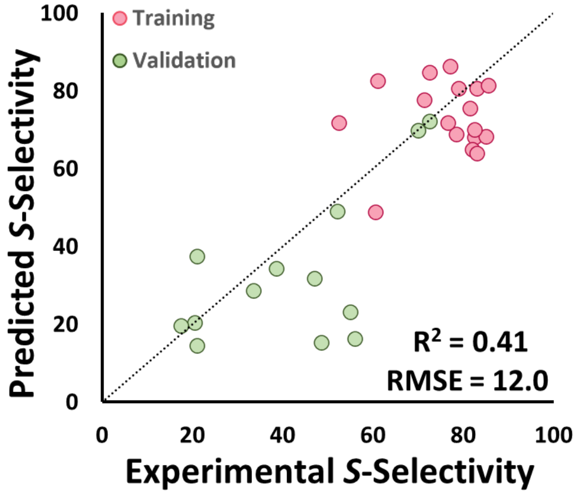

Figure S13. Performance of GFN-2xTB pseudo-TS model on training set, validation set I and II. Depicted R^2 and RMSE are in reference to the validation sets.

Table S6 shows the prediction capabilities of the DFT TS and GFN2-xTB pseudo-TS models with the validation set. L29, L30, L25, and L26 differ only by C2 substitution on the oxaphosphole ring. With only hydrogens at the C2 position on L29, the DFT TS captured the poor selectivity exhibited by this ligand as compared to the others. GFN2-xTB struggled to capture the poor selectivity and instead predicted that it would have similar selectivity to the other ligands.

Table S6. Comparison of the performance of DFT TS and GFN-2xTB pseudo-TS models on select validation set ligands. All values are reported in terms of % *S*-selectivity.

Ligand	Experiment	DFT TS	GFN-2xTB Pseudo-TS
L29 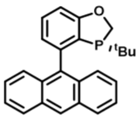	48.6	38.7	15.3
L30 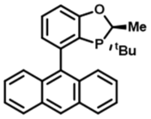	16.5	18.4	19.7
L25 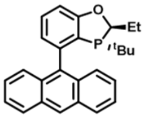	20.5	16.2	20.4
L26 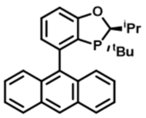	21.2	12.2	14.5

2.4.3 ΔΔG‡

A model using the difference in energy between the Pro-*S* and Pro-*R* transition states was also created. Using the geometry optimizations and single-point energy calculations, ΔΔG‡ was calculated by subtracting the energy of the Pro-*R* transition state from the Pro-*S* energy. Using Gibb's free energy equation, a linear regression was fit to the data. Using this regression, the ΔΔG‡ of validation set I and II was then used to predict the selectivity of these ligands. The resulting performance of this method is shown in Figure S14. Three different methods for single point calculations were tried. All calculations were performed with the 6-311++G(d,p) basis set for light atoms and the def2-TZVP basis set for Pd. The functional used is indicated above the plot in Figure S14.

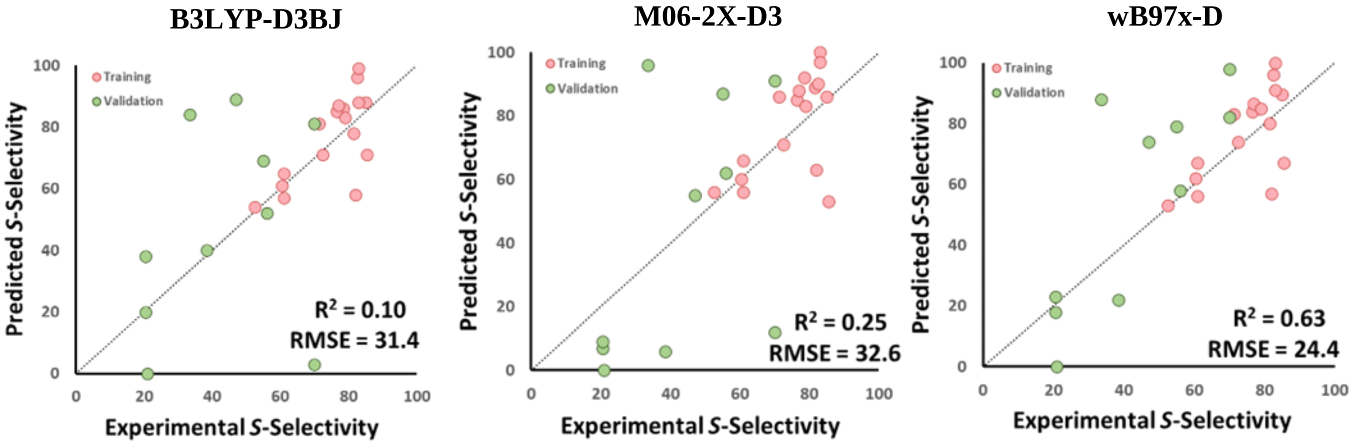

Figure S14. Performance of DFT ΔΔG‡ model on training set, validation set I and II. Depicted R^2 and RMSE are in reference to the validation sets. Three plots refer to different functionals used in the single-point energy calculations.

2.4.4 Other ML Models

Alternative methods to a NN were explored. These methods include random forest (RF), logistic regression, k-Nearest Neighbors (kNN), support vector regression (SVR), decision tree (DT), gradient boosting (GB), and bagging. All methods were implemented in Python using scikit-learn version 1.2.2. Table S7 highlights the performance of these different methods by comparing their R^2 values, RMSE, and slopes.

Table S7. Performance of 6 different ML models for predicting enantioselectivity of the asymmetric Negishi reaction. Shown are model R^2 values, RMSE, and slopes.

	RF	Logistic Regression	kNN	SVR	DT	GB	Bagging
R2	0.51	0.22	0.26	0.52	0.14	0.52	0.39
RMSE	6.63	9.39	8.52	10.5	11.9	6.65	7.35
Slope	0.46	0.38	0.17	0.00	0.44	0.60	0.41

Multivariate linear regression (MLR) was also attempted using the five features that were the most correlated to selectivity (Core 3, Rydberg 4, Bond Length 3-4, Valence 3, Total 3). Figure S15 shows the performance of the MLR model as well as statistics regarding the R^2 and RMSE.

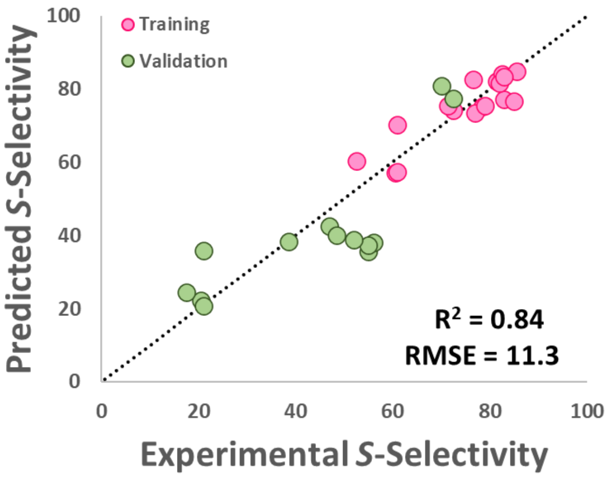

Figure S15. Performance of MLR model on training set, validation set I and II. Depicted R^2 and RMSE are in reference to the validation sets.

2.5 Feature Space

Figure S16 shows two examples of the chemical space of four different features.

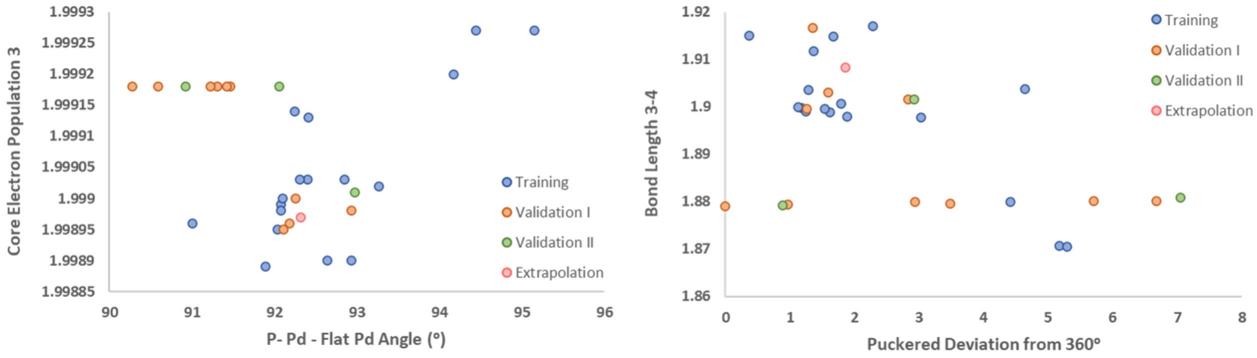

Figure S16. (Left) Chemical space analysis of the core electron population of atom 3 and P-Pd-Flat Pd bond angle. (Right) Chemical space analysis of the bond length between atom 3 and 4 and the puckered deviation from 360°.

These plots show that some of the validation I and II data points (orange and green points, respectively) fall within the chemical space, or cluster, of training points (blue points). Also evident from these plots is that some of the validation set points lie outside the chemical space of the training points. This indicates that this model can extrapolate to predict the selectivity of ligands whose features do not necessarily resemble those of the training set.

3.0 Experimental Details

3.1 General Procedure

All reactions were conducted in oven-dried glassware under an inert atmosphere of nitrogen or argon with magnetic stirring unless otherwise noted. THF (<0.02% water content) and MeOH were purchased from Sigma Aldrich and used directly without further purifications. 1.6 M *Tert*-butyllithium and zinc bromide were purchased Sigma Aldrich and were used directly. $Pd_2(dba)_3$ was purchased from Strem Chemicals and was used directly. Ligands **L18**, **L19**[17], **L20**[17]**, L21**[17], **L22**[17], **L23**[17], **L24**[17], **L25**[18], **L26**[17], **L27**[17], **L28**[17], **L29**[17], **L30**[17], and **L31** were purchased from Zejun Pharmaceuticals and were directly used. (*S*)-2,2'-dimethyl-1,1'-binaphthalene was synthesized using a literature procedure.[19]

General procedure for the asymmetric Negishi cross-coupling.

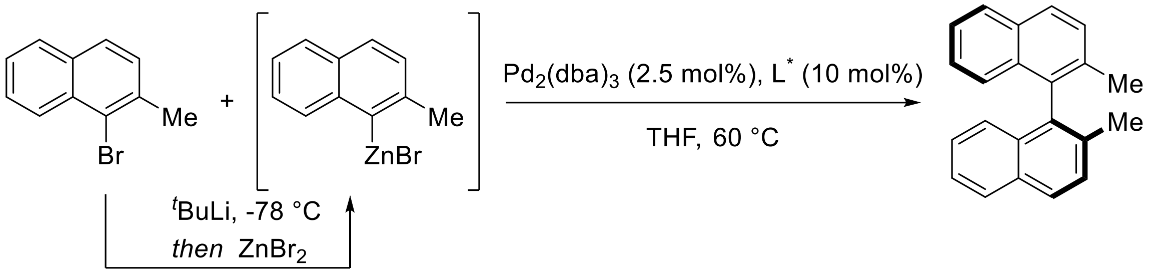

To a 20 mL vial equipped with a magnetic stir bar, 1-bromo-2-methylnapthalene (0.059 g, 0.267 mmol, 1 equiv.) was then added under nitrogen followed by tetrahydrofuran (1 mL, 0.3 M). The reaction was then cooled to –78 °C where a solution of *tert*-butyllithium in pentanes (1.6 M, 0.5 mL, 0.801 mmol 3.0 equiv.) was added dropwise while keeping the reaction at – 78 °C. After 20 min, a solution of zinc bromide (0.12 gm, 0.534 mmol, 2.0 equiv.) in tetrahydrofuran (1 mL) was added dropwise to the reaction while keeping the reaction at – 78 °C. After 5 minutes, the reaction was allowed to warm to room temperature and stirred for further 15 minutes. During this time, to a second Agilent 20 mL vial equipped with a magnetic stir bar, 1-bromo-2-methylnapthalene (0.088 gm, 0.4 mmol, 1.5 equiv.), $Pd_2(dba)_3$ (0.006 gm, 0.007 mmol, 2.5 mol%), and ligand (10 mol%) were added in the glovebox followed by tetrahydrofuran (0.25 mL) under nitrogen. The reaction mixture was stirred at room temperature for 2-3 minutes. The organozinc solution was then cannulated into the second vial. The combined mixture was then placed in a pre-heated 60 °C oil bath and allowed to stir for 16 h. After cooling the reaction to room temperature, the reaction mixture was quenched with methanol (1 mL). 4,4'-di-tertbutylbiphenyl (HPLC internal standard) followed tetrahydrofuran (1 mL) were added. Yields were obtained by HPLC. Calibration curve for 2,2'-dimethyl-1,1'-binaphthalene was obtained by adding a variable amount of 2,2'-dimethyl-1,1'-binaphthalene (9.8 mg, 53.3 mg, 73.8 mg, 124 mg) and 4'4'-di-tert-butylbiphenyl as an internal standard (20.5 mg, 19.3 mg, 23.3 mg, 20.0 mg) in tetrahydrofuran.

3.2 Characterization Data for the Validation Set

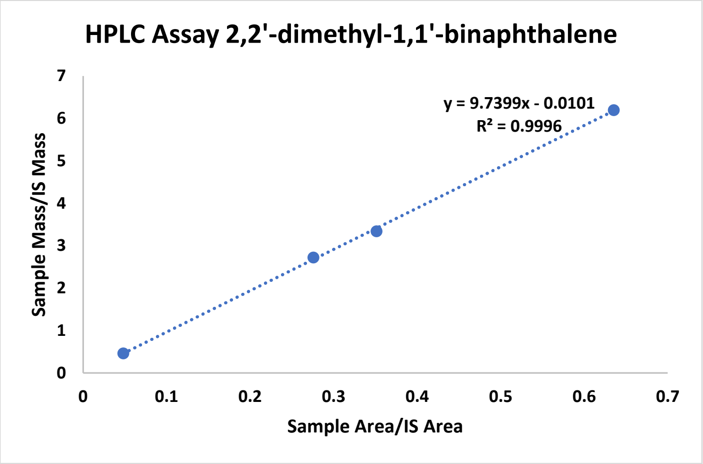

Enantiomeric excess was determined by chiral HPLC on a ChiralPAK OD-3 (3 µm, 4.6 x 250 mm, 100% hexanes, isocratic, 0.8 mL/min, run time: 25 min, 25 °C, Sig 220, 10 Ref = 500, 100, 9.96 min (S), 12.68 min (R)).

Racemate

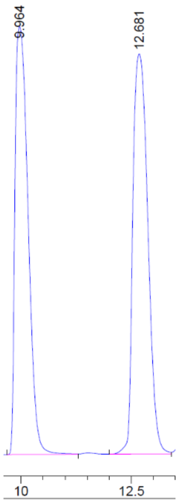

Peak #	RetTime [min]	Type	Width [min]	Area [mAU*s]	Height [mAU]	Area %
1	9.964	BV	0.3295	4.64342e4	2261.58130	48.1741
2	12.681	BV	0.3836	4.99541e4	2114.89307	51.8259

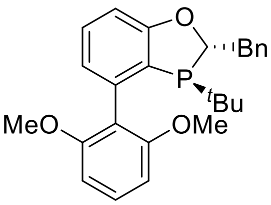

L18
Expt *er* = 72:28 (*S*:*R*)
HPLC Yield: 61%

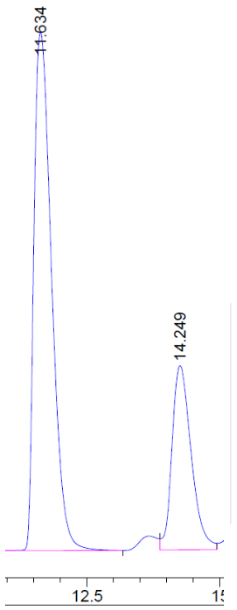

Peak #	RetTime [min]	Type	Width [min]	Area [mAU*s]	Height [mAU]	Area %
1	11.634	BB	0.3615	4.46211e4	1946.20593	72.0070
2	14.249	VV	0.3887	1.73467e4	691.49750	27.9930

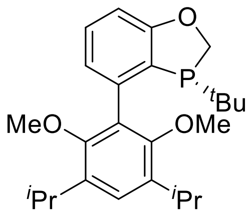

L19

er = 21:79 (*S*:*R*)

HPLC Yield = 59%

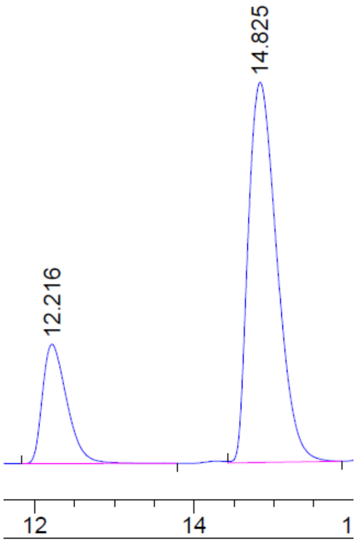

Peak #	RetTime [min]	Type	Width [min]	Area [mAU*s]	Height [mAU]	Area %
1	12.216	VB	0.3294	8134.83105	380.56027	21.1674
2	14.825	VB	0.3976	3.02962e4	1212.05701	78.8326

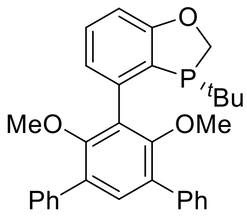

L20

er = 33:67 (*S*:*R*)

HPLC yield = 61%

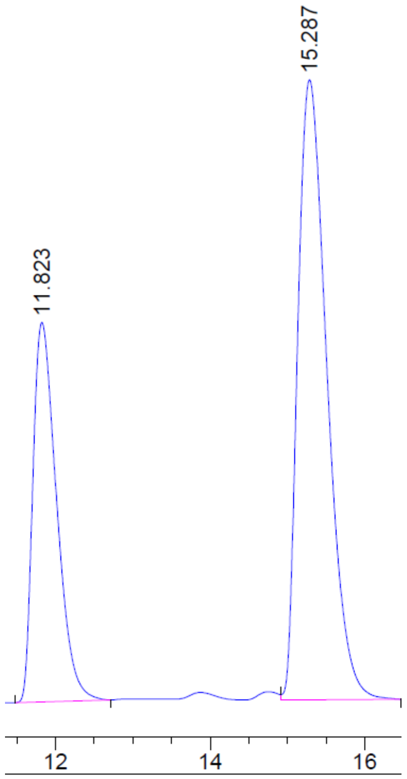

Peak #	RetTime [min]	Type	Width [min]	Area [mAU*s]	Height [mAU]	Area %
1	11.823	BB	0.3306	1.39444e4	654.48340	33.2219
2	15.287	VB	0.4138	2.80292e4	1069.27917	66.7781

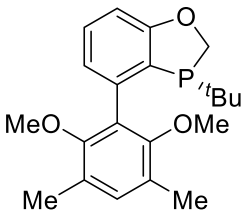

L21

er = 40:60 (*S*:*R*)

HPLC Yield = 67%

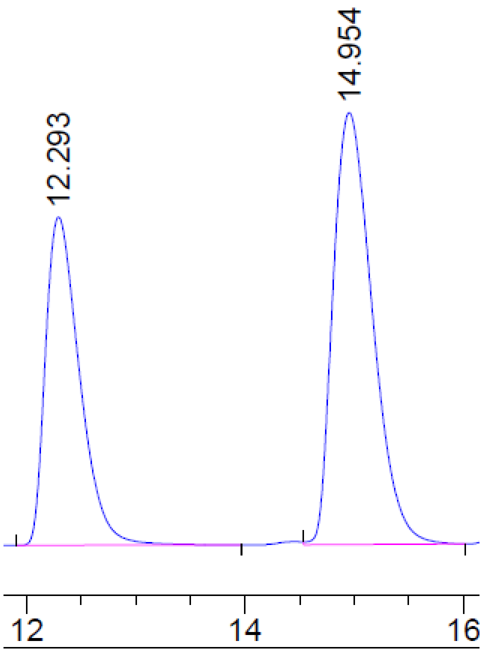

Peak #	RetTime [min]	Type	Width [min]	Area [mAU*s]	Height [mAU]	Area %
1	12.293	BB	0.3384	1.59449e4	731.16785	39.7891
2	14.954	VB	0.3985	2.41287e4	962.54333	60.2109
Totals :				4.00737e4	1693.71118	

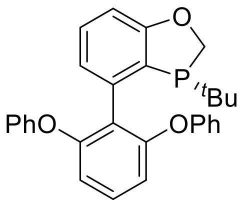

L22

er = 56:44 (*S*:*R*)

HPLC Yield = 57%

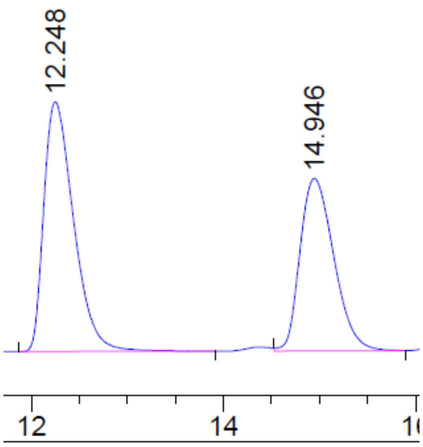

Peak #	RetTime [min]	Type	Width [min]	Area [mAU*s]	Height [mAU]	Area %
1	12.248	BB	0.3366	1.37341e4	634.37445	56.1025
2	14.946	VB	0.3902	1.07463e4	438.02005	43.8975

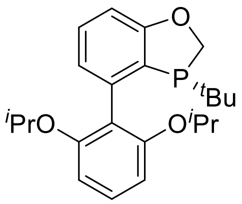

L23

er = 48:52 (*S*:*R*)

HPLC Yield = 58%

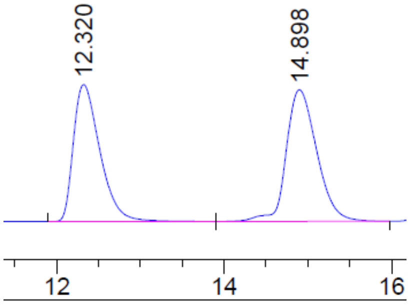

Peak #	RetTime [min]	Type	Width [min]	Area [mAU*s]	Height [mAU]	Area %
1	12.320	BB	0.3397	9839.43848	445.43774	47.5388
2	14.898	BB	0.3960	1.08583e4	427.90964	52.4612

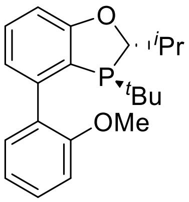

L24

er = 73:27 (*S*:*R*)

HPLC Yield = 98%

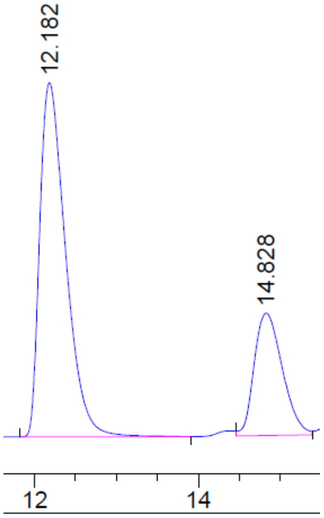

Peak #	RetTime [min]	Type	Width [min]	Area [mAU*s]	Height [mAU]	Area %
1	12.182	BB	0.3387	2.53639e4	1152.84131	72.6285
2	14.828	VV	0.3795	9558.90137	398.99704	27.3715

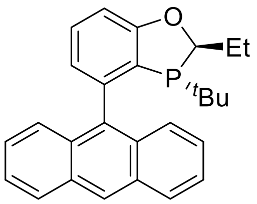

L25

er = 21:79 (*S*:*R*)

HPLC Yield = 78%

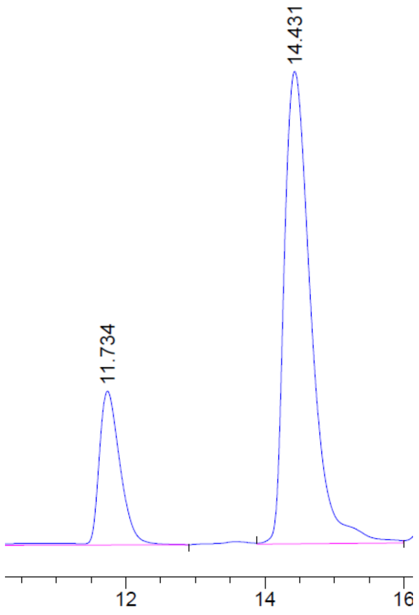

Peak #	RetTime [min]	Type	Width [min]	Area [mAU*s]	Height [mAU]	Area %
1	11.734	BB	0.3269	9173.14453	430.04968	20.5195
2	14.431	VV	0.4175	3.55313e4	1322.15747	79.4805

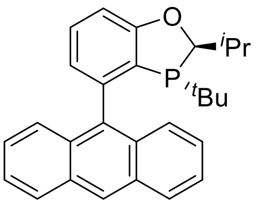

L26

er = 21:79 (*S*:*R*)

HPLC Yield = 70%

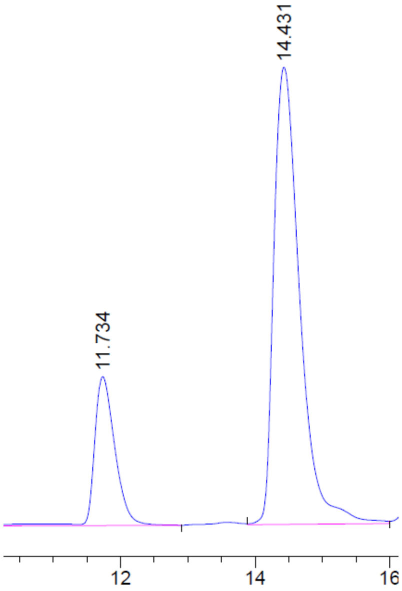

Peak #	RetTime [min]	Type	Width [min]	Area [mAU*s]	Height [mAU]	Area %
1	11.890	BB	0.3176	6731.66016	322.34122	21.1918
2	15.020	VV	0.4011	2.50337e4	957.30219	78.8082

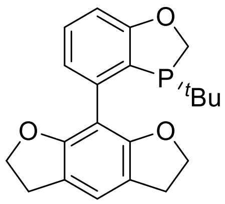

L27

er = 54:46 (*S*:*R*)

HPLC Yield = 86%

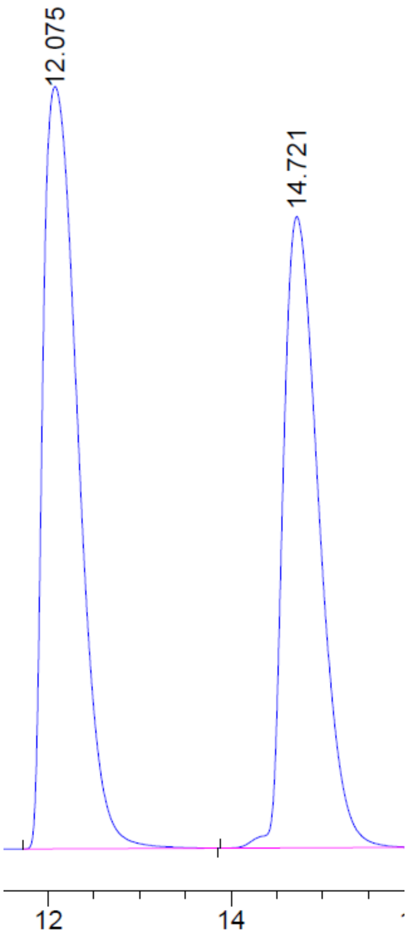

Peak #	RetTime [min]	Type	Width [min]	Area [mAU*s]	Height [mAU]	Area %
1	12.075	BB	0.4208	5.86097e4	2214.26367	54.0837
2	14.721	BB	0.4306	4.97588e4	1833.82141	45.9163

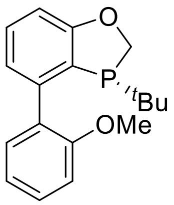

L28

er = 52.0:48.0 (*S*:*R*)

HPLC Yield = 61%

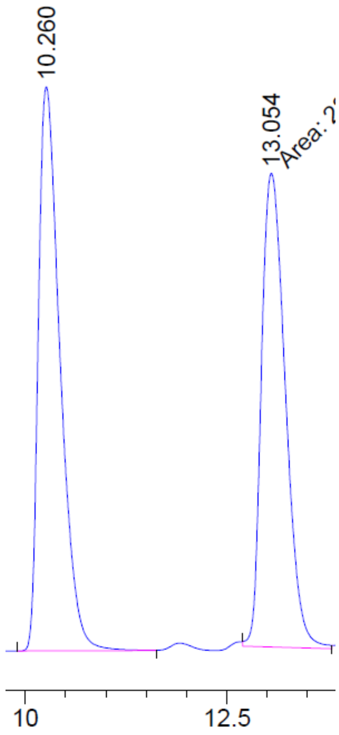

Peak #	RetTime [min]	Type	Width [min]	Area [mAU*s]	Height [mAU]	Area %
1	10.260	BV	0.2865	3.07276e4	1640.82190	51.9610
2	13.054	MM	0.3436	2.84084e4	1378.14807	48.0390

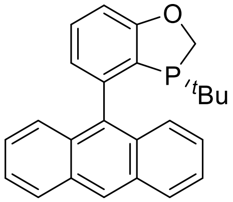

L29

er = 49:51 (*S*:*R*)

HPLC Yield = 72%

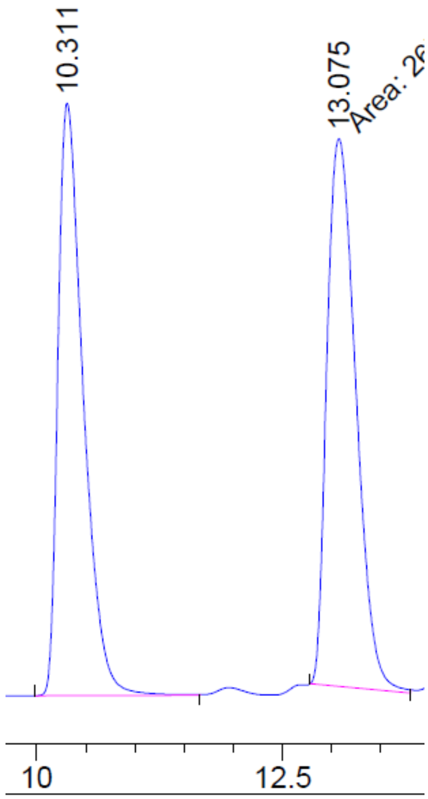

Peak #	RetTime [min]	Type	Width [min]	Area [mAU*s]	Height [mAU]	Area %
1	10.311	BV	0.2701	2.53200e4	1419.60669	48.6319
2	13.075	MM	0.3396	2.67445e4	1312.63330	51.3681

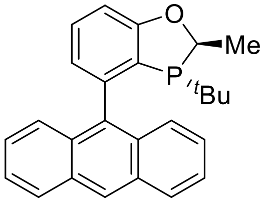

L30

er = 16:84 (*S*:*R*)

HPLC Yield = 51%

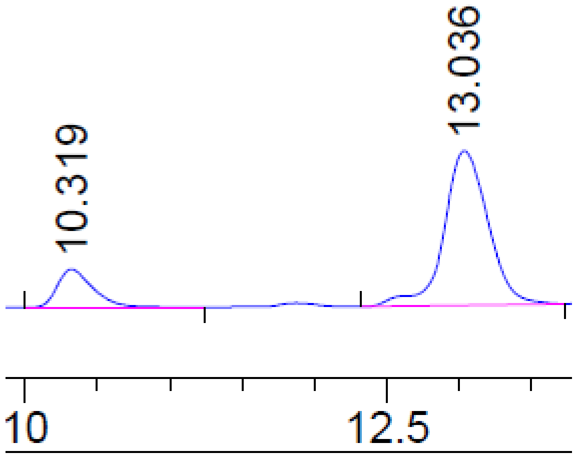

Peak #	RetTime [min]	Type	Width [min]	Area [mAU*s]	Height [mAU]	Area %
1	10.319	BB	0.2572	1110.98804	65.06600	16.4481
2	13.036	BB	0.3313	5643.52539	264.15491	83.5519

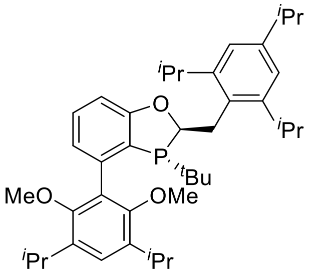

L31
er = 6:94 (*S*:*R*)
HPLC Yield = 78%

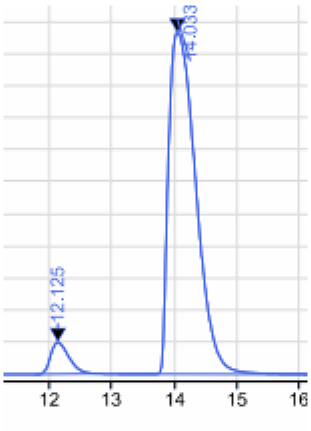

Signal: DAD1A,Sig=220,10 Ref=500,100

RT [min]	Type	Width [min]	Area	Height	Area%
12.125	BM m	0.96	4005.17	199.30	5.84
14.033	MB m	3.18	64574.05	2134.23	94.16
		**Sum**	**68579.22**		

^{1}H-NMR (500 MHz, $CDCl_3$): δ 7.33 (t, J = 7,79 Hz, 1H), 7.12 (s, 1H), 7.05 (dd, J = 7.25, 2.85, 1H), 7.0 (s, 2H), 6.87 (d, J = 8.2 Hz, 1H), 4.93 (dd, J = 10.82, 3.58 Hz, 1H), 3,64 (s, 3H), 3.44-3.38 (m, 1H), 3.35 (s, 3H), 3.29-3.21 (m, 1H), 3.18-3.10 (m, 3H), 2.99 (dt, J = 15.10, 4.71, 1H), 2.90-2.83 (m, 1H), 1.31-1.11 (m, 30H), 0.70 (d, J = 12.08, 9H) ppm.

^{13}C-NMR (126 MHz, $CDCl_3$): δ 163.4, 153.7, 152.8, 147.4, 146.9, 140.3, 140.2, 138.1, 137.3, 130.2, 130.2, 130.1, 129.1, 125.0, 124.8, 123.7, 123.7, 123.4, 121.1, 110.4, 85.0, 84.8, 62.4, 60.7, 34.3, 33.8, 33.5, 31.1, 31.0, 29.5, 27.1, 27.0, 26.8, 26.7, 24.7, 24.6, 24.3, 24.2, 24.2, 23.9, 23.5 ppm.

^{31}P-NMR (202 MHz, $CDCl_3$): δ 10.5 ppm.

HRMS**:** $[M + H]^+$ calcd for ($C_{41}H_{60}O_3P+$), 631.4275; found, 631.4269.

$[\alpha]_D^{22}$ = –22.89 (*c* 0.26, $CHCl_3$)

^{1}H-NMR spectrum of **L31** ($CDCl_3$)

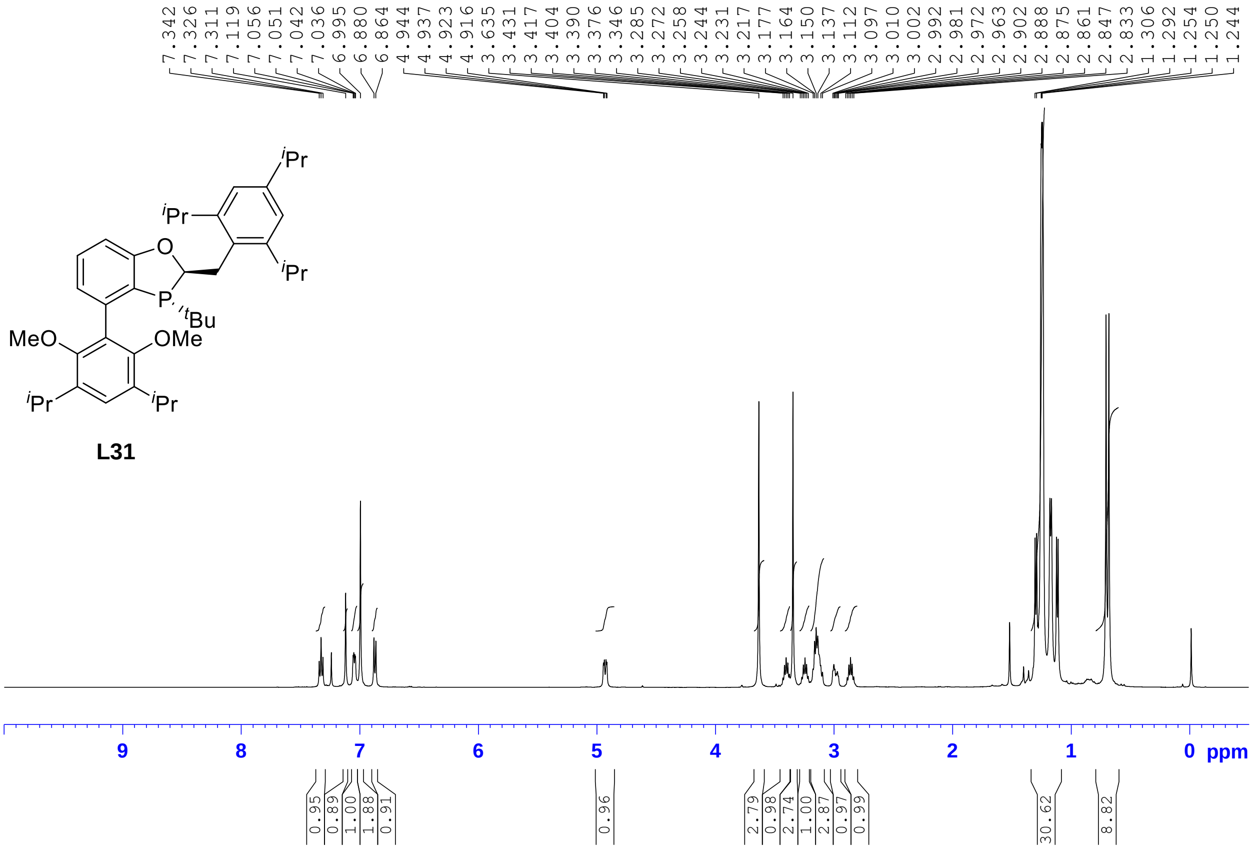

^{13}C-NMR spectrum of **L31** ($CDCl_3$)

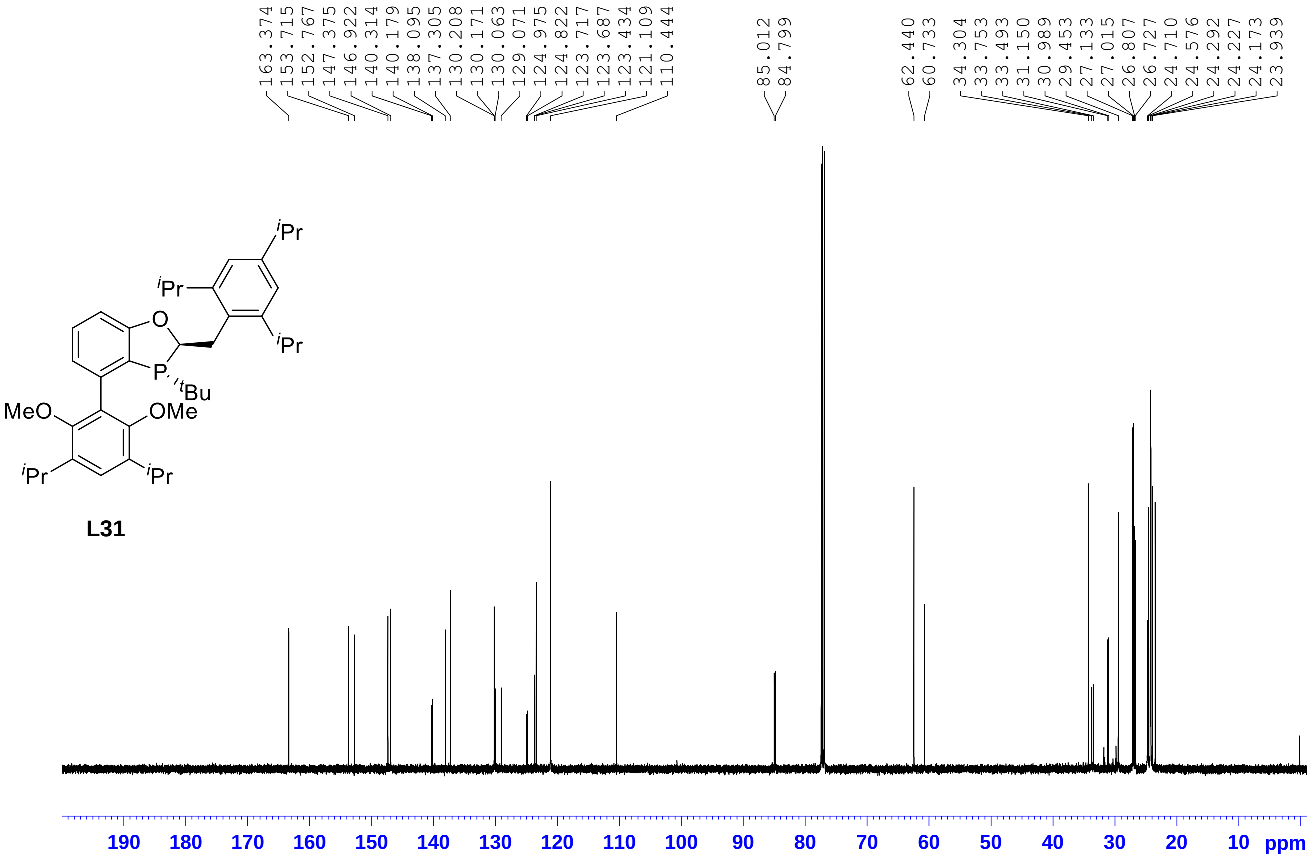

^{31}P-NMR spectrum of **L31** ($CDCl_3$)

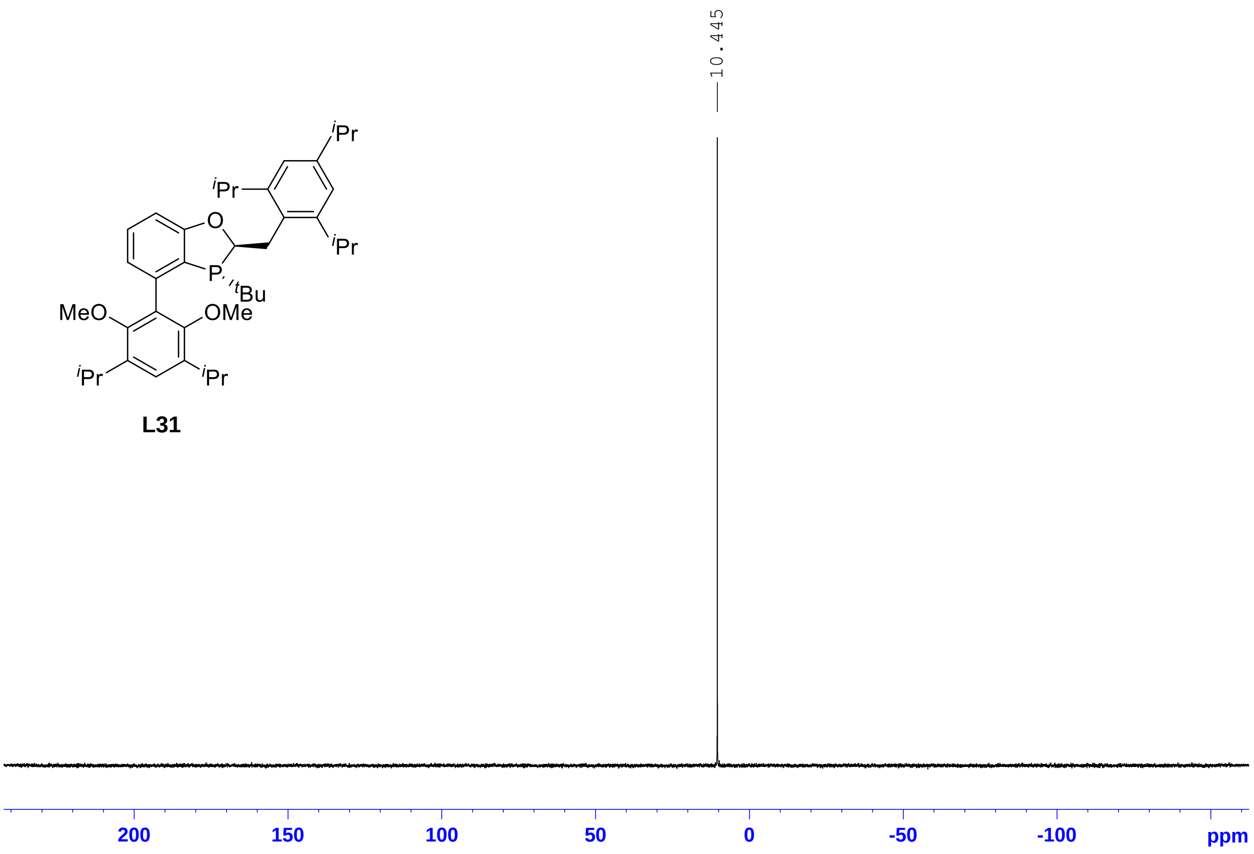

L31

4.0 Computational Coordinates

4.1 Transition State Coordinates

Pro-*S* L1

Pd	0.81133	-0.18600	-0.29301
C	2.44752	1.16551	-0.64754
C	3.48946	1.14939	-1.58545
C	1.89024	2.45001	-0.25033
C	2.39168	3.66817	-0.82828
C	2.66917	-0.44554	0.68028
C	2.93327	-1.83232	0.30415
C	3.18101	0.02021	1.90494
C	3.62257	-2.71332	1.21080
C	3.88564	-0.88088	2.75348
C	2.70754	-3.74845	-1.22917
C	3.82221	-4.08202	0.87432
C	3.36973	-4.59994	-0.31457
H	2.36613	-4.14137	-2.18344
H	4.34717	-4.71260	1.58868
H	3.52788	-5.64717	-0.55784
C	2.50366	-2.41919	-0.92947
H	2.00976	-1.78550	-1.65751
C	4.09288	-2.19893	2.43959
H	4.62572	-2.85581	3.12315
C	3.09028	1.43564	2.43329
H	3.65647	1.51937	3.36625
H	2.06371	1.74767	2.64291
H	3.50137	2.16161	1.72668
H	4.26971	-0.49404	3.69437
C	1.82656	4.92167	-0.46129
C	0.79231	5.00433	0.43961
C	0.28098	3.81689	1.01333
C	0.81365	2.59117	0.67713
C	3.44179	3.59900	-1.77250
H	3.83085	4.51693	-2.20651
C	3.95833	2.38108	-2.12755
H	4.76940	2.33869	-2.85059
H	2.23331	5.81997	-0.92083
H	0.36949	5.96875	0.70872
H	-0.53946	3.86276	1.72447
H	0.39780	1.69525	1.13152
C	4.21493	-0.07952	-2.08905
H	3.57150	-0.72879	-2.69121
H	5.05605	0.21781	-2.72296
H	4.61164	-0.68849	-1.27322
P	-1.26297	-0.43831	-1.47860
C	-2.70607	-1.31378	-0.75048
C	-1.98573	1.02635	-2.47594
C	-1.18736	-1.82138	-2.74965
C	-3.35574	-1.14820	0.48558
C	-3.16340	-2.29743	-1.64633
C	-3.19327	0.62822	-3.34281
C	-2.41197	2.12083	-1.48102
C	-0.84478	1.55695	-3.36658
O	-2.46964	-2.46955	-2.81107
H	-0.93774	-1.47228	-3.75407
C	-4.48896	-1.93262	0.75225
C	-2.86517	-0.21544	1.54718
C	-4.28934	-3.07409	-1.38177
H	-4.02012	0.24975	-2.73365
H	-3.55519	1.51320	-3.88341
H	-2.94467	-0.13474	-4.08727
H	-3.24078	1.78713	-0.84917
H	-1.58313	2.43320	-0.83852
H	-2.75186	3.00310	-2.04067
H	0.02492	1.85709	-2.77351
H	-0.51490	0.81523	-4.10448
H	-1.19687	2.43515	-3.92350
C	-4.95248	-2.87026	-0.17232
H	-4.99929	-1.81064	1.70244
C	-1.73720	-0.55229	2.32454
C	-3.57179	0.95849	1.86940
H	-4.61595	-3.81849	-2.10052
H	-5.82984	-3.46709	0.06272
C	-1.31477	0.27411	3.37662
O	-1.11925	-1.71989	1.99137
C	-3.15425	1.79408	2.91470
O	-4.66925	1.21357	1.09320
C	-2.02868	1.43755	3.65524
H	-0.44871	0.01495	3.97274
C	-0.07538	-2.20495	2.83059
H	-3.69543	2.70049	3.15646
C	-5.44973	2.36343	1.37856
H	-1.70417	2.07775	4.47132
H	0.21887	-3.16584	2.40681
H	0.78989	-1.53363	2.82648
H	-0.43315	-2.34864	3.85838
H	-6.27081	2.35023	0.65947
H	-5.85811	2.33048	2.39702
H	-4.87098	3.28740	1.25055
H	-0.43813	-2.55629	-2.42983

Pro-*R* L1

Pd	-0.76695	0.01658	0.17075
C	-2.31734	1.34118	-0.39624
C	-3.03992	1.52015	-1.58986
C	-1.86831	2.52996	0.32338
C	-2.08964	3.84163	-0.22807
C	-2.83891	-0.49238	0.46673
C	-2.97765	-1.72638	-0.29388
C	-3.71079	-0.27075	1.54268
C	-3.97403	-2.69698	0.07219
C	-4.68745	-1.25715	1.86491
C	-2.26121	-3.26964	-2.07386
C	-4.08561	-3.91883	-0.64906
C	-3.25074	-4.20921	-1.70089
H	-1.59331	-3.48677	-2.90355
H	-4.85069	-4.62723	-0.33876
H	-3.34510	-5.14798	-2.24004
C	-2.13610	-2.07701	-1.39386
H	-1.36335	-1.37363	-1.69497
C	-4.82720	-2.42717	1.16689
H	-5.58812	-3.15328	1.44257
C	-3.74662	0.96232	2.41936
H	-4.59926	0.90859	3.10334
H	-2.84707	1.06810	3.03366
H	-3.84501	1.88070	1.83550
H	-5.35172	-1.05582	2.70192
C	-1.60321	4.99739	0.44495
C	-0.91345	4.89851	1.62868
C	-0.69614	3.62145	2.19529
C	-1.15980	2.48677	1.56654
C	-2.79675	3.96137	-1.44496
H	-2.97803	4.94978	-1.86078
C	-3.25817	2.83859	-2.08170
H	-3.81863	2.94734	-3.00719
H	-1.79476	5.96934	-0.00478
H	-0.54796	5.78974	2.13191
H	-0.16711	3.53139	3.14043
H	-0.99122	1.52277	2.03381
C	-3.66923	0.42694	-2.42675
H	-2.93453	-0.25124	-2.86888
H	-4.24127	0.87120	-3.24731
H	-4.35297	-0.19239	-1.83975
P	1.07009	-1.38423	0.80544
C	2.52642	-1.66841	-0.27932
C	1.76037	-1.16987	2.57760
C	0.73620	-3.23013	0.69784
C	3.34453	-0.76400	-0.98034
C	2.78290	-3.04887	-0.37200
C	2.88188	-2.16835	2.91289
C	2.29706	0.26715	2.71111
C	0.57392	-1.36414	3.54300
O	1.94319	-3.89932	0.28726
H	0.41788	-3.66408	1.64798
C	4.42793	-1.27451	-1.71195
C	3.09357	0.71054	-1.00336
C	3.86117	-3.55395	-1.09625
H	3.74265	-2.04167	2.24891
H	3.22541	-1.99892	3.94245
H	2.54996	-3.20911	2.84123
H	3.18087	0.42150	2.08602
H	1.54376	1.01308	2.43410
H	2.58769	0.45203	3.75448
H	-0.24869	-0.67691	3.31687
H	0.17467	-2.38462	3.51307
H	0.90516	-1.17337	4.57227
C	4.68499	-2.64601	-1.75976
H	5.06601	-0.58389	-2.25429
C	2.07574	1.25417	-1.81338
C	3.92487	1.59765	-0.29345
H	4.03006	-4.62495	-1.13993
H	5.52882	-3.01402	-2.33754
C	1.86790	2.63936	-1.87750
O	1.34273	0.35046	-2.52664
C	3.72851	2.98323	-0.35378
O	4.90997	1.00700	0.45293
C	2.69519	3.48441	-1.14276
H	1.07136	3.05544	-2.48123
C	0.41045	0.84051	-3.48220
H	4.36079	3.66300	0.20395
C	5.82266	1.84038	1.14899
H	2.53074	4.55749	-1.18591
H	0.01473	-0.04305	-3.98660
H	-0.41279	1.38299	-3.00334
H	0.90217	1.48892	-4.21903
H	6.53029	1.16542	1.63412
H	6.36497	2.50436	0.46334
H	5.31754	2.44553	1.91321
H	-0.04099	-3.40958	-0.05298

Pro-*S* L2

Pd	0.76746	-0.18610	-0.32940
C	2.38856	1.15383	-0.75108
C	3.41650	1.08047	-1.70386
C	1.83903	2.46241	-0.42656

C	2.34371	3.64422	-1.07368
C	2.63884	-0.32700	0.69696
C	2.96221	-1.72808	0.43150
C	3.13551	0.24462	1.88533
C	3.70614	-2.50687	1.38772
C	3.88440	-0.56097	2.79115
C	2.79624	-3.76681	-0.94353
C	3.96947	-3.88519	1.14956
C	3.52172	-4.51336	0.01313
H	2.44804	-4.24700	-1.85428
H	4.53707	-4.43339	1.89845
H	3.72791	-5.56699	-0.15523
C	2.53519	-2.42996	-0.73809
H	1.98928	-1.87708	-1.49271
C	4.15992	-1.88473	2.57106
H	4.73022	-2.46277	3.29443
C	2.99401	1.68570	2.32628
H	3.56760	1.84780	3.24420
H	1.95846	1.96698	2.53331
H	3.36752	2.38419	1.57338
H	4.25228	-0.08943	3.69914
C	1.79675	4.92093	-0.76349
C	0.77716	5.06068	0.14698
C	0.25876	3.90882	0.78294
C	0.77070	2.66122	0.49902
C	3.38002	3.51639	-2.02639
H	3.76971	4.40629	-2.51476
C	3.88220	2.27655	-2.32166
H	4.68111	2.18843	-3.05403
H	2.20667	5.79017	-1.27334
H	0.36989	6.04232	0.37463
H	-0.55243	3.99930	1.50043
H	0.34871	1.79288	0.99839
C	4.12965	-0.17736	-2.14947
H	3.46794	-0.86948	-2.67939
H	4.94364	0.07895	-2.83460
H	4.56238	-0.72663	-1.30976
P	-1.37346	-0.43078	-1.46218
C	-2.78361	-1.22042	-0.58850
C	-2.12643	1.08358	-2.37161
C	-1.52877	-1.77536	-2.79641
C	-3.28774	-1.03711	0.71274
C	-3.39535	-2.15983	-1.43742
C	-3.41274	0.75069	-3.14983
C	-2.44922	2.16635	-1.32790
C	-1.04063	1.60187	-3.33530
O	-2.85123	-2.35304	-2.67260
H	-1.49303	-1.31093	-3.78565
C	-0.49727	-2.89416	-2.71848
C	-4.42941	-1.75964	1.09345
C	-2.64952	-0.14207	1.72786
C	-4.52926	-2.87646	-1.05835
H	-4.19794	0.37054	-2.48888
H	-3.78979	1.66879	-3.62018
H	-3.25486	0.01683	-3.94579
H	-3.23869	1.84080	-0.64398
H	-1.56933	2.44611	-0.74348
H	-2.80475	3.06807	-1.84552
H	-0.12451	1.87408	-2.80186
H	-0.78053	0.86452	-4.10508
H	-1.41061	2.49601	-3.85401
C	-5.04403	-2.65626	0.21768
H	-4.82849	-1.61907	2.09300
C	-1.48145	-0.54987	2.40413
C	-3.26250	1.06266	2.12258
H	-4.97500	-3.58772	-1.74599
H	-5.92453	-3.20505	0.54138
C	-0.92677	0.23686	3.42497
O	-0.95847	-1.74539	2.00973
C	-2.71116	1.86082	3.13453
O	-4.41243	1.38230	1.45414
C	-1.54793	1.43380	3.77313
H	-0.02711	-0.07627	3.93959
C	0.08250	-2.32397	2.79258
H	-3.18066	2.79038	3.43204
C	-5.10090	2.56842	1.81672
H	-1.11975	2.04511	4.56305
H	0.28990	-3.29218	2.33536
H	0.99198	-1.71566	2.76875
H	-0.24426	-2.46714	3.83070
H	-5.98414	2.60600	1.17650
H	-5.41511	2.54632	2.86834
H	-4.48787	3.46198	1.64103
H	-0.77683	-3.69206	-3.41550
H	0.49123	-2.51834	-2.99851
H	-0.43235	-3.31677	-1.71084

Pro-R L2

Pd	-0.77044	0.06453	0.31428
C	-2.34461	1.32630	-0.35466
C	-2.99823	1.50184	-1.58877
C	-2.01572	2.51790	0.42339
C	-2.24843	3.83121	-0.12016
C	-2.81470	-0.58194	0.40046

C	-2.83482	-1.76525	-0.44491
C	-3.77432	-0.46846	1.41594
C	-3.81748	-2.79300	-0.23084
C	-4.72990	-1.51056	1.59226
C	-1.90889	-3.14861	-2.25542
C	-3.82056	-3.96159	-1.04342
C	-2.89104	-4.14369	-2.03880
H	-1.15966	-3.28801	-3.03080
H	-4.57927	-4.71699	-0.85042
H	-2.90341	-5.04254	-2.64950
C	-1.88667	-2.00638	-1.48465
H	-1.10972	-1.26559	-1.65581
C	-4.76594	-2.63170	0.80542
H	-5.51653	-3.40167	0.96697
C	-3.92041	0.69708	2.36945
H	-4.82560	0.57581	2.97261
H	-3.07814	0.77458	3.06485
H	-3.99406	1.65264	1.84529
H	-5.46427	-1.39441	2.38583
C	-1.87622	4.99224	0.61382
C	-1.29490	4.89895	1.85469
C	-1.07593	3.62050	2.41718
C	-1.42570	2.48063	1.72659
C	-2.85773	3.94823	-1.38842
H	-3.04374	4.93684	-1.80159
C	-3.22854	2.82131	-2.07362
H	-3.72662	2.92668	-3.03448
H	-2.06963	5.96404	0.16468
H	-1.01743	5.79416	2.40483
H	-0.63662	3.53235	3.40738
H	-1.26132	1.51510	2.19011
C	-3.55778	0.41059	-2.47778
H	-2.79183	-0.25127	-2.88847
H	-4.09176	0.86000	-3.32094
H	-4.26351	-0.22779	-1.93883
P	1.20056	-1.06790	1.14174
C	2.56280	-1.53197	-0.00323
C	2.02091	-0.27269	2.68502
C	1.08694	-2.89102	1.67785
C	3.21947	-0.80431	-1.01576
C	2.92040	-2.87567	0.21325
C	3.34397	-0.95774	3.06946
C	2.28461	1.21446	2.38734
C	1.00936	-0.37310	3.84551
O	2.26976	-3.56697	1.18873
H	1.14336	-2.94164	2.76844
C	4.22220	-1.44490	-1.75895
C	2.93038	0.63062	-1.33145
C	3.92024	-3.50888	-0.52467
H	4.08686	-0.85737	2.27212
H	3.75252	-0.48336	3.97220
H	3.21957	-2.02427	3.28494
H	3.04702	1.33355	1.61565
H	1.37831	1.73836	2.06603
H	2.65215	1.70640	3.29879
H	0.04557	0.07683	3.58290
H	0.82373	-1.40595	4.16021
H	1.40510	0.16401	4.71738
C	4.56811	-2.77523	-1.51470
H	4.73465	-0.88522	-2.53522
C	1.89130	0.99466	-2.20979
C	3.77982	1.64581	-0.85012
H	4.16367	-4.54661	-0.32152
H	5.34701	-3.24652	-2.10840
C	1.67101	2.33808	-2.54837
O	1.15253	-0.03610	-2.71567
C	3.57208	2.98963	-1.18506
O	4.79862	1.22019	-0.03917
C	2.51114	3.31756	-2.02636
H	0.85759	2.62092	-3.20427
C	0.21902	0.25652	-3.74690
H	4.21784	3.76912	-0.80004
C	5.74018	2.17566	0.42269
H	2.33829	4.35873	-2.28452
H	-0.18931	-0.70611	-4.05969
H	-0.59492	0.89532	-3.38468
H	0.71122	0.73796	-4.60198
H	6.47603	1.61220	0.99936
H	6.24192	2.68185	-0.41212
H	5.27085	2.92706	1.07118
C	-0.14604	-3.64015	1.19423
H	-0.08302	-4.68692	1.51253
H	-1.05379	-3.19628	1.61366
H	-0.23086	-3.61036	0.10512

Pro-*S* L3

Pd	0.82526	-0.17697	-0.29536
C	2.37182	1.22282	-0.84617
C	3.42688	1.12600	-1.76722
C	1.73255	2.51893	-0.66382
C	2.17527	3.66164	-1.41871
C	2.67824	-0.11425	0.74072
C	3.10635	-1.50386	0.59556
C	3.10106	0.58827	1.88576

C	3.87839	-2.14573	1.62807
C	3.87540	-0.08619	2.87345
C	3.15530	-3.64161	-0.62784
C	4.26001	-3.51103	1.50177
C	3.90779	-4.25325	0.40113
H	2.88154	-4.21350	-1.51065
H	4.84447	-3.95451	2.30501
H	4.20805	-5.29418	0.31581
C	2.77244	-2.32213	-0.52785
H	2.20397	-1.87405	-1.33456
C	4.24862	-1.40026	2.76868
H	4.83759	-1.87563	3.54953
C	2.84903	2.04826	2.19503
H	3.38157	2.32836	3.10933
H	1.79088	2.27374	2.34970
H	3.19889	2.70295	1.39294
H	4.18282	0.48206	3.74799
C	1.54472	4.92555	-1.24752
C	0.50015	5.09067	-0.37055
C	0.04110	3.97703	0.36962
C	0.63426	2.74189	0.22007
C	3.23718	3.50883	-2.33837
H	3.57860	4.36951	-2.90846
C	3.82569	2.28305	-2.49745
H	4.64533	2.17604	-3.20379
H	1.91134	5.76333	-1.83682
H	0.02852	6.06232	-0.24860
H	-0.78917	4.08494	1.06175
H	0.25252	1.90331	0.79619
C	4.24364	-0.11114	-2.07168
H	3.65523	-0.89368	-2.56034
H	5.06441	0.14397	-2.74902
H	4.68041	-0.55093	-1.17228
P	-1.32293	-0.69964	-1.33622
C	-2.79249	-1.25426	-0.38227
C	-2.01318	0.59677	-2.57694
C	-1.46241	-2.29607	-2.35639
C	-3.33708	-0.80741	0.83532
C	-3.41303	-2.31732	-1.06366
C	-3.26890	0.12432	-3.33242
C	-2.36360	1.87054	-1.78813
C	-0.88001	0.90090	-3.57641
O	-2.81909	-2.78112	-2.20029
H	-1.35245	-2.04016	-3.41444
C	-0.48201	-3.42538	-2.02236
C	-4.52900	-1.39576	1.28903
C	-2.69460	0.22768	1.70320
C	-4.59900	-2.89640	-0.61562
H	-4.09253	-0.10167	-2.64824
H	-3.60324	0.93035	-3.99934
H	-3.08975	-0.75976	-3.95147
H	-3.17812	1.69311	-1.07865
H	-1.50318	2.26366	-1.24124
H	-2.69507	2.64764	-2.49061
H	0.01842	1.26637	-3.06923
H	-0.60209	0.02037	-4.16941
H	-1.21301	1.67469	-4.28056
C	-0.56709	-3.98248	-0.59786
C	-5.15462	-2.41339	0.56770
H	-4.95875	-1.05244	2.22468
C	-1.55567	-0.08889	2.47269
C	-3.28734	1.49329	1.87833
H	-5.05044	-3.70841	-1.17631
H	-1.55312	-4.41629	-0.40370
H	0.18281	-4.76752	-0.45559
H	-0.38322	-3.20754	0.15340
H	-6.07540	-2.85106	0.94434
C	-1.01640	0.83868	3.37704
O	-1.04754	-1.34073	2.29154
C	-2.75344	2.42822	2.77623
O	-4.40055	1.72923	1.12002
C	-1.62308	2.08464	3.51600
H	-0.14134	0.59503	3.96617
C	0.00029	-1.78205	3.15033
H	-3.21112	3.40083	2.90906
C	-5.06240	2.97669	1.25429
H	-1.20796	2.80285	4.21797
H	0.19778	-2.81595	2.86401
H	0.91152	-1.19057	3.01182
H	-0.31251	-1.74576	4.20179
H	-5.91508	2.93441	0.57424
H	-5.42289	3.13368	2.27930
H	-4.41140	3.81279	0.96765
H	-0.67040	-4.23529	-2.74043
H	0.53005	-3.05600	-2.22390

Pro-*R* L3

Pd	-0.75470	0.15094	0.27391
C	-2.25138	1.55397	-0.28915
C	-2.92139	1.83900	-1.49390
C	-1.82272	2.67835	0.53906
C	-1.97755	4.03136	0.07043
C	-2.83568	-0.35495	0.38519
C	-2.96348	-1.48672	-0.51954

C	-3.75635	-0.23067	1.43504
C	-4.00597	-2.45803	-0.32397
C	-4.77457	-1.21514	1.59024
C	-2.19659	-2.82367	-2.43602
C	-4.11402	-3.57830	-1.19544
C	-3.23378	-3.76448	-2.23418
H	-1.48730	-2.96694	-3.24744
H	-4.91503	-4.29190	-1.01458
H	-3.32785	-4.62481	-2.89150
C	-2.07042	-1.73112	-1.60596
H	-1.25246	-1.03345	-1.76611
C	-4.90895	-2.28974	0.75093
H	-5.70424	-3.01630	0.89911
C	-3.79668	0.89100	2.44952
H	-4.69390	0.80196	3.06996
H	-2.93517	0.87293	3.12518
H	-3.81429	1.87536	1.97643
H	-5.47593	-1.09194	2.41209
C	-1.51120	5.12434	0.85341
C	-0.91020	4.92671	2.07246
C	-0.76534	3.60832	2.56179
C	-1.20649	2.53279	1.82212
C	-2.60565	4.25642	-1.17381
H	-2.73244	5.27577	-1.53090
C	-3.06971	3.19496	-1.90498
H	-3.58164	3.38441	-2.84543
H	-1.64849	6.12937	0.46017
H	-0.56097	5.77093	2.66101
H	-0.31059	3.43823	3.53416
H	-1.09663	1.53513	2.23017
C	-3.57889	0.83885	-2.42224
H	-2.87101	0.15488	-2.89617
H	-4.10798	1.36959	-3.21993
H	-4.30787	0.21639	-1.89608
P	1.16552	-1.14017	0.99892
C	2.54073	-1.51134	-0.16360
C	1.98585	-0.54342	2.62962
C	1.00032	-3.00628	1.32878
C	3.22369	-0.69918	-1.09030
C	2.88388	-2.87207	-0.06300
C	3.25093	-1.34298	2.98789
C	2.35475	0.94289	2.47501
C	0.93235	-0.68510	3.74720
O	2.20297	-3.64197	0.83083
H	0.99790	-3.17930	2.40949
C	4.24323	-1.27652	-1.86243
C	2.93498	0.75618	-1.29188
C	3.89941	-3.44270	-0.82939
H	4.02138	-1.23224	2.21852
H	3.66406	-0.96568	3.93327
H	3.05297	-2.41185	3.11753
H	3.15455	1.07616	1.74364
H	1.49780	1.55229	2.17010
H	2.71564	1.32833	3.43890
H	0.01376	-0.13779	3.50925
H	0.66008	-1.72835	3.94305
H	1.33739	-0.27491	4.68160
C	4.57749	-2.62544	-1.73029
H	4.77608	-0.65320	-2.57374
C	1.89190	1.18392	-2.13666
C	3.78322	1.73382	-0.73649
H	4.13241	-4.49651	-0.71691
H	5.36975	-3.04603	-2.34405
C	1.66713	2.54847	-2.37107
O	1.15414	0.19244	-2.71737
C	3.57041	3.09868	-0.96680
O	4.80676	1.25074	0.03534
C	2.50616	3.48724	-1.77753
H	0.85010	2.87857	-2.99984
C	0.22126	0.55878	-3.72517
H	4.21485	3.84833	-0.52459
C	5.74376	2.17190	0.57025
H	2.32948	4.54466	-1.95396
H	-0.18527	-0.37898	-4.10829
H	-0.59362	1.16875	-3.31823
H	0.71369	1.10216	-4.54221
H	6.48294	1.56877	1.10074
H	6.24233	2.74444	-0.22255
H	5.27093	2.86775	1.27564
C	-0.22450	-3.67076	0.70522
H	-1.11705	-3.15148	1.07191
H	-0.20630	-3.51597	-0.37891
C	-0.31438	-5.16763	1.02332
H	-1.19999	-5.60503	0.55000
H	0.56869	-5.70199	0.65936
H	-0.39158	-5.34143	2.10441

Pro-*S* L4

Pd	0.83377	-0.14412	-0.22613
C	2.41606	1.11238	-0.98315
C	3.45578	0.85087	-1.88940
C	1.82086	2.44168	-0.98162
C	2.28961	3.45044	-1.89497
C	2.69989	0.00533	0.77821

C	3.08192	-1.40402	0.83073
C	3.15795	0.84950	1.80826
C	3.84231	-1.91819	1.94045
C	3.92005	0.29697	2.87793
C	3.04975	-3.69429	-0.07631
C	4.17724	-3.29981	2.00839
C	3.79045	-4.17871	1.02636
H	2.75007	-4.37580	-0.86822
H	4.75425	-3.64409	2.86396
H	4.05527	-5.23081	1.08907
C	2.71188	-2.36166	-0.16349
H	2.15232	-2.01328	-1.02365
C	4.24830	-1.03059	2.96076
H	4.82898	-1.40926	3.79862
C	2.95703	2.34634	1.90774
H	3.51187	2.73609	2.76694
H	1.90890	2.62637	2.03988
H	3.31504	2.86794	1.01669
H	4.25522	0.97364	3.66026
C	1.70133	4.74591	-1.89984
C	0.67391	5.06789	-1.04644
C	0.18995	4.08634	-0.15137
C	0.74216	2.82371	-0.12886
C	3.33434	3.13356	-2.79217
H	3.69553	3.89278	-3.48170
C	3.88175	1.87879	-2.77993
H	4.68856	1.64553	-3.47046
H	2.08629	5.47917	-2.60530
H	0.23456	6.06198	-1.05997
H	-0.62716	4.31878	0.52563
H	0.34342	2.08778	0.56403
C	4.22854	-0.44364	-2.02183
H	3.60977	-1.26626	-2.39331
H	5.05052	-0.31372	-2.73248
H	4.65948	-0.76811	-1.07210
P	-1.34600	-0.73953	-1.16292
C	-2.82137	-1.09617	-0.12635
C	-2.01084	0.39157	-2.56899
C	-1.55660	-2.45841	-1.94453
C	-3.33375	-0.45960	1.01857
C	-3.48861	-2.22170	-0.64242
C	-3.29325	-0.13802	-3.23656
C	-2.30603	1.77587	-1.96567
C	-0.88359	0.51085	-3.61335
O	-2.92738	-2.86438	-1.70668
H	-1.45852	-2.35473	-3.02987
C	-0.59640	-3.56734	-1.47529
C	-4.54050	-0.93133	1.56092
C	-2.64215	0.66217	1.72595
C	-4.68857	-2.68602	-0.10710
H	-4.11376	-0.23420	-2.51892
H	-3.60960	0.57652	-4.00832
H	-3.15403	-1.10709	-3.72480
H	-3.11534	1.73004	-1.23004
H	-1.42470	2.21149	-1.48881
H	-2.62136	2.45701	-2.76809
H	0.03419	0.91277	-3.17268
H	-0.64431	-0.45403	-4.07802
H	-1.20132	1.18834	-4.41679
C	-0.71029	-3.88833	0.02216
C	-0.79856	-4.83400	-2.32484
C	-5.21172	-2.01756	0.99830
H	-4.94524	-0.44147	2.44082
C	-1.50533	0.41313	2.52287
C	-3.18556	1.96160	1.72647
H	-5.17604	-3.55258	-0.54184
H	-1.69459	-4.30736	0.25838
H	0.04806	-4.62610	0.30525
H	-0.56028	-3.00150	0.64620
H	-1.81377	-5.22598	-2.20136
H	-0.64136	-4.63285	-3.39187
H	-0.09379	-5.61769	-2.02352
H	-6.14289	-2.36169	1.44069
C	-0.92025	1.43666	3.28379
O	-1.04566	-0.87025	2.51492
C	-2.60567	2.99176	2.48009
O	-4.29950	2.13158	0.95175
C	-1.47888	2.71206	3.25157
H	-0.04668	1.24449	3.89379
C	-0.00535	-1.22782	3.42061
H	-3.02548	3.99018	2.47891
C	-4.91253	3.41027	0.91435
H	-1.02827	3.50503	3.84238
H	0.15081	-2.29811	3.27920
H	0.92562	-0.69637	3.19653
H	-0.30523	-1.03438	4.45858
H	-5.77546	3.30598	0.25418
H	-5.25281	3.72363	1.90996
H	-4.23482	4.17187	0.50727
H	0.41335	-3.18392	-1.66934

Pro-*R* L4

Pd	-0.76603	0.05534	0.21438
C	-1.97438	1.74764	-0.28238

C	-2.66462	2.13084	-1.44993
C	-1.29563	2.79467	0.47805
C	-1.26545	4.15171	-0.00282
C	-2.88950	0.04162	0.54085
C	-3.37062	-1.04220	-0.30267
C	-3.64749	0.38959	1.67016
C	-4.59041	-1.73328	0.02087
C	-4.84564	-0.32660	1.95591
C	-3.14015	-2.54525	-2.23614
C	-5.05167	-2.79942	-0.80097
C	-4.35029	-3.20129	-1.91209
H	-2.56941	-2.86264	-3.10479
H	-5.97950	-3.29470	-0.52303
H	-4.71484	-4.01574	-2.53245
C	-2.67258	-1.51171	-1.45471
H	-1.73324	-1.02916	-1.71244
C	-5.31509	-1.34521	1.16999
H	-6.24033	-1.85973	1.41783
C	-3.33688	1.50162	2.64804
H	-4.15244	1.59958	3.37113
H	-2.42199	1.31461	3.21880
H	-3.21535	2.46685	2.15144
H	-5.41080	-0.03335	2.83726
C	-0.57763	5.16279	0.72479
C	0.07403	4.88089	1.90028
C	0.05323	3.55707	2.39453
C	-0.60413	2.56143	1.70571
C	-1.93007	4.46626	-1.20745
H	-1.91415	5.48923	-1.57625
C	-2.60570	3.48690	-1.88466
H	-3.13814	3.74724	-2.79617
H	-0.58506	6.17421	0.32393
H	0.59188	5.66329	2.44879
H	0.55465	3.31943	3.32897
H	-0.61341	1.55806	2.11302
C	-3.54942	1.25940	-2.31756
H	-3.01027	0.45346	-2.82088
H	-4.01960	1.87163	-3.09320
H	-4.34759	0.78644	-1.73957
P	1.13760	-1.43043	0.86497
C	2.62234	-1.63015	-0.20287
C	1.86132	-0.96030	2.58909
C	1.07744	-3.33279	1.05465
C	3.31355	-0.71867	-1.02465
C	3.08260	-2.95598	-0.11426
C	3.01864	-1.86518	3.05257
C	2.38808	0.48321	2.50869
C	0.69941	-1.04262	3.59867
O	2.38032	-3.82780	0.66085
H	0.98460	-3.54555	2.12402
C	0.00663	-4.15752	0.31981
C	4.46281	-1.16422	-1.69703
C	2.89250	0.70183	-1.23154
C	4.22709	-3.39511	-0.77838
H	3.85661	-1.84015	2.34941
H	3.38691	-1.49916	4.02049
H	2.72189	-2.90884	3.19100
H	3.27277	0.54636	1.86931
H	1.64094	1.18231	2.12505
H	2.67737	0.81820	3.51476
H	-0.13803	-0.39978	3.30761
H	0.31173	-2.06301	3.70483
H	1.04943	-0.72216	4.58887
C	0.02721	-3.96468	-1.20189
C	4.91473	-2.47830	-1.57012
H	5.00213	-0.46439	-2.32740
C	1.82920	1.02226	-2.09863
C	3.63142	1.76001	-0.66636
H	4.54826	-4.42656	-0.67634
H	1.00565	-4.22622	-1.61735
H	-0.72355	-4.60817	-1.67388
H	-0.19859	-2.93147	-1.48083
H	5.80667	-2.79460	-2.10459
C	1.48294	2.35628	-2.35478
O	1.19602	-0.04263	-2.67456
C	3.29162	3.09583	-0.91275
O	4.68471	1.38472	0.12441
C	2.21445	3.37470	-1.75092
H	0.64772	2.60303	-2.99750
C	0.24524	0.21780	-3.69693
H	3.84824	3.90745	-0.46100
C	5.49354	2.39778	0.69966
H	1.93772	4.40855	-1.93612
H	-0.07583	-0.76060	-4.05943
H	-0.62129	0.76698	-3.31115
H	0.69562	0.78235	-4.52401
H	6.27342	1.87447	1.25614
H	5.95743	3.02826	-0.07007
H	4.92015	3.03208	1.38822
C	-1.37852	-3.94290	0.94367
H	-2.12312	-4.57651	0.45022
H	-1.71245	-2.90683	0.84679
H	-1.37345	-4.20111	2.01065
H	0.30072	-5.19961	0.51745

Pro-*S* L5

Pd	0.73613	0.03976	0.07916
C	2.36146	-0.86351	1.13961
C	3.34482	-0.27519	1.95252
C	1.85749	-2.17754	1.51465
C	2.37446	-2.85413	2.67479
C	2.64584	-0.35171	-0.83824
C	2.98774	0.98946	-1.31980
C	3.18005	-1.44982	-1.54736
C	3.81791	1.16507	-2.48556
C	3.99411	-1.22179	-2.69396
C	2.80576	3.44359	-1.22621
C	4.12514	2.46580	-2.97348
C	3.63135	3.59145	-2.36292
H	2.41146	4.32647	-0.73262
H	4.76154	2.54374	-3.85241
H	3.87065	4.58088	-2.74384
C	2.49861	2.19577	-0.72664
H	1.86132	2.13122	0.14918
C	4.31169	0.02634	-3.15662
H	4.94129	0.15314	-4.03420
C	3.02804	-2.91871	-1.21246
H	3.62580	-3.51637	-1.90760
H	1.99556	-3.26755	-1.28691
H	3.36995	-3.14953	-0.20068
H	4.38624	-2.09190	-3.21450
C	1.87945	-4.13863	3.03614
C	0.89779	-4.75980	2.30223
C	0.36340	-4.10121	1.17085
C	0.82385	-2.85575	0.80238
C	3.36996	-2.22121	3.45266
H	3.76779	-2.72867	4.32817
C	3.81987	-0.97705	3.09628
H	4.58399	-0.49585	3.70216
H	2.29846	-4.61818	3.91823
H	0.53097	-5.74202	2.58880
H	-0.42099	-4.57243	0.58455
H	0.39094	-2.36769	-0.06683
C	3.99711	1.06924	1.72205
H	3.27469	1.88922	1.74599
H	4.73961	1.26225	2.50275
H	4.50874	1.12026	0.75693
P	-1.45946	0.74960	0.93658
C	-2.87316	0.85619	-0.23597
C	-2.16944	-0.21891	2.44606
C	-1.87313	2.52416	1.51508
C	-3.26298	0.02300	-1.30197
C	-3.63478	2.00121	0.04632
C	-3.51944	0.31775	2.95938
C	-2.37106	-1.68405	2.02671
C	-1.10489	-0.13553	3.55764
O	-3.20769	2.81968	1.05250
H	-1.91299	2.50084	2.60670
C	-0.94899	3.71619	1.15171
C	-4.43406	0.34470	-2.00760
C	-2.48873	-1.17589	-1.75195
C	-4.79784	2.32043	-0.65089
H	-4.28978	0.28627	2.18250
H	-3.85714	-0.32138	3.78614
H	-3.46502	1.34019	3.34421
H	-3.14627	-1.77950	1.26049
H	-1.44949	-2.13403	1.65423
H	-2.69193	-2.26530	2.90230
H	-0.15094	-0.56215	3.23285
H	-0.91961	0.89593	3.88114
H	-1.44944	-0.69892	4.43492
C	-0.77788	3.89202	-0.36190
C	-1.55450	4.98697	1.77597
C	-5.19242	1.46941	-1.68155
H	-4.74342	-0.29770	-2.82594
C	-1.30933	-1.02702	-2.50929
C	-2.99016	-2.47678	-1.54849
H	-5.35892	3.21229	-0.39128
H	-1.74233	4.10933	-0.83171
H	-0.10668	4.72931	-0.58099
H	-0.35932	2.99189	-0.81928
H	-2.57043	5.13277	1.40266
H	-1.59924	4.90348	2.86809
H	-0.96842	5.87543	1.51981
H	-6.09246	1.69440	-2.24761
C	-0.63777	-2.14469	-3.02755
O	-0.89229	0.25655	-2.70908
C	-2.32098	-3.60050	-2.05217
O	-4.15767	-2.55160	-0.83978
C	-1.15114	-3.41654	-2.78803
H	0.27316	-2.02718	-3.60062
C	0.13097	0.49394	-3.67322
H	-2.70548	-4.59990	-1.88977
C	-4.73276	-3.82788	-0.61138
H	-0.63124	-4.28374	-3.18630
H	0.24222	1.57786	-3.72264
H	1.08402	0.05072	-3.36915
H	-0.16444	0.10671	-4.65686

H	-5.65086	-3.64328	-0.05063
H	-4.97842	-4.33384	-1.55418
H	-4.06909	-4.47045	-0.01830
O	0.29578	3.35455	1.77668
C	1.21083	4.39410	2.07253
H	0.86417	5.03260	2.89622
H	2.13879	3.90452	2.37802
H	1.42395	5.03164	1.20335

Pro-*R* L5

Pd	-0.81329	-0.02800	-0.21279
C	-2.50967	-1.26906	0.16680
C	-3.29855	-1.50133	1.31086
C	-2.19949	-2.41292	-0.68737
C	-2.61152	-3.74173	-0.31533
C	-2.81435	0.70822	-0.49846
C	-2.92202	1.81516	0.43950
C	-3.63734	0.71893	-1.63535
C	-3.84964	2.88833	0.19892
C	-4.53707	1.80541	-1.83677
C	-2.22415	2.99805	2.47988
C	-3.94289	3.97518	1.11257
C	-3.15345	4.03557	2.23564
H	-1.58460	3.04174	3.35747
H	-4.65756	4.76604	0.89539
H	-3.23491	4.87121	2.92555
C	-2.11473	1.93602	1.60946
H	-1.38395	1.15567	1.80710
C	-4.65500	2.85213	-0.96148
H	-5.36191	3.65715	-1.14703
C	-3.69752	-0.34879	-2.70560
H	-4.49735	-0.11868	-3.41616
H	-2.76825	-0.41951	-3.27946
H	-3.89644	-1.33928	-2.29047
H	-5.16187	1.78494	-2.72638
C	-2.28044	-4.85801	-1.13328
C	-1.56580	-4.70657	-2.29617
C	-1.15756	-3.41076	-2.68547
C	-1.46342	-2.31565	-1.90748
C	-3.35365	-3.92116	0.87158
H	-3.67189	-4.92093	1.15777
C	-3.68536	-2.83407	1.63485
H	-4.28311	-2.98143	2.53102
H	-2.61532	-5.84232	-0.81288
H	-1.32393	-5.56693	-2.91458
H	-0.60186	-3.27220	-3.60910
H	-1.14798	-1.33294	-2.23545
C	-3.86281	-0.46328	2.25875
H	-3.09736	0.08524	2.81249
H	-4.51105	-0.95089	2.99330
H	-4.46186	0.28499	1.73313
P	1.47168	0.81689	-0.76579
C	2.90461	0.47877	0.33691
C	2.06032	0.20716	-2.49818
C	2.04875	2.63938	-0.86484
C	3.22840	-0.63660	1.13322
C	3.77826	1.57965	0.31433
C	3.46798	0.69386	-2.89108
C	2.07447	-1.33127	-2.48197
C	1.02881	0.71396	-3.52552
O	3.42424	2.66092	-0.43457
H	2.05848	2.90609	-1.92499
C	1.26435	3.74586	-0.10899
C	4.44068	-0.62170	1.84117
C	2.34515	-1.83426	1.28615
C	4.98287	1.59246	1.01503
H	4.22466	0.36126	-2.17422
H	3.72802	0.26814	-3.86959
H	3.53958	1.78174	-2.97997
H	2.85811	-1.71304	-1.82212
H	1.12075	-1.75661	-2.15979
H	2.28109	-1.70052	-3.49636
H	0.01525	0.37685	-3.28449
H	1.00728	1.80866	-3.58935
H	1.28734	0.33505	-4.52310
C	1.19149	3.47097	1.39424
C	5.30582	0.47142	1.77637
H	4.69793	-1.48084	2.45255
C	1.23298	-1.80073	2.15071
C	2.67910	-3.06107	0.67945
H	5.62700	2.46411	0.96535
H	2.19993	3.43626	1.81482
H	0.64299	4.28099	1.88383
H	0.68056	2.52869	1.60436
H	6.23586	0.45542	2.33847
C	0.44713	-2.94213	2.36132
O	1.00396	-0.60536	2.77253
C	1.89740	-4.20570	0.87874
O	3.80653	-3.04469	-0.09748
C	0.78498	-4.12680	1.71366
H	-0.42453	-2.90936	3.00219
C	0.05520	-0.57242	3.82852
H	2.14303	-5.14250	0.39432
C	4.22325	-4.25413	-0.71041

H	0.16757	-5.00787	1.86289
H	0.10153	0.43852	4.23794
H	-0.96044	-0.77241	3.46775
H	0.30977	-1.29605	4.61400
H	5.14261	-4.01312	-1.24740
H	4.43150	-5.03214	0.03552
H	3.47467	-4.62689	-1.42160
C	-0.12614	3.91596	-0.73244
H	-0.65224	4.73320	-0.22995
H	-0.72387	3.00842	-0.62831
C	2.39662	5.48315	-1.43305
H	3.20106	4.88318	-1.88249
H	1.56776	5.56344	-2.15146
H	2.78465	6.48934	-1.24890
H	-0.06477	4.15397	-1.80091
O	1.99504	4.98641	-0.17323

Pro-*S* L6

Pd	-0.94270	0.66320	-0.35680
C	-2.89954	1.53054	-0.57944
C	-3.39109	2.47405	-1.49432
C	-3.79434	0.47574	-0.12584
C	-5.14569	0.40776	-0.61607
C	-1.48860	2.43029	0.69731
C	-0.41039	3.32050	0.27356
C	-2.06379	2.64891	1.96382
C	0.09412	4.33604	1.16132
C	-1.54387	3.68032	2.79846
C	1.28995	4.04445	-1.35558
C	1.17995	5.16662	0.76594
C	1.77659	5.02679	-0.46307
H	1.74648	3.93122	-2.33504
H	1.52909	5.92005	1.46867
H	2.60759	5.66474	-0.75123
C	0.23831	3.23059	-0.99704
H	-0.12594	2.49420	-1.70401
C	-0.50189	4.49074	2.43199
H	-0.13257	5.26110	3.10486
C	-3.23987	1.90956	2.56489
H	-3.51877	2.37336	3.51622
H	-3.02322	0.85705	2.76463
H	-4.11676	1.93478	1.91332
H	-2.00642	3.82228	3.77217
C	-6.01695	-0.62902	-0.17947
C	-5.59695	-1.58955	0.70869
C	-4.26852	-1.54726	1.19124
C	-3.40474	-0.55444	0.78181
C	-5.59043	1.37856	-1.54195
H	-6.61180	1.33528	-1.91244
C	-4.73424	2.36324	-1.95731
H	-5.08685	3.10869	-2.66601
H	-7.03104	-0.64399	-0.57309
H	-6.27384	-2.37492	1.03485
H	-3.91553	-2.30268	1.88779
H	-2.38702	-0.55166	1.16295
C	-2.63348	3.65911	-2.05241
H	-1.80618	3.35918	-2.70321
H	-3.30539	4.28068	-2.65225
H	-2.21222	4.28876	-1.26561
P	0.11087	-1.20337	-1.52394
C	1.40756	-2.25259	-0.75219
C	-1.06307	-2.43923	-2.41350
C	1.35466	-0.81864	-2.90652
C	1.51844	-2.79674	0.54054
C	2.43166	-2.48705	-1.68836
C	-0.32968	-3.47928	-3.28017
C	-1.87584	-3.18130	-1.33827
C	-2.00991	-1.59675	-3.29086
O	2.32352	-1.88984	-2.91111
H	0.83853	-0.86470	-3.86982
C	2.09653	0.52918	-2.81650
C	2.62409	-3.61363	0.82676
C	0.53622	-2.53138	1.63741
C	3.52334	-3.30505	-1.40601
H	0.35687	-4.09063	-2.68659
H	-1.07393	-4.15318	-3.72539
H	0.23843	-3.03022	-4.09999
H	-1.23293	-3.80244	-0.70680
H	-2.43578	-2.49384	-0.69978
H	-2.60148	-3.84283	-1.83125
H	-2.56710	-0.86513	-2.69761
H	-1.47315	-1.05695	-4.08124
H	-2.73386	-2.25861	-3.78406
C	3.60183	-3.87206	-0.13553
H	2.71506	-4.04233	1.81979
C	0.53447	-1.30251	2.32935
C	-0.32986	-3.54911	2.08406
H	4.28989	-3.46362	-2.15719
H	4.44820	-4.50569	0.11583
C	-0.32799	-1.08938	3.41576
O	1.42529	-0.36552	1.89067
C	-1.19613	-3.34473	3.16679
O	-0.25806	-4.72538	1.39053
C	-1.18205	-2.11288	3.81851

H	-0.33463	-0.14353	3.94202
C	1.55437	0.84725	2.63219
H	-1.86222	-4.12843	3.50613
C	-1.08397	-5.80509	1.79636
H	-1.84807	-1.94862	4.66132
H	2.35500	1.40544	2.14606
H	0.63120	1.43447	2.60179
H	1.83275	0.64076	3.67347
H	-0.84659	-6.62942	1.12149
H	-0.87125	-6.10880	2.82962
H	-2.14921	-5.55656	1.70407
H	2.71860	0.61977	-3.71631
H	1.33695	1.32047	-2.86573
C	2.95476	0.69476	-1.59372
H	2.44523	0.65755	-0.63257
C	4.28373	0.87358	-1.63967
H	4.76127	0.95159	-2.61795
C	5.19006	0.99980	-0.48667
C	4.87594	0.47296	0.78080
C	6.42697	1.64850	-0.64695
C	5.75651	0.62252	1.85056
H	3.95292	-0.08283	0.91861
C	7.30734	1.79802	0.42410
H	6.69289	2.04744	-1.62351
C	6.97354	1.28924	1.68055
H	5.49873	0.20187	2.81939
H	8.25510	2.30956	0.27640
H	7.65968	1.39888	2.51619

Pro-*R* L6

Pd	-0.80926	-0.80556	0.18215
C	-1.92760	-2.57239	-0.21325
C	-2.14737	-3.34801	-1.36756
C	-3.06291	-2.32728	0.67298
C	-4.38378	-2.77047	0.30842
C	0.09475	-2.74193	0.36321
C	1.17556	-2.70965	-0.60994
C	0.21244	-3.60182	1.46431
C	2.34213	-3.53160	-0.42753
C	1.38526	-4.39873	1.60489
C	2.21926	-1.83569	-2.65851
C	3.40767	-3.48230	-1.37011
C	3.35540	-2.65593	-2.46706
H	2.17162	-1.17116	-3.51753
H	4.27313	-4.11868	-1.19816
H	4.17734	-2.62656	-3.17707
C	1.17574	-1.86357	-1.75947
H	0.32146	-1.20920	-1.91378
C	2.41622	-4.37739	0.70300
H	3.29150	-5.00732	0.84240
C	-0.82020	-3.79748	2.55263
H	-0.52210	-4.62334	3.20600
H	-0.93245	-2.91128	3.18595
H	-1.80732	-4.03175	2.14821
H	1.44854	-5.05683	2.46821
C	-5.49779	-2.48824	1.14767
C	-5.35000	-1.79608	2.32466
C	-4.06008	-1.36650	2.71166
C	-2.96597	-1.62466	1.91507
C	-4.55544	-3.49512	-0.89104
H	-5.54891	-3.83880	-1.16964
C	-3.46975	-3.77965	-1.67629
H	-3.61078	-4.36711	-2.58034
H	-6.47695	-2.84283	0.83282
H	-6.20892	-1.59047	2.95794
H	-3.92488	-0.83469	3.64977
H	-1.98805	-1.29608	2.24532
C	-1.09791	-3.84371	-2.34086
H	-0.61420	-3.04147	-2.90373
H	-1.55987	-4.52053	-3.06632
H	-0.30056	-4.39329	-1.83377
P	0.08598	1.38127	0.76138
C	-0.12062	2.82861	-0.35301
C	-0.38840	2.00523	2.51397
C	1.95697	1.70899	0.74696
C	-1.22663	3.27651	-1.10053
C	1.09003	3.54095	-0.42368
C	0.19404	3.39109	2.84580
C	-1.92358	2.07709	2.60122
C	0.13600	0.95997	3.51915
O	2.15024	3.07124	0.29568
H	2.34469	1.66903	1.76835
C	-1.08635	4.44572	-1.86414
C	-2.54673	2.57185	-1.12488
C	1.22677	4.70315	-1.18056
H	-0.16463	4.15392	2.14775
H	-0.12734	3.68423	3.85443
H	1.28812	3.40617	2.83151
H	-2.31909	2.86679	1.95725
H	-2.40039	1.13233	2.32217
H	-2.21627	2.30993	3.63461
H	-0.25752	-0.03987	3.30709
H	1.23025	0.89392	3.52286
H	-0.17641	1.24065	4.53357

C	0.11871	5.14940	-1.89780
H	-1.93669	4.79962	-2.43851
C	-2.74917	1.43600	-1.93392
C	-3.65153	3.09599	-0.42587
H	2.17811	5.22455	-1.20427
H	0.19873	6.04993	-2.50098
C	-4.00307	0.81150	-1.99982
O	-1.66363	1.01723	-2.64896
C	-4.90857	2.48186	-0.48694
O	-3.39958	4.22604	0.30592
C	-5.06391	1.33997	-1.26984
H	-4.15214	-0.07778	-2.59882
C	-1.85492	-0.00429	-3.61859
H	-5.75222	2.87735	0.06500
C	-4.47812	4.84369	0.98985
H	-6.03335	0.85151	-1.31247
H	-0.89182	-0.12158	-4.11892
H	-2.14180	-0.95393	-3.15246
H	-2.61432	0.28669	-4.35615
H	-4.05842	5.73297	1.46384
H	-5.27532	5.14270	0.29700
H	-4.89850	4.18601	1.76186
C	2.77040	0.76531	-0.14789
H	2.58221	-0.26717	0.16190
H	2.39110	0.85727	-1.17532
C	4.24323	1.06828	-0.10604
H	4.51725	2.09874	-0.32509
C	5.18562	0.15585	0.17803
H	4.86054	-0.86511	0.38274
C	6.64167	0.35963	0.23792
C	7.26285	1.59988	-0.00529
C	7.46617	-0.73481	0.55507
C	8.64638	1.73440	0.06711
H	6.65774	2.46703	-0.25336
C	8.85249	-0.60213	0.62789
H	7.00666	-1.70200	0.74650
C	9.45052	0.63473	0.38404
H	9.10184	2.70268	-0.12482
H	9.46468	-1.46562	0.87540
H	10.53031	0.74350	0.43958

Pro-*S* L7

Pd	-1.36974	-0.75140	-0.06660
C	-2.39706	-2.38746	0.87177
C	-1.91452	-3.58898	1.41319
C	-3.53549	-1.74738	1.51523
C	-4.13854	-2.33701	2.68104
C	-2.15001	-2.37985	-1.20774
C	-0.94049	-2.78653	-1.92080
C	-3.38907	-2.60793	-1.83787
C	-1.01705	-3.32859	-3.25288
C	-3.41552	-3.16747	-3.14828
C	1.51133	-2.97525	-2.09514
C	0.16822	-3.67041	-3.96293
C	1.41189	-3.49479	-3.40668
H	2.48776	-2.84651	-1.63529
H	0.06290	-4.07786	-4.96613
H	2.30876	-3.75956	-3.96018
C	0.37708	-2.64104	-1.38792
H	0.47664	-2.26099	-0.37913
C	-2.28617	-3.50842	-3.84500
H	-2.35463	-3.92575	-4.84688
C	-4.76270	-2.35020	-1.25657
H	-5.52879	-2.72679	-1.94154
H	-4.96278	-1.28816	-1.09328
H	-4.90163	-2.85048	-0.29501
H	-4.38768	-3.32854	-3.60780
C	-5.24468	-1.70458	3.31471
C	-5.76038	-0.52223	2.84132
C	-5.17279	0.07909	1.70432
C	-4.09932	-0.51252	1.07422
C	-3.61138	-3.54545	3.19050
H	-4.06581	-3.99872	4.06818
C	-2.53995	-4.13324	2.57198
H	-2.14457	-5.06550	2.96807
H	-5.67268	-2.18312	4.19297
H	-6.60664	-0.05124	3.33461
H	-5.56238	1.01777	1.31975
H	-3.66419	-0.02411	0.20636
C	-0.77206	-4.41871	0.86901
H	0.19387	-3.91106	0.95458
H	-0.69327	-5.35562	1.42904
H	-0.91016	-4.67209	-0.18461
P	-0.19399	1.14610	0.92060
C	0.03840	2.69102	-0.04554
C	-0.69379	1.72813	2.68194
C	1.69883	1.04005	1.09239
C	-0.85784	3.43753	-0.83377
C	1.37006	3.12516	0.07798
C	0.21176	2.84351	3.23592
C	-2.13999	2.24915	2.62962
C	-0.62479	0.48568	3.59164
O	2.23267	2.35006	0.79582
H	1.95624	0.84214	2.13562

C	2.35514	-0.02036	0.19291
C	-0.40091	4.63094	-1.41483
C	-2.26124	3.00808	-1.12467
C	1.82232	4.31149	-0.49837
H	0.18673	3.73605	2.60299
H	-0.15091	3.13218	4.23159
H	1.25564	2.53453	3.34573
H	-2.21983	3.14896	2.01239
H	-2.83279	1.49614	2.24632
H	-2.46305	2.51089	3.64674
H	-1.27981	-0.31419	3.23203
H	0.39299	0.08373	3.67124
H	-0.94579	0.75836	4.60556
C	0.91411	5.06472	-1.23964
H	-1.08820	5.21544	-2.01823
C	-2.51374	1.98945	-2.06659
C	-3.36047	3.69288	-0.57176
H	2.85558	4.61621	-0.36941
H	1.24055	5.99188	-1.70331
C	-3.82667	1.64645	-2.42351
O	-1.41064	1.39460	-2.60208
C	-4.67619	3.35193	-0.91494
O	-3.04673	4.69451	0.30537
C	-4.88990	2.33080	-1.83957
H	-4.01837	0.86057	-3.14316
C	-1.58181	0.51589	-3.71114
H	-5.52036	3.87623	-0.48425
C	-4.10198	5.44592	0.88366
H	-5.90756	2.06666	-2.11452
H	-0.57512	0.22320	-4.01189
H	-2.14685	-0.37944	-3.43392
H	-2.08097	1.03016	-4.54262
H	-3.62124	6.18866	1.52286
H	-4.69892	5.95763	0.11755
H	-4.76089	4.81472	1.49397
H	1.89639	-0.97715	0.45246
H	2.06138	0.16163	-0.84351
C	3.87042	-0.12164	0.32718
C	4.43993	-0.86669	1.38710
C	4.72708	0.50121	-0.61696
C	5.83363	-0.98914	1.46834
C	6.11096	0.34200	-0.49205
C	6.69066	-0.39890	0.54196
H	6.26659	-1.56882	2.28088
H	6.75967	0.81272	-1.22494
C	3.60683	-1.57750	2.45840
H	2.54957	-1.34626	2.30329
C	3.95389	-1.09103	3.87979
H	4.98635	-1.33760	4.15189
H	3.84052	-0.00438	3.96707
H	3.29488	-1.56445	4.61772
C	3.74059	-3.11019	2.35237
H	3.10692	-3.60618	3.09772
H	3.44122	-3.46668	1.36001
H	4.77400	-3.43379	2.52223
C	4.18792	1.30173	-1.80608
H	3.19203	1.66878	-1.54984
C	4.04972	0.40353	-3.05318
H	5.02647	0.00521	-3.35437
H	3.38669	-0.44891	-2.86618
H	3.64015	0.97144	-3.89781
C	5.02134	2.55423	-2.13380
H	5.17881	3.17087	-1.24209
H	6.00427	2.30603	-2.55075
H	4.49805	3.16187	-2.88123
C	8.20085	-0.55722	0.66236
H	8.38939	-1.18541	1.54408
C	8.80261	-1.28291	-0.55617
H	9.87996	-1.43860	-0.42156
H	8.33207	-2.26082	-0.70660
H	8.66316	-0.70083	-1.47497
C	8.90211	0.79367	0.90160
H	8.76473	1.46921	0.04886
H	8.50309	1.29439	1.79056
H	9.98037	0.65118	1.04366

Pro-*R* L7

Pd	1.48473	-0.77444	-0.20558
C	2.99008	-2.18772	0.29648
C	3.48416	-2.68751	1.51610
C	3.95057	-1.88781	-0.76193
C	5.36534	-1.99615	-0.51510
C	1.01877	-2.86189	0.04444
C	0.06635	-2.87858	1.14440
C	1.00559	-3.92990	-0.86448
C	-0.83987	-3.98433	1.30479
C	0.08702	-5.00166	-0.67107
C	-0.96943	-1.85815	3.12781
C	-1.77388	-3.99668	2.37852
C	-1.84322	-2.96070	3.27841
H	-1.02196	-1.02851	3.82830
H	-2.44230	-4.85043	2.46555
H	-2.56213	-2.98229	4.09299
C	-0.05326	-1.82367	2.09873

H	0.60202	-0.96151	1.99861
C	-0.79670	-5.04856	0.37475
H	-1.47184	-5.89204	0.49656
C	1.91648	-4.08455	-2.06243
H	1.73135	-5.04588	-2.55159
H	1.75299	-3.30410	-2.81205
H	2.97298	-4.04945	-1.78583
H	0.10770	-5.82060	-1.38607
C	6.30334	-1.65726	-1.53024
C	5.89404	-1.22533	-2.76799
C	4.50986	-1.12695	-3.03823
C	3.58154	-1.44677	-2.07166
C	5.80724	-2.44967	0.74654
H	6.87401	-2.53994	0.93760
C	4.89189	-2.79296	1.70604
H	5.24525	-3.17217	2.66194
H	7.36212	-1.75429	-1.29977
H	6.62134	-0.97256	-3.53492
H	4.17090	-0.80344	-4.01886
H	2.52749	-1.37421	-2.31188
C	2.66652	-3.20562	2.68135
H	2.02357	-2.44931	3.13617
H	3.33521	-3.58365	3.46106
H	2.01119	-4.02792	2.37947
P	0.18788	1.16234	-0.90754
C	0.11213	2.67053	0.14340
C	0.61274	1.81292	-2.66812
C	-1.72181	1.14795	-0.95120
C	1.10489	3.36542	0.86424
C	-1.20814	3.15525	0.16040
C	-0.15444	3.09461	-3.03900
C	2.12415	2.09925	-2.71923
C	0.28014	0.69453	-3.67442
O	-2.16175	2.45416	-0.51579
H	-2.04799	1.06172	-1.98892
C	0.74160	4.53914	1.54249
C	2.53612	2.93468	0.95246
C	-1.56298	4.32907	0.82482
H	0.08978	3.91793	-2.36090
H	0.12884	3.40307	-4.05448
H	-1.24032	2.95607	-3.02801
H	2.38893	2.94321	-2.07948
H	2.72101	1.23139	-2.42109
H	2.40485	2.36064	-3.74922
H	0.77437	-0.24790	-3.41425
H	-0.79446	0.50306	-3.74925
H	0.62708	0.99238	-4.67270

C	-0.56881	5.01738	1.51514
H	1.50452	5.07953	2.09400
C	2.94284	1.95251	1.87646
C	3.53273	3.60644	0.21720
H	-2.58982	4.67580	0.79739
H	-0.81996	5.93179	2.04615
C	4.29400	1.60157	2.00951
O	1.94901	1.40471	2.63763
C	4.88464	3.26366	0.34120
O	3.08109	4.60106	-0.60910
C	5.24553	2.25532	1.23227
H	4.60258	0.82508	2.69770
C	2.32900	0.62865	3.76566
H	5.64560	3.76777	-0.24151
C	4.03254	5.35963	-1.33863
H	6.29155	1.97759	1.32735
H	1.40074	0.38184	4.28411
H	2.83433	-0.29717	3.46922
H	2.98206	1.20123	4.43723
H	3.45751	6.10858	-1.88634
H	4.74268	5.86328	-0.67014
H	4.58722	4.73599	-2.05179
C	-2.38745	0.02949	-0.11789
H	-1.98288	-0.91571	-0.48270
H	-2.04021	0.10122	0.91573
C	-3.91480	0.02440	-0.18574
C	-4.66956	0.79749	0.73392
C	-4.61101	-0.74736	-1.15793
C	-6.06627	0.83207	0.62764
C	-6.00830	-0.65592	-1.22522
C	-6.76133	0.13389	-0.35591
H	-6.63522	1.42523	1.33851
H	-6.52388	-1.24472	-1.98099
C	-3.99925	-1.79466	-2.10452
H	-4.83538	-2.10794	-2.74240
C	-8.27847	0.21820	-0.45652
H	-8.61657	0.89284	0.34233
C	-4.03800	1.53497	1.91858
H	-2.98504	1.71448	1.70233
C	-8.73014	0.82914	-1.79687
H	-8.28502	1.81835	-1.95039
H	-8.43408	0.19568	-2.64166
H	-9.82143	0.93611	-1.82682
C	-8.94998	-1.14802	-0.21933
H	-8.66030	-1.56803	0.75020
H	-10.04248	-1.05162	-0.23809
H	-8.66665	-1.87077	-0.99381

C	-3.55549	-3.06286	-1.34658
H	-2.71541	-2.87662	-0.67170
H	-4.38039	-3.46260	-0.74664
H	-3.24064	-3.83915	-2.05492
C	-2.91119	-1.32019	-3.08416
H	-3.19513	-0.38069	-3.57291
H	-1.93888	-1.18499	-2.60423
H	-2.77321	-2.07393	-3.86917
C	-4.66024	2.91808	2.18619
H	-4.70203	3.51749	1.27059
H	-5.67749	2.84670	2.58810
H	-4.05885	3.46054	2.92496
C	-4.10950	0.65901	3.18730
H	-3.61091	-0.30448	3.03516
H	-3.62934	1.16403	4.03532
H	-5.15184	0.45502	3.46158

Pro-*S* L8

Pd	0.86593	-0.21542	-0.20557
C	2.37548	1.16639	-0.91217
C	3.44017	1.01591	-1.81512
C	1.69084	2.45170	-0.86293
C	2.09546	3.52620	-1.73119
C	2.71805	-0.01730	0.78988
C	3.19340	-1.39789	0.75847
C	3.11717	0.79483	1.86823
C	3.99317	-1.92094	1.83550
C	3.92033	0.23584	2.90324
C	3.31363	-3.62889	-0.27982
C	4.42307	-3.27794	1.82252
C	4.09234	-4.12340	0.79211
H	3.05689	-4.28406	-1.10830
H	5.02624	-3.63065	2.65632
H	4.42877	-5.15669	0.79350
C	2.88337	-2.31999	-0.28969
H	2.29408	-1.96304	-1.12725
C	4.34201	-1.06800	2.90552
H	4.95227	-1.45397	3.71865
C	2.80405	2.26407	2.05145
H	3.31762	2.64294	2.94070
H	1.73627	2.45783	2.18146
H	3.13297	2.86154	1.19735
H	4.20963	0.88655	3.72496
C	1.41836	4.77689	-1.69402
C	0.36296	4.99341	-0.84177
C	-0.05830	3.94692	0.01016
C	0.58160	2.72625	-0.00762
C	3.16682	3.31908	-2.62868
H	3.47929	4.12849	-3.28408
C	3.80072	2.10652	-2.65937
H	4.62820	1.95839	-3.34885
H	1.75783	5.56153	-2.36681
H	-0.14525	5.95405	-0.82323
H	-0.89524	4.09517	0.68623
H	0.22840	1.93979	0.65368
C	4.31071	-0.21023	-1.98900
H	3.76102	-1.06418	-2.39567
H	5.12425	0.00979	-2.68696
H	4.76044	-0.53579	-1.04833
P	-1.26493	-0.96579	-1.14243
C	-2.78510	-1.35911	-0.17243
C	-1.94533	0.11106	-2.58580
C	-1.29456	-2.79820	-1.69768
C	-3.39606	-0.71778	0.91915
C	-3.37960	-2.51854	-0.70797
C	-3.17102	-0.47839	-3.30843
C	-2.35750	1.46079	-1.96400
C	-0.79522	0.35707	-3.58327
O	-2.70556	-3.16819	-1.70225
C	-0.60539	-3.64955	-0.61898
C	-4.63069	-1.21095	1.37653
C	-2.77530	0.41552	1.67107
C	-4.60336	-3.00465	-0.25532
H	-4.01015	-0.62677	-2.62228
H	-3.49714	0.23081	-4.08133
H	-2.96722	-1.43271	-3.79908
H	-3.19054	1.34977	-1.26331
H	-1.52635	1.94623	-1.44595
H	-2.68574	2.13346	-2.76838
H	0.07918	0.79012	-3.08709
H	-0.47788	-0.55708	-4.09436
H	-1.13187	1.06380	-4.35344
C	-5.23117	-2.32298	0.78683
H	-5.11115	-0.71863	2.21594
C	-1.65535	0.19320	2.50036
C	-3.37141	1.69175	1.68430
H	-5.02930	-3.89813	-0.70016
H	-6.18525	-2.68181	1.16393
C	-1.13739	1.22126	3.30277
O	-1.14559	-1.07055	2.48206
C	-2.86015	2.72521	2.48183
O	-4.46359	1.83746	0.87469
C	-1.74794	2.47297	3.28309
H	-0.27799	1.04988	3.93859

C	-0.08218	-1.39073	3.37355
H	-3.32108	3.70530	2.49025
C	-5.11463	3.09684	0.83083
H	-1.35014	3.26855	3.90735
H	0.12796	-2.44886	3.21123
H	0.81847	-0.80759	3.15205
H	-0.38177	-1.23330	4.41779
H	-5.94536	2.97680	0.13311
H	-5.50647	3.38335	1.81558
H	-4.44466	3.88554	0.46465
H	-0.78455	-4.70828	-0.84113
H	0.47153	-3.47072	-0.59871
H	-1.00266	-3.43422	0.37748
C	-0.73144	-3.13268	-3.07365
H	0.32469	-2.85114	-3.13130
H	-0.80625	-4.21382	-3.24041
H	-1.27053	-2.63134	-3.87860

Pro-*R* L8

Pd	-0.82476	0.00755	-0.02200
C	-2.11196	1.67099	-0.36005
C	-2.92889	2.03989	-1.44712
C	-1.36807	2.72853	0.32167
C	-1.43355	4.08972	-0.14277
C	-2.89337	-0.02472	0.58406
C	-3.44712	-1.14727	-0.16062
C	-3.51189	0.33452	1.79331
C	-4.58561	-1.86985	0.34188
C	-4.63190	-0.41526	2.25618
C	-3.43978	-2.68638	-2.08175
C	-5.11832	-2.97044	-0.38622
C	-4.56658	-3.37574	-1.57726
H	-2.99042	-2.99906	-3.02091
H	-5.97995	-3.48854	0.02918
H	-4.98527	-4.21575	-2.12495
C	-2.90322	-1.62072	-1.39154
H	-2.03100	-1.11030	-1.79189
C	-5.16138	-1.47531	1.57020
H	-6.02376	-2.01434	1.95479
C	-3.13039	1.49313	2.68923
H	-3.85039	1.58020	3.50870
H	-2.14179	1.37122	3.14149
H	-3.12203	2.44453	2.15231
H	-5.08647	-0.11305	3.19648
C	-0.69031	5.11292	0.51015
C	0.11304	4.83576	1.58923
C	0.19679	3.50529	2.05920
C	-0.51680	2.49980	1.44626
C	-2.24455	4.39436	-1.25750
H	-2.30193	5.42076	-1.61237
C	-2.95955	3.39991	-1.87034
H	-3.59377	3.65054	-2.71711
H	-0.77770	6.12878	0.13045
H	0.67263	5.62725	2.08087
H	0.82406	3.27097	2.91519
H	-0.44509	1.49181	1.83620
C	-3.85730	1.13691	-2.23161
H	-3.33058	0.36342	-2.79606
H	-4.42896	1.73187	-2.95047
H	-4.57187	0.62266	-1.58389
P	0.97140	-1.59371	0.48309
C	2.60511	-1.68080	-0.37223
C	1.47833	-1.54779	2.34179
C	0.74238	-3.42817	-0.02135
C	3.46632	-0.68077	-0.85815
C	2.98403	-3.03249	-0.49263
C	2.48967	-2.62868	2.76804
C	2.13338	-0.17139	2.58141
C	0.19581	-1.64836	3.19223
O	2.09295	-3.97555	-0.06687
C	4.70703	-1.07188	-1.39156
C	3.12320	0.77388	-0.87372
C	4.21502	-3.41679	-1.01778
H	3.41807	-2.55912	2.19366
H	2.74284	-2.47694	3.82632
H	2.10560	-3.64536	2.66207
H	3.09300	-0.08392	2.06442
H	1.49699	0.65646	2.25625
H	2.32030	-0.04971	3.65734
H	-0.53931	-0.88977	2.90323
H	-0.28827	-2.62581	3.10817
H	0.44848	-1.49568	4.25014
C	5.07925	-2.41416	-1.45655
H	5.37852	-0.30581	-1.76563
C	2.14384	1.27764	-1.75437
C	3.85282	1.69483	-0.09526
H	4.46796	-4.46965	-1.08937
H	6.04467	-2.68458	-1.87625
C	1.88299	2.65248	-1.83391
O	1.50045	0.34735	-2.52063
C	3.59927	3.06975	-0.16785
O	4.80273	1.14752	0.72575
C	2.61274	3.52965	-1.03723
H	1.11609	3.03687	-2.49407

C	0.58605	0.80175	-3.50989
H	4.15210	3.77355	0.44196
C	5.58595	2.01496	1.52824
H	2.40039	4.59343	-1.08723
H	0.24737	-0.09537	-4.03154
H	-0.27612	1.30781	-3.05971
H	1.07649	1.47423	-4.22574
H	6.27500	1.37026	2.07720
H	6.16064	2.72252	0.91626
H	4.96790	2.57570	2.24174
C	-0.08781	-4.31412	0.89939
H	-0.14640	-5.32199	0.47164
H	0.34286	-4.39944	1.89806
H	-1.10538	-3.92093	0.98463
C	0.18282	-3.48579	-1.45041
H	-0.86986	-3.20182	-1.46311
H	0.73255	-2.82720	-2.13006
H	0.27581	-4.51541	-1.81574

Pro-*S* L9

Pd	-1.23187	0.59565	0.42069
C	-1.88715	2.60230	0.04800
C	-2.83385	3.39410	0.71724
C	-0.70723	3.25587	-0.50088
C	-0.52747	4.67628	-0.35717
C	-2.82691	1.02848	-0.94575
C	-3.86754	0.16948	-0.38319
C	-2.87867	1.29801	-2.32727
C	-4.86409	-0.43162	-1.23177
C	-3.89882	0.69911	-3.12088
C	-4.89753	-1.03978	1.50317
C	-5.83877	-1.31574	-0.68949
C	-5.85865	-1.62704	0.64849
H	-4.91154	-1.26332	2.56675
H	-6.57283	-1.74380	-1.36873
H	-6.60730	-2.30524	1.04923
C	-3.94865	-0.17503	1.00233
H	-3.23421	0.27389	1.68220
C	-4.85357	-0.13972	-2.61239
H	-5.60751	-0.58288	-3.25852
C	-1.96352	2.21584	-3.10934
H	-2.31646	2.29350	-4.14233
H	-0.93109	1.85914	-3.14309
H	-1.93657	3.22511	-2.69097
H	-3.90882	0.92654	-4.18391
C	0.63033	5.31036	-0.88888
C	1.60305	4.59273	-1.54222
C	1.45154	3.19322	-1.67954
C	0.34088	2.55527	-1.16992
C	-1.51065	5.42768	0.32586
H	-1.38345	6.50214	0.43347
C	-2.61161	4.79544	0.84072
H	-3.36410	5.38178	1.36265
H	0.72787	6.38636	-0.76147
H	2.47967	5.09152	-1.94741
H	2.21149	2.60959	-2.19229
H	0.25194	1.47813	-1.28426
C	-4.12856	2.90493	1.32817
H	-3.96411	2.22813	2.17249
H	-4.70790	3.75408	1.70372
H	-4.75054	2.37157	0.60521
P	0.36156	-0.13912	2.10918
C	1.38237	-1.65270	1.88790
C	1.58966	1.16648	2.80074
C	-0.30365	-0.91312	3.70905
C	2.08547	-2.14597	0.77368
C	1.41138	-2.37660	3.09290
C	2.43313	0.66325	3.98682
C	2.53936	1.57211	1.66097
C	0.74517	2.38031	3.23568
O	0.65804	-1.91341	4.12987
H	-0.32226	-0.16033	4.50183
C	-1.67178	-1.57302	3.58489
C	2.86402	-3.30347	0.93199
C	1.98064	-1.55077	-0.59416
C	2.18277	-3.52716	3.25019
H	3.04470	-0.20204	3.71351
H	3.11557	1.46530	4.29844
H	1.82864	0.39111	4.85706
H	3.16318	0.73031	1.34360
H	1.99620	1.95471	0.79304
H	3.20901	2.36781	2.01476
H	0.15345	2.78111	2.40729
H	0.05931	2.13332	4.05595
H	1.40991	3.17600	3.59685
C	2.92061	-3.97331	2.15559
H	3.41526	-3.68698	0.07924
C	0.80396	-1.70482	-1.34589
C	3.07756	-0.93204	-1.22865
H	2.18459	-4.05235	4.19966
H	3.52742	-4.86996	2.24897
C	0.70071	-1.25109	-2.66488
O	-0.27823	-2.27663	-0.70743
C	2.99977	-0.48538	-2.55030

O	4.22328	-0.83564	-0.46878
C	1.80648	-0.64887	-3.25679
H	-0.23097	-1.37935	-3.20456
H	3.86024	-0.02537	-3.02109
H	1.74348	-0.29909	-4.28343
H	-1.88940	-2.13043	4.50267
H	-2.44824	-0.81695	3.44314
H	-1.71001	-2.26610	2.73826
C	5.33165	-0.14645	-0.94366
C	5.33053	1.24877	-1.01418
C	6.47473	-0.88052	-1.25847
C	6.49342	1.90958	-1.41303
H	4.43272	1.80031	-0.75391
C	7.63400	-0.20793	-1.65034
H	6.44510	-1.96289	-1.18277
C	7.64580	1.18600	-1.73146
H	6.49926	2.99482	-1.46681
H	8.52774	-0.77659	-1.89199
H	8.54891	1.70672	-2.03670
C	-0.99055	-3.30007	-1.31320
C	-0.38694	-4.24835	-2.14379
C	-2.34587	-3.39087	-0.98971
C	-1.16023	-5.28989	-2.66004
H	0.66965	-4.17443	-2.37827
C	-3.10280	-4.44074	-1.50883
H	-2.78926	-2.64052	-0.34408
C	-2.51714	-5.39253	-2.34739
H	-0.69178	-6.02825	-3.30560
H	-4.15827	-4.50369	-1.25905
H	-3.11170	-6.20623	-2.75286

Pro-*R* L9

Pd	-1.46111	-0.53425	-0.31557
C	-2.44904	-1.69641	1.16760
C	-2.90873	-1.40989	2.46598
C	-1.79295	-2.98131	0.94053
C	-1.53857	-3.87682	2.03944
C	-3.60241	-0.62185	-0.24555
C	-3.98761	0.76569	-0.03842
C	-4.50890	-1.48141	-0.88190
C	-5.26191	1.24105	-0.50851
C	-5.75952	-0.96674	-1.33069
C	-3.53993	3.04115	0.77627
C	-5.63328	2.60220	-0.32197
C	-4.79763	3.49033	0.31057
H	-2.87606	3.73216	1.28862
H	-6.60381	2.92341	-0.69405
H	-5.09618	4.52558	0.45316
C	-3.14981	1.73157	0.59742
H	-2.17222	1.41715	0.95367
C	-6.13460	0.33947	-1.15969
H	-7.10127	0.69214	-1.51122
C	-4.30617	-2.95890	-1.13755
H	-5.23243	-3.40194	-1.51654
H	-3.52882	-3.15220	-1.88406
H	-4.02421	-3.49967	-0.23164
H	-6.44225	-1.65548	-1.82267
C	-0.85471	-5.10653	1.82596
C	-0.42201	-5.47785	0.57643
C	-0.67434	-4.62178	-0.51995
C	-1.33369	-3.42557	-0.33881
C	-1.98219	-3.51744	3.33069
H	-1.80218	-4.19250	4.16422
C	-2.65183	-2.33710	3.51667
H	-3.01152	-2.08444	4.51117
H	-0.68559	-5.75233	2.68507
H	0.09728	-6.42080	0.42730
H	-0.35366	-4.91026	-1.51753
H	-1.52555	-2.79598	-1.19906
C	-3.70779	-0.19925	2.89872
H	-3.13471	0.73031	2.84976
H	-4.03557	-0.32621	3.93516
H	-4.59876	-0.05465	2.28297
P	-0.01170	0.47982	-1.97883
C	1.40187	1.56360	-1.51692
C	0.71511	-0.76462	-3.25078
C	-0.61967	1.86932	-3.11890
C	2.35035	1.46825	-0.48010
C	1.47407	2.64475	-2.41272
C	1.65823	-0.11504	-4.27941
C	1.48862	-1.84614	-2.47527
C	-0.48155	-1.41162	-3.97757
O	0.51094	2.74489	-3.36963
H	-0.89117	1.44682	-4.08992
C	3.38154	2.41601	-0.41969
C	2.25867	0.42322	0.58492
C	2.49898	3.58960	-2.35235
H	2.52550	0.34555	-3.79641
H	2.03084	-0.88909	-4.96382
H	1.16240	0.64979	-4.88492
H	2.38428	-1.43770	-1.99989
H	0.87456	-2.32179	-1.70405
H	1.81364	-2.62947	-3.17420
H	-1.18340	-1.87277	-3.27508

H	-1.04189	-0.69177	-4.58549
H	-0.11589	-2.19385	-4.65564
C	3.45835	3.45379	-1.35195
H	4.11613	2.34824	0.37636
C	1.30349	0.52784	1.61279
C	3.12827	-0.67899	0.62971
H	2.52454	4.40506	-3.06774
H	4.26459	4.17877	-1.28160
C	1.16432	-0.44928	2.59640
O	0.45438	1.62474	1.62895
C	3.00820	-1.66873	1.61006
O	4.04560	-0.78649	-0.39855
C	2.01768	-1.55309	2.58258
H	0.39007	-0.33918	3.34765
H	3.67734	-2.52214	1.59487
H	1.90728	-2.32978	3.33311
C	-1.77498	2.69351	-2.56860
H	-1.99636	3.51624	-3.25758
H	-2.66953	2.07518	-2.45307
H	-1.53285	3.11533	-1.58926
C	5.33839	-1.21292	-0.15525
C	6.04694	-0.85913	0.99703
C	5.94894	-1.95911	-1.16579
C	7.37424	-1.26931	1.13277
H	5.56661	-0.27242	1.77297
C	7.27809	-2.35511	-1.01971
H	5.37516	-2.21325	-2.05127
C	7.99574	-2.01648	0.13001
H	7.92534	-0.99430	2.02814
H	7.75132	-2.93490	-1.80753
H	9.02941	-2.33015	0.24268
C	0.88684	2.79553	2.22903
C	1.91321	2.84356	3.17590
C	0.20615	3.95997	1.86099
C	2.24956	4.07036	3.75451
H	2.43638	1.93810	3.46266
C	0.54958	5.17432	2.45089
H	-0.57274	3.89558	1.10848
C	1.57326	5.23793	3.40082
H	3.04714	4.10521	4.49221
H	0.01871	6.07680	2.15964
H	1.84048	6.18661	3.85712

Pro-*S* L10

Pd	-1.09148	0.07190	0.42325
C	-2.41711	1.76960	0.44915
C	-3.51487	2.07292	1.27045
C	-1.57322	2.86629	-0.00592
C	-1.86796	4.22086	0.38101
C	-2.85947	0.12834	-0.76638
C	-3.52039	-1.09384	-0.31317
C	-3.10892	0.55779	-2.08458
C	-4.35193	-1.85634	-1.20835
C	-3.95077	-0.22269	-2.92812
C	-3.95045	-2.82148	1.39138
C	-4.95582	-3.07060	-0.77621
C	-4.76053	-3.55482	0.49418
H	-3.79664	-3.18721	2.40330
H	-5.57927	-3.61125	-1.48512
H	-5.22592	-4.48448	0.81054
C	-3.36103	-1.63982	0.99844
H	-2.75523	-1.08922	1.70861
C	-4.55049	-1.38620	-2.52465
H	-5.18392	-1.95135	-3.20437
C	-2.59613	1.82380	-2.73624
H	-3.02597	1.92259	-3.73797
H	-1.50872	1.83565	-2.84325
H	-2.86894	2.71691	-2.16835
H	-4.12205	0.13593	-3.94017
C	-1.03849	5.29066	-0.05730
C	0.06253	5.06448	-0.84736
C	0.37812	3.73886	-1.22423
C	-0.40931	2.68549	-0.81198
C	-2.98432	4.47178	1.21028
H	-3.21458	5.49360	1.50215
C	-3.76283	3.42808	1.63393
H	-4.62119	3.63087	2.26980
H	-1.29786	6.29965	0.25612
H	0.68702	5.89128	-1.17575
H	1.25019	3.53829	-1.84018
H	-0.13580	1.67714	-1.11117
C	-4.52451	1.08734	1.81758
H	-4.08139	0.37968	2.52470
H	-5.31498	1.62363	2.35157
H	-4.99792	0.49883	1.02811
P	0.86625	-0.41598	1.79764
C	2.24410	-1.47182	1.18813
C	1.72088	1.06377	2.67487
C	0.66601	-1.63469	3.23996
C	2.91033	-1.50858	-0.05111
C	2.62506	-2.37140	2.19989
C	2.85052	0.64170	3.63220
C	2.30723	1.98913	1.59541
C	0.62004	1.81178	3.45281

O	1.91499	-2.35865	3.36416
C	3.98667	-2.39659	-0.20371
C	2.49153	-0.69188	-1.23216
C	3.69429	-3.25252	2.04768
H	3.64720	0.10625	3.10649
H	3.29317	1.54356	4.07611
H	2.50189	0.00873	4.45366
H	3.09596	1.49125	1.02331
H	1.54246	2.34286	0.89997
H	2.74909	2.87088	2.08035
H	-0.18686	2.14330	2.79167
H	0.17950	1.19614	4.24708
H	1.05308	2.69926	3.93276
C	4.37851	-3.24455	0.83356
H	4.50902	-2.42641	-1.15480
C	1.35091	-1.06688	-1.97374
C	3.27723	0.38367	-1.69288
H	3.96068	-3.92527	2.85633
H	5.21321	-3.92483	0.68555
C	0.97511	-0.34845	-3.11943
O	0.67373	-2.14912	-1.49571
C	2.90972	1.09902	-2.84240
O	4.37955	0.66509	-0.93399
C	1.75925	0.72451	-3.53515
H	0.09112	-0.62214	-3.68148
C	-0.28652	-2.85211	-2.31738
H	3.50797	1.92599	-3.20394
C	5.37070	1.60856	-1.38399
H	1.47298	1.27775	-4.42574
H	-1.02695	-2.13332	-2.69014
H	4.86463	2.51364	-1.74527
C	-0.46762	-2.64099	3.08364
H	-1.43606	-2.13999	3.15970
H	-0.42190	-3.15257	2.11692
H	-0.39780	-3.39136	3.87907
C	6.18894	1.96278	-0.14670
H	6.66136	1.06503	0.26617
H	6.97366	2.68211	-0.40474
H	5.55218	2.40356	0.62599
C	6.22839	1.00298	-2.49641
H	5.62221	0.70717	-3.35763
H	6.97616	1.72810	-2.83718
H	6.75219	0.11579	-2.12374
C	-0.97774	-3.83309	-1.37812
H	-1.74935	-4.39114	-1.91867
H	-0.25073	-4.54589	-0.97278
H	-1.45507	-3.30559	-0.54857
C	0.40813	-3.56391	-3.47978
H	-0.33157	-4.09920	-4.08582
H	0.93977	-2.86466	-4.13138
H	1.13074	-4.29186	-3.09442
H	0.54448	-1.07978	4.17420

Pro-*R* L10

Pd	1.10384	-0.04910	-0.20293
C	2.22810	1.72217	0.15268
C	3.00272	2.14644	1.25016
C	1.39093	2.71666	-0.51494
C	1.33517	4.07254	-0.03407
C	3.16966	0.13115	-0.82415
C	3.84409	-0.93567	-0.09646
C	3.74470	0.57225	-2.02789
C	5.06515	-1.50869	-0.59834
C	4.94651	-0.03544	-2.49448
C	3.99763	-2.50627	1.79301
C	5.72001	-2.54887	0.11837
C	5.20791	-3.04069	1.29475
H	3.57224	-2.89556	2.71447
H	6.64227	-2.95155	-0.29476
H	5.71931	-3.83425	1.83300
C	3.34573	-1.49972	1.11442
H	2.40706	-1.11394	1.50350
C	5.59737	-1.03081	-1.81653
H	6.52064	-1.45705	-2.20138
C	3.23447	1.68768	-2.91459
H	3.93294	1.85306	-3.74057
H	2.25974	1.46388	-3.35753
H	3.13161	2.63131	-2.37365
H	5.36467	0.32861	-3.42972
C	0.50243	5.03294	-0.67441
C	-0.27662	4.69739	-1.75477
C	-0.24375	3.36975	-2.23912
C	0.55911	2.42546	-1.63950
C	2.11535	4.43426	1.08549
H	2.08202	5.45751	1.45240
C	2.91206	3.49837	1.68992
H	3.51840	3.79281	2.54303
H	0.50017	6.04826	-0.28332
H	-0.90589	5.44132	-2.23636
H	-0.85106	3.09005	-3.09592
H	0.57674	1.41899	-2.03973
C	3.99971	1.31721	2.03099
H	3.54101	0.47868	2.56022
H	4.49428	1.94412	2.77935

H	4.77545	0.89607	1.38608
P	-0.57496	-1.77285	-0.69820
C	-2.08821	-2.05344	0.31398
C	-1.19431	-1.73336	-2.51637
C	-0.17059	-3.61428	-0.47867
C	-2.98303	-1.15581	0.92706
C	-2.30742	-3.43757	0.44528
C	-2.15808	-2.88173	-2.86726
C	-1.92119	-0.39663	-2.74775
C	0.05800	-1.80806	-3.41262
O	-1.40762	-4.28348	-0.13164
H	0.14412	-4.03522	-1.43748
C	-4.08962	-1.68066	1.61328
C	-2.80902	0.32924	0.89833
C	-3.40869	-3.95620	1.12428
H	-3.05553	-2.86045	-2.24141
H	-2.47815	-2.77221	-3.91239
H	-1.69800	-3.86911	-2.76637
H	-2.85088	-0.34282	-2.17477
H	-1.30514	0.46385	-2.47245
H	-2.17509	-0.30245	-3.81289
H	0.76576	-1.00291	-3.18853
H	0.59206	-2.75959	-3.30188
H	-0.23949	-1.72200	-4.46613
C	-4.30201	-3.05703	1.70299
H	-4.78504	-0.99305	2.08433
C	-1.86542	0.95565	1.73817
C	-3.65563	1.13702	0.11156
H	-3.54083	-5.03071	1.19794
H	-5.16605	-3.43405	2.24409
C	-1.72861	2.35104	1.74365
O	-1.12420	0.11288	2.51807
C	-3.52942	2.53200	0.12216
O	-4.56281	0.45458	-0.65571
C	-2.55877	3.11783	0.93191
H	-0.98314	2.83627	2.36063
C	-0.38986	0.61698	3.65260
H	-4.16693	3.15789	-0.48973
C	-5.63155	1.14506	-1.32938
H	-2.44325	4.19761	0.92604
H	0.26022	1.43661	3.32068
H	-5.21936	2.01303	-1.86114
C	0.86649	-3.92260	0.59102
H	0.94029	-5.00819	0.72125
H	1.84525	-3.53150	0.30712
H	0.58623	-3.47839	1.55239
C	-6.17541	0.15375	-2.35304
H	-6.99990	0.60399	-2.91637
H	-5.39343	-0.14394	-3.05810
H	-6.54682	-0.74586	-1.85068
C	-6.69917	1.59751	-0.33178
H	-6.28329	2.26890	0.42508
H	-7.50636	2.12579	-0.85190
H	-7.12758	0.72735	0.17786
C	0.47989	-0.54470	4.11929
H	1.13508	-0.88147	3.31150
H	1.10011	-0.23668	4.96803
H	-0.14562	-1.38801	4.43199
C	-1.33884	1.09718	4.75256
H	-0.76466	1.46892	5.60889
H	-1.99107	1.90284	4.40438
H	-1.96831	0.26709	5.09216

Pro-*S* L11

Pd	1.04508	0.12101	-0.36837
C	2.38580	1.77998	-0.59304
C	3.51809	1.93074	-1.41124
C	1.54371	2.94627	-0.36409
C	1.87457	4.21421	-0.95917
C	2.74823	0.39614	0.92404
C	3.44395	-0.87842	0.74630
C	2.92544	1.06285	2.15468
C	4.23612	-1.44610	1.80764
C	3.71354	0.45726	3.17635
C	4.01136	-2.87076	-0.59093
C	4.89237	-2.69693	1.63467
C	4.78860	-3.40403	0.46191
H	3.92128	-3.41472	-1.52705
H	5.48302	-3.08153	2.46338
H	5.29623	-4.35735	0.34167
C	3.36781	-1.66106	-0.44533
H	2.78365	-1.27443	-1.26983
C	4.34665	-0.74717	3.02836
H	4.94302	-1.16742	3.83480
C	2.39791	2.42784	2.54312
H	2.79613	2.70880	3.52304
H	1.30818	2.45932	2.61239
H	2.69510	3.20204	1.83196
H	3.82061	0.99613	4.11445
C	1.05172	5.35215	-0.72963
C	-0.07478	5.27390	0.05327
C	-0.42577	4.03252	0.63139
C	0.35195	2.91472	0.41915
C	3.02226	4.31032	-1.77762

H	3.27945	5.26582	-2.22836
C	3.79847	3.20205	-1.98956
H	4.68157	3.28604	-2.61833
H	1.33841	6.29246	-1.19565
H	-0.69240	6.15200	0.22323
H	-1.31893	3.94813	1.24432
H	0.05456	1.97164	0.86942
C	4.53355	0.85898	-1.74223
H	4.10468	0.03617	-2.32159
H	5.34289	1.28721	-2.34158
H	4.98045	0.42269	-0.84560
P	-0.93276	-0.56427	-1.71107
C	-2.27193	-1.53628	-0.90510
C	-1.85099	0.82864	-2.67994
C	-0.86971	-1.88506	-3.09638
C	-2.87177	-1.42605	0.36533
C	-2.70468	-2.55002	-1.77796
C	-3.04881	0.32193	-3.50585
C	-2.37194	1.86385	-1.67179
C	-0.80573	1.48876	-3.60074
O	-2.07711	-2.67538	-2.97952
C	-3.91899	-2.30310	0.68858
C	-2.43992	-0.44309	1.40824
C	-3.74709	-3.41865	-1.45645
H	-3.80229	-0.15444	-2.87088
H	-3.52427	1.18168	-3.99669
H	-2.77073	-0.38590	-4.29193
H	-3.12964	1.43406	-1.01106
H	-1.56993	2.28210	-1.06208
H	-2.83699	2.69492	-2.22065
H	0.03849	1.88772	-3.02904
H	-0.41034	0.79510	-4.35297
H	-1.27261	2.32215	-4.14198
C	-4.35411	-3.27740	-0.21092
H	-4.38857	-2.21746	1.66351
C	-1.29107	-0.69293	2.18687
C	-3.23914	0.67639	1.72132
H	-4.05218	-4.18152	-2.16526
H	-5.16547	-3.94447	0.06856
C	-0.91244	0.19256	3.20838
O	-0.60266	-1.82969	1.87693
C	-2.86797	1.55984	2.74498
O	-4.36197	0.82229	0.95382
C	-1.70367	1.30725	3.46974
H	-0.01736	0.01449	3.79101
C	0.27152	-2.44628	2.85291
H	-3.47503	2.42270	2.98891
C	-5.37125	1.79404	1.28924
H	-1.41394	1.99092	4.26335
H	1.02183	-1.71204	3.17121
H	-4.88545	2.75572	1.50158
C	0.32562	-2.84621	-3.21148
H	0.00833	-3.57076	-3.97634
C	-6.22813	1.94159	0.03627
H	-6.68323	0.98105	-0.22799
H	-7.02761	2.67038	0.20801
H	-5.62301	2.28067	-0.80967
C	-6.18451	1.33393	2.50030
H	-5.55035	1.18352	3.37880
H	-6.94649	2.08035	2.75197
H	-6.68885	0.38795	2.27422
C	0.97321	-3.57890	2.11494
H	1.66789	-4.09015	2.78978
H	0.24000	-4.30720	1.75109
H	1.54190	-3.19675	1.26446
C	-0.52701	-2.95428	4.05486
H	0.14715	-3.42853	4.77703
H	-1.05835	-2.14647	4.56608
H	-1.26221	-3.69831	3.72835
H	-0.95081	-1.35151	-4.04784
C	1.55733	-2.11221	-3.76164
H	1.84776	-1.27316	-3.12098
H	2.41160	-2.79413	-3.83984
H	1.36182	-1.70944	-4.76338
C	0.60460	-3.63483	-1.92717
H	-0.28613	-4.18433	-1.60594
H	1.40537	-4.36479	-2.09295
H	0.91567	-2.98099	-1.10737

Pro-*R* L11

Pd	0.98221	0.29677	-0.41715
C	1.96555	2.03416	0.27924
C	2.39969	2.41913	1.56046
C	1.35251	3.04899	-0.57189
C	1.15113	4.38801	-0.08235
C	3.14502	0.44293	-0.40503
C	3.54605	-0.67492	0.43864
C	4.06281	0.92126	-1.35362
C	4.85264	-1.26141	0.29621
C	5.34549	0.30908	-1.45788
C	3.07780	-2.37418	2.15554
C	5.23457	-2.37448	1.09656
C	4.37040	-2.92865	2.00936
H	2.38190	-2.80840	2.86801

H	6.23237	-2.78529	0.95780
H	4.67160	-3.78263	2.61036
C	2.68698	-1.28855	1.40070
H	1.68499	-0.88702	1.53354
C	5.74267	-0.73507	-0.66679
H	6.73374	-1.16973	-0.77282
C	3.85123	2.08275	-2.30069
H	4.76406	2.26316	-2.87666
H	3.04732	1.89959	-3.01914
H	3.60396	3.00685	-1.77232
H	6.03564	0.70586	-2.19843
C	0.51533	5.36604	-0.89787
C	0.07369	5.06580	-2.16319
C	0.26537	3.75819	-2.66605
C	0.88116	2.79474	-1.89772
C	1.59258	4.71511	1.21829
H	1.45512	5.72806	1.58958
C	2.19645	3.75991	1.99347
H	2.54469	4.02802	2.98820
H	0.38711	6.36631	-0.48947
H	-0.40965	5.82269	-2.77530
H	-0.06817	3.50978	-3.67023
H	1.02558	1.80314	-2.31202
C	3.11413	1.54295	2.56540
H	2.51160	0.68945	2.88437
H	3.35921	2.12635	3.45851
H	4.04793	1.13772	2.16575
P	-0.59436	-1.38415	-1.22645
C	-1.92354	-1.96041	-0.08909
C	-1.46603	-0.92344	-2.87669
C	-0.14675	-3.21120	-1.57079
C	-2.74403	-1.26559	0.82319
C	-2.05674	-3.35590	-0.19548
C	-2.57128	-1.91942	-3.27050
C	-2.08338	0.47795	-2.73198
C	-0.37356	-0.87945	-3.96479
O	-1.22545	-4.01821	-1.04234
H	-0.16537	-3.37492	-2.65337
C	-3.70841	-1.98748	1.54261
C	-2.62560	0.20358	1.08404
C	-3.01123	-4.07371	0.52558
H	-3.36652	-1.94818	-2.51908
H	-3.02056	-1.60482	-4.22234
H	-2.19728	-2.93930	-3.40670
H	-2.91169	0.47457	-2.02094
H	-1.34996	1.22195	-2.40685
H	-2.47870	0.80043	-3.70577
H	0.43039	-0.18343	-3.70091
H	0.07975	-1.85914	-4.15202
H	-0.81747	-0.54057	-4.91006
C	-3.84350	-3.36898	1.39082
H	-4.35343	-1.45235	2.23258
C	-1.67857	0.69149	2.00283
C	-3.52930	1.11968	0.50191
H	-3.08145	-5.14973	0.40423
H	-4.59670	-3.90304	1.96432
C	-1.56257	2.06431	2.25805
O	-0.87582	-0.23061	2.63486
C	-3.42798	2.49039	0.76630
O	-4.47184	0.56305	-0.32202
C	-2.43215	2.94838	1.63001
H	-0.78978	2.43239	2.92306
C	-0.78846	-0.15815	4.07912
H	-4.10566	3.19953	0.30711
C	-5.60971	1.32720	-0.76750
H	-2.33856	4.01468	1.81522
H	-0.40907	0.83238	4.36343
H	-5.26197	2.29202	-1.15999
C	1.21269	-3.69766	-1.03493
H	1.34222	-3.32386	-0.01213
C	-6.21926	0.52172	-1.91009
H	-7.09707	1.03924	-2.31183
H	-5.49521	0.38523	-2.71881
H	-6.53003	-0.46661	-1.55475
C	-6.59693	1.54905	0.37952
H	-6.13370	2.08662	1.21190
H	-7.45721	2.13237	0.03213
H	-6.96041	0.58485	0.75181
C	0.22592	-1.21552	4.49333
H	-0.11453	-2.20970	4.18314
H	1.20079	-1.02399	4.03702
H	0.34791	-1.21400	5.58201
C	-2.15594	-0.38318	4.72492
H	-2.06769	-0.34081	5.81654
H	-2.87400	0.38184	4.41354
H	-2.55041	-1.36538	4.44306
C	1.28046	-5.23348	-1.00167
H	2.26168	-5.55344	-0.63288
H	0.51137	-5.65998	-0.35311
H	1.14657	-5.65688	-2.00606
C	2.34502	-3.13379	-1.90620
H	2.28157	-3.53396	-2.92772
H	2.31925	-2.04135	-1.95981
H	3.31798	-3.41856	-1.49373

Pro-*S* L12

Pd	-1.15008	0.02795	0.32058
C	-2.38754	1.78137	0.62791
C	-3.50492	2.01289	1.44732
C	-1.45925	2.88303	0.40575
C	-1.69152	4.16670	1.01462
C	-2.87221	0.38634	-0.85439
C	-3.63558	-0.83565	-0.61404
C	-3.03643	1.03280	-2.09439
C	-4.49191	-1.37811	-1.63670
C	-3.90408	0.46408	-3.06994
C	-4.26282	-2.76166	0.78964
C	-5.20507	-2.58979	-1.41381
C	-5.09640	-3.27710	-0.22986
H	-4.17772	-3.28905	1.73632
H	-5.84238	-2.96155	-2.21327
H	-5.64646	-4.20105	-0.07283
C	-3.56384	-1.58957	0.59913
H	-2.93867	-1.20989	1.39993
C	-4.60709	-0.69421	-2.86656
H	-5.25874	-1.09313	-3.64039
C	-2.39608	2.33583	-2.52102
H	-2.76028	2.61860	-3.51375
H	-1.30637	2.27519	-2.57588
H	-2.63406	3.15268	-1.83462
H	-4.00936	0.98672	-4.01761
C	-0.77995	5.23908	0.80701
C	0.34553	5.08137	0.03543
C	0.60131	3.82388	-0.55731
C	-0.26805	2.77046	-0.37245
C	-2.82961	4.34471	1.83237
H	-3.01213	5.31333	2.29151
C	-3.68808	3.29842	2.03524
H	-4.56294	3.44515	2.66385
H	-0.99615	6.19251	1.28432
H	1.03417	5.90885	-0.11471
H	1.49114	3.67461	-1.16151
H	-0.03728	1.81414	-0.83405
C	-4.60629	1.02715	1.77391
H	-4.25082	0.17548	2.36091
H	-5.38295	1.52284	2.36424
H	-5.08040	0.62320	0.87637
P	0.71813	-0.89255	1.60528
C	2.17985	-1.75838	0.87920
C	1.50835	0.31371	2.88086
C	0.34375	-2.54065	2.51062
C	2.96791	-1.48832	-0.25388
C	2.48825	-2.87814	1.67759
C	2.55746	-0.32778	3.80808
C	2.20425	1.41959	2.06292
C	0.37255	0.95410	3.70478
O	1.64730	-3.16178	2.71555
C	4.08465	-2.30666	-0.49880
C	2.65995	-0.41594	-1.25128
C	3.59792	-3.68357	1.43615
H	3.38774	-0.75595	3.23852
H	2.97170	0.45184	4.46179
H	2.14978	-1.11411	4.44702
H	3.03829	1.02868	1.47352
H	1.51110	1.93303	1.39179
H	2.60860	2.16861	2.75823
H	-0.37367	1.42606	3.05763
H	-0.14201	0.23447	4.34840
H	0.79582	1.73150	4.35480
C	4.40325	-3.37344	0.34070
H	4.69721	-2.10439	-1.37193
C	1.59566	-0.58111	-2.16481
C	3.50639	0.70562	-1.38650
H	3.80060	-4.53318	2.08013
H	5.27252	-3.98864	0.12312
C	1.36368	0.37223	-3.16835
O	0.84662	-1.70964	-2.00269
C	3.27449	1.65769	-2.39030
O	4.50535	0.77535	-0.45605
C	2.20696	1.47577	-3.26667
H	0.54716	0.25609	-3.86945
C	-0.05430	-2.15289	-3.04329
H	3.90862	2.52679	-2.49220
C	5.70395	1.57244	-0.58290
H	2.02850	2.21251	-4.04548
H	-0.70163	-1.31568	-3.33216
C	-0.44945	-3.45688	1.56622
H	-0.50734	-4.45608	2.01423
C	6.47485	1.29563	-1.87559
H	5.96622	1.67811	-2.76416
H	7.45955	1.77323	-1.81785
H	6.62584	0.21881	-2.00321
C	-0.91686	-3.23718	-2.40957
H	-1.64171	-3.61247	-3.13986
H	-0.29406	-4.07520	-2.07689
H	-1.46690	-2.84300	-1.55140
C	0.72890	-2.67085	-4.25094
H	0.03709	-3.00553	-5.03223

H	1.37824	-1.90107	-4.67778
H	1.35315	-3.52120	-3.95474
H	0.03554	-3.54477	0.59015
H	-1.46279	-3.08302	1.40677
C	-0.34306	-2.46773	3.86932
H	-1.32102	-1.98492	3.77734
H	-0.49928	-3.48507	4.24743
H	0.24695	-1.92344	4.60762
H	6.30095	1.17512	0.24518
C	5.46910	3.05850	-0.29113
H	6.43359	3.54730	-0.11007
H	4.97911	3.58864	-1.11238
H	4.85440	3.17293	0.60698

Pro-*R* L12

Pd	1.15000	0.07800	0.00700
C	2.32800	1.84200	0.15000
C	3.24300	2.29400	1.12100
C	1.42900	2.82700	-0.44900
C	1.46300	4.20400	-0.02900
C	3.11800	0.21200	-0.88900
C	3.85100	-0.86600	-0.23900
C	3.54000	0.61400	-2.16800
C	4.95200	-1.51300	-0.90300
C	4.63200	-0.05900	-2.79000
C	4.21800	-2.40300	1.65000
C	5.65100	-2.57800	-0.27000
C	5.30100	-3.02100	0.98300
H	3.93100	-2.74100	2.64300
H	6.47600	-3.03900	-0.80900
H	5.84500	-3.83300	1.45600
C	3.52400	-1.37300	1.05400
H	2.69200	-0.91400	1.58100
C	5.32100	-1.08200	-2.19700
H	6.15400	-1.56300	-2.70400
C	2.96900	1.74700	-2.99400
H	3.56200	1.87500	-3.90500
H	1.93600	1.57000	-3.30400
H	2.98200	2.69700	-2.45400
H	4.93000	0.27500	-3.78100
C	0.58400	5.15800	-0.61200
C	-0.32200	4.79800	-1.58000
C	-0.38300	3.44900	-1.99800
C	0.45800	2.50800	-1.44800
C	2.38500	4.59200	0.96800
H	2.41800	5.63100	1.28900
C	3.23400	3.66400	1.50900
H	3.94800	3.97900	2.26600
H	0.65100	6.19000	-0.27300
H	-0.98300	5.53900	-2.02300
H	-1.10000	3.14800	-2.75600
H	0.39700	1.48300	-1.79300
C	4.31500	1.47100	1.80300
H	3.90800	0.68100	2.44000
H	4.92700	2.11700	2.43900
H	4.98000	0.98600	1.08300
P	-0.45100	-1.76200	-0.28100
C	-2.09400	-1.94300	0.55000
C	-0.88500	-2.04400	-2.13700
C	-0.00700	-3.46200	0.48600
C	-3.10200	-1.01500	0.87200
C	-2.29400	-3.30300	0.86400
C	-1.76300	-3.27800	-2.41600
C	-1.66400	-0.79400	-2.59400
C	0.43100	-2.12700	-2.93800
O	-1.27600	-4.17000	0.58900
C	-4.29000	-1.49600	1.45200
C	-2.99400	0.46300	0.66600
C	-3.47400	-3.77600	1.43100
H	-2.71600	-3.22200	-1.88300
H	-1.98500	-3.31800	-3.49100
H	-1.28100	-4.21900	-2.14200
H	-2.63800	-0.71800	-2.10300
H	-1.11200	0.13100	-2.40100
H	-1.83800	-0.85900	-3.67700
H	1.07000	-1.25700	-2.75600
H	1.01300	-3.02200	-2.70100
H	0.19800	-2.16100	-4.01100
C	-4.47800	-2.85200	1.71400
H	-5.07000	-0.78400	1.70200
C	-2.15500	1.25200	1.48100
C	-3.84300	1.11300	-0.25500
H	-3.58100	-4.83300	1.65200
H	-5.40800	-3.19000	2.16400
C	-2.15500	2.65000	1.36500
O	-1.37400	0.56500	2.36800
C	-3.84700	2.50900	-0.37200
O	-4.62200	0.28100	-1.01500
C	-2.99900	3.25700	0.44200
H	-1.50300	3.26100	1.97500
C	-0.69800	1.25600	3.43900
H	-4.49400	3.01200	-1.07900
C	-5.65100	0.80400	-1.87500
H	-2.99000	4.33900	0.35000

H	-0.15800	2.11600	3.02300
H	-5.24500	1.65000	-2.44600
C	0.50200	-3.24700	1.92000
H	0.54000	-4.22100	2.42200
H	1.50300	-2.81800	1.91200
H	-0.15900	-2.59000	2.49200
C	-5.98900	-0.32700	-2.84100
H	-6.77000	-0.00900	-3.54000
H	-5.10500	-0.62100	-3.41500
H	-6.34900	-1.20200	-2.29000
C	-6.86300	1.25500	-1.05700
H	-6.59500	2.02900	-0.33200
H	-7.63900	1.65900	-1.71800
H	-7.28400	0.40300	-0.51200
C	0.32100	0.26600	3.98900
H	-0.18100	-0.62200	4.38800
H	1.00900	-0.04600	3.19700
H	0.90100	0.73000	4.79500
C	-1.69700	1.71300	4.50300
H	-1.17500	2.23700	5.31200
H	-2.44800	2.39100	4.08700
H	-2.21400	0.84600	4.92900
C	0.95800	-4.35500	-0.28300
H	1.92400	-3.85600	-0.40300
H	1.12000	-5.27900	0.28500
H	0.57600	-4.63100	-1.26700

Pro-*S* L13

Pd	-1.22792	0.01736	0.37522
C	-2.83785	1.42485	0.37904
C	-3.94940	1.54097	1.22929
C	-2.22590	2.64659	-0.12419
C	-2.75398	3.93276	0.24502
C	-3.00284	-0.26667	-0.81094
C	-3.38180	-1.60271	-0.35207
C	-3.38734	0.10481	-2.11535
C	-4.04764	-2.52659	-1.23408
C	-4.06271	-0.83721	-2.94414
C	-3.41175	-3.37890	1.35821
C	-4.36815	-3.84216	-0.79573
C	-4.05597	-4.27154	0.47129
H	-3.17167	-3.69786	2.36919
H	-4.87188	-4.50464	-1.49640
H	-4.30673	-5.27898	0.79260
C	-3.09403	-2.09970	0.95634
H	-2.61330	-1.43238	1.65977
C	-4.37606	-2.10774	-2.54126
H	-4.88531	-2.79582	-3.21194
C	-3.19987	1.45803	-2.76832
H	-3.69373	1.46621	-3.74494
H	-2.15001	1.71367	-2.93106
H	-3.63132	2.26545	-2.17115
H	-4.33914	-0.51887	-3.94626
C	-2.14430	5.12379	-0.24022
C	-1.04163	5.08112	-1.05897
C	-0.49988	3.82579	-1.42080
C	-1.07180	2.65816	-0.96401
C	-3.87650	3.99408	1.10202
H	-4.28476	4.96275	1.37998
C	-4.43706	2.83454	1.57027
H	-5.30177	2.89114	2.22732
H	-2.57398	6.07710	0.06009
H	-0.58663	5.99896	-1.42207
H	0.37710	3.77558	-2.06075
H	-0.63175	1.70639	-1.25179
C	-4.72964	0.39194	1.82888
H	-4.13411	-0.19612	2.53451
H	-5.59523	0.77497	2.37838
H	-5.09828	-0.29938	1.06693
P	0.69226	0.05601	1.88167
C	2.17410	-0.97533	1.52083
C	1.37280	1.73875	2.51020
C	0.51343	-0.88578	3.52634
C	2.91359	-1.15459	0.33664
C	2.53512	-1.70052	2.67129
C	2.51421	1.58640	3.53234
C	1.89348	2.54380	1.30719
C	0.18151	2.48316	3.14621
O	1.77645	-1.54355	3.79081
C	4.01201	-2.02878	0.36403
C	2.56994	-0.48032	-0.95666
C	3.63422	-2.55824	2.69976
H	3.37079	1.05824	3.10176
H	2.85693	2.58546	3.83355
H	2.21047	1.05712	4.44076
H	2.77884	2.08177	0.86242
H	1.12959	2.66457	0.53527
H	2.17949	3.54848	1.64854
H	-0.63418	2.62537	2.43072
H	-0.22085	1.96063	4.02285
H	0.51276	3.47397	3.48371
C	4.37140	-2.71141	1.52831
H	4.57776	-2.18517	-0.54814
C	1.49164	-0.95445	-1.73028

C	3.34385	0.59889	-1.43040
H	3.87949	-3.09259	3.61169
H	5.22553	-3.38356	1.51605
C	1.16288	-0.35351	-2.95906
O	0.73887	-1.97126	-1.18467
C	3.01762	1.24232	-2.63875
O	4.33679	1.04535	-0.58499
C	1.94168	0.73118	-3.37084
C	0.65398	-3.26420	-1.84243
C	5.70607	1.23016	-1.01916
H	1.67522	1.22771	-4.30217
H	0.02142	-3.16169	-2.73184
H	5.76287	2.13253	-1.63836
C	-0.59508	-1.92948	3.57373
H	-1.57422	-1.44106	3.57379
H	-0.54807	-2.61063	2.71832
H	-0.49959	-2.51579	4.49457
C	6.49140	1.46744	0.26615
H	6.44300	0.58301	0.91007
H	7.54138	1.68089	0.03737
H	6.07638	2.31742	0.81676
C	6.24066	0.04584	-1.82179
H	5.62058	-0.15944	-2.70043
H	7.25651	0.26722	-2.16848
H	6.27844	-0.85718	-1.20475
C	-0.04396	-4.17751	-0.84299
H	-0.21540	-5.16307	-1.29033
H	0.57422	-4.30408	0.05277
H	-1.00904	-3.75984	-0.54439
C	2.02532	-3.79228	-2.25899
H	1.90898	-4.76575	-2.74909
H	2.52177	-3.11744	-2.96423
H	2.67244	-3.91759	-1.38483
H	0.37227	-0.16702	4.33779
C	3.72540	2.48620	-3.12575
H	3.88159	3.20154	-2.31068
H	4.70717	2.27326	-3.56859
H	3.12705	2.98234	-3.89664
C	-0.01165	-0.80086	-3.79677
H	-0.29355	-0.01416	-4.50428
H	0.21620	-1.69771	-4.38800
H	-0.88497	-1.02557	-3.17686

Pro-*R* L13

Pd	1.18473	0.09295	-0.39908
C	2.56539	1.51291	0.39507
C	3.15937	1.68131	1.66043
C	2.13291	2.71209	-0.31996
C	2.24850	4.01155	0.29029
C	3.30608	-0.23019	-0.49102
C	3.48845	-1.46608	0.25673
C	4.25092	0.10027	-1.47479
C	4.61237	-2.32155	-0.01897
C	5.34759	-0.77722	-1.71267
C	2.75898	-3.11661	1.92704
C	4.77919	-3.54127	0.69520
C	3.87707	-3.93792	1.65206
H	2.03333	-3.42555	2.67404
H	5.64247	-4.15912	0.45738
H	4.01485	-4.87282	2.18896
C	2.57480	-1.92945	1.25211
H	1.69689	-1.32639	1.47373
C	5.53765	-1.94216	-1.01780
H	6.39265	-2.58140	-1.22439
C	4.24871	1.34933	-2.32889
H	5.14999	1.37783	-2.94907
H	3.39007	1.39445	-3.00561
H	4.23023	2.26106	-1.72679
H	6.06534	-0.49270	-2.47836
C	1.78093	5.17276	-0.38690
C	1.20978	5.09368	-1.63345
C	1.09780	3.83041	-2.25793
C	1.54468	2.69230	-1.62311
C	2.83447	4.11670	1.57057
H	2.93874	5.09623	2.03151
C	3.27589	2.98933	2.21129
H	3.73931	3.08526	3.19016
H	1.89007	6.13322	0.11242
H	0.85767	5.98868	-2.13954
H	0.66314	3.75319	-3.25105
H	1.45830	1.73981	-2.13197
C	3.74824	0.59433	2.53384
H	3.00569	-0.13507	2.86691
H	4.19399	1.04048	3.42825
H	4.53044	0.03138	2.01748
P	-0.56354	-1.21829	-1.46435
C	-2.04385	-1.84200	-0.55859
C	-1.22273	-0.45791	-3.10450
C	-0.22204	-3.00549	-2.01207
C	-2.87534	-1.23831	0.40571
C	-2.29864	-3.17128	-0.94694
C	-2.37087	-1.26988	-3.72966
C	-1.71545	0.97309	-2.82275
C	-0.03037	-0.39451	-4.08199

O	-1.45634	-3.74849	-1.84623
H	-0.00958	-3.01748	-3.08378
C	-3.95960	-1.97604	0.90457
C	-2.64149	0.15284	0.91435
C	-3.38061	-3.89936	-0.45304
H	-3.23731	-1.31135	-3.06252
H	-2.69033	-0.78470	-4.66200
H	-2.07999	-2.29650	-3.97331
H	-2.62134	0.97300	-2.21297
H	-0.95575	1.58035	-2.32056
H	-1.95825	1.46284	-3.77645
H	0.81689	0.15332	-3.65596
H	0.32838	-1.38620	-4.37946
H	-0.34229	0.12449	-4.99774
C	-4.21317	-3.28095	0.47559
H	-4.60196	-1.52196	1.65081
C	-1.70175	0.39317	1.93828
C	-3.36211	1.23666	0.37329
H	-3.54209	-4.91924	-0.78632
H	-5.06039	-3.82623	0.88357
C	-1.40664	1.70450	2.35432
O	-1.03247	-0.69302	2.46826
C	-3.08383	2.55891	0.76482
O	-4.25477	0.93606	-0.63689
C	-2.10784	2.74806	1.74611
C	-1.25836	-1.01712	3.86345
C	-5.65027	1.31513	-0.54838
H	-1.85617	3.76825	2.02930
H	-1.05530	-0.12685	4.46958
H	-5.72969	2.40307	-0.65332
C	0.88758	-3.72157	-1.25644
H	0.94320	-4.76211	-1.59573
H	1.85194	-3.23810	-1.43450
H	0.70471	-3.71600	-0.17873
C	-6.31200	0.65468	-1.75254
H	-7.37036	0.93266	-1.80578
H	-5.82347	0.96975	-2.67995
H	-6.23809	-0.43540	-1.67668
C	-6.29209	0.90008	0.77381
H	-5.76029	1.32866	1.62963
H	-7.32805	1.25649	0.80658
H	-6.29903	-0.18928	0.87727
C	-0.23836	-2.09464	4.20713
H	0.78012	-1.73446	4.03242
H	-0.32900	-2.38223	5.26031
H	-0.40324	-2.98398	3.58873
C	-2.69403	-1.46940	4.12291
H	-2.82687	-1.68310	5.19008
H	-3.41026	-0.68930	3.84493
H	-2.92693	-2.37330	3.55203
C	-0.32310	2.01491	3.36030
H	0.12475	2.98977	3.14471
H	-0.70395	2.05066	4.39022
H	0.47593	1.26989	3.32197
C	-3.72718	3.75933	0.10975
H	-3.82766	3.61688	-0.97155
H	-4.72712	3.97637	0.50860
H	-3.11379	4.65074	0.27452

Pro-*S* L14

Pd	-1.05181	-0.56809	-0.01166
C	-2.49606	-1.46467	1.31153
C	-2.50558	-2.68353	2.00569
C	-3.11242	-0.30495	1.94040
C	-3.69987	-0.41434	3.24905
C	-2.58313	-1.82555	-0.74557
C	-1.76279	-2.79161	-1.47320
C	-3.88374	-1.57028	-1.21839
C	-2.25285	-3.39799	-2.68378
C	-4.33535	-2.21890	-2.40300
C	0.34172	-4.02493	-1.82564
C	-1.42958	-4.28882	-3.42893
C	-0.15430	-4.59490	-3.02111
H	1.34116	-4.27708	-1.48050
H	-1.83953	-4.72410	-4.33778
H	0.46383	-5.27508	-3.60097
C	-0.43571	-3.16271	-1.08372
H	-0.03621	-2.75555	-0.16164
C	-3.55934	-3.08935	-3.12349
H	-3.94019	-3.55380	-4.03007
C	-4.91141	-0.66902	-0.56861
H	-5.85725	-0.73134	-1.11590
H	-4.60851	0.38125	-0.55434
H	-5.10638	-0.95411	0.46876
H	-5.34489	-2.00285	-2.74446
C	-4.27638	0.72661	3.87462
C	-4.28139	1.95478	3.25859
C	-3.70101	2.08439	1.97534
C	-3.14027	0.99361	1.34686
C	-3.68270	-1.66533	3.90738
H	-4.13251	-1.75640	4.89314
C	-3.10310	-2.74629	3.29776
H	-3.09894	-3.70539	3.81015
H	-4.71053	0.60204	4.86436

H	-4.72348	2.81778	3.74964
H	-3.69213	3.04866	1.47434
H	-2.69929	1.12531	0.36192
C	-1.95062	-3.99915	1.50411
H	-0.86151	-3.98239	1.39766
H	-2.19313	-4.79903	2.21060
H	-2.36332	-4.27784	0.53146
P	1.01616	0.54437	0.51916
C	1.89627	1.61396	-0.69105
C	1.22579	1.39242	2.22439
C	2.46027	-0.64381	0.46986
C	1.37749	2.67284	-1.45589
C	3.25585	1.24287	-0.82082
C	2.67255	1.82920	2.51429
C	0.30520	2.62511	2.26199
C	0.76152	0.37377	3.28434
H	2.68957	-1.04231	1.45751
C	2.25263	3.39185	-2.28381
C	-0.07276	3.04234	-1.48070
C	4.11828	1.96990	-1.64887
H	3.04566	2.53187	1.76265
H	2.70628	2.33489	3.48877
H	3.36399	0.98150	2.56375
H	0.62360	3.38692	1.54437
H	-0.73658	2.36105	2.05593
H	0.34372	3.07168	3.26506
H	-0.27344	0.05992	3.11787
H	1.39121	-0.52429	3.30151
H	0.82271	0.83305	4.27977
C	3.59960	3.04894	-2.36367
H	1.86193	4.21505	-2.87380
C	-0.99822	2.25056	-2.19263
C	-0.52466	4.25265	-0.92035
H	5.15826	1.69048	-1.72780
H	4.26144	3.61651	-3.01270
C	-2.34039	2.64105	-2.31217
O	-0.49487	1.11542	-2.75473
C	-1.86549	4.64757	-1.02654
O	0.42943	4.99406	-0.27896
C	-2.75610	3.83331	-1.72390
H	-3.04923	2.03034	-2.85727
C	-1.31415	0.37984	-3.65954
H	-2.21183	5.57490	-0.58719
C	0.05562	6.24116	0.28464
H	-3.79519	4.13806	-1.81633
H	-0.68384	-0.42669	-4.03592
H	-2.18423	-0.05335	-3.15530
H	-1.64156	1.01367	-4.49370
H	0.96833	6.65723	0.71503
H	-0.33204	6.92810	-0.47884
H	-0.69519	6.11893	1.07612
H	2.20839	-1.48574	-0.18537
N	3.61450	0.09156	-0.07300
C	4.90330	-0.39247	0.05289
O	5.90307	0.14894	-0.39095
O	4.88622	-1.55357	0.75021
C	6.13361	-2.28105	1.04313
C	7.05576	-1.41847	1.91180
H	7.92207	-2.01136	2.22648
H	7.40842	-0.54334	1.36429
H	6.52810	-1.08538	2.81255
C	6.80461	-2.72214	-0.26236
H	7.15304	-1.86261	-0.83655
H	7.66176	-3.36560	-0.03374
H	6.10224	-3.29790	-0.87535
C	5.62893	-3.49492	1.82891
H	5.11230	-3.17928	2.74134
H	4.93303	-4.08588	1.22445
H	6.47106	-4.13489	2.11229

Pro-*R* L14

Pd	-1.07001	-0.65560	0.10615
C	-2.72448	-1.90144	-0.34357
C	-3.13351	-2.57806	-1.50756
C	-3.74139	-1.20814	0.44324
C	-5.10515	-1.15210	-0.01582
C	-0.98882	-2.78457	0.40659
C	0.13400	-3.18335	-0.43157
C	-1.30848	-3.58385	1.51453
C	0.90533	-4.35295	-0.10585
C	-0.52262	-4.73916	1.79321
C	1.66033	-2.81803	-2.32931
C	2.02623	-4.71902	-0.90307
C	2.40466	-3.97332	-1.99355
H	1.94863	-2.21843	-3.18893
H	2.58371	-5.61003	-0.62213
H	3.26275	-4.26513	-2.59330
C	0.56928	-2.44433	-1.57412
H	0.01835	-1.54718	-1.84590
C	0.54418	-5.12201	1.02415
H	1.11747	-6.01269	1.26972
C	-2.45913	-3.35806	2.47135
H	-2.53652	-4.19998	3.16627
H	-2.33408	-2.45328	3.07441

H	-3.41581	-3.26593	1.95148
H	-0.79726	-5.34059	2.65650
C	-6.08448	-0.42920	0.72154
C	-5.76230	0.23206	1.88163
C	-4.43219	0.17976	2.35812
C	-3.46573	-0.51617	1.66568
C	-5.45376	-1.82560	-1.20748
H	-6.48434	-1.79730	-1.55364
C	-4.49898	-2.51707	-1.90687
H	-4.78613	-3.04805	-2.81134
H	-7.10358	-0.41523	0.34089
H	-6.52062	0.77979	2.43489
H	-4.16838	0.68438	3.28388
H	-2.45811	-0.55076	2.06480
C	-2.25790	-3.43019	-2.40135
H	-1.47728	-2.85751	-2.90930
H	-2.86964	-3.90685	-3.17366
H	-1.75147	-4.22089	-1.84103
P	0.75645	0.80401	0.64601
C	1.30169	2.18436	-0.43902
C	0.82291	1.47031	2.44108
C	2.43189	0.00300	0.44308
C	0.51537	3.18735	-1.03307
C	2.69692	2.14147	-0.67430
C	2.09125	2.28903	2.73674
C	-0.41900	2.34986	2.67291
C	0.75758	0.24502	3.37504
H	2.80706	-0.38447	1.39017
C	1.15311	4.17204	-1.80147
C	-0.97608	3.23808	-0.91900
C	3.32086	3.13465	-1.43876
H	2.17317	3.15919	2.07803
H	2.05622	2.65481	3.77196
H	3.00477	1.69443	2.63098
H	-0.37048	3.27057	2.08585
H	-1.34589	1.82477	2.41621
H	-0.47269	2.63049	3.73385
H	-0.13226	-0.36366	3.18155
H	1.63419	-0.40518	3.27499
H	0.71973	0.58400	4.41871
C	2.53292	4.14589	-1.98660
H	0.55376	4.95317	-2.25869
C	-1.78604	2.37257	-1.68277
C	-1.60999	4.22884	-0.14445
H	4.38814	3.10364	-1.59901
H	3.00991	4.91725	-2.58579
C	-3.18460	2.45765	-1.62952
O	-1.11861	1.48266	-2.47439
C	-3.00611	4.32482	-0.08657
O	-0.76835	5.06559	0.53938
C	-3.77541	3.42958	-0.82689
H	-3.80558	1.77518	-2.19582
C	-1.86806	0.71499	-3.40834
H	-3.49044	5.07751	0.52320
C	-1.33339	6.12451	1.29534
H	-4.85900	3.49116	-0.77819
H	-1.13037	0.16316	-3.99403
H	-2.53570	0.00538	-2.90637
H	-2.45049	1.36304	-4.07598
H	-0.48816	6.67455	1.71327
H	-1.92853	6.79688	0.66389
H	-1.96110	5.74849	2.11388
H	2.34791	-0.83776	-0.25176
N	3.34920	1.02358	-0.09602
C	4.70852	0.77885	-0.17495
O	4.97731	-0.43386	0.36191
O	5.53633	1.54136	-0.64802
C	6.34530	-0.97779	0.37485
C	6.14602	-2.34552	1.03490
H	5.44409	-2.95463	0.45610
H	7.10210	-2.87617	1.09555
H	5.74841	-2.23219	2.04889
C	6.85965	-1.13943	-1.05995
H	6.98669	-0.17054	-1.54504
H	7.82570	-1.65670	-1.04613
H	6.15934	-1.74293	-1.64803
C	7.25834	-0.08866	1.22594
H	6.83496	0.04205	2.22810
H	8.23932	-0.56576	1.33199
H	7.39117	0.89234	0.76745

Pro-*S* L15

Pd	1.11779	-0.74750	-0.14365
C	2.62878	-1.45216	-1.51768
C	2.73709	-2.62538	-2.28068
C	3.14897	-0.21286	-2.08024
C	3.75164	-0.20349	-3.38697
C	2.74677	-1.92384	0.50982
C	2.02829	-3.02215	1.15223
C	4.01791	-1.58292	1.00788
C	2.59327	-3.69490	2.29295
C	4.54484	-2.29319	2.12357
C	0.04428	-4.46189	1.41216
C	1.86850	-4.72632	2.95440

C	0.61599	-5.10327	2.53557
H	-0.93876	-4.76327	1.05936
H	2.33418	-5.20928	3.81081
H	0.07252	-5.88940	3.05287
C	0.72836	-3.46669	0.74857
H	0.27295	-3.00437	-0.12040
C	3.87079	-3.30653	2.75354
H	4.30834	-3.81916	3.60701
C	4.94196	-0.52296	0.44900
H	5.88244	-0.51868	1.00894
H	4.51930	0.48320	0.50815
H	5.18096	-0.70296	-0.60283
H	5.53072	-2.00929	2.48390
C	4.23754	1.01153	-3.94610
C	4.13593	2.20216	-3.26820
C	3.53438	2.21633	-1.98853
C	3.06163	1.05184	-1.42304
C	3.83868	-1.41308	-4.11280
H	4.29960	-1.41341	-5.09764
C	3.34229	-2.56776	-3.56950
H	3.41540	-3.49386	-4.13455
H	4.68783	0.97423	-4.93578
H	4.50896	3.12311	-3.70878
H	3.43660	3.14925	-1.44051
H	2.59987	1.09763	-0.43993
C	2.29127	-4.01004	-1.86280
H	1.20766	-4.08280	-1.73189
H	2.57343	-4.73810	-2.62956
H	2.74891	-4.32506	-0.92161
P	-1.04343	0.21376	-0.62511
C	-2.00880	1.25199	0.54948
C	-1.31685	0.99384	-2.35458
C	-2.39481	-1.07668	-0.53925
C	-1.57495	2.36614	1.28864
C	-3.34111	0.79147	0.67484
C	-2.78810	1.33718	-2.64631
C	-0.46915	2.27561	-2.43467
C	-0.80182	-0.02592	-3.38969
H	-2.58590	-1.52635	-1.51301
C	-2.51114	3.05376	2.07490
C	-0.14620	2.80757	1.34092
C	-4.26558	1.48771	1.46172
H	-3.19759	2.03922	-1.91341
H	-2.85634	1.81036	-3.63523
H	-3.42916	0.44967	-2.66553
H	-0.81669	3.03362	-1.72679
H	0.58931	2.07698	-2.24150

H	-0.54990	2.69735	-3.44618
H	0.24907	-0.27955	-3.22046
H	-1.38266	-0.95646	-3.38124
H	-0.89116	0.40373	-4.39617
C	-3.83422	2.62542	2.14305
H	-2.18563	3.91852	2.64481
C	0.78512	2.06582	2.09959
C	0.27066	4.01865	0.75304
H	-5.28509	1.14005	1.53635
H	-4.54508	3.16978	2.75944
C	2.11616	2.49449	2.21647
O	0.29345	0.93757	2.68564
C	1.59876	4.45446	0.87307
O	-0.69913	4.69470	0.06536
C	2.50332	3.68149	1.59928
H	2.83649	1.92075	2.78623
C	1.00306	0.28108	3.76091
H	1.92769	5.38207	0.42169
C	-0.46846	6.02786	-0.42963
H	3.53210	4.01880	1.69496
H	2.02282	0.05367	3.42485
H	0.50627	6.05787	-0.93434
H	-2.08595	-1.87411	0.14691
N	-3.60589	-0.41073	-0.03081
C	-4.85216	-0.99905	-0.13928
O	-5.89386	-0.52019	0.27953
O	-4.74068	-2.18302	-0.78787
C	-5.92587	-3.01717	-1.05297
C	-6.90883	-2.26525	-1.95707
H	-7.72390	-2.93685	-2.24971
H	-7.33218	-1.39988	-1.44535
H	-6.40442	-1.92715	-2.86920
C	-6.56624	-3.45654	0.26842
H	-6.98326	-2.60439	0.80678
H	-7.36953	-4.17373	0.06500
H	-5.82378	-3.95037	0.90519
C	-5.32418	-4.21843	-1.78820
H	-4.83078	-3.90054	-2.71263
H	-4.58597	-4.72725	-1.15969
H	-6.11211	-4.93405	-2.04536
C	-1.56473	6.27586	-1.46047
H	-2.55164	6.19754	-0.99204
H	-1.46131	7.27831	-1.88967
H	-1.50846	5.54247	-2.27031
C	-0.50323	7.04302	0.71401
H	0.25428	6.82294	1.47190
H	-0.32053	8.05342	0.33088

H	-1.48667	7.02924	1.19670
C	0.25499	-1.02587	3.99515
H	0.75277	-1.60928	4.77680
H	-0.77355	-0.82154	4.31298
H	0.22738	-1.62638	3.08264
C	1.03092	1.15851	5.01372
H	1.53109	2.11469	4.83573
H	0.00818	1.36271	5.34961
H	1.56229	0.64082	5.82033

Pro-*R* L15

Pd	0.74763	-1.15356	-0.15073
C	2.15887	-2.69740	0.20670
C	2.35262	-3.59706	1.27125
C	3.33772	-2.06663	-0.38326
C	4.64591	-2.30193	0.17154
C	0.40759	-3.17513	-0.81683
C	-0.83475	-3.53529	-0.14537
C	0.72196	-3.82802	-2.01958
C	-1.71890	-4.51379	-0.71990
C	-0.18245	-4.79287	-2.55023
C	-2.46689	-3.27100	1.68071
C	-2.94436	-4.84221	-0.07480
C	-3.31959	-4.24004	1.10207
H	-2.74913	-2.78727	2.61244
H	-3.58421	-5.58705	-0.54313
H	-4.25629	-4.50458	1.58570
C	-1.27557	-2.93624	1.07350
H	-0.63954	-2.18348	1.53176
C	-1.35919	-5.13376	-1.93826
H	-2.01950	-5.87938	-2.37445
C	1.98121	-3.63495	-2.83697
H	2.00624	-4.35850	-3.65768
H	2.04637	-2.63843	-3.28443
H	2.88553	-3.77692	-2.24043
H	0.09087	-5.28128	-3.48250
C	5.78836	-1.65308	-0.37574
C	5.67987	-0.79134	-1.44033
C	4.40655	-0.55158	-2.00569
C	3.28523	-1.16669	-1.49451
C	4.77628	-3.18863	1.26340
H	5.76219	-3.37812	1.68159
C	3.66757	-3.81248	1.77289
H	3.78563	-4.50910	2.59942
H	6.75822	-1.86338	0.07024
H	6.56159	-0.30669	-1.85149
H	4.30823	0.12141	-2.85318
H	2.32397	-0.97083	-1.95641
C	1.28474	-4.42894	1.94862
H	0.55470	-3.82771	2.49722
H	1.74873	-5.11256	2.66637
H	0.72040	-5.03084	1.23125
P	-0.78915	0.64504	-0.60733
C	-0.97802	2.13595	0.45688
C	-0.87093	1.25307	-2.42313
C	-2.56748	0.18709	-0.25768
C	0.02325	2.99885	0.93757
C	-2.33120	2.35071	0.81605
C	-1.96913	2.30476	-2.65586
C	0.49665	1.85240	-2.79587
C	-1.13248	0.01091	-3.29903
H	-3.08280	-0.12708	-1.16509
C	-0.35693	4.09018	1.73276
C	1.48318	2.81269	0.66367
C	-2.69558	3.44776	1.60560
H	-1.81289	3.19445	-2.03779
H	-1.95511	2.62031	-3.70808
H	-2.97086	1.91518	-2.44568
H	0.68679	2.78276	-2.25563
H	1.31718	1.15766	-2.58620
H	0.51330	2.07819	-3.87107
H	-0.37405	-0.76312	-3.14066
H	-2.11295	-0.43931	-3.10673
H	-1.10975	0.30215	-4.35749
C	-1.69425	4.30914	2.05069
H	0.41100	4.76228	2.10326
C	2.23195	1.84204	1.36040
C	2.16284	3.68991	-0.20747
H	-3.73195	3.60938	1.86143
H	-1.96865	5.15933	2.67003
C	3.61286	1.71716	1.15170
O	1.52235	1.05277	2.22228
C	3.54374	3.57470	-0.41242
O	1.36914	4.61214	-0.83697
C	4.24784	2.58205	0.26646
H	4.18561	0.95149	1.65932
C	2.19332	0.35064	3.29231
H	4.06824	4.23969	-1.08724
C	1.94374	5.70504	-1.57784
H	5.31660	2.47927	0.10067
H	2.99801	-0.26282	2.86850
H	2.72459	5.31515	-2.24448
H	-2.59733	-0.65226	0.44246
N	-3.22722	1.36738	0.32710

C	-4.59851	1.39111	0.50756
O	-5.13484	0.25184	0.01152
O	-5.22735	2.30000	1.02639
C	-6.58020	-0.01404	0.09701
C	-6.69770	-1.39136	-0.56286
H	-6.08156	-2.12688	-0.03548
H	-7.73933	-1.72913	-0.54456
H	-6.36552	-1.35081	-1.60549
C	-7.01563	-0.07668	1.56525
H	-6.91223	0.89594	2.04865
H	-8.06420	-0.38991	1.62327
H	-6.41135	-0.81076	2.10974
C	-7.35956	1.03914	-0.69813
H	-6.98779	1.08993	-1.72761
H	-8.41905	0.76095	-0.73386
H	-7.26906	2.02529	-0.24047
C	0.80563	6.26267	-2.42625
H	1.16034	7.10335	-3.03226
H	0.40874	5.49341	-3.09566
H	-0.00935	6.61455	-1.78478
C	2.53362	6.75155	-0.63121
H	3.31433	6.32398	0.00468
H	2.97188	7.57754	-1.20286
H	1.74717	7.15712	0.01483
C	2.75570	1.33414	4.31970
H	3.25488	0.78842	5.12845
H	3.48254	2.01738	3.87104
H	1.94406	1.92938	4.75272
C	1.14158	-0.57302	3.89374
H	0.30069	0.00890	4.28679
H	0.76583	-1.26392	3.13441
H	1.57624	-1.15718	4.71213

Pro-*S* L16

Pd	0.99112	-0.50069	-0.14760
C	2.70007	-0.80963	-1.40497
C	3.02606	-1.86915	-2.26724
C	3.07979	0.54025	-1.79895
C	3.77198	0.77060	-3.03906
C	2.78225	-1.43325	0.57338
C	2.22657	-2.68425	1.08915
C	3.95095	-0.93885	1.18797
C	2.82325	-3.34869	2.21911
C	4.51475	-1.64083	2.29230
C	0.50212	-4.44578	1.09745
C	2.24395	-4.53593	2.74922
C	1.10248	-5.07776	2.21059
H	-0.39339	-4.86997	0.65080
H	2.73079	-5.00468	3.60178
H	0.66936	-5.98347	2.62681
C	1.04982	-3.30040	0.56284
H	0.57784	-2.84307	-0.29718
C	3.98302	-2.79520	2.80335
H	4.44546	-3.29404	3.65185
C	4.74212	0.28620	0.78168
H	5.64942	0.35634	1.38975
H	4.18482	1.21647	0.91704
H	5.04756	0.24953	-0.26697
H	5.41555	-1.23110	2.74266
C	4.13117	2.09245	-3.42550
C	3.82375	3.17649	-2.63910
C	3.13042	2.97120	-1.42382
C	2.77092	1.70109	-1.02807
C	4.07830	-0.33112	-3.86969
H	4.60629	-0.16471	-4.80556
C	3.70830	-1.59332	-3.48668
H	3.94958	-2.43507	-4.13143
H	4.65532	2.22424	-4.36969
H	4.10520	4.18028	-2.94661
H	2.87279	3.81728	-0.79232
H	2.23392	1.57308	-0.09192
C	2.74243	-3.33536	-2.02378
H	1.67127	-3.55754	-1.99699
H	3.17685	-3.93699	-2.82819
H	3.16621	-3.68720	-1.07982
P	-1.24221	0.31283	-0.73015
C	-2.33990	0.97248	0.58670
C	-1.41023	1.54337	-2.19843
C	-2.55676	-0.97952	-1.18197
C	-2.04943	1.89034	1.61213
C	-3.64713	0.43761	0.45987
C	-2.86957	1.88133	-2.55222
C	-0.68428	2.84720	-1.82790
C	-0.70896	0.89040	-3.40591
H	-2.68002	-0.95935	-2.27030
C	-2.19141	-2.40679	-0.76169
C	-3.09220	2.32573	2.44485
C	-0.67474	2.40827	1.90177
C	-4.68306	0.90905	1.28160
H	-3.40567	2.31018	-1.69999
H	-2.87641	2.62548	-3.36019
H	-3.43238	1.01263	-2.90732
H	-1.18062	3.36125	-1.00017
H	0.35995	2.67096	-1.55946

H	-0.69382	3.52095	-2.69601
H	0.34380	0.67939	-3.19459
H	-1.18941	-0.04771	-3.71109
H	-0.75314	1.57247	-4.26532
C	-4.39026	1.85302	2.26193
H	-2.87510	3.03824	3.23397
C	0.27670	1.58995	2.54519
C	-0.34529	3.76040	1.68077
H	-5.69561	0.54299	1.15735
H	-5.18991	2.21411	2.90393
C	1.52966	2.09633	2.92287
O	-0.11229	0.30452	2.78319
C	0.90795	4.27321	2.04289
O	-1.32588	4.51720	1.09992
C	1.83014	3.43049	2.66136
H	2.25953	1.46197	3.40977
C	0.67981	-0.49302	3.65939
H	1.16066	5.31086	1.86286
C	-1.07276	5.89251	0.86438
H	2.80075	3.82439	2.95090
H	0.14115	-1.43540	3.76621
H	1.67012	-0.69445	3.23995
H	0.78084	-0.01024	4.64007
H	-1.98671	6.29009	0.41927
H	-0.86045	6.42919	1.79828
H	-0.23752	6.03694	0.16665
H	-3.02845	-3.08700	-0.95405
H	-1.33079	-2.75528	-1.34039
H	-1.93667	-2.46162	0.30156
N	-3.80764	-0.51592	-0.54388
C	-4.99462	-1.27926	-0.73041
C	-5.54326	-1.39600	-2.01627
C	-5.60953	-1.95862	0.33387
C	-6.68128	-2.17289	-2.23309
H	-5.07829	-0.86700	-2.84301
C	-6.75922	-2.71757	0.11532
H	-5.17852	-1.89169	1.32783
C	-7.29877	-2.83156	-1.16795
H	-7.09311	-2.25172	-3.23575
H	-7.22462	-3.23501	0.95017
H	-8.19014	-3.42935	-1.33621

Pro-*R* L16

Pd	-1.09962	0.40800	-0.20807
C	-3.22952	0.43704	-0.07688
C	-4.14480	0.66961	0.96837
C	-3.52904	-0.64095	-1.01702
C	-4.65827	-1.50867	-0.80177
C	-2.19851	2.22152	-0.50340
C	-1.72351	3.09547	0.55817
C	-2.70003	2.80667	-1.67511
C	-1.75995	4.52420	0.39486
C	-2.71623	4.22615	-1.79487
C	-0.71569	3.46123	2.77118
C	-1.27925	5.37573	1.42905
C	-0.76923	4.86404	2.59796
H	-0.30312	3.04533	3.68706
H	-1.32264	6.45081	1.26827
H	-0.40565	5.52618	3.37931
C	-1.17289	2.61581	1.78427
H	-1.10127	1.54067	1.92763
C	-2.27041	5.06505	-0.80748
H	-2.30752	6.14433	-0.93443
C	-3.26454	2.06292	-2.86552
H	-3.70240	2.77148	-3.57556
H	-2.49759	1.49853	-3.40650
H	-4.04400	1.35392	-2.57812
H	-3.11173	4.65125	-2.71435
C	-4.92046	-2.58832	-1.69093
C	-4.11695	-2.83189	-2.77781
C	-3.01319	-1.98268	-3.01943
C	-2.73629	-0.93310	-2.17078
C	-5.50592	-1.26739	0.30095
H	-6.36365	-1.91494	0.46738
C	-5.25682	-0.20653	1.13066
H	-5.93807	-0.00959	1.95497
H	-5.78498	-3.21725	-1.48910
H	-4.33113	-3.65756	-3.45127
H	-2.37929	-2.15096	-3.88605
H	-1.89224	-0.29087	-2.38955
C	-4.12523	1.81780	1.95681
H	-3.27466	1.78892	2.64231
H	-5.03595	1.79509	2.56338
H	-4.08557	2.78707	1.45290
P	1.28228	-0.04169	-0.52395
C	2.23438	-0.99709	0.73508
C	1.71235	-0.80027	-2.23413
C	2.54727	1.36746	-0.36027
C	1.84161	-2.10986	1.50018
C	3.49957	-0.39767	0.95598
C	3.21285	-1.07527	-2.43227
C	0.94298	-2.12589	-2.37812
C	1.22253	0.19713	-3.30368
H	2.76606	1.76155	-1.35675

C	2.72975	-2.61454	2.46274
C	0.52125	-2.80024	1.34849
C	4.35851	-0.89573	1.94476
H	3.60515	-1.74600	-1.66119
H	3.36277	-1.56392	-3.40483
H	3.81490	-0.16208	-2.43060
H	1.32542	-2.87991	-1.68575
H	-0.13027	-2.00688	-2.20132
H	1.07537	-2.50990	-3.39940
H	0.15584	0.41932	-3.19499
H	1.76705	1.14787	-3.26835
H	1.38098	-0.23204	-4.30186
C	3.96602	-2.00963	2.68215
H	2.43281	-3.47834	3.04925
C	-0.62308	-2.33730	2.02748
C	0.42540	-4.00985	0.63357
H	5.30499	-0.40642	2.14562
H	4.62733	-2.39773	3.45264
C	-1.84298	-3.02311	1.93689
O	-0.44821	-1.21305	2.78364
C	-0.78696	-4.70420	0.53817
O	1.58940	-4.44285	0.05534
C	-1.90966	-4.19279	1.18544
H	-2.73091	-2.64939	2.43054
C	-1.50397	-0.82200	3.65090
H	-0.86239	-5.62369	-0.02899
C	1.58530	-5.69046	-0.61968
H	-2.85671	-4.71882	1.10467
H	-1.12360	0.03164	4.21510
H	-2.39599	-0.52007	3.09022
H	-1.76521	-1.63047	4.34631
H	2.61231	-5.85077	-0.95324
H	1.28809	-6.50839	0.04937
H	0.91930	-5.67757	-1.49238
N	3.76793	0.73765	0.17470
C	5.04562	1.14025	-0.26079
C	5.26869	2.46458	-0.68779
C	6.12168	0.23208	-0.34023
C	6.51969	2.86222	-1.15784
H	4.46752	3.19291	-0.64368
C	7.36978	0.64590	-0.79908
H	5.97277	-0.80448	-0.06184
C	7.58474	1.96307	-1.21037
H	6.65863	3.89222	-1.47642
H	8.17781	-0.07967	-0.85039
H	8.55986	2.27935	-1.56885
C	2.03204	2.48895	0.54691
H	1.75666	2.09924	1.53088
H	2.80086	3.25504	0.69451
H	1.14543	2.95907	0.11449

Pro-*S* L17

Pd	1.24034	-0.51292	-0.30542
C	2.82324	-0.35421	-1.76135
C	3.41099	-1.31688	-2.59876
C	2.68720	1.00518	-2.26809
C	3.14557	1.34043	-3.59068
C	3.28909	-0.74902	0.23672
C	3.25036	-2.07240	0.85569
C	4.26361	0.15897	0.69674
C	4.13853	-2.40640	1.93925
C	5.12808	-0.22141	1.76329
C	2.27278	-4.32165	1.10199
C	4.06633	-3.68210	2.56643
C	3.15206	-4.62585	2.16656
H	1.55225	-5.06338	0.76713
H	4.75936	-3.89165	3.37845
H	3.10721	-5.59582	2.65454
C	2.32692	-3.09514	0.47675
H	1.65225	-2.88953	-0.34569
C	5.07669	-1.44589	2.37509
H	5.75600	-1.69188	3.18790
C	4.54362	1.54224	0.15023
H	5.41784	1.96680	0.65373
H	3.71117	2.23411	0.29929
H	4.75432	1.52479	-0.92198
H	5.86601	0.50418	2.09675
C	3.00573	2.66686	-4.08619
C	2.42729	3.65601	-3.32827
C	1.95608	3.34257	-2.03281
C	2.07837	2.06470	-1.53110
C	3.72595	0.33285	-4.39332
H	4.07746	0.58072	-5.39198
C	3.84152	-0.94055	-3.90382
H	4.29240	-1.70892	-4.52717
H	3.36730	2.87783	-5.09035
H	2.32796	4.66556	-3.71863
H	1.48749	4.10838	-1.42132
H	1.69993	1.85284	-0.53492
C	3.67647	-2.76639	-2.25398
H	2.75596	-3.34055	-2.11138
H	4.23097	-3.24517	-3.06692
H	4.26769	-2.87376	-1.34170
P	-1.20067	-0.51231	-0.61388

C	-2.35902	0.09193	0.69236
C	-1.82676	0.24730	-2.26224
C	-2.09267	-2.18816	-0.54745
C	-2.23094	1.18480	1.56647
C	-3.47612	-0.77456	0.80213
C	-3.33137	0.05364	-2.51614
C	-1.52229	1.75473	-2.23051
C	-1.02001	-0.42200	-3.39276
C	-3.25306	1.42755	2.49834
C	-1.03410	2.08247	1.60790
C	-4.46718	-0.53759	1.76372
H	-3.93975	0.48150	-1.71317
H	-3.60074	0.57240	-3.44631
H	-3.61188	-0.99636	-2.63790
H	-2.08401	2.26238	-1.44067
H	-0.45863	1.95503	-2.08422
H	-1.81439	2.19936	-3.19217
H	0.05659	-0.27558	-3.26413
H	-1.21315	-1.50034	-3.45545
H	-1.31145	0.01726	-4.35598
C	-4.35211	0.57679	2.59126
H	-3.16150	2.27640	3.16867
C	0.13951	1.65725	2.26529
C	-1.10090	3.40528	1.12605
H	-5.29623	-1.22609	1.88041
H	-5.11759	0.76386	3.34011
C	1.24275	2.51657	2.38619
O	0.10540	0.38558	2.75642
C	-0.00381	4.27037	1.25273
O	-2.28595	3.75026	0.53697
C	1.15471	3.80937	1.87670
H	2.15246	2.18725	2.87206
C	1.06698	-0.05904	3.74126
H	-0.04678	5.28873	0.88709
C	-2.56803	5.11695	0.18035
H	2.00571	4.47812	1.97536
H	2.07786	0.11940	3.35419
H	-1.69975	5.53615	-0.34514
C	-1.36304	-3.18119	0.36722
H	-0.40530	-3.47219	-0.06888
H	-1.16602	-2.73934	1.34938
H	-1.96541	-4.08295	0.52052
C	-3.74466	5.05266	-0.78781
H	-4.61021	4.58807	-0.30343
H	-4.02556	6.06121	-1.10990
H	-3.48692	4.46315	-1.67258
C	-2.88306	5.94692	1.42607
H	-2.04946	5.94351	2.13442
H	-3.09152	6.98627	1.14786
H	-3.76584	5.54086	1.93221
C	0.84539	-1.56101	3.87246
H	1.55932	-1.98484	4.58642
H	-0.16970	-1.76442	4.23199
H	0.98639	-2.06055	2.91085
C	0.86109	0.66833	5.07133
H	1.58960	0.31221	5.80851
H	0.98047	1.75083	4.97097
H	-0.14524	0.46751	5.45553
H	-2.17085	-2.59357	-1.56022
N	-3.44735	-1.88901	-0.05108
C	-4.57305	-2.58175	-0.53566
C	-5.84129	-1.96991	-0.61507
C	-4.44803	-3.90116	-1.01521
C	-6.93633	-2.66435	-1.12302
H	-5.96280	-0.94043	-0.29993
C	-5.54993	-4.58061	-1.53322
H	-3.49015	-4.40660	-0.97689
C	-6.80575	-3.97595	-1.58440
H	-7.89945	-2.16254	-1.17314
H	-5.41921	-5.59854	-1.89158
H	-7.66304	-4.51169	-1.98108

Pro-*R* L17

Pd	1.25527	-0.49436	-0.21696
C	3.32924	-0.01264	-0.13073
C	4.28744	-0.12525	0.89649
C	3.36682	1.18658	-0.96555
C	4.32370	2.23159	-0.71012
C	2.77974	-1.90644	-0.81530
C	2.58923	-3.02941	0.09337
C	3.36888	-2.16486	-2.06479
C	3.00094	-4.35640	-0.28166
C	3.74727	-3.49739	-2.39945
C	1.79849	-3.98004	2.22191
C	2.81259	-5.44757	0.61195
C	2.22949	-5.27229	1.84382
H	1.32122	-3.82890	3.18684
H	3.14092	-6.43364	0.29049
H	2.09327	-6.11435	2.51721
C	1.96949	-2.90944	1.37171
H	1.61414	-1.92671	1.67069
C	3.58428	-4.56027	-1.55197
H	3.90034	-5.55933	-1.84222
C	3.69219	-1.14520	-3.13539

H	4.21760	-1.63113	-3.96323
H	2.79841	-0.67384	-3.55452
H	4.33263	-0.34281	-2.76178
H	4.19964	-3.66258	-3.37425
C	4.34011	3.40898	-1.50976
C	3.44439	3.59021	-2.53512
C	2.49203	2.57998	-2.80050
C	2.46162	1.42979	-2.04370
C	5.24204	2.07478	0.35027
H	5.97070	2.85775	0.54727
C	5.21313	0.93613	1.11072
H	5.93534	0.82042	1.91520
H	5.08746	4.16746	-1.28600
H	3.46928	4.49312	-3.13966
H	1.78065	2.70521	-3.61257
H	1.73048	0.66583	-2.27774
C	4.49726	-1.30810	1.81784
H	3.64396	-1.50162	2.47203
H	5.36515	-1.12558	2.45907
H	4.68368	-2.23129	1.26290
P	-1.19847	-0.55337	-0.48521
C	-2.35885	0.37648	0.61246
C	-1.74430	-0.16400	-2.28457
C	-2.16291	-2.13828	-0.08031
C	-2.21373	1.64466	1.20203
C	-3.50045	-0.41266	0.90387
C	-3.25409	-0.31999	-2.53437
C	-1.34553	1.28928	-2.59755
C	-0.96744	-1.12936	-3.20240
H	-2.24720	-2.74450	-0.98675
C	-3.22831	2.11521	2.05045
C	-1.02000	2.52169	0.98990
C	-4.48538	0.06023	1.78129
H	-3.84392	0.31454	-1.86547
H	-3.47823	-0.01284	-3.56522
H	-3.59865	-1.35170	-2.42115
H	-1.93814	1.99874	-2.01414
H	-0.28973	1.48641	-2.39337
H	-1.52718	1.49175	-3.66251
H	0.11488	-1.04439	-3.05983
H	-1.24342	-2.17652	-3.02939
H	-1.19615	-0.90033	-4.25176
C	-4.34315	1.32974	2.33587
H	-3.12223	3.09757	2.50000
C	0.16048	2.31177	1.73112
C	-1.09303	3.64298	0.13844
H	-5.33274	-0.56343	2.04260

H	-5.10348	1.69944	3.01927
C	1.27054	3.15118	1.56114
O	0.12859	1.25620	2.60017
C	0.01013	4.49017	-0.02703
O	-2.29299	3.81122	-0.50155
C	1.18163	4.22296	0.67827
H	2.19394	2.96999	2.09666
C	1.07433	1.17477	3.68623
H	-0.03177	5.34232	-0.69382
C	-2.59045	5.03087	-1.20660
H	2.04609	4.86410	0.53373
H	2.09194	1.22079	3.27696
H	-1.74576	5.28181	-1.86198
N	-3.50839	-1.68698	0.31576
C	-4.65787	-2.41980	-0.03567
C	-4.58453	-3.81433	-0.22635
C	-5.89756	-1.78778	-0.26589
C	-5.70888	-4.54230	-0.61365
H	-3.65010	-4.33825	-0.06283
C	-7.01581	-2.52883	-0.63977
H	-5.97826	-0.71126	-0.17316
C	-6.93670	-3.91203	-0.81530
H	-5.61828	-5.61711	-0.74883
H	-7.95590	-2.01065	-0.81214
H	-7.81186	-4.48425	-1.10862
C	-1.47956	-2.94199	1.03042
H	-1.31286	-2.31772	1.91500
H	-2.10047	-3.79150	1.33493
H	-0.51127	-3.31691	0.69462
C	0.85323	-0.19716	4.31337
H	-0.16635	-0.27584	4.70620
H	0.99930	-0.98868	3.57369
H	1.55776	-0.35485	5.13718
C	0.85431	2.30253	4.69681
H	1.57474	2.21652	5.51822
H	0.97511	3.28845	4.23980
H	-0.15645	2.23785	5.11498
C	-3.80712	4.71612	-2.07092
H	-4.10248	5.60022	-2.64623
H	-3.58740	3.90348	-2.76982
H	-4.65086	4.41160	-1.44242
C	-2.85412	6.17479	-0.22581
H	-1.99091	6.35740	0.42065
H	-3.07555	7.09948	-0.77087
H	-3.71413	5.93273	0.40843

Pro-*S* L18

Pd	-0.59341	0.68153	-0.07177	C	1.73819	-2.92894	-2.88052
C	-1.95828	1.88978	-1.20176	C	-0.67564	-2.81314	-2.23872
C	-1.75178	3.07072	-1.93123	C	0.35027	-0.92537	-3.54884
C	-2.92598	0.92600	-1.70544	O	3.62345	-1.58474	-0.68233
C	-3.64387	1.18546	-2.92502	H	2.96877	-0.40502	-2.21252
C	-1.72324	2.25156	0.84396	C	1.67497	-3.99154	2.04383
C	-0.63569	3.01512	1.45337	C	-0.53490	-2.94553	1.58977
C	-2.98482	2.29761	1.46945	C	3.73531	-3.31642	0.95517
C	-0.83612	3.72638	2.68940	H	1.94216	-3.68498	-2.11583
C	-3.14043	3.03459	2.67924	H	1.43640	-3.45546	-3.79591
C	1.71860	3.74248	1.53580	H	2.67390	-2.40547	-3.09815
C	0.24332	4.41720	3.30872	H	-0.52312	-3.59340	-1.48717
C	1.50057	4.42401	2.75529	H	-1.52207	-2.19193	-1.93606
H	2.70426	3.75167	1.07787	H	-0.94603	-3.30566	-3.18303
H	0.04571	4.94207	4.24093	H	-0.47505	-0.25750	-3.28278
H	2.31642	4.95317	3.24037	H	1.23770	-0.31054	-3.74467
C	0.68672	3.07353	0.91515	H	0.09049	-1.42899	-4.48919
H	0.87335	2.57307	-0.02656	C	3.05362	-4.12006	1.86693
C	-2.11737	3.71744	3.28251	H	1.16321	-4.60797	2.77613
H	-2.28100	4.25998	4.21069	C	-1.02126	-1.87582	2.36879
C	-4.26556	1.65847	0.97682	C	-1.43737	-3.95643	1.20693
H	-5.09770	1.95724	1.62191	H	4.80951	-3.38225	0.81655
H	-4.22452	0.56629	0.98035	H	3.60636	-4.84317	2.46088
H	-4.51045	1.96293	-0.04373	C	-2.37502	-1.80393	2.73060
H	-4.12651	3.04915	3.13706	O	-0.09323	-0.95129	2.74610
C	-4.58047	0.23602	-3.42168	C	-2.79367	-3.88903	1.55452
C	-4.81933	-0.94578	-2.76256	O	-0.89489	-4.98498	0.48647
C	-4.11365	-1.22396	-1.56897	C	-3.24323	-2.81032	2.31453
C	-3.20038	-0.32147	-1.06833	H	-2.74709	-0.97895	3.32493
C	-3.39779	2.38914	-3.62405	C	-0.46058	0.02205	3.72059
H	-3.94113	2.59384	-4.54337	H	-3.48746	-4.66424	1.25337
C	-2.48256	3.28368	-3.13541	C	-1.73814	-6.05464	0.08978
H	-2.30281	4.20863	-3.67814	H	-4.29233	-2.75632	2.59286
H	-5.10380	0.46972	-4.34637	H	0.44489	0.59741	3.91686
H	-5.53850	-1.66103	-3.15312	H	-1.23752	0.69574	3.34639
H	-4.28503	-2.15640	-1.03776	H	-0.79961	-0.46207	4.64560
H	-2.66825	-0.56347	-0.15197	H	-1.09515	-6.75653	-0.44435
C	-0.81717	4.19913	-1.55354	H	-2.18458	-6.55837	0.95698
H	0.23524	3.89984	-1.58199	H	-2.53707	-5.71274	-0.58096
H	-0.93472	5.03072	-2.25519	C	3.19817	0.80353	-0.43904
H	-1.01501	4.58105	-0.54911	H	2.55197	1.59485	-0.83204
P	0.97859	-1.01680	-0.83854	H	2.98122	0.72079	0.63262
C	1.59946	-2.27720	0.34554	C	4.65001	1.17313	-0.66340
C	0.60065	-1.98237	-2.45507	C	5.01745	1.98731	-1.74378
C	2.78563	-0.50114	-1.13881	C	5.65736	0.70144	0.18937
C	0.92783	-3.06582	1.29854	C	6.35414	2.31967	-1.97086
C	2.99405	-2.39712	0.21478	H	4.24758	2.36993	-2.41153

C	6.99448	1.03257	-0.03271
H	5.38885	0.07020	1.03222
C	7.34785	1.84231	-1.11446
H	6.61743	2.95572	-2.81210
H	7.76141	0.65950	0.64131
H	8.38900	2.10265	-1.28585

Pro-*R* L18

Pd	0.56338	0.80993	0.23483
C	1.74158	2.52756	-0.17175
C	2.12982	3.18906	-1.35161
C	2.73918	2.35875	0.88142
C	4.10830	2.74545	0.65737
C	-0.34106	2.75110	0.07998
C	-1.27584	2.60912	-1.02492
C	-0.59842	3.72554	1.05392
C	-2.43868	3.45129	-1.10330
C	-1.76292	4.53859	0.93675
C	-2.05490	1.49146	-3.07241
C	-3.36740	3.29335	-2.17001
C	-3.18718	2.33705	-3.13991
H	-1.90807	0.72277	-3.82718
H	-4.23684	3.94658	-2.19271
H	-3.90738	2.22437	-3.94584
C	-1.13821	1.62706	-2.05211
H	-0.28525	0.95432	-2.01344
C	-2.65403	4.42147	-0.09726
H	-3.52843	5.06512	-0.15491
C	0.27151	4.03047	2.25371
H	-0.09742	4.92512	2.76486
H	0.26932	3.21689	2.98690
H	1.31199	4.21024	1.97460
H	-1.93619	5.28794	1.70553
C	5.09314	2.52624	1.66102
C	4.77140	1.95135	2.86622
C	3.42921	1.58466	3.11713
C	2.45681	1.78399	2.16143
C	4.45495	3.35293	-0.56915
H	5.48514	3.65538	-0.74202
C	3.49060	3.57700	-1.51637
H	3.76486	4.07668	-2.44242
H	6.11520	2.83299	1.44933
H	5.53346	1.79187	3.62441
H	3.15651	1.15008	4.07528
H	1.43336	1.51010	2.38897
C	1.22507	3.60350	-2.49371
H	0.77183	2.75977	-3.01906
H	1.79827	4.17929	-3.22715
H	0.40049	4.23288	-2.14701
P	-0.49930	-1.27941	0.82156
C	-0.54490	-2.66855	-0.38226
C	0.02271	-2.01844	2.51466
C	-2.39858	-1.37774	0.94399
C	0.45774	-3.23035	-1.19814
C	-1.85707	-3.16708	-0.47341
C	-0.56250	-3.42156	2.75220
C	1.55978	-2.08989	2.56244
C	-0.46024	-1.04486	3.60976
O	-2.81487	-2.61147	0.31996
H	-2.68525	-1.46094	1.99558
C	-3.15171	-0.19865	0.31022
C	0.10861	-4.27812	-2.06319
C	1.89154	-2.79887	-1.17263
C	-2.20075	-4.21187	-1.33066
H	-0.19001	-4.13571	2.01139
H	-0.26193	-3.77982	3.74629
H	-1.65705	-3.43295	2.71489
H	1.94519	-2.81022	1.83906
H	2.02432	-1.11761	2.36729
H	1.87528	-2.41865	3.56243
H	-0.09743	-0.02598	3.43466
H	-1.55180	-1.00527	3.69227
H	-0.07485	-1.37634	4.58276
C	-1.19943	-4.76206	-2.12620
H	0.87956	-4.71739	-2.68863
C	2.35197	-1.72355	-1.95679
C	2.84037	-3.54941	-0.45100
H	-3.22583	-4.56621	-1.36393
H	-1.44075	-5.57385	-2.80735
C	3.70981	-1.37119	-1.97031
O	1.40510	-1.08600	-2.70556
C	4.19897	-3.21012	-0.46157
C	4.61355	-2.11603	-1.21785
H	4.06097	-0.52943	-2.55345
C	1.84310	-0.12037	-3.65254
H	4.92293	-3.78029	0.10736
H	5.66465	-1.84086	-1.22494
H	0.94798	0.19255	-4.19283
H	2.29304	0.75039	-3.16162
H	2.56111	-0.55591	-4.35959
H	-2.74175	0.72579	0.73081
O	2.33396	-4.60963	0.25301
C	3.23765	-5.46068	0.94001
H	3.76604	-4.92876	1.74207

H	2.62583	-6.25245	1.37617
H	3.97235	-5.90362	0.25527
C	-4.64624	-0.26010	0.55122
C	-5.21767	0.41139	1.64114
C	-5.48855	-1.00024	-0.29022
C	-6.58993	0.34382	1.88874
H	-4.58121	1.00264	2.29673
C	-6.86095	-1.06933	-0.04763
H	-5.06222	-1.52601	-1.13997
C	-7.41699	-0.39831	1.04388
H	-7.01281	0.87654	2.73686
H	-7.49774	-1.64637	-0.71359
H	-8.48641	-0.44962	1.23119
H	-2.93194	-0.17377	-0.76213

Pro-*S* L19

Pd	-1.17645	0.10908	-0.39621
C	-2.44446	1.69367	0.23503
C	-2.86699	2.13624	1.50169
C	-2.09322	2.69994	-0.76405
C	-2.13977	4.10112	-0.43489
C	-3.30166	-0.15439	-0.16948
C	-3.37976	-1.23924	0.80098
C	-4.36765	0.01589	-1.06632
C	-4.52412	-2.11146	0.82263
C	-5.48436	-0.86648	-1.00014
C	-2.41627	-2.61214	2.60120
C	-4.58230	-3.19814	1.73996
C	-3.55324	-3.45308	2.61374
H	-1.59209	-2.80688	3.28257
H	-5.46476	-3.83415	1.72330
H	-3.60770	-4.29105	3.30366
C	-2.33849	-1.54560	1.73149
H	-1.44764	-0.92219	1.74714
C	-5.57652	-1.89007	-0.09493
H	-6.44799	-2.54004	-0.07503
C	-4.48118	1.09647	-2.11981
H	-5.44616	1.01880	-2.63015
H	-3.70322	1.02344	-2.88509
H	-4.41172	2.09937	-1.69014
H	-6.29836	-0.70431	-1.70263
C	-1.74422	5.07956	-1.38991
C	-1.30442	4.72208	-2.64102
C	-1.26021	3.35243	-2.98991
C	-1.64506	2.38583	-2.08690
C	-2.57864	4.48737	0.85086
H	-2.63193	5.54455	1.10012
C	-2.92841	3.53243	1.76966
H	-3.26705	3.84442	2.75466
H	-1.79633	6.12676	-1.09978
H	-1.00250	5.47994	-3.35902
H	-0.93038	3.05688	-3.98253
H	-1.61631	1.34432	-2.38935
C	-3.31330	1.25723	2.64942
H	-2.52484	0.58632	2.99916
H	-3.61675	1.87944	3.49717
H	-4.16507	0.62792	2.37556
P	0.15837	-1.69378	-1.21134
C	1.41529	-2.58295	-0.19550
C	0.88144	-1.53787	-2.98080
C	-0.80027	-3.31026	-1.26488
C	2.48651	-2.10635	0.58101
C	1.16119	-3.96754	-0.23633
C	1.79680	-2.71446	-3.35882
C	-0.31301	-1.46077	-3.95422
C	1.66925	-0.21613	-3.06195
O	0.09464	-4.39835	-0.96502
C	3.28296	-3.03953	1.26634
C	2.80444	-0.64923	0.72826
C	1.96349	-4.89367	0.42997
H	1.28027	-3.67800	-3.29194
H	2.14395	-2.59142	-4.39376
H	2.67905	-2.75158	-2.71277
H	-0.86192	-2.40597	-4.02395
H	-1.02309	-0.67637	-3.66838
H	0.05741	-1.22650	-4.96059
H	1.05637	0.64001	-2.75707
H	2.56863	-0.24353	-2.44300
H	1.98904	-0.04871	-4.09964
C	3.03108	-4.41174	1.18185
H	4.10197	-2.67953	1.88225
C	3.79950	-0.05061	-0.06352
C	2.13677	0.13507	1.68703
H	1.73344	-5.95204	0.36533
H	3.66469	-5.10927	1.72330
C	4.11716	1.31269	0.06301
C	2.43813	1.49749	1.85632
C	3.42572	2.04588	1.03181
C	1.54049	-1.01084	3.68176
H	3.65610	3.10289	1.13937
H	-1.25953	-3.51321	-2.23313
C	5.13705	1.98868	-0.84645
C	1.71039	2.35961	2.88076
C	5.66339	-1.39062	-0.72039

O	4.40102	-0.81471	-1.05394
O	1.12764	-0.44789	2.43671
H	2.24011	-1.84109	3.52969
H	2.00752	-0.25596	4.32765
H	0.63464	-1.38563	4.16438
H	5.55868	-2.14370	0.06879
H	6.03052	-1.87164	-1.63033
H	6.38291	-0.62683	-0.39913
C	4.48762	3.10240	-1.69154
H	4.10597	3.91535	-1.06274
H	5.22037	3.53346	-2.38454
H	3.64913	2.71333	-2.27895
H	5.50295	1.23052	-1.54540
H	0.93156	1.73687	3.33124
C	2.65799	2.82766	4.00343
H	3.45148	3.47475	3.60978
H	2.10629	3.40032	4.75866
H	3.14129	1.98121	4.50519
C	1.00684	3.56111	2.22215
H	0.31165	3.23587	1.44265
H	0.43580	4.12387	2.96995
H	1.72761	4.25190	1.76793
C	6.34396	2.53180	-0.05702
H	7.08455	2.96904	-0.73749
H	6.03989	3.31382	0.64913
H	6.83732	1.74058	0.51931
H	-1.58094	-3.27669	-0.49733

Pro-*R* L19

Pd	1.21406	-0.09390	-0.28340
C	3.09950	-0.98380	0.21965
C	4.27116	-1.18010	-0.53017
C	2.72037	-2.01275	1.17926
C	3.51746	-3.19957	1.33302
C	2.81293	1.02412	0.58191
C	2.88837	2.09227	-0.41420
C	3.18987	1.33102	1.90365
C	3.24327	3.43266	-0.02491
C	3.56048	2.66512	2.23455
C	2.59503	2.94716	-2.70956
C	3.25337	4.48471	-0.98360
C	2.93016	4.25863	-2.29981
H	2.35760	2.74901	-3.75161
H	3.52750	5.48152	-0.64469
H	2.94346	5.07160	-3.02100
C	2.57973	1.91125	-1.80026
H	2.33542	0.91411	-2.14891
C	3.57650	3.68824	1.32347
H	3.85420	4.69619	1.62277
C	3.29823	0.35497	3.05579
H	3.71492	0.86242	3.93142
H	2.33665	-0.07081	3.35282
H	3.95292	-0.48740	2.81510
H	3.84181	2.87044	3.26464
C	3.11930	-4.21727	2.24516
C	1.97124	-4.10168	2.99133
C	1.17036	-2.94448	2.84944
C	1.53736	-1.94343	1.97613
C	4.68796	-3.34638	0.55374
H	5.30087	-4.23671	0.67155
C	5.03575	-2.36619	-0.33842
H	5.94081	-2.48460	-0.92968
H	3.74854	-5.10048	2.33215
H	1.67871	-4.88829	3.68184
H	0.26038	-2.84006	3.43464
H	0.90451	-1.06353	1.88242
C	4.84812	-0.21232	-1.54061
H	4.21143	-0.09316	-2.42294
H	5.81954	-0.57444	-1.89149
H	4.99710	0.78465	-1.11797
P	-0.37854	-1.22367	-1.68844
C	-1.76094	-0.23552	-2.39621
C	-1.07977	-2.97298	-1.35435
C	0.29529	-1.37643	-3.44291
C	-2.74395	0.54815	-1.76571
C	-1.71421	-0.28238	-3.80189
C	-2.02360	-3.45498	-2.46956
C	0.13033	-3.92075	-1.22899
C	-1.84041	-2.94431	-0.01562
O	-0.73814	-1.03638	-4.38345
H	1.12531	-0.67235	-3.57314
C	-3.64337	1.26412	-2.57484
C	-2.86562	0.67688	-0.27761
C	-2.62676	0.40407	-4.60196
H	-1.53322	-3.49095	-3.44816
H	-2.37255	-4.47001	-2.23637
H	-2.90489	-2.81127	-2.55363
H	0.69092	-4.01011	-2.16694
H	0.82306	-3.59555	-0.44706
H	-0.22523	-4.92603	-0.96792
H	-2.14854	-3.96605	0.24470
H	-1.21130	-2.56913	0.79803
H	-2.74515	-2.33370	-0.07718
C	-3.59080	1.18435	-3.96860

H	-4.37854	1.90724	-2.10098
C	-3.92951	0.06049	0.40834
C	-1.93990	1.43978	0.45774
H	-2.55738	0.33603	-5.68261
H	-4.30010	1.75054	-4.56643
C	-4.08018	0.19717	1.79991
C	-2.07944	1.63326	1.84243
C	-3.15821	1.00505	2.47229
C	-6.03866	-0.31393	-0.67108
H	-3.26854	1.12731	3.54696
H	0.64943	-2.38182	-3.67991
C	-5.16230	-0.55014	2.57236
H	-5.96151	0.45161	-1.45110
C	-1.09466	2.48493	2.63482
C	-0.97201	3.23984	-0.78338
H	-0.01138	3.47149	-1.24550
O	-0.82855	1.94259	-0.19586
O	-4.75154	-0.80287	-0.29908
H	-1.20441	3.99263	-0.01952
H	-6.58178	0.09996	0.18732
H	-6.58927	-1.17181	-1.06510
H	-1.75988	3.23832	-1.54563
H	-5.71120	-1.16658	1.85459
H	-0.27365	2.74427	1.96012
C	-4.55067	-1.51440	3.60816
H	-5.33945	-2.09062	4.10684
H	-3.86288	-2.22024	3.13026
H	-3.99332	-0.97479	4.38289
C	-6.16332	0.41082	3.24261
H	-6.63387	1.07762	2.51084
H	-6.95653	-0.15063	3.75078
H	-5.66990	1.04066	3.99262
C	-1.74963	3.79456	3.11886
H	-2.57386	3.59267	3.81427
H	-1.01700	4.42138	3.64132
H	-2.15729	4.37432	2.28287
C	-0.48076	1.71649	3.81972
H	0.28348	2.32852	4.31263
H	-1.23417	1.45765	4.57362
H	-0.00571	0.78873	3.48502

Pro-*S* L20

Pd	-1.50020	0.03334	-0.54757
C	-2.47504	1.92760	-0.53335
C	-2.97178	2.75973	0.48638
C	-1.77541	2.55281	-1.65304
C	-1.54253	3.97379	-1.66446
C	-3.63821	0.20105	-0.62685
C	-4.05989	-0.54373	0.55158
C	-4.52355	0.29492	-1.71083
C	-5.35223	-1.17494	0.58553
C	-5.79753	-0.33725	-1.62783
C	-3.63583	-1.49572	2.78052
C	-5.74769	-1.93887	1.71948
C	-4.91309	-2.10336	2.79846
H	-2.96473	-1.62188	3.62614
H	-6.73207	-2.40181	1.70595
H	-5.22601	-2.69466	3.65489
C	-3.23107	-0.74315	1.69907
H	-2.24119	-0.29243	1.71192
C	-6.21149	-1.04401	-0.52968
H	-7.19363	-1.50990	-0.50450
C	-4.27106	1.04740	-2.99912
H	-5.16309	1.01315	-3.63224
H	-3.44595	0.62353	-3.57943
H	-4.02845	2.09830	-2.82117
H	-6.46563	-0.24139	-2.48042
C	-0.80516	4.57544	-2.72247
C	-0.29764	3.82768	-3.75671
C	-0.53012	2.43323	-3.77642
C	-1.24453	1.82655	-2.76667
C	-2.05964	4.75993	-0.61171
H	-1.90332	5.83592	-0.62412
C	-2.75473	4.16473	0.40849
H	-3.16343	4.78490	1.20289
H	-0.65083	5.65173	-2.68977
H	0.26659	4.30001	-4.55634
H	-0.15199	1.83289	-4.59983
H	-1.42163	0.75783	-2.81968
C	-3.77380	2.31721	1.69132
H	-3.21475	1.64927	2.35156
H	-4.06888	3.19060	2.28130
H	-4.68537	1.78588	1.40371
P	-0.50093	-2.13068	-0.69682
C	0.53375	-2.90168	0.61942
C	0.29020	-2.62330	-2.37145
C	-1.75579	-3.49567	-0.38512
C	1.66859	-2.43278	1.30410
C	0.00835	-4.16571	0.95151
C	0.92212	-4.02536	-2.34397
C	-0.82375	-2.56466	-3.43660
C	1.36482	-1.57510	-2.71641
O	-1.10595	-4.58728	0.29226
C	2.24906	-3.25701	2.28305

C	2.29416	-1.09159	1.06585
C	0.59473	-4.98946	1.91205
H	0.19691	-4.80139	-2.07675
H	1.32004	-4.26768	-3.33877
H	1.75180	-4.07196	-1.63210
H	-1.58017	-3.34464	-3.29750
H	-1.33486	-1.59545	-3.43976
H	-0.38162	-2.71511	-4.43002
H	0.95953	-0.55691	-2.70713
H	2.20645	-1.62362	-2.02169
H	1.75547	-1.77342	-3.72403
C	1.72465	-4.51885	2.57462
H	3.11611	-2.89493	2.82765
C	3.45757	-0.98332	0.28480
C	1.79380	0.05068	1.71558
H	0.15714	-5.95770	2.13170
H	2.19471	-5.13341	3.33782
C	4.13017	0.24643	0.14079
C	2.45235	1.29157	1.61423
C	3.60886	1.35082	0.82658
C	0.81901	-0.18202	3.86602
H	4.09434	2.31379	0.69930
H	-2.20361	-3.88825	-1.29932
C	5.06245	-2.74510	0.12890
O	3.88748	-2.11276	-0.38547
O	0.63385	-0.07430	2.45183
H	1.36142	-1.10187	4.11728
H	1.35557	0.68619	4.26518
H	-0.18148	-0.22045	4.30228
H	4.86568	-3.18087	1.11562
H	5.31071	-3.54248	-0.57504
H	5.90137	-2.04421	0.19318
H	-2.54737	-3.10332	0.26171
C	1.97368	2.52026	2.30589
C	2.87950	3.30068	3.04494
C	0.63940	2.95013	2.21233
C	2.46706	4.47530	3.67471
H	3.91206	2.97311	3.13587
C	0.23033	4.12689	2.83928
H	-0.07286	2.37097	1.63439
C	1.13887	4.89243	3.57392
H	3.18301	5.06097	4.24571
H	-0.80173	4.45006	2.73865
H	0.81462	5.80793	4.06184
C	5.33806	0.41914	-0.71362
C	6.44550	1.13591	-0.22831
C	5.39238	-0.08044	-2.02678
C	7.56952	1.34983	-1.02667
H	6.42708	1.51672	0.78944
C	6.51585	0.13489	-2.82415
H	4.54686	-0.63222	-2.42241
C	7.60909	0.84990	-2.32916
H	8.41548	1.90365	-0.62788
H	6.53425	-0.25258	-3.83957
H	8.48340	1.01515	-2.95303

Pro-*R* L20

Pd	1.57347	-0.35502	-0.00867
C	3.30735	-0.80107	1.15959
C	4.51321	-1.43676	0.82024
C	2.74485	-1.05359	2.47995
C	3.41139	-1.93423	3.40085
C	3.19383	1.03202	0.25553
C	3.49716	1.30292	-1.15107
C	3.49303	2.04021	1.19416
C	4.02808	2.57951	-1.55492
C	4.05860	3.26933	0.75026
C	3.47907	0.67823	-3.53843
C	4.25863	2.86687	-2.92971
C	3.98362	1.94546	-3.91061
H	3.27779	-0.07037	-4.30034
H	4.65851	3.84554	-3.18602
H	4.16190	2.17940	-4.95680
C	3.25378	0.37450	-2.21309
H	2.88554	-0.61409	-1.96243
C	4.31290	3.54702	-0.56703
H	4.73396	4.50479	-0.86337
C	3.31064	1.95601	2.69461
H	3.68144	2.87253	3.16374
H	2.26518	1.83947	2.99081
H	3.85725	1.11470	3.12949
H	4.29534	4.01744	1.50314
C	2.83706	-2.20598	4.67476
C	1.63749	-1.65273	5.05284
C	0.96061	-0.79367	4.15576
C	1.49973	-0.50963	2.91984
C	4.62908	-2.53929	3.01372
H	5.14344	-3.19814	3.70906
C	5.14302	-2.29312	1.76737
H	6.08010	-2.76301	1.47806
H	3.37225	-2.87597	5.34426
H	1.21006	-1.87215	6.02763
H	0.01079	-0.34972	4.44206
H	0.96073	0.15494	2.24834

C	5.25917	-1.27708	-0.48635
H	4.68878	-1.64091	-1.34613
H	6.19440	-1.84468	-0.45439
H	5.51056	-0.23279	-0.69169
P	0.12660	-2.14175	-0.71626
C	-1.03363	-1.86089	-2.11682
C	-0.73970	-3.35778	0.48207
C	1.09019	-3.29588	-1.85666
C	-2.07163	-0.92659	-2.27670
C	-0.73101	-2.73732	-3.17555
C	-1.56088	-4.42792	-0.25685
C	0.36666	-4.01586	1.33100
C	-1.66575	-2.54843	1.40896
O	0.27944	-3.63440	-2.99398
H	1.98940	-2.78244	-2.21552
C	-2.76097	-0.89682	-3.50122
C	-2.49792	0.04207	-1.21584
C	-1.43115	-2.72280	-4.38108
H	-0.95351	-5.01592	-0.95322
H	-1.99344	-5.12410	0.47441
H	-2.38646	-3.97929	-0.81832
H	1.02785	-4.65632	0.73592
H	0.98190	-3.27046	1.84385
H	-0.09791	-4.65234	2.09526
H	-2.10062	-3.22165	2.16019
H	-1.12042	-1.76319	1.94208
H	-2.49135	-2.09394	0.85555
C	-2.44863	-1.78445	-4.53322
H	-3.54381	-0.15829	-3.64487
C	-3.72157	-0.15340	-0.54896
C	-1.74230	1.19614	-0.94260
H	-1.16459	-3.41927	-5.16920
H	-2.99860	-1.73583	-5.46926
C	-4.21106	0.79825	0.36750
C	-2.22362	2.19382	-0.07141

Pro-*S* L21

Pd	-0.89541	0.02706	-0.32340
C	-2.51287	1.18480	0.40561
C	-3.07496	1.34441	1.68481
C	-2.34325	2.37215	-0.42700
C	-2.68496	3.67438	0.08384
C	-2.90549	-0.74547	-0.28858
C	-2.76552	-1.94307	0.52914
C	-3.94484	-0.69656	-1.22921
C	-3.66663	-3.05055	0.35172
C	-4.82033	-1.81302	-1.36124
C	-1.58865	-3.29057	2.21819
C	-3.50370	-4.23907	1.11747
C	-2.48685	-4.36734	2.03245
H	-0.77859	-3.38010	2.93755
H	-4.20472	-5.05427	0.95131
H	-2.37047	-5.28317	2.60592
C	-1.72838	-2.12444	1.49617
H	-1.01967	-1.31668	1.66217
C	-4.70064	-2.95010	-0.60730
H	-5.39062	-3.78038	-0.73740
C	-4.26859	0.46988	-2.13751
H	-5.18319	0.26069	-2.70098
H	-3.47771	0.66784	-2.86714
H	-4.42599	1.39591	-1.57862
H	-5.62042	-1.74382	-2.09439
C	-2.46524	4.83806	-0.70552
C	-1.92262	4.75683	-1.96480
C	-1.59142	3.48715	-2.49182
C	-1.80005	2.34425	-1.75128
C	-3.23864	3.77698	1.37906
H	-3.51304	4.75600	1.76500
C	-3.42846	2.64850	2.13362
H	-3.86517	2.74145	3.12508
H	-2.73936	5.80200	-0.28206
H	-1.75859	5.65422	-2.55548
H	-1.17830	3.40782	-3.49402
H	-1.55197	1.38313	-2.18865
C	-3.38591	0.23571	2.66655
H	-2.49049	-0.29199	3.00469
H	-3.87738	0.65106	3.55195
H	-4.05274	-0.51654	2.23602
P	0.93064	-1.24160	-1.18908
C	2.39005	-1.70825	-0.16562
C	1.60561	-0.75833	-2.91578
C	0.58483	-3.08099	-1.35955
C	3.20426	-0.94082	0.68589
C	2.64011	-3.08694	-0.29952
C	2.80069	-1.61902	-3.35977
C	0.43792	-0.92145	-3.91060
C	2.02232	0.72396	-2.87324
O	1.79685	-3.81235	-1.08556
C	4.27524	-1.57539	1.33690
C	2.94679	0.51013	0.95224
C	3.71272	-3.71295	0.33541
H	2.55431	-2.68523	-3.39865
H	3.11859	-1.31215	-4.36541
H	3.65465	-1.49089	-2.68741

H	0.13427	-1.96672	-4.03603
H	-0.44322	-0.34922	-3.59972
H	0.74893	-0.55283	-4.89674
H	1.20923	1.36516	-2.51399
H	2.89819	0.88005	-2.23873
H	2.28681	1.05413	-3.88729
C	4.53168	-2.93697	1.15176
H	4.90059	-0.99590	2.00974
C	3.69403	1.50829	0.30298
C	1.96024	0.90319	1.87375
H	3.87586	-4.77691	0.19874
H	5.36739	-3.40137	1.66860
C	3.44928	2.87203	0.52805
C	1.69190	2.25697	2.13009
C	2.44616	3.21068	1.44167
C	1.73788	-0.54893	3.73677
H	2.23233	4.26388	1.61215
H	0.24035	-3.36806	-2.35425
C	4.20944	3.94112	-0.22047
C	0.59371	2.66260	3.08295
C	5.98177	1.12795	-0.25974
O	4.61341	1.12232	-0.66010
O	1.19477	-0.05989	2.51034
H	2.67268	-1.09607	3.56678
H	1.92069	0.26703	4.44828
H	0.99148	-1.22896	4.15428
H	6.29546	2.11353	0.10553
H	6.16885	0.37863	0.51866
H	6.56242	0.87357	-1.14978
H	4.44098	3.61728	-1.23969
H	-0.37332	2.25833	2.76545
H	-0.17159	-3.36645	-0.62036
H	3.62404	4.86440	-0.27472
H	5.16174	4.18988	0.26755
H	0.50800	3.75256	3.12867
H	0.77608	2.29927	4.10159

Pro-*R* L21

Pd	0.89853	0.06925	-0.22731
C	2.80176	-0.90227	-0.41491
C	3.81383	-0.80269	-1.38573
C	2.54847	-2.21212	0.17491
C	3.31608	-3.35878	-0.23019
C	2.68761	0.82065	0.67043
C	2.66763	2.17270	0.11101
C	3.29181	0.64360	1.93167
C	3.19872	3.28399	0.85752
C	3.82703	1.76954	2.61874
C	2.01727	3.79245	-1.63328
C	3.12276	4.60705	0.33818
C	2.53905	4.86793	-0.87779
H	1.56766	3.98068	-2.60487
H	3.53965	5.41449	0.93637
H	2.48630	5.88365	-1.26081
C	2.08661	2.50130	-1.15554
H	1.69098	1.69780	-1.76734
C	3.78428	3.04450	2.11990
H	4.19741	3.87751	2.68380
C	3.48499	-0.66982	2.65943
H	4.05117	-0.50111	3.58068
H	2.54256	-1.14803	2.93780
H	4.03835	-1.39514	2.05630
H	4.28916	1.59697	3.58769
C	3.03963	-4.64021	0.32482
C	2.03416	-4.82171	1.24343
C	1.25991	-3.70865	1.64575
C	1.51356	-2.45660	1.12864
C	4.33148	-3.19815	-1.20045
H	4.92262	-4.05880	-1.50389
C	4.55435	-1.96272	-1.74978
H	5.33721	-1.84829	-2.49592
H	3.64384	-5.48185	-0.00711
H	1.83202	-5.80692	1.65533
H	0.46005	-3.83787	2.37033
H	0.90224	-1.61794	1.45442
C	4.23914	0.46529	-2.09375
H	3.44609	0.88467	-2.71991
H	5.09202	0.25879	-2.74779
H	4.53941	1.24912	-1.39298
P	-0.98710	-0.50178	-1.60732
C	-2.46350	0.59385	-1.53648
C	-1.64908	-2.27978	-1.85238
C	-0.73902	0.09563	-3.37762
C	-3.24775	1.00232	-0.44305
C	-2.75282	1.09428	-2.81918
C	-2.81112	-2.34902	-2.85811
C	-0.45910	-3.12679	-2.34744
C	-2.11979	-2.81379	-0.48738
O	-1.95645	0.69734	-3.85287
H	0.05099	0.85570	-3.39544
C	-4.30408	1.89865	-0.68013
C	-2.99357	0.54656	0.96064
C	-3.81718	1.96315	-3.05643
H	-2.52888	-1.98677	-3.85221

H	-3.13563	-3.39274	-2.96592
H	-3.67239	-1.76659	-2.51580
H	-0.10379	-2.80973	-3.33520
H	0.38510	-3.08674	-1.65208
H	-0.77396	-4.17466	-2.43732
H	-2.40373	-3.86968	-0.59259
H	-1.32476	-2.75733	0.26276
H	-2.99618	-2.27227	-0.12047
C	-4.58877	2.36131	-1.96712
H	-4.89370	2.25150	0.16036
C	-3.88508	-0.33573	1.60133
C	-1.87817	1.00803	1.68059
H	-4.00994	2.32171	-4.06219
H	-5.41194	3.05453	-2.11858
C	-3.67445	-0.75711	2.92465
C	-1.65124	0.63301	3.01382
C	-2.56258	-0.24735	3.60249
C	-6.22497	-0.42855	1.09354
H	-2.38382	-0.57292	4.62563
H	-0.46764	-0.70667	-4.06699
H	-6.33491	0.61937	0.79094
C	-1.14343	3.20075	1.07240
H	-0.29160	3.64690	0.55697
O	-0.93200	1.78588	1.03764
O	-4.90179	-0.89808	0.84855
H	-1.18165	3.56761	2.10570
H	-6.50972	-0.53049	2.14727
H	-6.88599	-1.04809	0.48267
H	-2.07246	3.46854	0.55529
C	-4.57860	-1.77220	3.58345
H	-5.45439	-1.30590	4.05480
H	-4.94790	-2.49963	2.85408
H	-4.04176	-2.31360	4.36902
C	-0.44778	1.14128	3.77112
H	0.46704	1.05196	3.17696
H	-0.55193	2.20171	4.03546
H	-0.31570	0.58400	4.70395

Pro-*S* 22

Pd	1.47300	0.20924	-0.28692
C	2.89186	-1.30616	-0.76434
C	3.83571	-2.03192	-0.01398
C	2.11290	-2.02644	-1.76915
C	2.24845	-3.45321	-1.91221
C	3.47943	0.69544	-0.87314
C	3.98481	1.43271	0.27612
C	4.03992	0.95805	-2.13219
C	5.01490	2.42243	0.10949
C	5.06331	1.94228	-2.24929
C	3.95520	1.99711	2.67248
C	5.48391	3.16488	1.22959
C	4.97104	2.96428	2.48839
H	3.54343	1.82536	3.66374
H	6.26244	3.90598	1.06171
H	5.33840	3.53956	3.33397
C	3.48486	1.26468	1.60354
H	2.70198	0.52805	1.76857
C	5.54234	2.65464	-1.18171
H	6.32725	3.39553	-1.31288
C	3.67805	0.26332	-3.42702
H	4.34890	0.59225	-4.22672
H	2.65650	0.48318	-3.75267
H	3.76108	-0.82349	-3.34806
H	5.48047	2.12199	-3.23730
C	1.44958	-4.16290	-2.85205
C	0.53508	-3.51544	-3.64704
C	0.39609	-2.11315	-3.53499
C	1.15787	-1.40256	-2.63355
C	3.18906	-4.13493	-1.10906
H	3.30424	-5.21058	-1.21961
C	3.95455	-3.43707	-0.21245
H	4.69030	-3.97165	0.38351
H	1.58602	-5.23953	-2.92851
H	-0.06619	-4.06980	-4.36289
H	-0.31243	-1.58664	-4.16885
H	1.03870	-0.32599	-2.58270
C	4.80649	-1.46420	1.00000
H	4.31222	-1.05367	1.88480
H	5.48609	-2.25119	1.34140
H	5.41373	-0.65894	0.57817
P	-0.15309	1.92601	0.13768
C	-1.59332	1.74304	1.26954
C	-0.80353	2.89516	-1.38094
C	0.46215	3.28970	1.27607
C	-2.59370	0.75697	1.34636
C	-1.62263	2.82158	2.17368
C	-1.73503	4.05852	-0.99832
C	0.43506	3.43149	-2.12659
C	-1.56174	1.91370	-2.29354
O	-0.63056	3.75221	2.09065
C	-3.61089	0.90541	2.30235
C	-2.62587	-0.45303	0.46868
C	-2.63404	2.96897	3.12189
H	-1.24245	4.79502	-0.35529

H	-2.06028	4.57882	-1.90936
H	-2.63109	3.70227	-0.48044
H	0.99230	4.16653	-1.53437
H	1.12732	2.62658	-2.39513
H	0.11555	3.93189	-3.05016
H	-0.95416	1.03779	-2.54572
H	-2.49093	1.56749	-1.83298
H	-1.82608	2.41994	-3.23190
C	-3.63175	1.99815	3.17096
H	-4.38905	0.15093	2.36543
C	-3.61904	-0.60894	-0.51460
C	-1.73105	-1.52338	0.65078
H	-2.62148	3.81799	3.79738
H	-4.42919	2.08603	3.90386
C	-3.71289	-1.76189	-1.29680
C	-1.81267	-2.68861	-0.11526
C	-2.80614	-2.79877	-1.08505
H	-2.86177	-3.69550	-1.69517
H	0.86440	4.14754	0.73316
O	-4.44423	0.47945	-0.73462
O	-0.73594	-1.35939	1.59363
H	1.24544	2.89072	1.92833
H	-4.48028	-1.83449	-2.05966
H	-1.09567	-3.48608	0.04384
C	-5.80202	0.31983	-0.93291
C	-6.42359	1.27005	-1.74719
C	-6.55248	-0.67879	-0.30452
C	-7.80419	1.21827	-1.93428
H	-5.81539	2.03713	-2.21593
C	-7.93317	-0.72201	-0.50693
H	-6.06360	-1.41034	0.32996
C	-8.56559	0.22124	-1.31916
H	-8.28450	1.95931	-2.56752
H	-8.51604	-1.49832	-0.01830
H	-9.64035	0.18088	-1.47015
C	-0.41888	-2.42518	2.42672
C	-1.40475	-3.06204	3.18553
C	0.92215	-2.78978	2.54017
C	-1.03486	-4.07745	4.06747
H	-2.44152	-2.75562	3.08805
C	1.27905	-3.80311	3.43235
H	1.66147	-2.28938	1.92353
C	0.30583	-4.45047	4.19611
H	-1.79822	-4.57278	4.66165
H	2.32310	-4.09102	3.52028
H	0.58840	-5.24017	4.88648

Pro-*R* L22

Pd	-1.48956	-0.45090	0.34973
C	-2.71109	-2.08070	-0.36321
C	-3.75899	-2.80652	0.22226
C	-1.80234	-2.77959	-1.26175
C	-1.97915	-4.18274	-1.52675
C	-3.31603	-0.09648	-0.65019
C	-3.99542	0.72722	0.34713
C	-3.62572	0.10968	-2.00628
C	-4.91024	1.76043	-0.06479
C	-4.55746	1.12487	-2.36249
C	-4.39053	1.44359	2.67219
C	-5.52979	2.60260	0.90172
C	-5.27512	2.46046	2.24357
H	-4.19755	1.30722	3.73322
H	-6.21425	3.37007	0.54669
H	-5.75286	3.11242	2.96986
C	-3.78183	0.61168	1.75828
H	-3.12379	-0.17198	2.11684
C	-5.17645	1.92977	-1.44205
H	-5.87743	2.69875	-1.75841
C	-3.08000	-0.67903	-3.17643
H	-3.53198	-0.32230	-4.10709
H	-1.99616	-0.58831	-3.28177
H	-3.29949	-1.74671	-3.08611
H	-4.77770	1.26171	-3.41832
C	-1.07326	-4.87282	-2.38013
C	-0.00630	-4.23097	-2.96073
C	0.19109	-2.85426	-2.70466
C	-0.67736	-2.16211	-1.88882
C	-3.05297	-4.86790	-0.91408
H	-3.19662	-5.92638	-1.11732
C	-3.89752	-4.19389	-0.07289
H	-4.72166	-4.72929	0.39221
H	-1.24358	-5.93293	-2.55537
H	0.68015	-4.77202	-3.60668
H	1.03364	-2.33178	-3.14913
H	-0.49731	-1.10468	-1.71005
C	-4.81999	-2.25407	1.14927
H	-4.40693	-1.90169	2.09885
H	-5.55108	-3.03363	1.38463
H	-5.36047	-1.41483	0.70416
P	0.33631	-0.50016	1.91328
C	1.63647	0.79589	2.06440
C	1.21250	-2.16589	2.26118
C	-0.25495	0.03377	3.61663
C	2.50915	1.35095	1.11142

C	1.70075	1.24682	3.39641
C	2.19742	-2.09440	3.44097
C	0.10052	-3.19173	2.55903
C	1.96558	-2.58704	0.98659
O	0.81117	0.72484	4.28997
H	-1.10014	0.72318	3.49968
C	3.45577	2.29578	1.53733
C	2.45394	1.01847	-0.34547
C	2.64174	2.18347	3.81982
H	1.71188	-1.79863	4.37654
H	2.64511	-3.08506	3.59747
H	3.01180	-1.38970	3.24517
H	-0.44093	-2.96144	3.48458
H	-0.62757	-3.24746	1.74342
H	0.54948	-4.18558	2.68461
H	2.40437	-3.58249	1.13996
H	1.30026	-2.64625	0.11993
H	2.78290	-1.89803	0.75491
C	3.52562	2.69637	2.87276
H	4.13372	2.72783	0.80768
C	3.46930	0.26891	-0.96550
C	1.43836	1.53727	-1.16974
H	2.66133	2.49925	4.85768
H	4.26568	3.43264	3.17436
C	3.46857	0.01986	-2.34047
C	1.42235	1.30449	-2.54852
C	2.44210	0.54759	-3.12249
H	2.43275	0.35962	-4.19255
H	-0.56365	-0.80298	4.24696
O	0.43778	2.24981	-0.54735
O	4.42316	-0.27002	-0.12518
C	-0.08328	3.38901	-1.14529
C	0.73829	4.35424	-1.73488
C	-1.46293	3.57695	-1.05916
C	0.16103	5.51534	-2.25054
H	1.81120	4.19841	-1.78250
C	-2.02447	4.74698	-1.57220
H	-2.07637	2.81202	-0.59552
C	-1.21898	5.71759	-2.17191
H	0.79764	6.26809	-2.70809
H	-3.09924	4.89020	-1.50581
H	-1.66144	6.62492	-2.57323
C	5.73608	-0.42144	-0.53378
C	6.38886	-1.58658	-0.12689
C	6.42119	0.57114	-1.24118
C	7.73895	-1.75961	-0.43234
H	5.83129	-2.33534	0.42660
C	7.76952	0.38095	-1.54705
H	5.90550	1.47616	-1.54523
C	8.43384	-0.78047	-1.14596
H	8.24617	-2.66619	-0.11375
H	8.30343	1.15186	-2.09627
H	9.48396	-0.91971	-1.38571
H	0.62519	1.72093	-3.15423
H	4.25835	-0.57657	-2.78293

Pro-*S* L23

Pd	-1.07573	0.11154	-0.37803
C	-2.53081	1.41366	0.42501
C	-3.11330	1.51102	1.70012
C	-2.13168	2.64406	-0.25041
C	-2.26272	3.91384	0.41565
C	-3.18048	-0.34491	-0.51548
C	-3.26549	-1.63565	0.15383
C	-4.15190	-0.02646	-1.47562
C	-4.31053	-2.56309	-0.18865
C	-5.17494	-0.97221	-1.77513
C	-2.40060	-3.33027	1.71511
C	-4.36978	-3.84128	0.43444
C	-3.43730	-4.22741	1.36649
H	-1.65742	-3.62207	2.45257
H	-5.17442	-4.51400	0.14526
H	-3.49217	-5.20833	1.83121
C	-2.32366	-2.08350	1.13129
H	-1.51540	-1.41529	1.41931
C	-5.26480	-2.19475	-1.16463
H	-6.06253	-2.88818	-1.41994
C	-4.25350	1.27460	-2.24216
H	-5.17251	1.28603	-2.83640
H	-3.41964	1.42078	-2.93570
H	-4.27413	2.14314	-1.57949
H	-5.91661	-0.69530	-2.52061
C	-1.81905	5.10827	-0.21854
C	-1.25476	5.08641	-1.47095
C	-1.12682	3.85162	-2.14819
C	-1.55336	2.68137	-1.55933
C	-2.83556	3.95249	1.70631
H	-2.94999	4.90969	2.20987
C	-3.25023	2.79165	2.30627
H	-3.70371	2.83840	3.29354
H	-1.94064	6.04727	0.31752
H	-0.92023	6.00644	-1.94303
H	-0.69704	3.82340	-3.14610
H	-1.45595	1.75148	-2.10893

C	-3.65667	0.36326	2.52241
H	-2.87888	-0.33952	2.83279
H	-4.13225	0.74915	3.42962
H	-4.40430	-0.21529	1.97295
P	0.66084	-1.26794	-1.26815
C	2.12473	-1.74488	-0.25455
C	1.32223	-0.83161	-3.01162
C	0.28507	-3.10345	-1.40991
C	2.94811	-0.97937	0.59201
C	2.35815	-3.12646	-0.38172
C	2.43496	-1.77722	-3.49555
C	0.11286	-0.90761	-3.96663
C	1.85141	0.61411	-2.98236
O	1.49242	-3.84883	-1.14549
C	4.02213	-1.61796	1.23240
C	2.65885	0.46487	0.86050
C	3.43420	-3.75702	0.24399
H	2.10646	-2.82044	-3.54402
H	2.75216	-1.48016	-4.50427
H	3.31321	-1.72788	-2.84410
H	-0.28176	-1.92538	-4.06528
H	-0.70644	-0.26069	-3.63430
H	0.42060	-0.57922	-4.96795
H	1.10423	1.31378	-2.59121
H	2.75865	0.70381	-2.37942
H	2.10166	0.92777	-4.00524
C	4.26874	-2.98184	1.04592
H	4.65649	-1.04418	1.90130
C	3.35805	1.49714	0.20537
C	1.62333	0.82532	1.75034
H	3.58809	-4.82308	0.11295
H	5.10719	-3.44986	1.55492
C	2.99846	2.83670	0.38832
C	1.26380	2.16391	1.93091
C	1.94677	3.16475	1.24154
C	1.48288	-0.67256	3.63365
H	1.64939	4.20239	1.36134
H	-0.07636	-3.39590	-2.39721
C	5.70901	1.11735	-0.26598
O	4.33250	1.17370	-0.71734
O	0.90830	-0.15547	2.40537
H	2.56200	-0.79955	3.47743
H	5.72389	0.64773	0.72618
H	-0.46568	-3.37273	-0.65875
C	6.43516	0.22177	-1.26201
H	7.49188	0.12306	-0.99019
H	5.98243	-0.77385	-1.28047
H	6.37638	0.64965	-2.26910
C	6.32218	2.51411	-0.17195
H	6.28838	3.01257	-1.14740
H	5.79075	3.13523	0.55542
H	7.36944	2.44584	0.14515
C	0.84452	-2.03692	3.85876
H	1.04687	-2.69781	3.01071
H	1.24360	-2.50020	4.76790
H	-0.24091	-1.93559	3.97256
C	1.24554	0.29167	4.79493
H	1.69812	1.26871	4.59838
H	0.17164	0.43425	4.96047
H	1.68807	-0.10727	5.71533
H	3.52274	3.60134	-0.17459
H	0.43080	2.39765	2.58500

Pro-*R* L23

Pd	-1.08999	-0.03479	0.23442
C	-2.88532	-1.21794	0.26214
C	-3.91027	-1.34763	1.21714
C	-2.48812	-2.41193	-0.47650
C	-3.13853	-3.67244	-0.23756
C	-2.94891	0.61626	-0.60419
C	-3.10983	1.87598	0.12495
C	-3.51434	0.53368	-1.89385
C	-3.79315	2.99255	-0.47583
C	-4.20137	1.65636	-2.43496
C	-2.67026	3.32947	2.07005
C	-3.89647	4.23414	0.21291
C	-3.34205	4.41214	1.45689
H	-2.24242	3.44976	3.06209
H	-4.42578	5.04757	-0.27883
H	-3.42536	5.36679	1.96957
C	-2.56761	2.11396	1.42851
H	-2.06683	1.29622	1.93578
C	-4.34318	2.84490	-1.76788
H	-4.87099	3.67976	-2.22287
C	-3.51273	-0.67954	-2.79938
H	-4.07269	-0.45890	-3.71353
H	-2.50832	-0.99024	-3.09797
H	-3.97989	-1.54701	-2.32438
H	-4.63158	1.55458	-3.42834
C	-2.72408	-4.83894	-0.94044
C	-1.69188	-4.80110	-1.84644
C	-1.02897	-3.57451	-2.08378
C	-1.41769	-2.43001	-1.42182
C	-4.17581	-3.74016	0.72013

H	-4.67804	-4.68774	0.89918
C	-4.52952	-2.61380	1.41568
H	-5.32630	-2.67546	2.15325
H	-3.24343	-5.77201	-0.73240
H	-1.38367	-5.70095	-2.37228
H	-0.20729	-3.52958	-2.79386
H	-0.88881	-1.50111	-1.62259
C	-4.47248	-0.23287	2.07177
H	-3.73007	0.19200	2.75396
H	-5.29541	-0.61299	2.68501
H	-4.86093	0.59364	1.47032
P	0.81654	-0.56719	1.60488
C	2.27995	0.54456	1.53219
C	1.50764	-2.33662	1.83533
C	0.57961	0.01485	3.38014
C	3.03775	0.98333	0.43243
C	2.59267	1.01240	2.82136
C	2.64563	-2.40636	2.86856
C	0.32080	-3.21112	2.28769
C	2.02447	-2.83692	0.47428
O	1.80649	0.59807	3.85636
H	-0.20066	0.78473	3.40636
C	4.11145	1.85713	0.67310
C	2.72824	0.57417	-0.97362
C	3.66888	1.86558	3.06082
H	2.33229	-2.07648	3.86437
H	2.98945	-3.44569	2.95714
H	3.50302	-1.79815	2.56326
H	-0.06534	-2.91203	3.26985
H	-0.50594	-3.17551	1.57140
H	0.65130	-4.25456	2.37283
H	2.32685	-3.88872	0.56968
H	1.24971	-2.78463	-0.29683
H	2.90006	-2.27265	0.14008
C	4.42642	2.28052	1.96707
H	4.68981	2.22661	-0.16784
C	3.58588	-0.29857	-1.67806
C	1.56994	1.04423	-1.63124
H	3.88285	2.19937	4.07081
H	5.26099	2.95949	2.12088
C	3.29812	-0.68381	-2.99161
C	1.28645	0.65715	-2.94661
C	2.15560	-0.19662	-3.62243
C	5.98740	-0.37439	-1.24080
H	1.92424	-0.50752	-4.63761
H	0.30195	-0.79095	4.06284
C	0.43762	3.20560	-1.20300
O	0.67083	1.79422	-0.90524
O	4.64512	-0.87009	-1.00662
H	5.93314	0.71602	-1.35801
H	-0.17474	3.50259	-0.34743
H	3.94434	-1.39918	-3.48692
H	0.36903	0.99372	-3.41330
C	6.77229	-0.71430	0.02107
H	6.30710	-0.25058	0.89526
H	6.79123	-1.79945	0.17252
H	7.80545	-0.35911	-0.06124
C	6.61151	-0.98579	-2.49565
H	6.07677	-0.68637	-3.40149
H	7.65105	-0.65249	-2.59693
H	6.60518	-2.07963	-2.42948
C	-0.37714	3.42681	-2.47742
H	0.21591	3.24105	-3.37915
H	-1.26656	2.79042	-2.49165
H	-0.70944	4.47111	-2.51017
C	1.73966	4.00274	-1.19487
H	1.51598	5.07040	-1.30136
H	2.28704	3.85371	-0.26037
H	2.38824	3.71178	-2.02944

Pro-*S* L24

Pd	-0.59687	0.21270	-0.21590
C	-2.43780	-0.58469	-1.00763
C	-3.29941	-0.10845	-2.00790
C	-2.27014	-2.02447	-0.86858
C	-2.96943	-2.92637	-1.74543
C	-2.47386	0.70554	0.65031
C	-2.41092	2.16391	0.58506
C	-3.22380	0.12554	1.69181
C	-3.03383	2.97195	1.60151
C	-3.83591	0.96745	2.66494
C	-1.63658	4.25650	-0.45954
C	-2.93351	4.39099	1.55854
C	-2.24779	5.02957	0.55432
H	-1.10498	4.74940	-1.26938
H	-3.42051	4.96162	2.34634
H	-2.18157	6.11402	0.53167
C	-1.71762	2.88144	-0.43841
H	-1.25007	2.31229	-1.23323
C	-3.74447	2.33423	2.64173
H	-4.22389	2.93730	3.40920
C	-3.49966	-1.34866	1.89600
H	-4.19203	-1.48126	2.73322
H	-2.59839	-1.92419	2.12153

H	-3.95393	-1.80585	1.01337
H	-4.40376	0.48957	3.45953
C	-2.79683	-4.33282	-1.61651
C	-1.96100	-4.86674	-0.66571
C	-1.25484	-3.99679	0.19664
C	-1.40440	-2.63074	0.09048
C	-3.82067	-2.39553	-2.74081
H	-4.35526	-3.07213	-3.40319
C	-3.96595	-1.03906	-2.85689
H	-4.62677	-0.63996	-3.62254
H	-3.34463	-4.97942	-2.29868
H	-1.83887	-5.94313	-0.57796
H	-0.58205	-4.39955	0.94840
H	-0.84174	-1.98730	0.76152
C	-3.63615	1.34021	-2.28753
H	-2.77869	1.90486	-2.66673
H	-4.42082	1.39864	-3.04806
H	-3.99707	1.86102	-1.39788
P	1.69468	0.04428	-1.04618
C	3.16979	0.02683	0.05151
C	2.04113	-1.35778	-2.31798
C	2.45253	1.54183	-1.93568
C	3.42746	-0.64581	1.26128
C	4.17238	0.83744	-0.50922
C	3.44413	-1.29496	-2.94935
C	1.88829	-2.70622	-1.59320
C	0.96565	-1.23551	-3.41538
O	3.87276	1.53211	-1.64254
H	2.36874	1.37691	-3.01458
C	1.86248	2.92650	-1.61038
C	4.71296	-0.54578	1.81972
C	2.41264	-1.45077	2.00682
C	5.44795	0.93707	0.04645
H	4.23354	-1.37811	-2.19594
H	3.55696	-2.13989	-3.64170
H	3.61662	-0.37829	-3.52090
H	2.64824	-2.83130	-0.81486
H	0.90352	-2.81935	-1.13436
H	2.01669	-3.52229	-2.31695
H	-0.04458	-1.30936	-3.00126
H	1.04051	-0.28839	-3.96443
H	1.09560	-2.04524	-4.14531
C	2.01175	3.32987	-0.13620
C	2.47553	3.99165	-2.53594
C	5.70878	0.22382	1.21377
H	4.91902	-1.06075	2.75300
C	1.33838	-0.82568	2.68357
C	2.58276	-2.83011	2.16279
H	6.19594	1.56570	-0.42553
H	3.06726	3.45007	0.13170
H	1.50716	4.28527	0.04294
H	1.57135	2.59015	0.53991
H	3.55642	4.06866	-2.37682
H	2.30564	3.75278	-3.59317
H	2.03328	4.97503	-2.33869
H	6.69263	0.28590	1.67119
C	0.46468	-1.58799	3.46966
O	1.24071	0.52756	2.54292
C	1.71119	-3.59654	2.94131
C	0.65258	-2.96763	3.59225
H	-0.35759	-1.11304	3.99157
C	0.30473	1.23084	3.35571
H	1.86803	-4.66631	3.04399
H	-0.03322	-3.54296	4.20854
H	0.45576	2.28736	3.13052
H	-0.72699	0.95630	3.11218
H	0.49871	1.05011	4.42089
H	0.79190	2.85053	-1.83947
H	3.42283	-3.30571	1.66362

Pro-*R* L24

Pd	-0.58017	-0.05454	0.18165
C	-2.25850	1.18442	-0.28438
C	-3.03834	1.36151	-1.44504
C	-1.93775	2.37194	0.50481
C	-2.32879	3.68257	0.05426
C	-2.59090	-0.74654	0.49355
C	-2.70568	-1.90407	-0.38019
C	-3.41934	-0.68351	1.62462
C	-3.63935	-2.95649	-0.07913
C	-4.32506	-1.75171	1.88777
C	-2.02145	-3.20011	-2.35567
C	-3.73948	-4.09247	-0.93034
C	-2.95445	-4.21825	-2.05112
H	-1.38709	-3.29319	-3.23311
H	-4.45768	-4.86632	-0.66813
H	-3.04355	-5.08962	-2.69443
C	-1.90321	-2.09350	-1.54397
H	-1.17205	-1.32721	-1.78773
C	-4.44526	-2.84871	1.07645
H	-5.15601	-3.63823	1.30810
C	-3.48028	0.44927	2.62549
H	-4.28956	0.27070	3.34013
H	-2.55670	0.54908	3.20429

H	-3.66599	1.41337	2.14698
H	-4.95250	-1.67477	2.77247
C	-1.98684	4.83962	0.80851
C	-1.27984	4.74576	1.98200
C	-0.89166	3.46922	2.44758
C	-1.20951	2.33525	1.73294
C	-3.05881	3.80378	-1.14763
H	-3.35963	4.78980	-1.49394
C	-3.40158	2.67932	-1.84941
H	-3.99077	2.78328	-2.75724
H	-2.30631	5.80821	0.42978
H	-1.02786	5.63690	2.55058
H	-0.34166	3.37720	3.38028
H	-0.91037	1.36880	2.11929
C	-3.61442	0.27943	-2.33530
H	-2.85510	-0.29458	-2.87216
H	-4.26978	0.73283	-3.08523
H	-4.20818	-0.44163	-1.76820
P	1.67782	-0.91070	0.83926
C	3.18568	-0.59070	-0.16680
C	2.17308	-0.31660	2.60578
C	2.20603	-2.74473	0.96177
C	3.60090	0.53867	-0.90204
C	4.03310	-1.71065	-0.10051
C	3.52944	-0.85559	3.09968
C	2.24980	1.22037	2.59151
C	1.05417	-0.77943	3.55990
O	3.60827	-2.79613	0.60006
H	2.15785	-3.02219	2.01924
C	1.46524	-3.82883	0.16033
C	4.86755	0.51163	-1.50892
C	2.79770	1.79089	-1.04958
C	5.29064	-1.73527	-0.70487
H	4.34837	-0.56174	2.43608
H	3.73749	-0.43072	4.09076
H	3.55010	-1.94439	3.20228
H	3.10183	1.56812	2.00069
H	1.35026	1.68696	2.18325
H	2.38639	1.58728	3.61798
H	0.07527	-0.39613	3.25418
H	0.97892	-1.87235	3.61149
H	1.26335	-0.41826	4.57542
C	1.43931	-3.56061	-1.34978
C	5.69923	-0.60592	-1.40805
H	5.19143	1.37855	-2.07644
C	1.66174	1.85225	-1.88677
C	3.23546	2.97139	-0.43827
H	5.91207	-2.62071	-0.62042
H	2.45376	-3.45350	-1.74719
H	0.95874	-4.39485	-1.87273
H	0.87827	-2.65256	-1.58865
H	6.67201	-0.59801	-1.89255
C	0.97515	3.06121	-2.05395
O	1.31643	0.69135	-2.52059
C	2.55599	4.17996	-0.60122
C	1.41988	4.21506	-1.40531
H	0.08921	3.10991	-2.67555
C	0.31541	0.75230	-3.52747
H	2.91195	5.07805	-0.10520
H	0.86826	5.14153	-1.53713
H	0.25791	-0.25277	-3.94965
H	-0.65863	1.03016	-3.10924
H	0.59183	1.46318	-4.31747
C	0.07858	-4.10276	0.75676
H	-0.42314	-4.90688	0.20826
H	-0.56610	-3.22176	0.70965
H	0.15717	-4.41222	1.80705
H	2.07722	-4.72981	0.31823
H	4.13024	2.93179	0.17743

Pro-*S* L26

Pd	-0.82844	0.28826	-0.33731
C	-1.76397	2.14089	0.08935
C	-2.16026	2.75513	1.28959
C	-1.16876	2.97501	-0.94939
C	-0.92246	4.37291	-0.70798
C	-2.98387	0.44626	-0.24039
C	-3.33519	-0.50532	0.80278
C	-3.94909	0.76026	-1.20813
C	-4.63633	-1.11861	0.82052
C	-5.22856	0.13525	-1.14694
C	-2.77121	-1.85847	2.77903
C	-4.96414	-2.07876	1.81877
C	-4.05625	-2.44962	2.78116
H	-2.04425	-2.14295	3.53582
H	-5.95790	-2.52092	1.79621
H	-4.31895	-3.18682	3.53520
C	-2.43132	-0.92284	1.82591
H	-1.43947	-0.48027	1.84761
C	-5.57472	-0.76774	-0.17704
H	-6.56392	-1.21908	-0.16308
C	-3.78594	1.74656	-2.34415
H	-4.72870	1.84426	-2.89122
H	-3.02486	1.43477	-3.06562

H	-3.50197	2.74032	-1.98937
H	-5.95756	0.40132	-1.90855
C	-0.29627	5.17636	-1.70173
C	0.08681	4.65042	-2.91138
C	-0.15990	3.28353	-3.17588
C	-0.76483	2.48453	-2.23048
C	-1.31583	4.93322	0.52704
H	-1.14344	5.99106	0.71137
C	-1.91848	4.14630	1.47246
H	-2.23198	4.59393	2.41241
H	-0.12832	6.22752	-1.47718
H	0.56429	5.27574	-3.66100
H	0.12218	2.85828	-4.13552
H	-0.95517	1.44305	-2.46411
C	-2.87567	2.08906	2.44376
H	-2.28227	1.29893	2.90873
H	-3.10073	2.83019	3.21714
H	-3.82097	1.63642	2.13135
P	0.57479	-1.59325	-0.99678
C	1.79918	-2.31635	0.18699
C	1.50230	-1.33330	-2.66160
C	-0.15543	-3.34523	-1.26340
C	2.75597	-1.72891	1.04750
C	1.65100	-3.71627	0.20965
C	2.52175	-2.44808	-2.95221
C	0.43892	-1.28838	-3.77936
C	2.21795	0.02855	-2.61874
O	0.71287	-4.28235	-0.58743
C	3.53805	-2.56697	1.85993
C	2.99926	-0.25315	1.17362
C	2.43306	-4.54593	1.01574
H	2.06114	-3.44116	-2.99342
H	2.99609	-2.26471	-3.92550
H	3.31317	-2.47591	-2.19719
H	-0.06814	-2.24757	-3.92675
H	-0.32621	-0.53026	-3.58097
H	0.92810	-1.03099	-4.72774
H	1.52241	0.84882	-2.41507
H	3.00615	0.05456	-1.86467
H	2.68821	0.22135	-3.59262
C	3.38318	-3.95480	1.84032
H	4.27379	-2.11348	2.51704
C	4.09966	0.34162	0.51164
C	2.21180	0.51184	2.06657
H	2.27928	-5.61958	0.98628
H	4.00225	-4.57600	2.48196
C	4.34399	1.75864	0.66281
C	2.47746	1.92363	2.22577
C	3.51675	2.51296	1.49988
C	0.48482	0.68352	3.78638
H	3.70599	3.57831	1.61364
H	-0.07700	-3.59268	-2.32727
C	5.44546	2.35386	-0.02936
C	5.01009	-0.40656	-0.30537
C	1.18634	-0.07017	2.88045
C	1.69585	2.67969	3.15469
C	6.06454	0.19981	-0.93819
H	1.89875	3.74338	3.25138
C	0.73277	2.07973	3.92034
H	5.60739	3.42288	0.08668
C	6.28361	1.60135	-0.80759
H	0.99088	-1.13317	2.79081
H	-0.27087	0.21358	4.41017
H	0.15726	2.66173	4.63459
H	4.85933	-1.47568	-0.40874
H	6.74267	-0.39285	-1.54640
H	7.12022	2.06568	-1.32237
C	-1.61854	-3.55706	-0.82901
H	-1.76759	-3.08160	0.14809
C	-1.94842	-5.05324	-0.69495
H	-1.32448	-5.54146	0.05749
H	-2.99823	-5.17502	-0.40534
H	-1.80076	-5.57850	-1.64797
C	-2.56098	-2.89622	-1.84580
H	-2.34976	-1.83039	-1.97091
H	-2.47735	-3.38656	-2.82565
H	-3.59990	-2.98474	-1.51365

Pro-*R* L26

Pd	-1.03298	0.06295	0.21726
C	-2.11997	-1.46761	1.28037
C	-3.19095	-1.38712	2.18349
C	-1.13003	-2.51595	1.47533
C	-1.24970	-3.43506	2.57612
C	-2.74842	-0.86057	-0.64524
C	-3.55523	0.31999	-0.93932
C	-2.91203	-1.99107	-1.46642
C	-4.42096	0.34874	-2.09010
C	-3.79668	-1.92203	-2.58071
C	-4.27049	2.63073	-0.47135
C	-5.17166	1.51726	-2.39972
C	-5.10180	2.64232	-1.61485
H	-4.22112	3.50668	0.17050
H	-5.81178	1.49476	-3.27907

H	-5.68408	3.52637	-1.86071
C	-3.52929	1.51354	-0.15448
H	-2.90911	1.52357	0.73388
C	-4.51658	-0.80213	-2.90198
H	-5.17497	-0.79203	-3.76752
C	-2.25627	-3.34304	-1.28774
H	-2.65891	-4.04750	-2.02231
H	-1.17329	-3.30871	-1.42726
H	-2.43793	-3.75932	-0.29374
H	-3.89670	-2.81013	-3.19996
C	-0.27175	-4.44929	2.77469
C	0.80844	-4.57476	1.93493
C	0.95042	-3.67366	0.85451
C	0.01588	-2.68353	0.64100
C	-2.34417	-3.30804	3.46125
H	-2.44324	-4.00256	4.29201
C	-3.26744	-2.31652	3.26137
H	-4.10908	-2.22903	3.94437
H	-0.39886	-5.12374	3.61884
H	1.54957	-5.35252	2.09943
H	1.80679	-3.75071	0.19110
H	0.15346	-1.99805	-0.19131
C	-4.33547	-0.39916	2.12823
H	-4.01174	0.62891	2.31950
H	-5.07928	-0.64923	2.89103
H	-4.83871	-0.40364	1.15888
P	0.77826	1.45755	1.08664
C	2.18941	2.09299	0.07442
C	1.57291	0.86842	2.73674
C	0.36260	3.26114	1.53365
C	3.02264	1.47808	-0.88565
C	2.38635	3.45723	0.36579
C	2.59612	1.86179	3.31691
C	0.41479	0.65821	3.73351
C	2.27457	-0.47590	2.48307
O	1.53949	4.05579	1.24538
C	4.04662	2.24320	-1.47266
C	2.87166	0.06605	-1.36909
C	3.41137	4.20874	-0.20899
H	2.15862	2.83300	3.56696
H	3.01385	1.44140	4.24129
H	3.42860	2.03418	2.62729
H	-0.10805	1.59318	3.96994
H	-0.32139	-0.05746	3.35410
H	0.81755	0.26499	4.67600
H	1.59405	-1.22488	2.07325
H	3.12044	-0.37030	1.79755

H	2.66689	-0.86280	3.43313
C	4.24496	3.58190	-1.13040
H	4.68521	1.77484	-2.21517
C	3.84714	-0.90444	-1.03339
C	1.82602	-0.25844	-2.26765
H	3.52567	5.25450	0.05674
H	5.04476	4.14556	-1.60312
C	3.74855	-2.23798	-1.58430
C	1.73651	-1.59595	-2.80899
C	2.69536	-2.54815	-2.45036
C	5.87977	-1.58934	0.14377
H	2.62752	-3.55155	-2.86607
H	0.22696	3.31257	2.61810
C	5.77556	-2.90117	-0.40081
C	4.73855	-3.21163	-1.23906
C	4.95077	-0.62787	-0.16088
H	5.04841	0.36775	0.25765
H	6.70575	-1.35044	0.80825
H	6.52134	-3.64992	-0.14840
H	4.64877	-4.20853	-1.66445
C	0.67850	-1.91122	-3.71946
C	0.85622	0.70143	-2.70545
C	-0.23676	-0.96523	-4.09582
C	-0.13794	0.36107	-3.58708
H	0.62310	-2.92392	-4.11190
H	-1.03856	-1.21787	-4.78321
H	-0.86056	1.10860	-3.90164
H	0.93069	1.72005	-2.34337
C	-0.86891	3.90493	0.87156
H	-1.71804	3.27063	1.15515
C	-0.79655	3.95236	-0.66068
H	-0.71198	2.94958	-1.09240
H	-1.70779	4.40296	-1.06747
H	0.05602	4.55246	-0.99812
C	-1.10670	5.30861	1.45537
H	-1.22216	5.27674	2.54588
H	-0.26815	5.97398	1.22466
H	-2.01682	5.75120	1.03449

Pro-*S* L27

Pd	0.88989	0.02975	0.12053
C	2.31470	1.53651	-0.33935
C	3.03928	1.85606	-1.50380
C	1.75365	2.63733	0.44212
C	1.89087	3.99869	-0.00863
C	2.99755	-0.26073	0.44128
C	3.28303	-1.42597	-0.38585

C	3.82118	-0.01461	1.55069
C	4.37416	-2.30284	-0.05492
C	4.89549	-0.90592	1.83506
C	2.76242	-2.93289	-2.26280
C	4.62879	-3.46102	-0.84204
C	3.84443	-3.77895	-1.92436
H	2.13251	-3.17260	-3.11552
H	5.46273	-4.09893	-0.55727
H	4.04828	-4.66903	-2.51351
C	2.49871	-1.80218	-1.51964
H	1.65771	-1.17145	-1.79866
C	5.17568	-2.00832	1.07180
H	6.00755	-2.66248	1.32139
C	3.70513	1.15669	2.50176
H	4.52896	1.13477	3.22183
H	2.77398	1.14330	3.07613
H	3.74443	2.11666	1.98040
H	5.51928	-0.68573	2.69808
C	1.29849	5.06493	0.72560
C	0.58007	4.83131	1.87283
C	0.43985	3.50397	2.33920
C	1.00859	2.45469	1.65091
C	2.61490	4.25725	-1.19386
H	2.73179	5.28345	-1.53430
C	3.16679	3.21834	-1.89726
H	3.73299	3.43318	-2.80036
H	1.42984	6.07789	0.35112
H	0.13265	5.65506	2.42267
H	-0.11208	3.30694	3.25452
H	0.89637	1.44966	2.04269
C	3.76335	0.87757	-2.40400
H	3.09214	0.16784	-2.89463
H	4.29599	1.42191	-3.18996
H	4.49784	0.28363	-1.85288
P	-0.71578	-1.63279	0.76216
C	-2.12337	-2.14299	-0.30186
C	-1.41952	-1.56643	2.54101
C	-0.06981	-3.39154	0.61371
C	-3.12922	-1.38279	-0.92628
C	-2.13274	-3.54410	-0.43435
C	-2.33115	-2.76062	2.87306
C	-0.21052	-1.54606	3.49741
C	-2.21553	-0.25639	2.69326
O	-1.14001	-4.25040	0.17781
C	-4.13543	-2.06047	-1.63425
C	-3.18317	0.10664	-0.87569
C	-3.13047	-4.21435	-1.14026
H	-1.81014	-3.71994	2.78905
H	-2.69103	-2.66790	3.90669
H	-3.20699	-2.79256	2.21769
H	0.37481	-2.47161	3.44725
H	0.46559	-0.71250	3.27905
H	-0.56316	-1.43786	4.53152
H	-1.61973	0.61924	2.41144
H	-3.12578	-0.26330	2.08626
H	-2.51805	-0.13454	3.74234
C	-4.13554	-3.45278	-1.73380
H	-4.91771	-1.48253	-2.11572
C	-4.23021	0.79565	-0.24547
C	-2.27688	0.94882	-1.53744
H	-3.10524	-5.29643	-1.21662
H	-4.92320	-3.95040	-2.29333
C	-4.37355	2.18565	-0.25091
C	-2.39405	2.34380	-1.57705
C	-3.44522	2.97996	-0.92218
H	-3.53843	4.06323	-0.93538
H	0.31618	-3.78627	1.55616
C	-5.57364	2.53475	0.60170
C	-1.22283	2.88938	-2.36295
H	-0.48251	3.36132	-1.70530
H	-5.27050	2.97087	1.56359
H	0.72842	-3.41575	-0.13534
O	-5.20787	0.15818	0.47231
O	-1.19245	0.48633	-2.23143
C	-0.66295	1.60042	-3.00380
C	-6.22351	1.14357	0.78789
H	-7.06009	0.99639	0.09335
H	-6.56885	0.94383	1.80456
H	-6.26364	3.24107	0.12696
H	-1.51016	3.62491	-3.12242
H	-1.00987	1.47679	-4.03707
H	0.42463	1.53024	-2.97159

Pro-*R* L27

Pd	-0.89636	-0.21123	-0.29686
C	-2.50625	1.13821	-0.73568
C	-3.56916	1.05753	-1.64788
C	-1.91277	2.44303	-0.48020
C	-2.40311	3.61155	-1.16063
C	-2.73910	-0.28963	0.75878
C	-3.04760	-1.70425	0.55255
C	-3.21949	0.32678	1.93017
C	-3.73781	-2.45680	1.56858
C	-3.92187	-0.45273	2.89269

C	-2.91032	-3.79024	-0.75412
C	-3.98164	-3.84860	1.39618
C	-3.57216	-4.51211	0.26570
H	-2.60187	-4.29928	-1.66361
H	-4.50550	-4.37749	2.18946
H	-3.76354	-5.57528	0.14791
C	-2.66427	-2.44202	-0.61348
H	-2.17431	-1.91158	-1.42207
C	-4.16529	-1.79282	2.73997
H	-4.69557	-2.35271	3.50673
C	-3.10868	1.79536	2.28291
H	-3.65468	1.99446	3.21033
H	-2.07800	2.12818	2.43127
H	-3.53240	2.43368	1.50256
H	-4.27677	0.05016	3.78907
C	-1.80200	4.88088	-0.92955
C	-0.74273	5.02534	-0.06641
C	-0.24042	3.88621	0.60514
C	-0.80750	2.64685	0.40098
C	-3.47769	3.47667	-2.06958
H	-3.85877	4.35736	-2.58084
C	-4.02619	2.24143	-2.29474
H	-4.85475	2.14805	-2.99278
H	-2.20159	5.74048	-1.46334
H	-0.29293	6.00086	0.09859
H	0.59887	3.98340	1.28888
H	-0.39774	1.78681	0.92584
C	-4.32690	-0.19926	-2.01625
H	-3.70557	-0.91963	-2.55787
H	-5.17120	0.05081	-2.66617
H	-4.72358	-0.71479	-1.13815
P	1.05543	-0.56006	-1.65627
C	2.49492	-1.53705	-1.06868
C	1.81683	0.86586	-2.68294
C	0.78272	-1.91221	-2.93448
C	3.28047	-1.40264	0.08936
C	2.81404	-2.53008	-2.01172
C	2.90760	0.39294	-3.66039
C	0.65769	1.51759	-3.46224
C	2.42195	1.88877	-1.70343
O	2.00490	-2.65171	-3.10509
H	0.00929	-2.59721	-2.56606
C	4.40703	-2.22914	0.23103
C	2.94204	-0.45706	1.19038
C	3.92786	-3.35510	-1.86781
H	2.53123	-0.32471	-4.39632
H	3.29484	1.26009	-4.21209
H	3.74980	-0.06924	-3.13618
H	0.20005	0.82374	-4.17828
H	-0.12750	1.88210	-2.79339
H	1.03989	2.37196	-4.03618
H	2.78962	2.75512	-2.27012
H	1.68023	2.25206	-0.98547
H	3.27025	1.46864	-1.15300
C	4.72686	-3.18193	-0.73816
H	5.02393	-2.12952	1.11837
C	3.76552	0.61450	1.56403
C	1.82851	-0.62882	2.02658
H	4.14743	-4.10646	-2.61924
H	5.60049	-3.81353	-0.60079
C	3.51423	1.45341	2.65443
C	1.55015	0.18013	3.13524
C	2.39781	1.23680	3.46146
C	5.36496	2.22030	1.39855
H	2.19815	1.86919	4.32343
H	0.48168	-1.52830	-3.91187
C	4.65798	2.43895	2.75678
H	5.08284	2.99105	0.67109
H	4.33591	3.47884	2.87986
C	0.26569	-0.31718	3.76221
C	0.08919	-1.67619	3.04969
H	0.32658	-0.43584	4.84959
H	-0.93405	-1.88899	2.74137
O	4.90853	0.94205	0.88618
O	0.90377	-1.61902	1.84450
H	6.45434	2.17632	1.46426
H	5.32110	2.20097	3.60003
H	-0.57766	0.35192	3.54894
H	0.46866	-2.50403	3.66104

Pro-*S* L28

Pd	0.54592	-0.19008	-0.28035
C	2.36730	0.88544	-0.67113
C	3.30899	0.78585	-1.70479
C	2.09065	2.19807	-0.10700
C	2.75976	3.36354	-0.62010
C	2.39695	-0.86848	0.48357
C	2.36518	-2.23498	-0.03252
C	3.08639	-0.62808	1.68599
C	2.94386	-3.31301	0.72675
C	3.67253	-1.72261	2.38390
C	1.66474	-3.91432	-1.69495
C	2.85389	-4.65434	0.25863
C	2.22133	-4.95943	-0.92174

H	1.18399	-4.14068	-2.64321
H	3.30377	-5.43818	0.86416
H	2.15924	-5.98809	-1.26649
C	1.73961	-2.60712	-1.26587
H	1.32205	-1.82451	-1.88926
C	3.59862	-3.01841	1.94338
H	4.04905	-3.82630	2.51524
C	3.31338	0.72051	2.33478
H	3.96070	0.60583	3.20984
H	2.38761	1.19469	2.67102
H	3.79627	1.42474	1.65193
H	4.19789	-1.50798	3.31142
C	2.46723	4.65062	-0.08790
C	1.54272	4.81755	0.91489
C	0.86940	3.68430	1.42787
C	1.13782	2.42689	0.93179
C	3.69906	3.21146	-1.66598
H	4.21439	4.08757	-2.05221
C	3.95180	1.96632	-2.17802
H	4.68007	1.85902	-2.97834
H	2.99391	5.50723	-0.50305
H	1.32798	5.80700	1.30984
H	0.13209	3.79859	2.21800
H	0.60149	1.57480	1.34239
C	3.75085	-0.49158	-2.38507
H	2.95287	-0.94692	-2.98027
H	4.58157	-0.28317	-3.06639
H	4.08784	-1.24546	-1.66966
P	-1.61262	0.07159	-1.29968
C	-3.14914	-0.61938	-0.56059
C	-2.12131	1.75360	-2.06045
C	-1.87122	-1.13252	-2.71973
C	-3.69616	-0.48157	0.72886
C	-3.83651	-1.37868	-1.52434
C	-3.43545	1.68516	-2.85823
C	-2.26952	2.77203	-0.91586
C	-0.96480	2.18670	-2.98342
O	-3.25302	-1.53258	-2.74876
H	-1.62305	-0.70955	-3.69567
C	-4.95362	-1.05252	0.98363
C	-3.00319	0.22838	1.84660
C	-5.08361	-1.94811	-1.26944
H	-4.27805	1.38581	-2.22687
H	-3.66151	2.67990	-3.26504
H	-3.38094	0.98819	-3.70044
H	-3.08573	2.50255	-0.23822
H	-1.35163	2.86279	-0.32770
H	-2.49844	3.76022	-1.33693
H	-0.01701	2.25399	-2.44037
H	-0.82733	1.49821	-3.82623
H	-1.18697	3.17587	-3.40461
C	-5.63812	-1.76493	-0.00457
H	-5.38209	-0.95485	1.97646
C	-1.86849	-0.33779	2.47457
C	-3.52700	1.41631	2.36601
H	-5.58601	-2.52039	-2.04236
H	-6.60679	-2.20188	0.22295
C	-1.27932	0.30422	3.57115
O	-1.42599	-1.52234	1.96411
C	-2.93993	2.06431	3.45623
C	-1.81355	1.50302	4.05257
H	-0.41186	-0.12740	4.05680
C	-0.44424	-2.25489	2.69246
H	-3.36478	2.98905	3.83528
H	-1.34523	1.98685	4.90577
H	-0.31690	-3.19346	2.15197
H	0.51572	-1.72845	2.71973
H	-0.78871	-2.45917	3.71453
H	-1.25293	-2.02214	-2.54754
H	-4.41499	1.83594	1.90037

Pro-*S* L29

Pd	-0.90953	0.05655	-0.36112
C	-2.20876	1.66395	0.14344
C	-2.77818	2.09134	1.35611
C	-1.71869	2.68180	-0.78185
C	-1.74648	4.07448	-0.41681
C	-3.04635	-0.19682	-0.40312
C	-3.27380	-1.26679	0.55624
C	-3.98180	-0.01340	-1.43180
C	-4.42239	-2.12378	0.43165
C	-5.10807	-0.88283	-1.51368
C	-2.59661	-2.60574	2.50460
C	-4.62378	-3.19111	1.35162
C	-3.73479	-3.43518	2.37038
H	-1.88522	-2.79040	3.30595
H	-5.50364	-3.81789	1.22321
H	-3.90019	-4.25491	3.06449
C	-2.38014	-1.56496	1.62823
H	-1.49738	-0.94386	1.75039
C	-5.33457	-1.90021	-0.62486
H	-6.20930	-2.53905	-0.72004
C	-3.93855	1.06328	-2.49407
H	-4.85230	1.03045	-3.09551

H	-3.09571	0.94115	-3.18156
H	-3.85857	2.06408	-2.06347
H	-5.81742	-0.71544	-2.32067
C	-1.22304	5.06227	-1.29738
C	-0.68130	4.72359	-2.51320
C	-0.65706	3.36378	-2.89905
C	-1.15819	2.38906	-2.06424
C	-2.30353	4.44431	0.82657
H	-2.33758	5.49520	1.10435
C	-2.80468	3.48203	1.66203
H	-3.24669	3.78179	2.60905
H	-1.26435	6.10206	-0.98010
H	-0.28484	5.48837	-3.17568
H	-0.24722	3.08211	-3.86548
H	-1.14018	1.35643	-2.39141
C	-3.42342	1.21641	2.40842
H	-2.71235	0.54048	2.88964
H	-3.86062	1.84350	3.19168
H	-4.22241	0.59634	1.99388
P	0.77452	-1.50174	-1.14674
C	2.13219	-2.16331	-0.08063
C	1.60162	-0.99118	-2.80659
C	0.27224	-3.29675	-1.52291
C	2.99474	-1.54074	0.84841
C	2.23747	-3.55503	-0.27526
C	2.73674	-1.93964	-3.22989
C	0.49924	-0.98523	-3.88691
C	2.15136	0.43862	-2.65470
O	1.38561	-4.14796	-1.15196
C	3.94412	-2.33085	1.51842
C	2.95766	-0.07744	1.17892
C	3.18700	-4.33448	0.38675
H	2.39652	-2.97287	-3.35405
H	3.14887	-1.60699	-4.19195
H	3.55375	-1.94012	-2.50178
H	0.12938	-1.98929	-4.12153
H	-0.35840	-0.37158	-3.59226
H	0.90887	-0.56751	-4.81571
H	1.37243	1.14842	-2.35882
H	2.95691	0.49131	-1.91923
H	2.56279	0.77318	-3.61683
C	4.04290	-3.70446	1.28482
H	4.60741	-1.85386	2.23355
C	3.91580	0.79863	0.61541
C	2.03779	0.40189	2.14209
H	3.23069	-5.40215	0.19853
H	4.78639	-4.28927	1.81975
C	3.88481	2.20425	0.95304
C	2.02522	1.80666	2.48228
C	2.93155	2.67174	1.86217
C	0.29866	0.02298	3.81626
H	2.91193	3.73006	2.11373
H	0.15593	-3.41508	-2.60274
C	4.84656	3.08442	0.36404
C	4.94213	0.35308	-0.28149
C	1.13991	-0.46312	2.84802
C	1.10750	2.27637	3.47363
C	5.85492	1.22775	-0.81252
H	1.10144	3.33828	3.70666
C	0.27162	1.41251	4.12863
H	4.80201	4.13988	0.62191
C	5.80542	2.61508	-0.49300
H	1.15194	-1.52376	2.62213
H	-0.35697	-0.65690	4.35397
H	-0.41032	1.77928	4.89060
H	4.99580	-0.70132	-0.53018
H	6.62586	0.86133	-1.48514
H	6.53328	3.29409	-0.92851
C	-0.98165	-3.78679	-0.81383
H	-0.90271	-3.67120	0.27042
H	-1.85619	-3.22112	-1.14660
H	-1.13596	-4.84728	-1.04242

Pro-*S* L29

Pd	-0.94280	0.03960	-0.07170
C	-2.31430	1.64280	0.24110
C	-3.13910	2.01840	1.31650
C	-1.60130	2.69450	-0.47880
C	-1.69010	4.06460	-0.04420
C	-3.02190	-0.13390	-0.59340
C	-3.46270	-1.26620	0.20960
C	-3.71140	0.14410	-1.78370
C	-4.56280	-2.08420	-0.22560
C	-4.80070	-0.68790	-2.17170
C	-3.23660	-2.76340	2.15200
C	-4.96690	-3.21160	0.54360
C	-4.32330	-3.55330	1.70840
H	-2.72120	-3.02050	3.07400
H	-5.80210	-3.80590	0.17920
H	-4.64180	-4.41840	2.28380
C	-2.82850	-1.66460	1.42630
H	-1.99120	-1.07110	1.78580
C	-5.22290	-1.76200	-1.43360
H	-6.06100	-2.37090	-1.76360

C	-3.42900	1.29560	-2.72410
H	-4.17700	1.31710	-3.52280
H	-2.44790	1.21950	-3.20270
H	-3.45940	2.26100	-2.21250
H	-5.31770	-0.44410	-3.09680
C	-0.95150	5.08210	-0.71070
C	-0.13660	4.79300	-1.77810
C	-0.04480	3.45710	-2.23140
C	-0.75340	2.45440	-1.60660
C	-2.52070	4.38170	1.05270
H	-2.60380	5.41660	1.37690
C	-3.21830	3.38960	1.69040
H	-3.86610	3.65000	2.52400
H	-1.05070	6.10400	-0.35080
H	0.42180	5.58030	-2.27760
H	0.58240	3.21700	-3.08590
H	-0.67550	1.44310	-1.99030
C	-4.01570	1.09850	2.13870
H	-3.44340	0.36670	2.71510
H	-4.60450	1.68620	2.84990
H	-4.71350	0.53200	1.51610
P	0.56440	-1.74260	-0.62110
C	1.96410	-2.28150	0.45470
C	1.20900	-1.80970	-2.42350
C	-0.17140	-3.45590	-0.37550
C	3.00060	-1.54880	1.06920
C	1.88490	-3.67150	0.66980
C	2.10230	-3.03200	-2.69690
C	-0.03380	-1.85090	-3.33670
C	1.99690	-0.51760	-2.70550
O	0.85750	-4.35050	0.08990
C	3.93540	-2.24420	1.85540
C	3.14530	-0.06240	0.93960
C	2.82210	-4.35970	1.44040
H	1.58260	-3.97810	-2.51350
H	2.41970	-3.02500	-3.74830
H	3.00480	-3.01830	-2.07760
H	-0.60590	-2.77860	-3.22370
H	-0.71190	-1.01410	-3.13790
H	0.28480	-1.79090	-4.38560
H	1.40170	0.37680	-2.49320
H	2.91450	-0.45980	-2.11420
H	2.28370	-0.48880	-3.76530
C	3.85040	-3.62770	2.02920
H	4.73130	-1.68550	2.33840
C	4.20060	0.47890	0.16530
C	2.26980	0.79610	1.64750

H	2.72610	-5.43200	1.57570
H	4.58640	-4.13760	2.64510
C	4.34950	1.91380	0.06520
C	2.42850	2.22850	1.53770
C	3.45650	2.74680	0.74500
C	0.41540	1.16600	3.19790
H	3.56950	3.82580	0.66310
H	-0.59070	-3.87540	-1.29190
C	5.41210	2.45240	-0.72670
C	5.13930	-0.33650	-0.54940
C	1.23380	0.30620	2.50900
C	1.54070	3.08750	2.25820
C	6.14560	0.22030	-1.29650
H	1.66460	4.16200	2.14920
C	0.56130	2.57600	3.06600
H	5.50790	3.53380	-0.78940
C	6.28810	1.63450	-1.38850
H	1.12050	-0.76470	2.63370
H	-0.35450	0.76850	3.85360
H	-0.11010	3.23870	3.60370
H	5.04710	-1.41530	-0.48890
H	6.84340	-0.42290	-1.82600
H	7.09170	2.05720	-1.98530
H	-0.96040	-3.40400	0.38180

4.2 GFN2-xTB Coordinates

L1

C	-1.72812	2.74794	1.36035
C	-2.87166	2.29366	0.48698
C	-2.72046	1.38376	-0.56414
Pd	-0.88623	0.57444	-0.92813
P	1.32029	-0.28685	-1.32767
C	2.09814	1.02401	-0.30397
C	2.86944	1.02617	0.85680
C	3.09148	2.25374	1.50227
C	2.54963	3.43309	1.02477
C	1.76067	3.44292	-0.12294
C	1.55112	2.23583	-0.75519
O	0.73641	2.12725	-1.86731
C	1.22096	1.04004	-2.68305
C	3.40245	-0.21559	1.45586
C	4.69505	-0.25994	2.01523
C	5.14320	-1.39630	2.68490
C	4.31036	-2.49758	2.80535
C	3.05243	-2.50121	2.22509
C	2.61398	-1.37287	1.53525

O	1.40885	-1.34547	0.87767
C	0.42780	-2.34006	1.15227
O	5.47205	0.84017	1.83869
C	6.81128	0.85827	2.29344
C	2.70219	-1.38652	-2.08280
C	2.78746	-2.76277	-1.40764
C	2.31101	-1.63123	-3.55158
C	4.07542	-0.70833	-2.01862
C	-2.42420	-0.45315	-0.09316
C	-3.08210	-1.42590	-0.85625
C	-2.70518	-1.75650	-2.28060
C	-4.11173	-2.20244	-0.29160
C	-4.44446	-2.10770	1.03457
C	-3.60528	-1.36464	1.89779
C	-3.74079	-1.43320	3.30373
C	-2.81733	-0.84886	4.13326
C	-1.70058	-0.19714	3.58430
C	-1.57941	-0.07138	2.22068
C	-2.54472	-0.59931	1.33647
C	-3.72670	1.45105	-1.59152
C	-3.46268	1.03484	-2.91246
C	-4.42812	1.07708	-3.88914
C	-5.72171	1.52793	-3.57950
C	-5.99309	2.01137	-2.32479
C	-4.99702	2.03147	-1.32216
C	-5.19716	2.68997	-0.08604
C	-4.12018	2.89745	0.73431
H	-0.77530	2.34167	1.01255
H	-1.65934	3.83767	1.30679
H	-1.87726	2.47475	2.40625
H	3.67435	2.27758	2.41400
H	2.73176	4.35983	1.55692
H	1.31034	4.35154	-0.50050
H	2.14806	1.35477	-3.17985
H	0.45901	0.85129	-3.44185
H	6.13227	-1.42671	3.12158
H	4.65353	-3.37300	3.34461
H	2.42760	-3.37963	2.30772
H	0.14643	-2.31691	2.20899
H	-0.43872	-2.08583	0.53870
H	0.77892	-3.34032	0.87997
H	7.21951	1.82537	1.99425
H	7.40176	0.05891	1.83025
H	6.86669	0.76613	3.38460
H	3.43776	-3.41848	-1.99059
H	3.20310	-2.68861	-0.40512
H	1.80124	-3.22423	-1.34940
H	2.97438	-2.37748	-3.99273
H	1.28713	-2.00290	-3.61831
H	2.39069	-0.72151	-4.14516
H	4.39296	-0.58280	-0.98387
H	4.05232	0.27285	-2.49301
H	4.81646	-1.32212	-2.53434
H	-1.79465	-1.23093	-2.58119
H	-3.50593	-1.51575	-2.98153
H	-2.50759	-2.82942	-2.35436
H	-4.65669	-2.88122	-0.94118
H	-5.28121	-2.66376	1.44222
H	-4.57620	-1.98946	3.71762
H	-2.92374	-0.91719	5.21089
H	-0.93284	0.20123	4.23918
H	-0.68902	0.38847	1.79978
H	-2.44709	0.73995	-3.16753
H	-4.19340	0.77196	-4.90327
H	-6.48854	1.52733	-4.34687
H	-6.97158	2.42030	-2.09336
H	-6.17087	3.10207	0.15385
H	-4.21903	3.52707	1.61351

L2

C	3.39900	0.38935	-0.89255
C	2.84248	0.99217	0.37523
C	1.74438	0.48246	1.07076
Pd	0.86535	-1.13127	0.08327
P	-0.34442	-1.01273	-1.89822
C	-2.15467	-1.21025	-2.08303
C	-3.21699	-0.39069	-1.70156
C	-4.52327	-0.78485	-1.99454
C	-4.78013	-1.97499	-2.65917
C	-3.73912	-2.81056	-3.03687
C	-2.44226	-2.42075	-2.73875
O	-1.38903	-3.19604	-3.09388
C	-0.17157	-2.80938	-2.46024
C	0.06985	-3.71245	-1.24573
C	-3.02791	0.89173	-0.96875
C	-3.06485	0.89822	0.42957
C	-2.97847	2.09995	1.13085
C	-2.84604	3.28855	0.43113
C	-2.83409	3.30761	-0.95553
C	-2.94925	2.10792	-1.65643
O	-3.01547	2.03299	-3.01649
C	-3.02786	3.21568	-3.79272
O	-3.19912	-0.31600	1.03540
C	-3.74608	-0.38408	2.34019

C	0.12127	-0.02490	-3.46795
C	1.46148	-0.52734	-4.02768
C	0.29228	1.43808	-3.02881
C	-0.93353	-0.11198	-4.57751
C	1.80131	-1.26928	1.90590
C	0.86768	-1.66924	2.86995
C	-0.55923	-2.04311	2.53403
C	1.24907	-1.85105	4.21365
C	2.54527	-1.68177	4.61886
C	3.55552	-1.54818	3.63965
C	4.92498	-1.59286	3.98752
C	5.89906	-1.62192	3.02402
C	5.53051	-1.66013	1.66968
C	4.20909	-1.56953	1.31090
C	3.18297	-1.43826	2.26958
C	1.15420	1.33964	2.06235
C	-0.11700	1.09236	2.62258
C	-0.62499	1.85565	3.64255
C	0.10729	2.94165	4.14848
C	1.30781	3.26693	3.57585
C	1.84625	2.49845	2.51772
C	3.04702	2.88147	1.88191
C	3.47877	2.17818	0.79202
H	3.24570	1.09153	-1.71621
H	2.89626	-0.54779	-1.14900
H	4.47171	0.20881	-0.81425
H	-5.34514	-0.14469	-1.69532
H	-5.80324	-2.25869	-2.87889
H	-3.91813	-3.74704	-3.54939
H	0.63179	-2.97040	-3.18797
H	0.98968	-3.40418	-0.73538
H	-0.76736	-3.63904	-0.55123
H	0.17305	-4.75122	-1.55862
H	-3.01128	2.11387	2.21124
H	-2.76398	4.22181	0.97691
H	-2.75070	4.25135	-1.47746
H	-2.09307	3.77850	-3.68353
H	-3.13377	2.89722	-4.83133
H	-3.87323	3.86023	-3.52456
H	-4.67440	0.19522	2.40806
H	-3.04235	-0.02401	3.09855
H	-3.96186	-1.43760	2.52746
H	1.33644	-1.47508	-4.55105
H	1.85732	0.20034	-4.73829
H	2.19712	-0.66464	-3.23553
H	-0.60946	1.80348	-2.54183
H	0.50161	2.07101	-3.89285
H	1.11868	1.53157	-2.32399
H	-0.60065	0.46320	-5.44354
H	-1.88592	0.29296	-4.24069
H	-1.08390	-1.14608	-4.88747
H	-0.87156	-1.67926	1.54793
H	-1.25737	-1.67264	3.28577
H	-0.63682	-3.13373	2.51951
H	0.47867	-2.09546	4.93834
H	2.81639	-1.72983	5.66742
H	5.18983	-1.63621	5.03904
H	6.94722	-1.66063	3.29896
H	6.29380	-1.77647	0.90807
H	3.93325	-1.67760	0.27005
H	-0.72192	0.30312	2.20024
H	-1.59865	1.62623	4.06241
H	-0.29452	3.52925	4.96632
H	1.86604	4.13122	3.92081
H	3.58634	3.75306	2.23512
H	4.35083	2.51285	0.23897

L3

C	-2.71371	1.85316	-2.44718
C	-3.14205	1.10015	-1.21471
C	-2.27293	0.72903	-0.19341
Pd	-0.21990	0.69082	-0.21082
P	0.61710	-1.18421	-1.28989
C	2.38006	-1.51975	-0.97396
C	3.11071	-1.47822	0.21102
C	4.45732	-1.85349	0.18277
C	5.06471	-2.24215	-1.00184
C	4.35822	-2.25239	-2.19809
C	3.02318	-1.88033	-2.16883
O	2.26623	-1.86027	-3.29487
C	1.03330	-1.15038	-3.13680
C	1.17433	0.25399	-3.74253
C	2.35618	1.03730	-3.17677
C	2.52471	-1.04353	1.50570
C	2.51850	-1.93648	2.59195
C	2.06093	-1.53271	3.84333
C	1.62816	-0.22701	4.02621
C	1.61840	0.67014	2.97174
C	2.04768	0.25337	1.71289
O	1.98103	1.10446	0.62082
C	2.37237	2.46442	0.81451
O	2.97010	-3.19117	2.32732
C	2.96485	-4.18215	3.33678
C	-0.10492	-2.94553	-1.12055

C	0.09422	-3.41516	0.32814
C	-1.60681	-2.84994	-1.42990
C	0.53174	-3.99535	-2.04206
C	-1.27323	2.36346	0.43427
C	-1.79954	2.74053	1.66957
C	-1.91657	1.83866	2.87217
C	-2.28334	4.04963	1.88268
C	-2.19830	5.02729	0.93204
C	-1.49241	4.76029	-0.25976
C	-1.21164	5.79135	-1.18683
C	-0.41832	5.56658	-2.28014
C	0.12907	4.28913	-2.48427
C	-0.16861	3.26757	-1.61989
C	-1.00759	3.44081	-0.49125
C	-2.77877	-0.17968	0.80198
C	-1.92609	-0.84652	1.71735
C	-2.39910	-1.73908	2.64471
C	-3.77562	-2.01254	2.71906
C	-4.63449	-1.40836	1.84005
C	-4.16361	-0.50881	0.85434
C	-5.03220	0.03582	-0.11560
C	-4.51269	0.77802	-1.14054
H	-3.38372	1.62496	-3.27741
H	-1.70165	1.57735	-2.74389
H	-2.74339	2.93118	-2.27697
H	5.03058	-1.83754	1.10225
H	6.10996	-2.53065	-0.99704
H	4.82617	-2.53805	-3.13138
H	0.27798	-1.68864	-3.72285
H	1.30213	0.14709	-4.82441
H	0.24077	0.79648	-3.56840
H	3.29493	0.54540	-3.43130
H	2.36842	2.04420	-3.59410
H	2.29057	1.11085	-2.09039
H	2.04833	-2.22067	4.67802
H	1.28537	0.09085	5.00410
H	1.27015	1.68339	3.12029
H	3.30786	2.51111	1.37975
H	1.59642	3.03575	1.33500
H	2.52207	2.88898	-0.17952
H	3.61305	-3.90291	4.17582
H	3.35098	-5.09093	2.87179
H	1.95022	-4.36952	3.70757
H	1.15330	-3.56423	0.53822
H	-0.30042	-2.68895	1.03666
H	-0.42497	-4.36206	0.48599
H	-1.76903	-2.49488	-2.44879
H	-2.07438	-3.83074	-1.32867
H	-2.09972	-2.16237	-0.74254
H	0.08740	-4.97031	-1.83215
H	1.60505	-4.06698	-1.86994
H	0.36089	-3.76704	-3.09297
H	-2.87043	1.30788	2.87281
H	-1.86647	2.43465	3.78514
H	-1.11047	1.10895	2.90626
H	-2.74746	4.27180	2.83883
H	-2.60775	6.01602	1.10633
H	-1.61817	6.77963	-0.99699
H	-0.19584	6.36661	-2.97744
H	0.78855	4.11414	-3.32733
H	0.27443	2.28848	-1.79290
H	-0.85600	-0.64450	1.67292
H	-1.71355	-2.23372	3.32415
H	-4.14592	-2.70885	3.46336
H	-5.69693	-1.62754	1.87272
H	-6.09311	-0.18284	-0.06768
H	-5.16687	1.13741	-1.92897

L4

C	-1.68025	-1.71285	1.63863
C	-1.75138	-0.26435	2.06702
C	-1.65369	0.80075	1.16858
Pd	-1.11946	0.08140	-0.69018
P	1.05782	-0.72062	-1.30144
C	2.63950	-1.55461	-0.90249
C	3.01765	-2.31152	0.20270
C	4.25220	-2.97093	0.17022
C	5.09560	-2.85039	-0.92300
C	4.76294	-2.03393	-1.99806
C	3.53943	-1.38511	-1.96366
O	3.13862	-0.55260	-2.96028
C	2.05485	0.29392	-2.55591
C	2.66739	1.58765	-1.97595
C	3.60911	2.22011	-3.00563
C	1.58882	2.58458	-1.56443
C	2.22132	-2.30379	1.44729
C	1.81293	-1.07664	1.99382
C	1.25171	-1.02169	3.26832
C	1.04718	-2.19662	3.97532
C	1.38300	-3.42759	3.43118
C	1.97916	-3.48084	2.17294
O	2.34795	-4.64152	1.56750
C	2.10145	-5.88494	2.19377
O	2.00102	0.03864	1.22483

C	2.00555	1.31283	1.85337
C	0.32704	-2.07037	-2.45846
C	-0.95570	-1.48500	-3.07046
C	-0.04017	-3.26375	-1.56090
C	1.20386	-2.59308	-3.60304
C	-2.97661	0.99903	-0.31479
C	-4.20792	0.60056	0.20348
C	-4.45560	-0.69013	0.93630
C	-5.34097	1.43745	0.09428
C	-5.30574	2.63881	-0.55298
C	-4.14366	2.99402	-1.27076
C	-4.12805	4.13479	-2.10666
C	-3.05597	4.40958	-2.91291
C	-1.95950	3.53234	-2.92068
C	-1.93773	2.44922	-2.08004
C	-3.00065	2.14962	-1.19081
C	-1.45797	2.11661	1.70752
C	-1.12630	3.23858	0.91306
C	-0.96893	4.49190	1.44563
C	-1.13896	4.70311	2.82340
C	-1.42477	3.63943	3.63699
C	-1.56925	2.33296	3.11392
C	-1.78811	1.23318	3.97264
C	-1.82361	-0.03301	3.45562
H	-1.96031	-1.83697	0.58970
H	-2.32408	-2.34549	2.25058
H	-0.65618	-2.07472	1.75168
H	4.55974	-3.56795	1.02000
H	6.04498	-3.37444	-0.92577
H	5.43322	-1.89688	-2.83682
H	1.48038	0.52892	-3.45996
H	3.25404	1.31085	-1.08998
H	4.39452	1.51759	-3.28168
H	4.07179	3.11464	-2.58803
H	3.05843	2.49877	-3.90548
H	0.90180	2.12177	-0.85488
H	1.02194	2.92119	-2.43310
H	2.03930	3.45556	-1.08803
H	0.95416	-0.07739	3.70282
H	0.60473	-2.15304	4.96406
H	1.19465	-4.33061	3.99613
H	2.64453	-5.97180	3.14254
H	1.03048	-6.03736	2.37330
H	2.46180	-6.64983	1.50338
H	0.99170	1.63043	2.11893
H	2.63400	1.30271	2.75020
H	2.42372	2.01573	1.13174
H	-1.62048	-1.09698	-2.27827
H	-0.72160	-0.66846	-3.75435
H	-1.50688	-2.24994	-3.61988
H	0.85416	-3.69389	-1.10846
H	-0.53766	-4.03828	-2.14736
H	-0.71548	-2.95579	-0.76235
H	0.65554	-3.36547	-4.14690
H	2.12460	-3.03509	-3.22494
H	1.45685	-1.79980	-4.30445
H	-5.52708	-0.87387	1.02275
H	-4.01341	-1.53331	0.40735
H	-4.04098	-0.65102	1.94591
H	-6.25966	1.11533	0.57434
H	-6.17204	3.29020	-0.57364
H	-5.00295	4.77676	-2.11738
H	-3.05664	5.27906	-3.56054
H	-1.13157	3.71078	-3.59809
H	-1.08589	1.77010	-2.13065
H	-0.97522	3.10491	-0.14896
H	-0.70919	5.32471	0.80138
H	-1.02789	5.70023	3.23455
H	-1.53279	3.77962	4.70776
H	-1.88650	1.40153	5.03923
H	-1.92176	-0.88819	4.11682

L5

C	0.24095	-4.25752	-1.45638
C	-0.83170	-3.39174	-2.06586
C	-1.11505	-2.07859	-1.69108
Pd	-0.12162	-0.56630	-0.60900
P	0.46519	1.15496	0.84088
C	2.28761	1.21892	0.92813
C	3.25362	1.29435	-0.07921
C	4.59802	1.11443	0.25336
C	4.98577	0.85696	1.56011
C	4.04638	0.82155	2.58003
C	2.71125	1.00968	2.25040
O	1.76576	1.01634	3.21569
C	0.43356	1.25874	2.75326
C	-0.52332	0.32009	3.54356
O	-0.14268	-1.03132	3.37679
C	0.72363	-1.64690	4.31125
C	-1.95002	0.41674	2.99340
C	-0.51796	0.77046	5.01345
C	2.94237	1.71934	-1.47213
C	3.40538	2.99003	-1.86815
C	3.18277	3.46386	-3.15808

C	2.48415	2.67770	-4.06372
C	2.01483	1.42865	-3.69851
C	2.25197	0.94979	-2.40891
O	1.80459	-0.29900	-2.03865
C	1.94144	-1.33455	-3.01722
O	4.05490	3.70576	-0.91152
C	4.56095	4.99467	-1.19919
C	-0.05385	2.95153	0.42057
C	-0.08618	3.13016	-1.10487
C	-1.47947	3.19114	0.94140
C	0.89640	4.00551	1.00571
C	-1.15720	-2.07133	0.34700
C	-0.24407	-2.85213	1.05512
C	1.24889	-2.62519	0.97597
C	-0.69005	-3.83341	1.96395
C	-2.02302	-4.03537	2.19629
C	-2.97507	-3.17609	1.60537
C	-4.34900	-3.29127	1.92106
C	-5.26927	-2.40970	1.41973
C	-4.83979	-1.35630	0.59589
C	-3.51696	-1.23916	0.25561
C	-2.53944	-2.15566	0.70836
C	-2.10270	-1.38587	-2.48739
C	-2.32471	0.01049	-2.37512
C	-3.24270	0.68182	-3.14062
C	-4.02605	-0.01451	-4.07532
C	-3.83516	-1.36026	-4.23796
C	-2.86750	-2.06180	-3.48086
C	-2.61652	-3.42815	-3.72429
C	-1.59647	-4.04455	-3.05902
H	-0.05159	-4.59110	-0.45832
H	1.19028	-3.73178	-1.37303
H	0.40520	-5.14350	-2.07106
H	5.34727	1.18881	-0.52618
H	6.03471	0.70698	1.78988
H	4.33212	0.65871	3.61142
H	0.16767	2.28419	3.05546
H	0.89630	-2.66006	3.94257
H	0.26793	-1.71693	5.30720
H	1.68509	-1.12739	4.38979
H	-1.95384	0.19480	1.92684
H	-2.57361	-0.32124	3.49615
H	-2.36837	1.40824	3.15719
H	0.49425	0.77579	5.41402
H	-1.14069	0.10864	5.61411
H	-0.92116	1.78065	5.08073
H	3.54051	4.43869	-3.46097
H	2.30054	3.04996	-5.06505
H	1.45380	0.82895	-4.40312
H	2.77582	-1.11528	-3.68894
H	2.15055	-2.26005	-2.47872
H	1.01988	-1.45089	-3.59617
H	5.03122	5.34772	-0.27965
H	3.75841	5.68619	-1.48215
H	5.31112	4.96157	-1.99810
H	-0.57420	4.07347	-1.35705
H	0.92041	3.15214	-1.51350
H	-0.63706	2.32224	-1.58545
H	-1.51260	3.18658	2.03024
H	-1.83953	4.16350	0.60027
H	-2.15869	2.42475	0.56587
H	1.91305	3.84868	0.64539
H	0.57118	5.00099	0.69775
H	0.90847	3.97613	2.09498
H	1.80464	-3.54534	1.16099
H	1.54030	-1.89619	1.73561
H	1.55449	-2.21767	0.00864
H	0.04903	-4.44722	2.46986
H	-2.35795	-4.82149	2.86421
H	-4.65945	-4.08941	2.58801
H	-6.32014	-2.50463	1.67015
H	-5.55912	-0.62967	0.23385
H	-3.21053	-0.40177	-0.35727
H	-1.72830	0.58244	-1.66396
H	-3.36692	1.75296	-3.02422
H	-4.76371	0.51748	-4.66582
H	-4.41555	-1.91327	-4.96967
H	-3.20792	-3.96149	-4.46021
H	-1.36615	-5.08193	-3.28176

L6

C	-4.55758	1.04302	-1.58799
C	-3.80050	-0.03045	-2.32798
C	-2.55272	-0.52630	-1.95538
Pd	-1.11324	0.10924	-0.59933
P	0.50029	0.40003	1.05385
C	1.83351	1.58368	0.65280
C	1.77611	2.88473	0.15104
C	2.96541	3.60043	-0.01196
C	4.19006	3.02852	0.30130
C	4.26715	1.72753	0.77983
C	3.08515	1.02111	0.94719
O	3.08554	-0.24491	1.42863
C	1.84086	-0.92279	1.25141

C	1.94887	-1.82950	0.00895
C	2.98863	-2.88645	0.22946
C	4.25596	-2.74107	-0.16404
C	5.33998	-3.69769	0.04254
C	5.14335	-4.95410	0.62713
C	6.20496	-5.82607	0.80206
C	7.48457	-5.46573	0.39889
C	7.69480	-4.22302	-0.18383
C	6.63431	-3.34950	-0.36119
C	0.48862	3.53133	-0.20730
C	-0.35252	3.00566	-1.18772
C	-1.55128	3.62276	-1.53113
C	-1.92589	4.78413	-0.87755
C	-1.11039	5.33609	0.10145
C	0.09414	4.72038	0.43236
O	0.94003	5.18932	1.38738
C	0.61180	6.34771	2.13044
O	-0.03297	1.80801	-1.81765
C	0.94265	1.87156	-2.85468
C	0.17013	0.84761	2.88111
C	-0.44630	2.25343	2.93325
C	-0.83832	-0.17750	3.42154
C	1.41960	0.84555	3.77258
C	-2.69120	-1.08480	-0.01545
C	-2.78854	-2.47261	0.01800
C	-1.86169	-3.40125	-0.72290
C	-3.80824	-3.10345	0.76034
C	-4.69964	-2.39829	1.52082
C	-4.55748	-0.99925	1.63555
C	-5.37458	-0.25056	2.51580
C	-5.18233	1.09316	2.69455
C	-4.15462	1.74594	1.99251
C	-3.37013	1.04458	1.11388
C	-3.54258	-0.34416	0.88027
C	-1.88492	-1.36178	-2.92628
C	-0.49942	-1.65235	-2.84460
C	0.15375	-2.41196	-3.78031
C	-0.55268	-2.95095	-4.86846
C	-1.88549	-2.67036	-5.00963
C	-2.56655	-1.85282	-4.07713
C	-3.90428	-1.46186	-4.29546
C	-4.46581	-0.53034	-3.46844
H	-5.15951	0.61518	-0.78401
H	-3.88451	1.78492	-1.16248
H	-5.23333	1.55788	-2.27286
H	2.92408	4.61619	-0.38690
H	5.09932	3.60339	0.16570
H	5.21586	1.26663	1.02313
H	1.68976	-1.55487	2.13415
H	2.20150	-1.21008	-0.85626
H	0.97439	-2.29308	-0.15921
H	2.65396	-3.76054	0.77925
H	4.53895	-1.83043	-0.68759
H	4.15288	-5.25885	0.94635
H	6.03369	-6.79614	1.25623
H	8.31308	-6.15111	0.53753
H	8.69073	-3.93438	-0.50208
H	6.80745	-2.37998	-0.81691
H	-2.17325	3.18009	-2.29831
H	-2.86144	5.26975	-1.12954
H	-1.42096	6.24389	0.60158
H	0.52656	7.22828	1.48284
H	-0.32301	6.21362	2.68729
H	1.43148	6.49820	2.83516
H	0.61715	2.56196	-3.64099
H	1.03554	0.86576	-3.26607
H	1.91105	2.19988	-2.45918
H	0.29539	3.00294	2.65799
H	-0.79474	2.46958	3.94466
H	-1.29405	2.33877	2.25450
H	-1.76083	-0.15363	2.84141
H	-0.42669	-1.18676	3.37437
H	-1.08052	0.04680	4.46164
H	2.17008	1.53883	3.39399
H	1.86391	-0.14643	3.83923
H	1.14152	1.16039	4.78024
H	-2.21420	-3.56824	-1.74296
H	-0.85139	-2.99759	-0.77239
H	-1.81604	-4.36794	-0.21931
H	-3.87846	-4.18603	0.71703
H	-5.48620	-2.90353	2.07014
H	-6.15217	-0.77152	3.06523
H	-5.80743	1.65550	3.37932
H	-3.98569	2.80606	2.14697
H	-2.57968	1.56995	0.57730
H	0.07763	-1.23485	-2.02044
H	1.21819	-2.59664	-3.68368
H	-0.03550	-3.56706	-5.59559
H	-2.43958	-3.05147	-5.86155
H	-4.44849	-1.85622	-5.14636
H	-5.46481	-0.16073	-3.67910

L7

C	-4.13690	0.10760	-1.02180

C	-3.56100	-0.25330	0.31810
C	-2.26590	0.05930	0.73420
Pd	-0.69000	0.79690	-0.35200
P	0.70310	-0.73470	-1.43940
C	2.47880	-0.55100	-1.75340
C	3.53940	-0.60780	-0.86220
C	4.83560	-0.58630	-1.37710
C	5.05280	-0.47740	-2.73810
C	3.99700	-0.31010	-3.62150
C	2.70780	-0.32480	-3.11890
O	1.62050	-0.09610	-3.89320
C	0.48800	0.18730	-3.06660
C	0.54180	1.65090	-2.57270
C	-0.78640	-0.09910	-3.84290
C	3.33390	-0.52360	0.59520
C	3.94200	-1.44890	1.45580
C	3.91510	-1.24700	2.82970
C	3.29640	-0.12000	3.34130
C	2.66220	0.78530	2.51390
C	2.65170	0.57540	1.13800
O	1.99890	1.40590	0.26270
C	1.82200	2.76260	0.64210
O	4.52670	-2.51740	0.84340
C	5.13340	-3.50280	1.64180
C	0.47360	-2.59270	-1.65350
C	1.15790	-3.17010	-2.88790
C	1.07270	-3.20860	-0.38590
C	-1.03510	-2.84530	-1.67760
C	-2.02890	1.96130	0.65510
C	-1.93700	2.44510	1.96070
C	-1.00410	1.90200	3.00800
C	-2.76810	3.49210	2.39910
C	-3.63760	4.13030	1.56690
C	-3.62250	3.82360	0.19380
C	-4.37460	4.58710	-0.72270
C	-4.27790	4.37930	-2.06880
C	-3.41630	3.38440	-2.54600
C	-2.71380	2.59860	-1.67310
C	-2.79670	2.76100	-0.27000
C	-1.76880	-0.69290	1.86010
C	-0.38770	-0.86200	2.11200
C	0.07550	-1.60370	3.16550
C	-0.82070	-2.20150	4.05990
C	-2.16310	-2.10210	3.82840
C	-2.66240	-1.40020	2.71250
C	-4.03060	-1.43710	2.38550
C	-4.43060	-0.94910	1.17730
H	-4.87940	-0.63750	-1.30370
H	-3.36150	0.11360	-1.78550
H	-4.62870	1.07990	-1.00010
H	5.67320	-0.63530	-0.69860
H	6.06360	-0.47560	-3.11840
H	4.16070	-0.15740	-4.67640
H	1.58390	1.94470	-2.43600
H	0.02830	1.73100	-1.57620
H	0.08520	2.33710	-3.28810
H	-0.94830	0.66110	-4.60630
H	-0.71920	-1.06520	-4.33730
H	-1.63600	-0.11280	-3.15750
H	4.38000	-1.94740	3.50430
H	3.29570	0.04670	4.40780
H	2.16740	1.64040	2.94450
H	1.69950	3.32570	-0.28520
H	0.92610	2.89780	1.25920
H	2.70020	3.13940	1.17350
H	5.51590	-4.24810	0.94620

L8

C	-3.05698	1.40331	-2.49284
C	-3.24814	0.51179	-1.29799
C	-2.29589	0.38253	-0.28278
Pd	-0.27739	0.84795	-0.37635
P	0.95713	-0.67989	-1.59951
C	2.77022	-0.54527	-1.41838
C	3.56030	-0.39216	-0.28128
C	4.95168	-0.42084	-0.42168
C	5.54003	-0.58859	-1.66589
C	4.76517	-0.71184	-2.81299
C	3.38702	-0.68026	-2.67336
O	2.55687	-0.78794	-3.74284
C	1.23286	-0.31081	-3.44924
C	1.25310	1.21527	-3.63646
C	0.26217	-0.91327	-4.45860
C	2.98553	-0.18336	1.07224
C	3.31392	-1.07220	2.11253
C	2.84662	-0.85750	3.40607
C	2.06361	0.25561	3.67957
C	1.72365	1.14349	2.67376
C	2.17317	0.91119	1.37546
O	1.79553	1.75859	0.34164
C	2.01287	3.15460	0.54436
O	4.08857	-2.13302	1.76214
C	4.43757	-3.11377	2.71995
C	0.78422	-2.57980	-1.45343

C	1.66467	-3.40191	-2.40412
C	1.18860	-2.96786	-0.02069
C	-0.69414	-2.93835	-1.66571
C	-1.79122	2.07226	0.39520
C	-1.95225	3.22732	-0.39400
C	-1.03438	3.55055	-1.55093
C	-2.90066	4.21287	-0.06361
C	-3.67836	4.10957	1.05905
C	-3.35932	3.12399	2.02076
C	-3.97590	3.12572	3.29292
C	-3.55537	2.28000	4.28646
C	-2.46300	1.43267	4.04958
C	-1.88215	1.38555	2.80633
C	-2.34415	2.16796	1.72890
C	-2.52764	-0.67065	0.68097
C	-1.50414	-1.15342	1.52686
C	-1.72019	-2.14523	2.45168
C	-2.99827	-2.70951	2.58787
C	-4.00757	-2.30820	1.75180
C	-3.79185	-1.31792	0.76558
C	-4.79033	-0.99864	-0.18188
C	-4.48476	-0.15658	-1.21631
H	-3.70120	1.08041	-3.31231
H	-2.02338	1.37665	-2.84156
H	-3.31726	2.43644	-2.24475
H	5.57506	-0.30959	0.45768
H	6.62110	-0.61166	-1.74714
H	5.21371	-0.82899	-3.79110
H	1.86024	1.69253	-2.86718
H	0.24045	1.61062	-3.59149
H	1.68044	1.45127	-4.61121
H	0.50027	-0.54260	-5.45662
H	0.32847	-1.99771	-4.47658
H	-0.75769	-0.62336	-4.20960
H	3.09238	-1.54475	4.20455
H	1.70957	0.42566	4.68972
H	1.10529	2.00568	2.88639
H	1.96166	3.62649	-0.43815
H	1.24795	3.58954	1.19651
H	3.00343	3.32099	0.97858
H	5.03407	-3.85759	2.18876
H	3.54748	-3.59737	3.13929
H	5.03586	-2.68506	3.53259
H	2.71818	-3.16806	-2.25433
H	1.51849	-4.46390	-2.19631
H	1.41923	-3.22568	-3.44855
H	2.26089	-2.83723	0.12139
H	0.94439	-4.01609	0.15993
H	0.66540	-2.36514	0.71964
H	-1.02119	-2.68087	-2.67282
H	-1.32378	-2.40482	-0.95287
H	-0.84383	-4.00969	-1.52034
H	-0.46763	2.67085	-1.87005
H	-1.57199	3.96043	-2.40743
H	-0.31198	4.30396	-1.22161
H	-3.02875	5.05228	-0.73989
H	-4.46968	4.82228	1.26209
H	-4.77293	3.83842	3.47907
H	-4.02950	2.29196	5.26159
H	-2.06768	0.82308	4.85494
H	-1.00353	0.77061	2.66943
H	-0.50158	-0.74066	1.41619
H	-0.90517	-2.49416	3.07640
H	-3.17185	-3.47632	3.33485
H	-4.98986	-2.76520	1.81817
H	-5.76548	-1.46753	-0.11301
H	-5.21911	0.02984	-1.99365

L9

C	-2.79433	-0.15771	3.06302
C	-2.93162	1.25745	2.56200
C	-2.34984	1.75803	1.39979
Pd	-0.84326	1.06777	0.13584
P	0.46137	0.40469	-1.67274
C	2.25131	0.23265	-1.36065
C	2.93659	-0.38818	-0.31722
C	4.33150	-0.46165	-0.37374
C	5.03207	0.09856	-1.43165
C	4.36760	0.75711	-2.45757
C	2.98284	0.81652	-2.40674
O	2.26694	1.42582	-3.38105
C	0.89746	1.65306	-3.02954
C	0.74971	3.10954	-2.59059
C	2.24808	-0.94459	0.87365
C	2.42266	-2.29045	1.23235
C	1.86078	-2.80387	2.39898
C	1.11901	-1.97894	3.23016
C	0.92620	-0.64678	2.89917
C	1.48088	-0.14646	1.72716
O	1.19295	1.17596	1.39726
C	2.16716	2.14683	1.49091
C	1.80534	3.44028	1.12795
C	2.74473	4.45608	1.18755
C	4.04030	4.18819	1.61136

C	4.38668	2.89947	1.99109
C	3.45451	1.87242	1.93819
O	3.20381	-3.05182	0.40957
C	3.00275	-4.39947	0.23207
C	4.12869	-5.17321	-0.03688
C	3.98256	-6.52905	-0.28379
C	2.72247	-7.11309	-0.26472
C	1.60348	-6.33297	-0.00669
C	1.73449	-4.97443	0.23978
C	0.14148	-1.15759	-2.72586
C	-1.32965	-1.11111	-3.16618
C	1.03623	-1.29143	-3.96592
C	0.37291	-2.39367	-1.84538
C	-2.80148	0.48319	-0.13043
C	-3.78650	1.06150	-0.92497
C	-3.68561	2.44284	-1.51828
C	-4.96883	0.35456	-1.22737
C	-5.17191	-0.93546	-0.82099
C	-4.13380	-1.62282	-0.15734
C	-4.24403	-3.00062	0.14670
C	-3.20346	-3.69053	0.70903
C	-1.99900	-3.02217	0.98661
C	-1.87983	-1.68161	0.72301
C	-2.93829	-0.91882	0.16771
C	-2.43943	3.18448	1.20247
C	-1.67682	3.85665	0.21446
C	-1.70513	5.21772	0.05306
C	-2.52862	6.00666	0.87334
C	-3.27745	5.40600	1.84952
C	-3.23189	4.00678	2.05424
C	-3.92499	3.40866	3.12694
C	-3.73219	2.08124	3.38377
H	-1.81165	-0.56835	2.84334
H	-2.93280	-0.18278	4.14514
H	-3.55047	-0.80497	2.61442
H	4.86819	-0.95954	0.42493
H	6.11392	0.03074	-1.45187
H	4.90281	1.21080	-3.28173
H	0.30683	1.50168	-3.94085
H	1.25878	3.28090	-1.64237
H	1.19479	3.75814	-3.34519
H	-0.30421	3.36041	-2.48806
H	2.02456	-3.84167	2.66049
H	0.69413	-2.37637	4.14409
H	0.35788	0.01710	3.53714
H	0.78744	3.63971	0.81673
H	2.46193	5.46292	0.90218
H	4.77508	4.98342	1.65288
H	5.39255	2.68524	2.33451
H	3.72729	0.87040	2.24410
H	5.10327	-4.70097	-0.05014
H	4.85829	-7.13378	-0.49082
H	2.61234	-8.17405	-0.45587
H	0.61676	-6.78242	-0.00187
H	0.85940	-4.36415	0.42576
H	-1.99079	-1.08279	-2.29983
H	-1.52295	-0.22814	-3.77720
H	-1.57287	-1.99706	-3.75494
H	2.08962	-1.30133	-3.68804
H	0.86929	-0.48102	-4.67371
H	0.80833	-2.23240	-4.47039
H	0.10951	-3.29812	-2.39633
H	-0.23934	-2.35324	-0.94581
H	1.42060	-2.46170	-1.55302
H	-4.34088	2.52726	-2.38631
H	-2.66692	2.65564	-1.84331
H	-3.98581	3.20322	-0.79509
H	-5.73726	0.86334	-1.80118
H	-6.09849	-1.45165	-1.04586
H	-5.17255	-3.50842	-0.09376
H	-3.29545	-4.74803	0.93045
H	-1.16442	-3.56846	1.41331
H	-0.93748	-1.18208	0.94250
H	-1.03858	3.26502	-0.43935
H	-1.09388	5.68791	-0.70955
H	-2.55866	7.08149	0.73360
H	-3.90624	6.00142	2.50379
H	-4.55986	4.01543	3.76291
H	-4.20958	1.62913	4.24779

L10

C	-1.96761	-3.38948	-1.58177
C	-2.44767	-2.07644	-2.14856
C	-2.62802	-0.91429	-1.40573
Pd	-1.95007	-0.32537	0.48343
P	-0.01262	0.91861	1.12309
C	1.55390	0.12353	1.67560
C	2.82281	-0.02419	1.10151
C	3.72051	-0.93872	1.66951
C	3.38470	-1.68517	2.78607
C	2.15405	-1.51084	3.40341
C	1.26726	-0.60427	2.84426
O	0.06161	-0.38827	3.42139
C	-0.54794	0.83142	2.96348

C	-2.04575	0.69181	3.20203
C	3.25570	0.75111	-0.08005
C	2.39629	0.92537	-1.16676
C	2.79341	1.61003	-2.31343
C	4.07251	2.13545	-2.37380
C	4.94149	2.00866	-1.29878
C	4.54121	1.32979	-0.15004
O	5.31159	1.21370	0.95855
C	6.62542	1.76045	1.06280
C	7.63717	0.83040	0.39061
C	6.89730	1.89487	2.55922
O	1.10459	0.45705	-1.03893
C	0.71082	-0.73371	-1.74047
C	0.44587	-0.45463	-3.21665
C	1.71906	-1.86033	-1.53838
C	0.26464	2.81207	0.97269
C	1.53875	3.27533	1.68899
C	-0.95027	3.52118	1.59730
C	0.31719	3.24827	-0.50051
C	-3.61324	-1.50003	0.31796
C	-4.95142	-1.17894	0.13599
C	-5.44466	0.14101	-0.39969
C	-5.95632	-2.12027	0.44803
C	-5.66680	-3.34505	0.98091
C	-4.33281	-3.66175	1.31504
C	-4.01427	-4.86378	1.99040
C	-2.73451	-5.13044	2.39658
C	-1.71638	-4.19556	2.14071
C	-1.99449	-3.04145	1.45746
C	-3.30276	-2.72936	0.99869
C	-2.96071	0.28948	-2.11990
C	-2.98221	1.56503	-1.49875
C	-3.25003	2.71648	-2.19068
C	-3.53304	2.66382	-3.56661
C	-3.51552	1.45729	-4.21257
C	-3.21426	0.25911	-3.52220
C	-3.12879	-0.97153	-4.20697
C	-2.72562	-2.09073	-3.53300
H	-2.79471	-3.94359	-1.13338
H	-1.20282	-3.24257	-0.82075
H	-1.54065	-4.00515	-2.37479
H	4.69278	-1.07945	1.21442
H	4.09508	-2.39814	3.18934
H	1.87789	-2.05973	4.29450
H	-0.15800	1.64517	3.59794
H	-2.45359	-0.09942	2.55653
H	-2.56273	1.62473	2.98652

H	-2.23344	0.41229	4.23905
H	2.10038	1.74556	-3.13220
H	4.39669	2.66718	-3.26104
H	5.92648	2.45084	-1.36570
H	6.65792	2.75790	0.59527
H	7.41004	0.72389	-0.66908
H	7.59805	-0.15446	0.85531
H	8.64226	1.23613	0.49820
H	7.88641	2.31930	2.72488
H	6.14762	2.54496	3.00833
H	6.84125	0.91677	3.03606
H	-0.23905	-1.00635	-1.25147
H	-0.09130	-1.29787	-3.64983
H	1.37613	-0.31526	-3.76573
H	-0.16787	0.43955	-3.31628
H	2.67758	-1.60809	-1.99226
H	1.86961	-2.04216	-0.47504
H	1.33789	-2.76941	-2.00142
H	1.61921	4.36280	1.63483
H	1.52539	2.98344	2.73942
H	2.42303	2.84233	1.22303
H	-1.88097	3.13210	1.18209
H	-0.97335	3.40853	2.68000
H	-0.90376	4.58950	1.37777
H	1.28454	3.02545	-0.94301
H	-0.45644	2.74698	-1.08283
H	0.15909	4.32617	-0.57413
H	-6.49173	0.28631	-0.13119
H	-4.87281	0.97193	0.01130
H	-5.36841	0.17349	-1.48828
H	-6.98888	-1.85332	0.24584
H	-6.45431	-4.06075	1.18979
H	-4.81546	-5.56546	2.19945
H	-2.50160	-6.04873	2.92412
H	-0.70829	-4.39060	2.48940
H	-1.18422	-2.33026	1.28283
H	-2.76479	1.62973	-0.43167
H	-3.24515	3.67252	-1.67905
H	-3.75445	3.57776	-4.10640
H	-3.71769	1.40046	-5.27732
H	-3.35108	-1.01044	-5.26747
H	-2.61785	-3.02934	-4.06743

L11

C	-4.55239	-2.64741	-0.42729
C	-4.26339	-1.43979	-1.28312
C	-3.07155	-0.72549	-1.28864

Pd	-1.48478	-0.58901	0.01037
P	0.17430	-0.26383	1.64582
C	1.82844	0.47655	1.85910
C	2.47314	1.47672	1.13462
C	3.69677	1.96856	1.60094
C	4.27679	1.46130	2.75357
C	3.66091	0.44485	3.47134
C	2.44056	-0.03233	3.01506
O	1.77185	-0.99196	3.69283
C	0.62895	-1.51393	3.00454
C	0.92681	-2.94645	2.52257
C	1.47405	-3.78247	3.68330
C	1.88072	-2.97075	1.33067
C	1.96082	1.98201	-0.15919
C	1.76156	1.09573	-1.22260
C	1.48623	1.57905	-2.50102
C	1.37054	2.94426	-2.70491
C	1.50200	3.83899	-1.65045
C	1.80114	3.36121	-0.37728
O	1.94848	4.14490	0.72178
C	1.72976	5.55469	0.69988
C	1.37066	5.94219	2.13244
C	2.99294	6.27338	0.22263
O	1.84080	-0.24991	-0.95700
C	2.92415	-1.02405	-1.49697
C	2.50724	-1.72569	-2.78872
C	4.18952	-0.18776	-1.67785
C	-0.84125	0.98702	2.67947
C	-0.27545	1.32898	4.06299
C	-2.25318	0.40268	2.84075
C	-0.93778	2.28432	1.86181
C	-1.53203	-2.16386	-1.32268
C	-1.10617	-2.22862	-2.64149
C	-0.94323	-1.02858	-3.53794
C	-0.82779	-3.47691	-3.24071
C	-0.95196	-4.65471	-2.55793
C	-1.34154	-4.63820	-1.20253
C	-1.44844	-5.83795	-0.45945
C	-1.80856	-5.82655	0.86091
C	-2.06282	-4.59993	1.49902
C	-1.96155	-3.42521	0.79876
C	-1.62224	-3.38776	-0.57864
C	-3.04548	0.50457	-2.03861
C	-1.97652	1.43448	-1.94112
C	-1.99030	2.63871	-2.59361
C	-3.06530	2.98310	-3.42995
C	-4.11557	2.11601	-3.56322
C	-4.14524	0.88884	-2.85991
C	-5.27516	0.04779	-2.92506
C	-5.33212	-1.05694	-2.12509
H	-5.62643	-2.83630	-0.39788
H	-4.06767	-3.53796	-0.83032
H	-4.21295	-2.49882	0.59639
H	4.19528	2.75584	1.04796
H	5.22694	1.85733	3.09442
H	4.10416	0.03353	4.36913
H	-0.17744	-1.58353	3.74765
H	-0.02818	-3.37997	2.20432
H	2.42855	-3.38203	4.02481
H	1.62142	-4.81394	3.36239
H	0.77351	-3.77354	4.51907
H	2.08228	-3.99816	1.02690
H	1.42078	-2.44862	0.49028
H	2.82548	-2.48672	1.57936
H	1.37155	0.89332	-3.32850
H	1.17062	3.32266	-3.70087
H	1.38845	4.89798	-1.83900
H	0.88394	5.79353	0.03498
H	0.47719	5.40141	2.44226
H	2.18739	5.67975	2.80417
H	1.18203	7.01284	2.19665
H	3.24547	5.96239	-0.78999
H	3.82693	6.02859	0.87980
H	2.83391	7.35087	0.23299
H	3.12442	-1.79223	-0.73658
H	3.27570	-2.44144	-3.07973
H	2.36788	-1.01194	-3.59898
H	1.57247	-2.25906	-2.62265
H	4.44238	0.31311	-0.74334
H	4.05458	0.56397	-2.45517
H	5.01380	-0.84105	-1.96131
H	-0.22332	0.44892	4.70259
H	0.72267	1.75788	3.97901
H	-0.92241	2.06303	4.54739
H	-2.23271	-0.52726	3.41008
H	-2.90307	1.11024	3.35852
H	-2.69242	0.19488	1.85554
H	0.04188	2.75088	1.76318
H	-1.33001	2.08336	0.86216
H	-1.60746	2.99130	2.35498
H	-0.29646	-1.26741	-4.38309
H	-1.91088	-0.71231	-3.93305
H	-0.50648	-0.19177	-2.99804
H	-0.51521	-3.48896	-4.28024

H	-0.74600	-5.60273	-3.04238
H	-1.23651	-6.77554	-0.96316
H	-1.89233	-6.75124	1.42065
H	-2.33851	-4.58640	2.54765
H	-2.15298	-2.48363	1.31392
H	-1.11717	1.19317	-1.31264
H	-1.16662	3.33227	-2.46843
H	-3.05694	3.93116	-3.95599
H	-4.96079	2.36709	-4.19598
H	-6.10260	0.31016	-3.57490
H	-6.22542	-1.67374	-2.12915

L12

C	-3.33177	-2.39636	1.58064
C	-3.90934	-1.28046	0.74897
C	-3.17050	-0.17946	0.32183
Pd	-1.13244	0.06373	0.40390
P	0.00680	-0.88942	-1.37824
C	1.23860	0.39226	-1.81767
C	2.35113	0.90605	-1.15550
C	3.04304	1.98316	-1.72039
C	2.58243	2.59964	-2.87359
C	1.42004	2.15976	-3.49670
C	0.76949	1.06029	-2.96279
O	-0.38000	0.58098	-3.50650
C	-0.58872	-0.81260	-3.20313
C	-2.07934	-1.10303	-3.34305
C	0.18830	-1.57941	-4.28114
C	2.81253	0.36980	0.14940
C	4.06333	-0.26878	0.23234
C	4.51872	-0.77145	1.44871
C	3.72624	-0.65110	2.58301
C	2.49527	-0.02130	2.52340
C	2.05012	0.50712	1.31137
O	0.79820	1.09601	1.26561
C	0.62460	2.48484	1.63442
C	0.72120	3.36443	0.38978
C	1.59977	2.93435	2.71471
O	4.73300	-0.37653	-0.94355
C	6.03270	-0.95802	-1.04372
C	7.09371	0.04753	-0.59317
C	6.20505	-1.33409	-2.51312
C	0.90436	-2.56180	-1.14814
C	-0.08915	-3.68370	-1.49337
C	2.21026	-2.73074	-1.93532
C	1.23046	-2.71231	0.34695
C	-2.40200	0.71727	1.90725
C	-3.08095	1.92186	2.10024
C	-3.21777	3.00956	1.06455
C	-3.71729	2.20547	3.32759
C	-3.63514	1.36906	4.40535
C	-2.76996	0.25581	4.33992
C	-2.48186	-0.51246	5.49223
C	-1.53051	-1.49756	5.46854
C	-0.82440	-1.74717	4.28021
C	-1.12815	-1.04712	3.14074
C	-2.12744	-0.04480	3.10500
C	-3.76768	0.65096	-0.69267
C	-3.00371	1.55675	-1.47137
C	-3.56540	2.32581	-2.45848
C	-4.94470	2.24496	-2.71384
C	-5.71357	1.35427	-2.01247
C	-5.14609	0.51663	-1.02339
C	-5.90333	-0.49841	-0.39851
C	-5.27189	-1.40290	0.41142
H	-2.26906	-2.52867	1.37455
H	-3.45826	-2.19873	2.64695
H	-3.84048	-3.33389	1.35020
H	3.94085	2.34523	-1.23308
H	3.12560	3.44280	-3.28539
H	1.02740	2.65089	-4.37772
H	-2.41092	-0.85373	-4.35135
H	-2.65025	-0.50773	-2.63430
H	-2.27303	-2.15801	-3.15496
H	0.04782	-2.65402	-4.20139
H	-0.18148	-1.25526	-5.25517
H	1.24857	-1.33982	-4.22768
H	5.47597	-1.26978	1.52325
H	4.07756	-1.06076	3.52306
H	1.86321	0.05300	3.39887
H	-0.39996	2.53274	2.03142
H	0.38128	4.37186	0.62648
H	1.74661	3.41124	0.02540
H	0.08829	2.95343	-0.39646
H	1.39132	3.97447	2.96257
H	1.47928	2.33213	3.61384
H	2.62995	2.85558	2.36829
H	6.08695	-1.86885	-0.42540
H	7.03262	0.94714	-1.20500
H	8.08627	-0.38866	-0.69721
H	6.93599	0.32388	0.44834
H	6.11978	-0.44454	-3.13650
H	7.18014	-1.79224	-2.67191
H	5.42652	-2.03879	-2.80272

H	-1.01915	-3.54878	-0.93862
H	-0.32337	-3.70916	-2.55559
H	0.33592	-4.65069	-1.21837
H	2.83380	-1.84159	-1.85008
H	2.02392	-2.92507	-2.98902
H	2.76875	-3.57960	-1.53543
H	0.32982	-2.59480	0.94909
H	1.96406	-1.97668	0.66553
H	1.63869	-3.70636	0.53828
H	-2.38230	3.00907	0.36756
H	-4.14589	2.89971	0.50038
H	-3.24148	3.98286	1.55836
H	-4.29945	3.11920	3.40030
H	-4.16449	1.58733	5.32612
H	-3.01094	-0.28200	6.41153
H	-1.30361	-2.06981	6.36136
H	-0.03815	-2.49416	4.26687
H	-0.55584	-1.24686	2.23673
H	-1.92913	1.61903	-1.29878
H	-2.94598	2.99299	-3.04707
H	-5.38718	2.87158	-3.48043
H	-6.77308	1.25585	-2.22646
H	-6.96128	-0.58960	-0.61790
H	-5.82842	-2.24071	0.82036

L13

C	-3.47183	-0.51839	-2.67454
C	-3.98559	-0.44167	-1.25247
C	-3.20898	-0.06730	-0.15528
Pd	-1.20378	0.13917	-0.63705
P	0.05770	-1.84215	-0.59306
C	1.65106	-1.70652	-1.49651
C	2.59948	-0.67713	-1.55313
C	3.57615	-0.69389	-2.55131
C	3.66321	-1.74393	-3.45490
C	2.76848	-2.80001	-3.38646
C	1.76129	-2.75127	-2.43148
O	0.83786	-3.73350	-2.37649
C	-0.31557	-3.41853	-1.59710
C	-1.50528	-3.28558	-2.54182
C	2.68139	0.40093	-0.53155
C	2.26921	1.71307	-0.79327
C	2.38642	2.70107	0.19179
C	1.94093	4.11215	-0.07699
C	2.91655	2.35834	1.42890
C	3.38590	1.07720	1.69828
C	3.96592	0.75256	3.04904
C	3.29989	0.11045	0.69349
O	3.82018	-1.15058	0.91319
C	5.08114	-1.46067	0.29949
C	6.22938	-1.10040	1.24329
C	5.08117	-2.94916	-0.03536
O	1.85627	2.07274	-2.05293
C	0.53602	1.80391	-2.51714
C	-0.21839	3.12453	-2.66809
C	0.63377	1.06384	-3.84891
C	0.40908	-2.66058	1.09670
C	0.78530	-1.60057	2.14005
C	-0.89577	-3.32552	1.57099
C	1.53656	-3.69552	1.02332
C	-2.40039	1.77511	-0.07339
C	-3.30724	2.62851	-0.69762
C	-3.89049	2.40844	-2.06655
C	-3.79206	3.78343	-0.04329
C	-3.37747	4.14372	1.20586
C	-2.31950	3.42785	1.80606
C	-1.74547	3.86951	3.02079
C	-0.63914	3.25747	3.54654
C	-0.04754	2.19270	2.84877
C	-0.60922	1.73170	1.68556
C	-1.79542	2.28374	1.13923
C	-3.73980	-0.32522	1.15255
C	-2.96024	-0.24531	2.32792
C	-3.48600	-0.47398	3.57304
C	-4.84408	-0.79862	3.71856
C	-5.62775	-0.93018	2.60307
C	-5.09930	-0.72498	1.30753
C	-5.88958	-0.95528	0.15996
C	-5.31864	-0.87103	-1.07875
H	-4.10059	0.04004	-3.36829
H	-2.44252	-0.15518	-2.75752
H	-3.49416	-1.55836	-3.00363
H	4.27732	0.13024	-2.61121
H	4.43602	-1.73887	-4.21499
H	2.81871	-3.63573	-4.07246
H	-0.49915	-4.28143	-0.94481
H	-1.35541	-2.45003	-3.22401
H	-1.60740	-4.19901	-3.12817
H	-2.41351	-3.13292	-1.96173
H	2.08343	4.36574	-1.12698
H	2.50045	4.81671	0.53849
H	0.88036	4.21959	0.16195
H	2.99270	3.11902	2.19892
H	3.32699	1.15097	3.83859

H	4.05507	-0.32487	3.18044
H	4.95542	1.20074	3.15913
H	5.18341	-0.87814	-0.62948
H	6.10696	-1.61606	2.19505
H	7.17961	-1.39448	0.79848
H	6.24304	-0.02632	1.42164
H	6.02750	-3.22549	-0.49934
H	4.94365	-3.53785	0.87088
H	4.27104	-3.17168	-0.72834
H	0.00420	1.15463	-1.77183
H	-1.15458	2.96396	-3.20047
H	0.39293	3.82883	-3.23121
H	-0.43809	3.54972	-1.69028
H	1.19615	0.14109	-3.71961
H	-0.36242	0.82630	-4.22009
H	1.15022	1.68716	-4.57833
H	1.10633	-2.08886	3.06164
H	1.59583	-0.96952	1.78746
H	-0.07070	-0.97032	2.37341
H	-1.72936	-2.62308	1.51303
H	-1.14310	-4.20647	0.97944
H	-0.78650	-3.63890	2.61040
H	1.34083	-4.45956	0.27194
H	2.47458	-3.20027	0.78138
H	1.64591	-4.18726	1.99144
H	-4.75328	1.74096	-2.01344
H	-4.22861	3.35734	-2.48446
H	-3.15473	1.98595	-2.74743
H	-4.54560	4.37701	-0.55123
H	-3.80629	4.99991	1.71435
H	-2.18985	4.72524	3.51892
H	-0.20186	3.60335	4.47639
H	0.86071	1.73908	3.22870
H	-0.09892	0.93585	1.14234
H	-1.90724	-0.01546	2.24176
H	-2.85062	-0.40957	4.44950
H	-5.25710	-0.96298	4.70744
H	-6.67107	-1.21451	2.69417
H	-6.92797	-1.24477	0.27640
H	-5.89454	-1.12614	-1.96284

L14

C	2.25533	-2.54536	-2.80820
C	3.19019	-2.20830	-1.67496
C	2.85758	-1.38379	-0.59956
Pd	1.06120	-0.62948	0.10532
P	-0.75223	0.66143	0.61993
C	-2.23001	-0.39249	0.69240
C	-2.48065	-1.45375	1.56534
C	-3.65773	-2.18647	1.42539
C	-4.55560	-1.88204	0.41279
C	-4.29796	-0.86107	-0.48878
C	-3.12139	-0.12955	-0.36169
N	-2.76172	0.94822	-1.18875
C	-1.55735	1.66127	-0.75180
C	-3.29799	1.10124	-2.45663
C	-3.29085	2.49628	-3.11769
C	-4.08419	2.35439	-4.42801
C	-1.90644	3.06510	-3.46366
C	-4.04786	3.45455	-2.18260
O	-3.88511	0.18557	-2.99564
C	-1.55539	-1.81488	2.67265
C	-0.29765	-2.38128	2.45500
C	0.51745	-2.75396	3.52276
C	0.06899	-2.56906	4.81928
C	-1.17866	-2.01367	5.06820
C	-1.98827	-1.63707	3.99921
O	-3.21545	-1.07058	4.14649
C	-3.74751	-0.84034	5.43681
O	0.12682	-2.54575	1.15017
C	0.79532	-3.76978	0.83230
C	-0.87509	1.86602	2.09065
C	-2.32186	2.13348	2.51953
C	-0.09165	1.27000	3.26966
C	-0.18411	3.18228	1.69650
C	2.17315	0.32851	-1.33283
C	3.15084	1.30600	-1.16232
C	3.80870	1.60428	0.16010
C	3.58398	2.08249	-2.25531
C	3.01982	1.96920	-3.49671
C	1.89680	1.13213	-3.67360
C	1.17683	1.12180	-4.89189
C	0.01579	0.40605	-5.02539
C	-0.47446	-0.33250	-3.93397
C	0.23259	-0.37162	-2.75960
C	1.45309	0.33070	-2.58290
C	3.77502	-1.39612	0.51863
C	3.40598	-0.91468	1.80114
C	4.25001	-0.97200	2.88004
C	5.54312	-1.50246	2.73710
C	5.93451	-2.00544	1.52511
C	5.06073	-2.00057	0.41239
C	5.41521	-2.64472	-0.79216
C	4.47159	-2.78938	-1.77095

H	1.22339	-2.60786	-2.46641
H	2.52728	-3.51146	-3.23628
H	2.31521	-1.79886	-3.60261
H	-3.86708	-2.99521	2.11571
H	-5.47240	-2.45332	0.31985
H	-5.01086	-0.64785	-1.27216
H	-0.86069	1.72382	-1.58927
H	-1.81375	2.67787	-0.44465
H	-4.16941	3.32197	-4.92253
H	-5.08485	1.97271	-4.22856
H	-3.57830	1.65828	-5.09643
H	-2.02061	3.85244	-4.21034
H	-1.26611	2.29041	-3.88369
H	-1.41554	3.50685	-2.59847
H	-4.14672	4.43146	-2.65658
H	-5.04479	3.06568	-1.97401
H	-3.52345	3.58284	-1.23633
H	1.49796	-3.17246	3.33716
H	0.70091	-2.85618	5.65189
H	-1.50806	-1.87641	6.08941
H	-3.11810	-0.15073	6.01184
H	-4.72899	-0.38807	5.28402
H	-3.86461	-1.77688	5.99457
H	0.31262	-4.60698	1.34449
H	1.85440	-3.72864	1.10782
H	0.70935	-3.90104	-0.24754
H	-2.79520	1.21253	2.86063
H	-2.33948	2.85538	3.33800
H	-2.90674	2.53716	1.69251
H	-0.54875	0.34555	3.61549
H	0.93797	1.05913	2.97660
H	-0.07446	1.97571	4.10206
H	0.80660	2.98557	1.28312
H	-0.76390	3.73637	0.95895
H	-0.06809	3.81616	2.57721
H	4.66733	0.95107	0.32860
H	4.16241	2.63620	0.17696
H	3.10724	1.47350	0.98477
H	4.39451	2.78672	-2.09489
H	3.38547	2.55764	-4.33076
H	1.55169	1.71872	-5.71715
H	-0.53440	0.41458	-5.95957
H	-1.41478	-0.86408	-4.02179
H	-0.16857	-0.95092	-1.92713
H	2.40499	-0.50566	1.94828
H	3.92084	-0.60899	3.84761
H	6.21339	-1.52567	3.58919
H	6.91877	-2.44700	1.40567
H	6.40524	-3.07302	-0.90177
H	4.70759	-3.36185	-2.66277

L15

C	2.69747	-2.24295	2.76833
C	3.78788	-1.43377	2.09602
C	3.54583	-0.49357	1.09281
Pd	1.49663	-0.37961	0.91483
P	-0.86135	-0.24393	0.92159
C	-1.93697	0.73928	-0.20415
C	-2.89963	0.37394	-1.15266
C	-3.38735	1.34883	-2.03156
C	-2.94951	2.65936	-1.96319
C	-2.03163	3.04912	-0.99796
C	-1.53294	2.08584	-0.13489
N	-0.64610	2.37167	0.92971
C	-0.77183	1.34765	1.96541
C	0.27656	3.37975	0.85299
C	1.08975	3.77395	2.10354
C	2.26382	2.80394	2.31859
C	0.20379	3.88633	3.35678
C	1.67500	5.16818	1.81340
O	0.43816	3.99596	-0.18712
C	-3.42952	-1.00054	-1.25796
C	-2.57200	-2.10098	-1.19084
C	-3.04160	-3.40447	-1.33511
C	-4.39372	-3.61532	-1.54690
C	-5.27771	-2.54676	-1.58113
C	-4.80870	-1.24337	-1.42961
O	-5.62319	-0.15822	-1.42916
C	-7.02364	-0.24752	-1.16202
C	-7.58433	1.14122	-1.45497
C	-7.25551	-0.65090	0.29546
O	-1.24725	-1.86320	-0.88097
C	-0.24596	-1.96424	-1.91039
C	-0.01163	-3.40636	-2.34898
C	-0.56872	-1.05136	-3.08886
C	-1.88859	-1.28768	2.15624
C	-1.78237	-2.78802	1.84070
C	-1.24969	-1.08782	3.54439
C	-3.36336	-0.87463	2.21365
C	2.69630	-0.97610	-0.69921
C	3.19191	-2.22731	-1.04767
C	3.25291	-3.39861	-0.10332
C	3.68143	-2.46313	-2.35163
C	3.64808	-1.50437	-3.32497

C	3.02129	-0.26635	-3.06217
C	2.83860	0.69402	-4.08621
C	2.14107	1.85066	-3.85950
C	1.58754	2.08991	-2.59052
C	1.78610	1.18974	-1.57721
C	2.52466	-0.00904	-1.75337
C	4.61563	0.40417	0.75631
C	4.44769	1.55999	-0.04124
C	5.49221	2.38432	-0.37170
C	6.79090	2.10197	0.07910
C	6.99468	1.02114	0.89439
C	5.92668	0.17348	1.27035
C	6.14318	-0.87901	2.18696
C	5.08164	-1.61500	2.63033
H	3.02417	-3.26574	2.96292
H	2.45526	-1.78244	3.72982
H	1.77799	-2.27833	2.17326
H	-4.11002	1.06919	-2.78763
H	-3.33941	3.39322	-2.65937
H	-1.72003	4.08138	-0.92688
H	-1.66652	1.56532	2.55926
H	0.08933	1.35482	2.62582
H	2.67341	2.92251	3.32190
H	3.05316	3.01406	1.59760
H	1.97012	1.75685	2.18939
H	-0.77690	4.28275	3.09266
H	0.66858	4.56793	4.07031
H	0.07053	2.93031	3.85921
H	0.87142	5.89094	1.67320
H	2.27743	5.14482	0.90619
H	2.30259	5.49105	2.64406
H	-2.35904	-4.23952	-1.25871
H	-4.77040	-4.62460	-1.66681
H	-6.33253	-2.73734	-1.72929
H	-7.49474	-0.98100	-1.83671
H	-7.10643	1.87785	-0.80976
H	-7.38449	1.40437	-2.49282
H	-8.65944	1.15583	-1.28214
H	-6.78375	0.07377	0.95873
H	-8.32361	-0.68496	0.50554
H	-6.82552	-1.63267	0.48996
H	0.65962	-1.60127	-1.40597
H	-0.82872	-3.77135	-2.97016
H	0.91225	-3.45077	-2.92562
H	0.09072	-4.04790	-1.47484
H	-1.49547	-1.35715	-3.57421
H	0.24394	-1.10027	-3.81249
H	-0.67039	-0.02245	-2.74630
H	-2.43285	-3.06660	1.01594
H	-0.75699	-3.05587	1.58266
H	-2.08261	-3.37355	2.71196
H	-1.65429	-1.82033	4.24509
H	-0.16816	-1.22854	3.49518
H	-1.45397	-0.09747	3.94813
H	-3.85780	-1.07579	1.26422
H	-3.46454	0.18822	2.43505
H	-3.87625	-1.43899	2.99493
H	3.44552	-4.32032	-0.65334
H	4.05306	-3.26778	0.62858
H	2.31000	-3.51503	0.43176
H	4.10893	-3.43658	-2.57002
H	4.05755	-1.69786	-4.31017
H	3.24918	0.48224	-5.06831
H	1.99850	2.57366	-4.65484
H	1.00539	2.98518	-2.40691
H	1.33545	1.38817	-0.60495
H	3.46094	1.82289	-0.39198
H	5.31240	3.26358	-0.98030
H	7.61461	2.74881	-0.20116
H	7.98391	0.80491	1.28506
H	7.14400	-1.06042	2.56316
H	5.22228	-2.37202	3.39541

L16

C	-3.76637	1.80029	0.20295
C	-3.66622	0.38188	-0.28455
C	-2.60519	-0.46225	0.04070
Pd	-0.75207	0.03991	0.79611
P	0.67307	0.87263	-0.82528
C	2.43312	0.86969	-0.36368
C	3.18069	-0.15839	0.20760
C	4.56078	0.00815	0.35241
C	5.17783	1.17593	-0.07322
C	4.44153	2.22267	-0.60692
C	3.05834	2.08290	-0.71453
N	2.20780	3.02766	-1.25703
C	0.77636	2.73152	-1.07712
C	0.23989	3.50260	0.13303
C	2.55969	4.38939	-1.39693
C	3.31418	5.05084	-0.42706
C	3.63492	6.38887	-0.59758
C	3.19910	7.07922	-1.72084
C	2.43271	6.42535	-2.67695
C	2.11286	5.08616	-2.51983

C	2.57688	-1.42634	0.69368
C	3.01627	-2.65408	0.16675
C	2.54760	-3.85926	0.68296
C	1.65857	-3.85114	1.74867
C	1.20582	-2.65658	2.28251
C	1.64941	-1.45286	1.73817
O	1.15428	-0.24110	2.19942
C	1.06985	-0.06282	3.61234
O	3.90094	-2.57385	-0.86284
C	4.36461	-3.74758	-1.50142
C	0.76499	0.25535	-2.63226
C	1.31224	-1.17907	-2.63794
C	-0.67017	0.25930	-3.18093
C	1.65811	1.09860	-3.55305
C	-2.38752	-0.50085	1.96244
C	-2.78975	0.58661	2.75389
C	-2.02205	1.88947	2.79828
C	-3.87070	0.48346	3.65078
C	-4.55360	-0.69101	3.81309
C	-4.03466	-1.86973	3.23158
C	-4.58856	-3.13283	3.54315
C	-4.00337	-4.29210	3.10576
C	-2.80889	-4.22365	2.37295
C	-2.27990	-3.00564	2.02573
C	-2.89618	-1.78634	2.37778
C	-2.53934	-1.71879	-0.66789
C	-1.36882	-2.51302	-0.68730
C	-1.30253	-3.71003	-1.35599
C	-2.42682	-4.19122	-2.04564
C	-3.56768	-3.43375	-2.09089
C	-3.64221	-2.18024	-1.43976
C	-4.77359	-1.34702	-1.58432
C	-4.74352	-0.08189	-1.06547
H	-4.41925	2.38216	-0.44870
H	-2.78694	2.27936	0.21953
H	-4.18506	1.82738	1.21227
H	5.15148	-0.78958	0.78667
H	6.25400	1.27549	0.01795
H	4.93793	3.12295	-0.94674
H	0.22774	3.06088	-1.96598
H	0.39852	4.57323	0.00125
H	-0.82933	3.32253	0.23274
H	0.74281	3.18537	1.04680
H	3.63012	4.52593	0.46647
H	4.22027	6.89925	0.15939
H	3.45106	8.12567	-1.84801
H	2.08603	6.96104	-3.55375
H	1.52320	4.57344	-3.27096
H	2.87826	-4.80398	0.27256
H	1.30796	-4.79045	2.16032
H	0.50346	-2.65541	3.10541
H	0.19232	-0.56833	4.02953
H	1.97591	-0.44105	4.09522
H	0.98452	1.01107	3.78766
H	4.92035	-4.38897	-0.80716
H	3.53695	-4.31761	-1.93989
H	5.03375	-3.41604	-2.29742
H	2.35310	-1.19069	-2.31535
H	0.73662	-1.82390	-1.97609
H	1.25948	-1.59261	-3.64664
H	-1.31189	-0.39093	-2.58590
H	-1.08812	1.26707	-3.16270
H	-0.67967	-0.09869	-4.21180
H	1.27262	2.11045	-3.67034
H	2.67501	1.15537	-3.16640
H	1.69374	0.63498	-4.54094
H	-1.32157	1.97277	1.96232
H	-2.68220	2.75787	2.79625
H	-1.43609	1.92080	3.72133
H	-4.17382	1.37116	4.19751
H	-5.43519	-0.74562	4.44187
H	-5.47746	-3.16674	4.16502
H	-4.43249	-5.25683	3.35279
H	-2.29687	-5.13679	2.08904
H	-1.33239	-2.98358	1.50615
H	-0.48143	-2.14592	-0.17268
H	-0.38319	-4.28552	-1.35124
H	-2.37772	-5.14747	-2.55474
H	-4.43241	-3.77739	-2.64948
H	-5.62976	-1.69860	-2.14895
H	-5.57718	0.59142	-1.23971

L17

C	-1.79985	-0.55767	4.07702
C	-2.10338	-1.87870	3.40705
C	-1.63763	-2.20458	2.12556
Pd	-0.66606	-0.60568	1.21693
P	-0.22072	1.38280	0.10537
C	1.52869	1.84781	-0.12219
C	2.59737	1.05245	-0.52647
C	3.86319	1.63397	-0.66105
C	4.06650	2.96748	-0.33794
C	3.02095	3.76030	0.11243
C	1.74627	3.20433	0.19119

N	0.62132	3.87600	0.64570	H	-1.05362	-0.72080	4.85871
C	-0.45683	2.98178	1.07724	H	-1.38438	0.16101	3.36600
C	-0.32733	2.71873	2.58135	H	4.69385	1.02580	-0.99916
C	0.38701	5.24054	0.46957	H	5.06151	3.39156	-0.41667
C	-0.53436	5.91034	1.28286	H	3.19972	4.78476	0.41374
C	-0.78223	7.26045	1.09436	H	-1.42117	3.45981	0.87769
C	-0.12316	7.96929	0.09933	H	-1.21920	2.20839	2.94058
C	0.78080	7.30605	-0.72074	H	-0.21955	3.65602	3.12596
C	1.03265	5.95593	-0.54691	H	0.54649	2.10045	2.78987
C	2.49116	-0.41114	-0.75471	H	-1.05530	5.38378	2.07227
C	2.84535	-0.95533	-2.00615	H	-1.49643	7.76380	1.73698
C	2.91580	-2.33632	-2.16926	H	-0.31716	9.02605	-0.04089
C	2.66053	-3.17879	-1.09495	H	1.28820	7.84320	-1.51470
C	2.28783	-2.66463	0.13426	H	1.71366	5.45038	-1.22024
C	2.17730	-1.28417	0.28744	H	3.18818	-2.77466	-3.11792
O	1.70966	-0.77290	1.49047	H	2.74211	-4.25112	-1.22918
C	2.58144	-0.89835	2.63114	H	2.07199	-3.31634	0.97079
C	3.46318	0.34574	2.72372	H	3.21076	-1.79109	2.49030
C	1.71017	-1.07488	3.86628	H	4.10392	0.28366	3.60229
O	3.09343	-0.03032	-2.96908	H	4.08517	0.42963	1.83343
C	3.34080	-0.28124	-4.34970	H	2.83981	1.23648	2.79729
C	4.68684	-0.97146	-4.58546	H	1.02945	-1.91282	3.71740
C	2.17206	-0.99083	-5.03651	H	1.12798	-0.17087	4.04334
C	-0.95231	1.83703	-1.60048	H	2.33197	-1.27072	4.73889
C	-0.32362	0.92026	-2.65978	H	3.40966	0.73471	-4.76996
C	-2.46530	1.58079	-1.53401	H	5.45288	-0.48374	-3.98365
C	-0.71432	3.28863	-2.03647	H	4.95442	-0.88056	-5.63776
C	-2.45737	-1.36595	0.55585	H	4.65913	-2.02805	-4.32696
C	-3.74659	-0.94846	0.87844	H	2.31495	-0.94917	-6.11584
C	-4.04867	-0.00997	2.01413	H	1.24118	-0.48396	-4.78542
C	-4.86105	-1.42748	0.16064	H	2.09114	-2.03405	-4.73829
C	-4.72726	-2.26018	-0.91610	H	-0.49713	-0.12826	-2.42352
C	-3.43503	-2.54215	-1.41209	H	0.75111	1.09101	-2.72376
C	-3.25272	-3.24879	-2.62368	H	-0.76509	1.12584	-3.63660
C	-2.00707	-3.41101	-3.17136	H	-2.92952	2.20526	-0.76913
C	-0.89063	-2.85132	-2.53020	H	-2.92716	1.81292	-2.49511
C	-1.04319	-2.19809	-1.33183	H	-2.67029	0.53670	-1.29857
C	-2.30218	-2.04922	-0.70553	H	-1.12614	3.43458	-3.03709
C	-1.61465	-3.61268	1.81607	H	0.35074	3.51502	-2.07257
C	-0.81379	-4.15323	0.78834	H	-1.20324	3.99369	-1.36569
C	-0.84091	-5.48475	0.45802	H	-3.22990	0.69324	2.17025
C	-1.68487	-6.36639	1.15076	H	-4.95195	0.56270	1.79892
C	-2.42000	-5.89973	2.20873	H	-4.21785	-0.56911	2.93719
C	-2.37467	-4.53829	2.58860	H	-5.85063	-1.13011	0.49374
C	-3.02261	-4.09195	3.76194	H	-5.59706	-2.65470	-1.42906
C	-2.80038	-2.81385	4.19658	H	-4.12907	-3.64318	-3.12799
H	-2.67915	-0.12133	4.55221	H	-1.87949	-3.94667	-4.10563

H	0.09245	-2.93578	-2.98115
H	-0.17060	-1.75433	-0.85684
H	-0.12186	-3.50361	0.27132
H	-0.20221	-5.85977	-0.33458
H	-1.72299	-7.41277	0.86853
H	-3.03336	-6.57824	2.79310
H	-3.62468	-4.78437	4.33965
H	-3.18580	-2.49044	5.15876

L18

C	-0.28459	-2.69771	-2.39933
C	-1.64960	-2.12196	-2.09920
C	-2.64564	-2.34259	-3.06769
C	-3.88553	-1.78192	-2.96683
C	-4.13312	-0.82570	-1.96198
C	-3.11717	-0.54317	-1.00829
C	-3.33065	0.54716	-0.14479
C	-4.50096	1.25526	-0.14388
C	-5.53662	0.90446	-1.01763
C	-5.34360	-0.10579	-1.91775
C	-1.91710	-1.32903	-0.97943
Pd	-0.19773	-0.86629	0.05929
P	1.17003	0.88277	-0.71429
C	1.69874	0.93268	-2.52653
C	2.49856	-0.34621	-2.77808
C	0.39252	0.92942	-3.32337
C	2.55597	2.13543	-2.91117
C	2.75471	1.10322	0.12722
C	3.64113	0.12520	0.55872
C	3.21845	-1.28558	0.46773
C	3.91581	-2.22144	-0.31051
O	5.02825	-1.74949	-0.93772
C	5.76284	-2.62474	-1.75796
C	3.44757	-3.52692	-0.40129
C	2.29739	-3.90222	0.27463
C	1.60069	-2.99806	1.05660
C	2.06152	-1.68332	1.14542
O	1.42944	-0.76037	1.97116
C	1.52117	-1.05606	3.35042
C	4.85257	0.52384	1.11117
C	5.14692	1.87161	1.24137
C	4.24307	2.85165	0.86131
C	3.03343	2.46009	0.30804
O	2.08548	3.33995	-0.08776
C	0.84441	2.69894	-0.35610
C	-0.10018	2.82323	0.84997
C	-0.57475	4.23390	1.02538
C	0.17417	5.15818	1.74134
C	-0.25522	6.46828	1.85845
C	-1.43909	6.86808	1.26222
C	-2.19127	5.95243	0.54577
C	-1.75992	4.64379	0.42609
C	-1.76765	-2.06054	0.79515
C	-2.13509	-3.40515	0.73694
C	-1.58244	-4.39613	-0.24350
C	-3.13459	-3.91546	1.58916
C	-3.75984	-3.15269	2.52714
C	-3.28734	-1.84958	2.77139
C	-2.25316	-1.32654	1.94824
C	-1.67989	-0.10098	2.37062
C	-2.15148	0.59553	3.45179
C	-3.24333	0.11513	4.18359
C	-3.78671	-1.09267	3.85051
H	0.39401	-2.57334	-1.54012
H	0.13349	-2.16432	-3.25563
H	-0.33641	-3.75007	-2.67620
H	-2.41067	-2.99388	-3.89928
H	-4.66230	-2.01315	-3.68092
H	-2.52992	0.85212	0.51194
H	-4.62624	2.09093	0.53019
H	-6.46857	1.45062	-0.99421
H	-6.11414	-0.35900	-2.63286
H	2.60396	-0.52768	-3.84722
H	3.49324	-0.25872	-2.34403
H	2.00220	-1.20413	-2.32436
H	-0.12638	1.88117	-3.21450
H	0.58554	0.76755	-4.38290
H	-0.27459	0.14443	-2.96184
H	2.02701	3.07640	-2.78112
H	3.47186	2.16961	-2.32323
H	2.83398	2.05138	-3.96160
H	5.15670	-3.00004	-2.58859
H	6.58702	-2.03272	-2.15282
H	6.16557	-3.46869	-1.18886
H	3.96812	-4.25972	-0.99611
H	1.94849	-4.92117	0.19484
H	0.72681	-3.30219	1.62161
H	0.99288	-0.25971	3.87252
H	1.05434	-2.01457	3.59813
H	2.57074	-1.07150	3.66677
H	5.55756	-0.22245	1.44390
H	6.09391	2.16819	1.66760
H	4.46355	3.89987	0.98641
H	0.38969	3.20781	-1.21389

H	-0.95633	2.17287	0.65033
H	0.42961	2.47396	1.74161
H	1.10007	4.84896	2.20468
H	0.33489	7.18010	2.41757
H	-1.77538	7.89035	1.35601
H	-3.11655	6.25845	0.07925
H	-2.34864	3.92941	-0.13328
H	-1.73804	-5.40701	0.13009
H	-0.51856	-4.24820	-0.40038
H	-2.09852	-4.30665	-1.20008
H	-3.43202	-4.94649	1.45342
H	-4.57081	-3.54765	3.12142
H	-0.80085	0.28444	1.84615
H	-1.68057	1.52472	3.74350
H	-3.62958	0.68582	5.01576
H	-4.60068	-1.50800	4.42863

L19

C	-3.53691	-3.08074	-1.12184
C	-3.18233	-2.52671	0.22586
C	-3.78404	-3.14327	1.33907
C	-3.53546	-2.73592	2.61586
C	-2.52454	-1.78408	2.85327
C	-1.87900	-1.18029	1.74130
C	-0.72310	-0.40697	2.00312
C	-0.30501	-0.16084	3.28359
C	-1.01989	-0.66707	4.37572
C	-2.09957	-1.47572	4.16002
C	-2.34107	-1.43455	0.40366
Pd	-1.04875	-0.50099	-0.87761
P	0.18085	0.90295	-2.30716
C	0.13577	2.72639	-1.83337
C	0.59189	2.79480	-0.37724
C	1.00886	3.61004	-2.71769
C	-1.33203	3.15248	-1.91351
C	1.89708	0.58240	-2.81841
C	2.96263	0.08983	-2.07504
C	2.81240	-0.24369	-0.63717
C	3.22670	0.56896	0.41948
O	3.75247	1.83118	0.28662
C	4.54996	2.19462	-0.81237
C	3.04308	0.12763	1.74077
C	3.43955	0.96892	2.93430
C	4.96141	1.12359	3.00044
C	2.75761	2.33869	2.95568
C	2.55842	-1.15000	1.95443
C	2.21028	-2.01507	0.92473
C	1.86984	-3.46163	1.19976
C	2.95471	-4.10697	2.06648
C	0.50015	-3.61785	1.86083
C	2.28741	-1.52014	-0.37417
O	1.93812	-2.28325	-1.47646
C	0.55579	-2.55608	-1.61968
C	4.15796	-0.19792	-2.73096
C	4.30935	0.06909	-4.07918
C	3.25839	0.57530	-4.82595
C	2.04478	0.79080	-4.19626
O	0.94662	1.19120	-4.88162
C	-0.22809	0.94597	-4.13189
C	-3.02297	0.07527	-0.62487
C	-3.74749	-0.15058	-1.79719
C	-3.13363	-0.69462	-3.06679
C	-5.10112	0.22605	-1.87656
C	-5.74136	0.82974	-0.83417
C	-4.99517	1.25931	0.28102
C	-3.60772	0.95523	0.34524
C	-2.85638	1.58601	1.35495
C	-3.44436	2.36166	2.31622
C	-4.83052	2.55459	2.31708
C	-5.58246	2.03122	1.30303
H	-2.68314	-3.04209	-1.79981
H	-4.36349	-2.51681	-1.55526
H	-3.84932	-4.11892	-1.02502
H	-4.48596	-3.94500	1.15485
H	-4.05739	-3.17570	3.45285
H	-0.10933	-0.03868	1.16537
H	0.58603	0.42091	3.45872
H	-0.69453	-0.43914	5.38059
H	-2.63244	-1.91808	4.99008
H	0.41857	3.78798	0.03396
H	1.64895	2.56533	-0.28109
H	0.02192	2.07445	0.21616
H	0.70621	3.55749	-3.76158
H	2.05648	3.31979	-2.65026
H	0.92421	4.64642	-2.39307
H	-1.46758	4.12153	-1.43647
H	-1.67146	3.23669	-2.94419
H	-1.96593	2.42655	-1.39688
H	3.96890	2.32863	-1.72862
H	5.34441	1.46726	-0.99583
H	4.99681	3.15073	-0.53496
H	3.12365	0.42174	3.83057
H	5.44210	0.14731	3.00136
H	5.32275	1.69040	2.14781

H	5.24348	1.65041	3.90937
H	1.68152	2.23848	2.83511
H	3.13230	2.96601	2.15438
H	2.95460	2.83153	3.90568
H	2.47154	-1.50045	2.97151
H	1.86583	-3.99207	0.24272
H	2.94681	-3.69963	3.07382
H	2.78326	-5.17916	2.13360
H	3.93826	-3.94052	1.63248
H	-0.29191	-3.23095	1.22266
H	0.29604	-4.66838	2.05686
H	0.46471	-3.07782	2.80397
H	0.31585	-2.44054	-2.68639
H	0.32255	-3.58521	-1.31990
H	-0.06323	-1.85290	-0.99148
H	4.96959	-0.63970	-2.17216
H	5.25013	-0.14371	-4.56404
H	3.35334	0.76523	-5.88286
H	-0.62413	-0.03830	-4.42820
H	-0.96905	1.70618	-4.38722
H	-2.17522	-1.20153	-2.87914
H	-2.95134	0.14897	-3.73818
H	-3.80740	-1.38030	-3.57859
H	-5.64206	-0.00265	-2.78516
H	-6.80007	1.03944	-0.87567
H	-1.78129	1.49112	1.33921
H	-2.83625	2.83482	3.07348
H	-5.29160	3.14345	3.09672
H	-6.64581	2.22042	1.25274

L20

C	4.11990	-0.48765	1.59823
C	3.91973	0.75760	0.77382
C	4.74243	1.85129	1.09928
C	4.56070	3.08333	0.54299
C	3.40295	3.32039	-0.22587
C	2.55948	2.22403	-0.55148
C	1.30374	2.51642	-1.11784
C	0.94081	3.79857	-1.43827
C	1.82577	4.86042	-1.21729
C	3.02684	4.62233	-0.60897
C	2.94792	0.88240	-0.21931
Pd	1.69466	-0.65244	-0.58596
P	0.61298	-2.07908	1.36450
C	-0.80638	-2.93484	2.31140
C	-1.69133	-1.84411	2.91716
C	-1.64763	-3.84806	1.42639
C	-0.16275	-3.71059	3.46306
C	0.05598	-2.00963	-0.37338
C	-0.97187	-1.28393	-1.00104
C	-1.95851	-0.46719	-0.26123
C	-3.32120	-0.70625	-0.48360
O	-3.62010	-1.79157	-1.26780
C	-4.67526	-2.64389	-0.87438
C	-4.27791	0.11348	0.12451
C	-5.72656	-0.00296	-0.12640
C	-6.21498	0.00317	-1.43053
C	-7.57562	-0.06018	-1.66871
C	-8.46509	-0.13053	-0.60956
C	-7.98803	-0.12806	0.69054
C	-6.62826	-0.05858	0.93175
C	-3.83133	1.15452	0.92527
C	-2.48759	1.41563	1.16767
C	-2.18784	2.65599	1.91239
C	-2.73264	2.87824	3.17315
C	-2.51799	4.07804	3.82622
C	-1.76773	5.07456	3.22409
C	-1.23540	4.86748	1.96290
C	-1.44725	3.66829	1.30806
C	-1.53500	0.55697	0.60594
O	-0.19401	0.56265	0.85472
C	0.34705	1.27438	1.94854
C	-1.10219	-1.37858	-2.38750
C	-0.20732	-2.11475	-3.15058
C	0.83989	-2.79679	-2.55568
C	0.96534	-2.74695	-1.16727
O	1.86617	-3.54553	-0.52686
C	1.62650	-3.59075	0.86784
C	3.23222	-0.14199	-1.83631
C	2.94156	0.50428	-3.03570
C	1.54720	0.82320	-3.50559
C	3.98295	0.83773	-3.92214
C	5.27365	0.45790	-3.69957
C	5.54298	-0.46546	-2.66863
C	4.49498	-0.81954	-1.77686
C	4.70828	-1.91895	-0.92520
C	5.91136	-2.57248	-0.88249
C	6.97974	-2.13535	-1.67398
C	6.79044	-1.10896	-2.55773
H	3.30100	-1.19637	1.43064
H	5.07453	-0.96525	1.38420
H	4.11603	-0.20874	2.65348
H	5.54662	1.68664	1.80353
H	5.24051	3.89717	0.74806

H	0.59184	1.70463	-1.23979
H	-0.03210	3.99958	-1.86704
H	1.54219	5.86410	-1.50042
H	3.69939	5.43795	-0.38181
H	-1.08841	-1.13510	3.48346
H	-2.23593	-1.30322	2.14806
H	-2.41838	-2.28892	3.59646
H	-2.11460	-3.28232	0.62247
H	-1.05195	-4.64292	0.98072
H	-2.43420	-4.31310	2.02019
H	-0.93313	-4.08023	4.13863
H	0.41170	-4.56488	3.11366
H	0.50224	-3.05934	4.02998
H	-5.59868	-2.41286	-1.41202
H	-4.86541	-2.58406	0.19958
H	-4.35786	-3.65683	-1.12997
H	-5.52041	0.06730	-2.25601
H	-7.94355	-0.05293	-2.68446
H	-9.52728	-0.18343	-0.79688
H	-8.67879	-0.17973	1.51953
H	-6.25591	-0.06067	1.94652
H	-4.56439	1.82759	1.34592
H	-3.31663	2.09990	3.64424
H	-2.94067	4.23792	4.80745
H	-1.60446	6.01201	3.73444
H	-0.65567	5.64169	1.48236
H	-1.04731	3.51702	0.31647
H	-0.30632	1.22001	2.82190
H	0.53459	2.32059	1.69559
H	1.30185	0.78614	2.16337
H	-1.90698	-0.84617	-2.87055
H	-0.32442	-2.15286	-4.22286
H	1.54198	-3.38548	-3.12603
H	1.10791	-4.53220	1.09645
H	2.59629	-3.60369	1.37907
H	1.41273	0.38392	-4.49568
H	0.79634	0.38413	-2.84094
H	1.38527	1.89657	-3.59388
H	3.73116	1.42490	-4.79483
H	6.07301	0.77772	-4.35173
H	3.86686	-2.29422	-0.35478
H	6.03986	-3.43484	-0.24321
H	7.93587	-2.63499	-1.61020
H	7.58722	-0.79980	-3.22008

L21

C	-1.94417	0.71869	-3.77619
C	-2.17014	-0.63717	-3.16798
C	-2.75693	-1.59869	-4.01373
C	-2.95956	-2.88883	-3.62226
C	-2.46655	-3.31684	-2.37564
C	-1.88018	-2.35612	-1.50652
C	-1.25788	-2.84878	-0.32991
C	-1.26974	-4.17732	-0.00745
C	-1.91952	-5.10187	-0.83776
C	-2.49972	-4.67513	-1.99804
C	-1.83554	-0.96248	-1.86043
Pd	-0.80131	-0.00242	-0.39713
P	0.08408	1.13310	1.41246
C	0.20307	0.31941	3.11133
C	-1.23942	0.05127	3.54784
C	0.92159	-1.01724	2.92700
C	0.95013	1.17976	4.12402
C	1.64583	2.02516	1.18870
C	2.86193	1.54995	0.70946
C	3.02625	0.10939	0.41800
C	3.86402	-0.67561	1.21523
O	4.48155	-0.07505	2.30120
C	5.87909	0.09996	2.20788
C	3.95639	-2.05254	0.99619
C	4.77326	-2.93276	1.89529
C	3.22845	-2.60838	-0.04521
C	2.40479	-1.85445	-0.86759
C	1.63223	-2.48805	-1.98442
C	2.29853	-0.48706	-0.61344
O	1.45617	0.30020	-1.39170
C	2.10737	0.90108	-2.49327
C	3.90375	2.45048	0.51761
C	3.74269	3.79179	0.82255
C	2.53992	4.27250	1.30842
C	1.48491	3.38622	1.47176
O	0.27678	3.79933	1.90617
C	-0.71060	2.78471	1.80471
C	-2.75555	0.46347	-0.64217
C	-3.88269	-0.12989	-0.10034
C	-3.86459	-1.45760	0.60282
C	-5.13066	0.51294	-0.20490
C	-5.26577	1.75051	-0.76210
C	-4.12179	2.45854	-1.17664
C	-2.85315	1.81978	-1.09858
C	-1.71420	2.60423	-1.42188
C	-1.82539	3.90585	-1.82697
C	-3.08708	4.50524	-1.94944
C	-4.20670	3.79231	-1.62861

H	-1.78874	0.61738	-4.84961
H	-2.80522	1.36963	-3.62090
H	-1.06605	1.19430	-3.34441
H	-3.06180	-1.28384	-5.00291
H	-3.44390	-3.60058	-4.27537
H	-0.71858	-2.14467	0.32144
H	-0.77374	-4.52397	0.88866
H	-1.94124	-6.14670	-0.56301
H	-2.97750	-5.37938	-2.66536
H	-1.79597	-0.41339	2.72966
H	-1.75426	0.96754	3.82957
H	-1.25750	-0.62100	4.40436
H	0.80802	-1.63556	3.81655
H	1.98261	-0.86365	2.75145
H	0.50678	-1.56304	2.07563
H	1.98908	1.30708	3.82244
H	0.93583	0.69540	5.09973
H	0.49751	2.16401	4.22939
H	6.39650	-0.82485	1.94147
H	6.13286	0.87017	1.47203
H	6.20543	0.42870	3.19333
H	4.30656	-3.91107	1.98345
H	4.85527	-2.49213	2.88640
H	5.77682	-3.07766	1.49271
H	3.29179	-3.67422	-0.21205
H	0.74528	-1.89761	-2.22056
H	2.24849	-2.55480	-2.88218
H	1.32125	-3.49431	-1.71347
H	2.87805	1.60300	-2.15327
H	1.34647	1.44117	-3.05563
H	2.56745	0.14533	-3.13930
H	4.84073	2.10012	0.10933
H	4.56500	4.47509	0.67061
H	2.40163	5.31519	1.54483
H	-1.40326	3.05319	0.99392
H	-1.28128	2.76613	2.73756
H	-4.04843	-2.27381	-0.09699
H	-4.64349	-1.48274	1.36325
H	-2.90222	-1.62788	1.08507
H	-6.00134	-0.00458	0.17436
H	-6.23606	2.21768	-0.84944
H	-0.71544	2.15952	-1.32928
H	-0.93875	4.48291	-2.05005
H	-3.16318	5.52971	-2.28397
H	-5.18543	4.24661	-1.69779

L22

C	-3.85318	-1.55469	0.78166
C	-4.08555	-0.16733	0.25428
C	-5.37105	0.09918	-0.26128
C	-5.69176	1.29282	-0.83569
C	-4.70320	2.28324	-0.98464
C	-3.40653	2.04247	-0.45016
C	-2.42021	3.03401	-0.68903
C	-2.69897	4.18167	-1.37749
C	-3.99137	4.42077	-1.86581
C	-4.96778	3.48687	-1.67050
C	-3.12268	0.82526	0.24683
Pd	-1.12887	0.74535	0.66189
P	-0.30649	-0.05835	-1.40717
C	0.48612	0.75187	-2.93032
C	1.14413	-0.27392	-3.84698
C	1.52950	1.75632	-2.43805
C	-0.61402	1.53061	-3.65940
C	0.52522	-1.66110	-1.17936
C	1.80774	-1.96256	-0.73310
C	2.80367	-0.90926	-0.45676
C	2.58786	0.02801	0.55989
O	1.42996	-0.10912	1.26690
C	0.96400	0.96885	2.00599
C	0.74091	0.79341	3.36890
C	0.26518	1.84926	4.12731
C	0.04090	3.08468	3.54369
C	0.29360	3.27285	2.19391
C	0.75555	2.22139	1.40842
C	3.55600	0.98257	0.85105
C	4.72988	1.01392	0.11978
C	4.95860	0.10282	-0.89377
C	4.00075	-0.86593	-1.17220
O	4.13393	-1.74211	-2.22037
C	5.30744	-2.42781	-2.38848
C	6.18873	-2.69821	-1.34809
C	7.32485	-3.44865	-1.59163
C	7.58719	-3.93703	-2.85956
C	6.69968	-3.67736	-3.89127
C	5.56360	-2.92615	-3.66217
C	2.16124	-3.29518	-0.54124
C	1.24357	-4.30851	-0.75193
C	-0.04220	-4.02181	-1.17503
C	-0.39301	-2.69851	-1.39315
O	-1.63682	-2.36277	-1.80138
C	-1.71037	-1.00970	-2.21140
C	-2.30068	0.78842	2.31821
C	-2.95884	1.80153	2.98552

C	-3.22498	3.15809	2.39559
C	-3.43884	1.58659	4.29428
C	-3.24028	0.41277	4.95655
C	-2.50541	-0.62113	4.34658
C	-2.03209	-0.43258	3.01828
C	-1.24543	-1.47819	2.46152
C	-0.96494	-2.61965	3.15892
C	-1.45735	-2.79851	4.45987
C	-2.21265	-1.81706	5.03486
H	-3.97281	-1.58637	1.86508
H	-4.57151	-2.24434	0.34175
H	-2.85188	-1.90292	0.53284
H	-6.11704	-0.68096	-0.19273
H	-6.68770	1.47937	-1.21121
H	-1.40190	2.86896	-0.31879
H	-1.92272	4.91410	-1.55210
H	-4.20203	5.33653	-2.39867
H	-5.96655	3.64826	-2.05196
H	0.44656	-1.05743	-4.13824
H	1.49523	0.21739	-4.75380
H	1.99715	-0.74161	-3.35953
H	1.04819	2.53786	-1.84966
H	2.02000	2.23193	-3.28654
H	2.29535	1.27283	-1.83470
H	-0.16570	2.23135	-4.36244
H	-1.21936	2.09728	-2.94958
H	-1.27540	0.87598	-4.22103
H	0.93310	-0.17235	3.81155
H	0.07700	1.70343	5.17963
H	-0.32330	3.90610	4.14095
H	0.15524	4.24868	1.74838
H	1.07986	2.39008	0.38282
H	3.39308	1.68662	1.65338
H	5.47604	1.76009	0.34530
H	5.86938	0.13353	-1.47215
H	5.98071	-2.32922	-0.35394
H	8.00542	-3.65597	-0.77911
H	8.47589	-4.52159	-3.04185
H	6.89412	-4.06046	-4.88182
H	4.85920	-2.71794	-4.45226
H	3.16000	-3.53278	-0.20618
H	1.53269	-5.33507	-0.58418
H	-0.77234	-4.79758	-1.34052
H	-2.68552	-0.61588	-1.90789
H	-1.64414	-0.98049	-3.30722
H	-3.33931	3.89476	3.18902
H	-4.14519	3.14650	1.80989
H	-2.41171	3.47242	1.74609
H	-3.97768	2.39094	4.77713
H	-3.61935	0.26946	5.95871
H	-0.83971	-1.37922	1.44808
H	-0.35778	-3.39154	2.70617
H	-1.23286	-3.70817	4.99833
H	-2.59497	-1.93503	6.03971

L23

C	-3.54601	-1.99204	0.94935
C	-3.77818	-0.64306	0.33738
C	-5.11337	-0.20219	0.30824
C	-5.47013	0.99846	-0.22887
C	-4.46179	1.90651	-0.60896
C	-3.10343	1.49534	-0.54455
C	-2.12503	2.50075	-0.70707
C	-2.45884	3.79429	-1.01319
C	-3.79750	4.15581	-1.19828
C	-4.77683	3.22755	-0.98361
C	-2.77088	0.12051	-0.24589
Pd	-0.72251	-0.05683	-0.14935
P	0.16419	1.04104	1.74344
C	-0.09677	-0.01632	3.28512
C	0.69457	0.42037	4.51332
C	0.31872	-1.42661	2.86733
C	-1.60039	0.02428	3.56463
C	1.94820	1.29322	1.78455
C	2.91163	0.51690	1.16050
C	2.45682	-0.46341	0.15943
C	2.82991	-1.81483	0.21634
O	3.54993	-2.15748	1.31616
C	4.01157	-3.48882	1.49974
C	5.27278	-3.74671	0.67531
C	4.28980	-3.63437	2.99266
C	2.44562	-2.67525	-0.80779
C	1.66718	-2.20788	-1.85313
C	1.24079	-0.89019	-1.90004
C	1.64042	-0.00774	-0.88978
O	1.33663	1.34355	-0.98140
C	2.00222	2.06152	-2.02443
C	1.42600	3.47166	-2.00770
C	3.51353	2.07694	-1.80409
C	4.24965	0.79818	1.41801
C	4.58588	1.85339	2.25073
C	3.62188	2.68437	2.80485
C	2.28783	2.40502	2.55418
O	1.26041	3.16217	3.01011

C	0.02901	2.78383	2.40768
C	-1.92342	-0.90814	-1.57842
C	-1.99348	-2.30762	-1.57710
C	-1.17801	-3.16459	-0.63865
C	-2.74558	-2.97683	-2.55761
C	-3.37678	-2.30764	-3.56798
C	-3.08687	-0.94253	-3.76912
C	-2.27661	-0.27079	-2.81712
C	-1.77936	1.00038	-3.15723
C	-2.16679	1.63655	-4.30607
C	-3.08054	1.02575	-5.17304
C	-3.50995	-0.24780	-4.91912
H	-4.21116	-2.12667	1.80155
H	-2.51869	-2.09615	1.29120
H	-3.76833	-2.77394	0.22237
H	-5.87114	-0.86659	0.70097
H	-6.50689	1.29036	-0.30842
H	-1.07494	2.25323	-0.53629
H	-1.68789	4.54683	-1.11001
H	-4.04909	5.16926	-1.47610
H	-5.81990	3.49920	-1.06843
H	0.45019	1.43807	4.80936
H	0.45702	-0.23836	5.34846
H	1.76662	0.36157	4.33077
H	0.02779	-2.15928	3.61931
H	1.39771	-1.48269	2.72376
H	-0.17290	-1.68331	1.91970
H	-1.89670	0.98898	3.97316
H	-1.87826	-0.74676	4.28183
H	-2.15941	-0.13847	2.63995
H	3.22144	-4.19728	1.21265
H	5.08727	-3.57463	-0.38245
H	5.60335	-4.77262	0.81193
H	6.06390	-3.07459	0.99927
H	5.03215	-2.90252	3.29979
H	4.65944	-4.63251	3.20827
H	3.37960	-3.46163	3.56091
H	2.74600	-3.71072	-0.80480
H	1.39327	-2.87931	-2.65334
H	0.68220	-0.52270	-2.75298
H	1.78504	1.58225	-2.99233
H	1.57959	3.91367	-1.02575
H	1.92091	4.08485	-2.75538
H	0.36052	3.44973	-2.22061
H	3.90917	1.06304	-1.79950
H	3.99529	2.63440	-2.60281
H	3.74829	2.54543	-0.85081
H	5.02086	0.20337	0.95358
H	5.62772	2.05888	2.44852
H	3.89422	3.53206	3.41311
H	-0.76870	2.89648	3.14514
H	-0.18142	3.46706	1.57104
H	-1.77856	-3.89790	-0.10277
H	-0.44637	-3.71699	-1.23077
H	-0.61911	-2.54059	0.07271
H	-2.83403	-4.05317	-2.48316
H	-4.02331	-2.82304	-4.26319
H	-1.02385	1.44457	-2.52064
H	-1.76303	2.60846	-4.55301
H	-3.41044	1.54898	-6.05919
H	-4.16153	-0.75701	-5.61603

L24

C	0.15507	2.85380	1.02728
C	-1.35637	2.82701	1.11884
C	-1.96510	3.96433	1.68129
C	-3.30192	4.00176	1.95002
C	-4.04904	2.80823	1.88164
C	-3.42371	1.62847	1.39455
C	-4.10561	0.41281	1.58862
C	-5.37932	0.37159	2.08725
C	-6.04336	1.55519	2.42734
C	-5.37552	2.74551	2.35073
C	-2.12218	1.70138	0.78976
Pd	-0.85731	0.26926	0.06159
P	0.52020	-1.45984	-0.55881
C	0.73053	-2.10043	1.21397
C	1.62414	-3.32774	1.35467
C	-0.66665	-2.37307	1.77920
C	1.32668	-0.90726	1.96567
C	2.17867	-1.74389	-1.22450
C	3.31724	-0.95088	-1.16116
C	3.26910	0.46541	-0.75523
C	2.49394	1.35852	-1.48738
C	2.57357	2.72111	-1.25666
C	3.41527	3.20416	-0.26931
C	4.15953	2.33006	0.50014
C	4.09011	0.96206	0.26551
O	4.85049	0.05507	0.95938
C	5.11486	0.24282	2.29148
C	6.28575	-0.32903	2.77963
C	6.58963	-0.23824	4.12409
C	5.73361	0.41998	4.99173
C	4.56327	0.97702	4.50671

C	4.24607	0.89062	3.16336
C	4.52604	-1.48588	-1.60193
C	4.58402	-2.76569	-2.12352
C	3.44127	-3.53763	-2.24997
C	2.23700	-3.01953	-1.80056
O	1.08951	-3.72309	-1.88826
C	-0.05340	-2.95768	-1.52291
C	-0.87518	-2.54396	-2.76014
C	-1.26891	-3.77933	-3.56501
C	-0.11912	-1.53907	-3.62366
C	-2.39489	1.11758	-1.01088
C	-2.27764	2.20898	-1.86163
C	-1.07183	3.09802	-1.85420
C	-3.32846	2.53745	-2.73577
C	-4.45403	1.77278	-2.82985
C	-4.51023	0.53658	-2.15600
C	-3.44506	0.17657	-1.28720
C	-3.43620	-1.14797	-0.79433
C	-4.45146	-2.02721	-1.06035
C	-5.55098	-1.62278	-1.82685
C	-5.56788	-0.36977	-2.37136
H	0.52161	3.80463	0.64219
H	0.55728	2.73946	2.03887
H	0.55305	2.03121	0.40735
H	-1.34756	4.83318	1.86781
H	-3.78030	4.90891	2.28926
H	-3.58556	-0.51397	1.39878
H	-5.87116	-0.57946	2.23165
H	-7.06084	1.51715	2.78840
H	-5.84525	3.66223	2.67955
H	2.64012	-3.10965	1.02924
H	1.24623	-4.16376	0.76872
H	1.66310	-3.63616	2.39919
H	-1.17175	-3.17535	1.24060
H	-1.27834	-1.45401	1.72569
H	-0.59481	-2.66525	2.82700
H	2.30729	-0.65699	1.56530
H	0.66078	-0.03136	1.86224
H	1.43590	-1.12891	3.02746
H	1.86061	0.97140	-2.27532
H	1.99609	3.40699	-1.85704
H	3.49858	4.26653	-0.09709
H	4.81932	2.70333	1.26976
H	6.93911	-0.84116	2.09056
H	7.50171	-0.68112	4.49518
H	5.97503	0.49435	6.04101
H	3.88413	1.48171	5.17787
H	3.32505	1.31825	2.79547
H	5.41795	-0.88152	-1.54471
H	5.53056	-3.16308	-2.45781
H	3.46999	-4.52532	-2.68152
H	-0.67988	-3.61098	-0.90013
H	-1.78368	-2.05322	-2.39042
H	-1.85260	-4.46460	-2.95436
H	-0.37994	-4.29994	-3.91338
H	-1.86667	-3.49150	-4.42614
H	0.05317	-0.62109	-3.05528
H	-0.70871	-1.28347	-4.50091
H	0.83655	-1.94353	-3.95315
H	-0.18200	2.50739	-1.62844
H	-0.94781	3.56511	-2.82962
H	-1.18210	3.88693	-1.10839
H	-3.23420	3.43928	-3.32492
H	-5.27835	2.06968	-3.46130
H	-2.56824	-1.48755	-0.22307
H	-4.40765	-3.03925	-0.68288
H	-6.36319	-2.31213	-2.00611
H	-6.38681	-0.05752	-3.00418

L25

C	-3.79892	0.98353	1.75021
C	-3.56766	0.39686	0.38554
C	-4.69295	-0.22219	-0.19876
C	-4.65953	-0.76950	-1.44563
C	-3.46188	-0.75157	-2.18465
C	-2.31385	-0.13539	-1.61192
C	-1.11334	-0.17663	-2.36885
C	-1.05964	-0.76040	-3.60445
C	-2.20409	-1.34622	-4.16344
C	-3.37633	-1.34072	-3.46300
C	-2.38137	0.47473	-0.31770
Pd	-0.45330	0.91524	0.21162
P	0.30779	-1.36453	0.97403
C	0.11047	-2.98848	0.00299
C	0.68166	-4.20295	0.73413
C	-1.38281	-3.18820	-0.25566
C	0.84992	-2.83099	-1.32587
C	2.00746	-1.54753	1.59470
C	3.20734	-1.14845	1.01425
C	3.21834	-0.60422	-0.35794
C	2.50019	0.55922	-0.65489
C	1.85912	1.33747	0.36729
C	1.22023	2.52717	0.05121
C	1.17079	2.97153	-1.30372

C	1.75858	2.24731	-2.28994
C	2.45488	1.03604	-2.00314
C	3.12828	0.34040	-2.99627
C	3.86847	-0.80575	-2.71065
C	3.91453	-1.29570	-1.37018
C	4.63424	-2.49331	-1.12638
C	5.30161	-3.13315	-2.12782
C	5.28357	-2.62613	-3.44105
C	4.57582	-1.49796	-3.72394
C	4.38677	-1.32554	1.73553
C	4.36946	-1.92256	2.98421
C	3.18331	-2.35965	3.54955
C	2.00060	-2.15518	2.85556
O	0.80672	-2.54457	3.35365
C	-0.28108	-1.93390	2.67389
C	-0.85817	-0.78874	3.51366
C	0.22783	0.09368	4.11597
C	-1.72547	2.56792	-0.07542
C	-2.21146	3.19826	-1.20827
C	-2.05272	2.69355	-2.61513
C	-2.92402	4.41125	-1.09267
C	-3.11445	5.03976	0.09946
C	-2.52775	4.50687	1.26093
C	-1.82679	3.27238	1.16960
C	-1.18261	2.82005	2.34954
C	-1.23679	3.52069	3.52219
C	-1.96234	4.71688	3.60331
C	-2.59528	5.19219	2.49141
H	-4.42342	0.31570	2.34232
H	-4.31900	1.93870	1.65791
H	-2.87219	1.16057	2.28548
H	-5.60933	-0.24676	0.37510
H	-5.53841	-1.22768	-1.87573
H	-0.20475	0.27957	-1.95475
H	-0.13010	-0.77560	-4.15665
H	-2.14969	-1.80039	-5.14196
H	-4.26642	-1.79477	-3.87586
H	1.74628	-4.08313	0.92809
H	0.17801	-4.39575	1.67789
H	0.55457	-5.08198	0.10319
H	-1.93613	-3.32795	0.67175
H	-1.80214	-2.32967	-0.77448
H	-1.53723	-4.07027	-0.87537
H	0.57601	-3.63945	-2.00235
H	0.59504	-1.88970	-1.80693
H	1.92615	-2.86630	-1.17560
H	2.11339	1.11995	1.40305
H	0.96403	3.23739	0.83432
H	0.68711	3.91399	-1.52080
H	1.74179	2.59152	-3.31452
H	3.08894	0.70252	-4.01437
H	4.64500	-2.90431	-0.12773
H	5.84767	-4.04171	-1.91973
H	5.82517	-3.14611	-4.21738
H	4.53931	-1.10634	-4.73059
H	5.31949	-0.98704	1.30894
H	5.29433	-2.05354	3.52575
H	3.15999	-2.83977	4.51470
H	-1.06220	-2.69403	2.55063
H	-1.46895	-1.20333	4.31909
H	-1.50899	-0.20714	2.85283
H	0.85493	-0.48148	4.79393
H	-0.22259	0.90810	4.67697
H	0.86481	0.51114	3.33452
H	-1.09391	2.20330	-2.75750
H	-2.84428	1.98694	-2.86603
H	-2.11660	3.52726	-3.31282
H	-3.33085	4.84476	-1.99629
H	-3.67689	5.96018	0.16249
H	-0.60391	1.89129	2.30778
H	-0.71354	3.16163	4.39738
H	-2.00616	5.25592	4.53841
H	-3.14772	6.12087	2.52785

L26

C	0.04147	0.09932	2.61002
C	-1.24240	-0.61172	2.26118
C	-2.25038	-0.63001	3.23859
C	-3.51396	-1.08256	2.97544
C	-3.88716	-1.32213	1.63743
C	-2.88801	-1.25176	0.63247
C	-3.30396	-1.22589	-0.71151
C	-4.61892	-1.39215	-1.05584
C	-5.58216	-1.60642	-0.06317
C	-5.22433	-1.54686	1.25558
C	-1.50147	-1.15310	0.99532
Pd	-0.08167	-0.96638	-0.43959
P	0.09256	1.23946	-1.24173
C	-1.06295	2.68407	-0.84606
C	-0.73874	3.93999	-1.65067
C	-2.49769	2.22629	-1.10900
C	-0.91147	2.95638	0.64960
C	1.66303	2.09243	-1.58066
C	2.68351	2.46886	-0.71344

C	2.75347	2.05126	0.70221
C	2.68233	3.00400	1.72814
C	2.35696	4.36241	1.47402
C	2.26125	5.26386	2.48943
C	2.48733	4.86678	3.82381
C	2.81256	3.57680	4.10845
C	2.92599	2.60695	3.07966
C	3.30616	1.29604	3.34811
C	3.39818	0.34571	2.33626
C	3.06496	0.71255	0.99414
C	3.11526	-0.29456	-0.00887
C	3.52202	-1.56395	0.28415
C	3.91855	-1.90044	1.59639
C	3.84634	-0.97585	2.59230
C	3.76237	3.19017	-1.22422
C	3.84144	3.49841	-2.57026
C	2.86940	3.06109	-3.45242
C	1.79096	2.34809	-2.95208
O	0.83410	1.87196	-3.77186
C	-0.12158	1.05963	-3.10165
C	-0.02748	-0.41730	-3.54643
C	0.15828	-0.49841	-5.06140
C	1.07769	-1.20533	-2.83242
C	-0.71508	-2.74173	0.42737
C	-0.17484	-3.40413	1.53122
C	0.93489	-2.82142	2.34811
C	-0.68209	-4.63956	1.96571
C	-1.69031	-5.27846	1.30664
C	-2.08290	-4.79563	0.04238
C	-1.52296	-3.58008	-0.43461
C	-1.72565	-3.28494	-1.79423
C	-2.52098	-4.05347	-2.60346
C	-3.18452	-5.16654	-2.07965
C	-2.94960	-5.53609	-0.78439
H	0.56671	-0.35346	3.44753
H	-0.19429	1.12323	2.90698
H	0.70177	0.14338	1.73575
H	-1.99602	-0.28356	4.23137
H	-4.25177	-1.17074	3.75889
H	-2.56639	-0.98673	-1.47546
H	-4.91929	-1.35311	-2.09299
H	-6.61112	-1.77790	-0.34448
H	-5.97048	-1.64259	2.03205
H	-1.47279	4.71265	-1.42573
H	-0.76725	3.75318	-2.72257
H	0.24676	4.32502	-1.39501
H	-2.69422	1.28564	-0.59340
H	-3.19634	2.97033	-0.72998
H	-2.69746	2.09318	-2.17061
H	-1.24923	2.08744	1.21111
H	0.12378	3.16677	0.91133
H	-1.51881	3.81192	0.94153
H	2.18236	4.67087	0.45299
H	2.00866	6.29324	2.28157
H	2.40189	5.59900	4.61311
H	2.99169	3.26356	5.12709
H	3.54630	1.01254	4.36332
H	2.91506	-0.01021	-1.03203
H	3.57358	-2.31513	-0.49174
H	4.27368	-2.89995	1.79842
H	4.13289	-1.22930	3.60282
H	4.55090	3.49273	-0.55119
H	4.68336	4.06254	-2.94197
H	2.93277	3.25653	-4.51074
H	-1.10777	1.43027	-3.41356
H	-1.00678	-0.85504	-3.30603
H	0.11539	-1.53381	-5.39176
H	-0.62249	0.06157	-5.57110
H	1.11976	-0.08005	-5.34886
H	0.89259	-1.33443	-1.72940
H	1.15136	-2.20511	-3.26575
H	2.03939	-0.70929	-2.97907
H	0.58830	-2.56660	3.34865
H	1.34486	-1.94354	1.85385
H	1.72829	-3.56196	2.45213
H	-0.27405	-5.05807	2.87584
H	-2.13717	-6.18047	1.69775
H	-1.17794	-2.45646	-2.22890
H	-2.63470	-3.80431	-3.64933
H	-3.84379	-5.74774	-2.70803
H	-3.39975	-6.43167	-0.37889

L27

C	-3.29905	-0.24382	-3.01597
C	-4.00259	-0.51461	-1.70325
C	-5.40498	-0.61144	-1.72312
C	-6.11159	-1.01229	-0.62430
C	-5.42241	-1.55669	0.47916
C	-4.00291	-1.53496	0.47753
C	-3.33032	-2.31670	1.43459
C	-4.00953	-2.96134	2.43273
C	-5.40300	-2.85485	2.51802
C	-6.09425	-2.18970	1.54326
C	-3.29924	-0.78634	-0.52260

Pd	-1.29169	-0.59927	-0.77530
P	0.94011	-0.52767	-1.42463
C	1.81993	0.13580	-2.96229
C	2.60881	1.37605	-2.54298
C	2.75357	-0.89229	-3.58967
C	0.70732	0.55501	-3.92552
C	2.16794	-1.56359	-0.58276
C	3.22430	-1.20650	0.26061
C	3.54555	0.19121	0.56849
C	2.57781	1.17474	0.78320
O	1.24591	0.94008	0.74798
C	0.55398	2.16188	1.02594
C	1.60486	3.27745	1.16078
C	2.88845	2.50945	1.03402
C	4.19889	2.93730	1.10024
C	5.18210	1.98526	0.90975
C	4.86372	0.65356	0.65543
O	5.96238	-0.12271	0.46177
C	7.12720	0.69823	0.54878
C	6.67744	2.12572	0.91161
C	4.01602	-2.22079	0.79952
C	3.77826	-3.55014	0.50626
C	2.74575	-3.91409	-0.33901
C	1.94621	-2.91731	-0.87559
O	0.93248	-3.23950	-1.71370
C	0.15115	-2.11719	-2.06432
C	-2.39202	0.59662	0.42454
C	-3.01064	1.81497	0.16414
C	-3.17398	2.34389	-1.22889
C	-3.55176	2.56910	1.21914
C	-3.43278	2.17962	2.52193
C	-2.61089	1.08051	2.84654
C	-2.03325	0.32347	1.79380
C	-1.04414	-0.62809	2.13967
C	-0.71443	-0.87530	3.44564
C	-1.37433	-0.20143	4.48115
C	-2.29453	0.76421	4.18313
H	-2.24722	0.05600	-2.86008
H	-3.30297	-1.16942	-3.60040
H	-3.81047	0.51650	-3.60499
H	-5.92508	-0.33450	-2.63074
H	-7.19165	-0.99674	-0.61951
H	-2.26125	-2.46394	1.32893
H	-3.47366	-3.56471	3.15113
H	-5.92787	-3.33712	3.33014
H	-7.17518	-2.16390	1.55612
H	3.42298	1.11109	-1.87186
H	1.95958	2.08696	-2.03265
H	3.03230	1.86690	-3.41884
H	3.20498	-0.47903	-4.49116
H	3.55249	-1.15564	-2.89775
H	2.22505	-1.80335	-3.86478
H	1.11548	1.14045	-4.74787
H	0.19564	-0.30757	-4.35038
H	-0.02728	1.17169	-3.39855
H	-0.01540	2.01940	1.94842
H	-0.15483	2.32715	0.20684
H	1.51253	4.02626	0.37124
H	1.52739	3.78945	2.12035
H	4.44641	3.96989	1.28853
H	7.63630	0.65992	-0.41996
H	7.78628	0.26123	1.30442
H	7.04725	2.43073	1.89246
H	7.01837	2.85826	0.17881
H	4.82546	-1.95541	1.45922
H	4.40472	-4.31491	0.94008
H	2.54717	-4.94390	-0.58895
H	-0.85970	-2.21712	-1.55602
H	-0.02609	-2.13522	-3.14612
H	-2.32185	2.04995	-1.84677
H	-4.08950	1.95582	-1.67676
H	-3.23670	3.43052	-1.21232
H	-4.09575	3.47051	0.97192
H	-3.90750	2.73813	3.31510
H	-0.47847	-1.13643	1.34402
H	0.06063	-1.59003	3.68340
H	-1.13089	-0.42902	5.50901
H	-2.77751	1.32561	4.97087

L28

Pd	0.17739	-0.57566	0.09088
C	2.73459	-1.09623	0.25685
C	3.39410	-1.38952	1.43551
C	1.81188	-2.04341	-0.30731
C	1.63780	-3.30821	0.33571
C	3.03004	0.16104	-0.48566
C	2.58585	1.40817	0.04586
C	3.76796	0.12758	-1.65158
C	2.88550	2.60730	-0.65200
C	4.01789	1.32650	-2.34839
C	1.60757	2.72174	1.82704
C	2.50885	3.84489	-0.09078
C	1.89925	3.90415	1.13025
H	1.14861	2.78284	2.80410

H	2.73606	4.74935	-0.63775
H	1.64042	4.85748	1.56774
C	1.91009	1.50058	1.28757
H	1.70096	0.59767	1.85747
C	3.58394	2.52939	-1.87476
H	3.79062	3.44136	-2.41630
C	4.33960	-1.14673	-2.20217
H	5.37094	-0.98516	-2.51231
H	3.77786	-1.47486	-3.07719
H	4.31573	-1.94447	-1.46396
H	4.57715	1.27534	-3.27200
C	0.88310	-4.30952	-0.30200
C	0.29560	-4.08328	-1.51949
C	0.43637	-2.84449	-2.15073
C	1.18270	-1.83606	-1.57464
C	2.28896	-3.53947	1.56762
H	2.12690	-4.48304	2.07004
C	3.13412	-2.60809	2.09550
H	3.64548	-2.80568	3.02717
H	0.78732	-5.26946	0.18685
H	-0.26564	-4.86515	-2.00989
H	-0.00860	-2.69346	-3.12516
H	1.43365	-0.95606	-2.16467
C	4.41673	-0.46403	2.03025
H	3.99693	0.08657	2.87270
H	5.26482	-1.03877	2.39893
H	4.77165	0.25663	1.29774
P	-1.39954	1.19900	-0.02614
C	-3.12967	1.21300	0.51382
C	-1.59140	1.37888	-1.89673
C	-1.33707	2.97344	0.54846
C	-3.99411	0.13860	0.67550
C	-3.57031	2.51972	0.72641
C	-2.61091	2.41786	-2.35102
C	-0.19624	1.73193	-2.41474
C	-1.99434	-0.01024	-2.39567
O	-2.64889	3.50640	0.61219
C	-5.32587	0.39471	0.98305
C	-3.45502	-1.22076	0.51913
C	-4.89903	2.77122	1.03335
H	-2.35286	3.41459	-2.00016
H	-2.64217	2.44128	-3.44025
H	-3.60850	2.17308	-1.98964
H	0.07820	2.75144	-2.14685
H	0.54206	1.05649	-1.96899
H	-0.14940	1.63828	-3.49902
H	-1.37302	-0.77685	-1.91109
H	-3.03438	-0.21838	-2.15063
H	-1.87024	-0.08668	-3.47535
C	-5.76779	1.69773	1.14504
H	-6.00974	-0.43137	1.10911
C	-4.02807	-2.13001	-0.35304
C	-2.30745	-1.58356	1.25506
H	-5.23330	3.78534	1.18367
H	-6.80549	1.88272	1.38061
C	-3.47472	-3.38722	-0.53228
C	-1.73600	-2.83667	1.04338
C	-2.33008	-3.72870	0.15932
C	-0.68375	-0.96823	2.87423
H	-1.89759	-4.70925	0.03567
H	-0.72700	3.61141	-0.09406
H	-0.90414	2.99133	1.55849
O	-1.90526	-0.70201	2.21712
H	-4.91155	-1.83900	-0.90332
H	-3.93503	-4.09107	-1.20888
H	-0.87649	-3.16413	1.61293
H	-0.75243	-1.85928	3.50856
H	0.13413	-1.09894	2.12554
H	-0.48616	-0.09595	3.49792

L29

Pd	-0.56351	0.02116	0.67156
C	-3.06327	1.86969	0.12215
C	-4.10094	2.44599	0.82764
C	-1.84239	2.56923	-0.07367
C	-1.71532	3.88866	0.43942
C	-3.23692	0.50092	-0.43520
C	-3.07383	-0.61703	0.43364
C	-3.60957	0.31472	-1.75095
C	-3.35315	-1.92462	-0.05375
C	-3.83308	-0.99137	-2.22646
C	-2.63080	-1.55476	2.62966
C	-3.28901	-3.01694	0.83650
C	-2.92642	-2.83723	2.14424
H	-2.38317	-1.43372	3.67626
H	-3.53385	-4.00199	0.46389
H	-2.87692	-3.68155	2.81721
C	-2.71190	-0.45521	1.80432
H	-2.63960	0.55192	2.21649
C	-3.71926	-2.07761	-1.40681
H	-3.91903	-3.07156	-1.78188
C	-3.77526	1.47991	-2.68221
H	-4.46457	1.23190	-3.48627
H	-2.81697	1.74884	-3.12894

H	-4.14686	2.35372	-2.15086
H	-4.11400	-1.12199	-3.26219
C	-0.54472	4.62459	0.16610
C	0.46695	4.07873	-0.57179
C	0.35925	2.77064	-1.06559
C	-0.76772	2.01962	-0.82812
C	-2.78166	4.43808	1.17874
H	-2.67280	5.43293	1.58692
C	-3.93715	3.73474	1.36538
H	-4.75145	4.16968	1.92788
H	-0.47064	5.63482	0.54404
H	1.35473	4.65265	-0.79354
H	1.16320	2.37932	-1.67436
H	-0.91150	1.06683	-1.35352
C	-5.39520	1.71277	1.02557
H	-5.26729	0.89230	1.73226
H	-6.16033	2.38228	1.41118
H	-5.74227	1.28318	0.08705
P	0.40061	-1.85901	-0.54214
C	1.92525	-2.44558	0.26076
C	0.89871	-1.83587	-2.36630
C	-0.25356	-3.59127	-0.31377
C	3.00135	-1.71897	0.76206
C	1.90948	-3.84194	0.37244
C	1.63944	-3.08725	-2.82510
C	-0.39833	-1.66369	-3.15774
C	1.78746	-0.60524	-2.54598
O	0.81718	-4.49099	-0.09529
C	4.05496	-2.41723	1.35340
C	3.07327	-0.24359	0.68562
C	2.97372	-4.52715	0.93607
H	1.02653	-3.97980	-2.72291
H	1.91015	-2.98216	-3.87544
H	2.55498	-3.23109	-2.25380
H	-1.04073	-2.53681	-3.05389
H	-0.95323	-0.79703	-2.79732
H	-0.18375	-1.52059	-4.21590
H	1.36890	0.24136	-1.99971
H	2.78727	-0.79172	-2.16078
H	1.87003	-0.33651	-3.59831
C	4.04384	-3.79830	1.42740
H	4.88661	-1.86006	1.75919
C	4.11044	0.34867	-0.06047
C	2.15019	0.55312	1.37836
H	2.94530	-5.60328	0.99536
H	4.87503	-4.31617	1.88230
C	4.23951	1.77090	-0.07966
C	2.28979	1.97854	1.35336
C	3.33636	2.54843	0.63995
C	0.18682	0.81861	2.82268
H	3.44642	3.62409	0.63593
H	-0.84277	-3.94243	-1.16342
C	5.28728	2.35820	-0.83196
C	5.03031	-0.41213	-0.82739
C	1.09397	-0.00965	2.17406
C	1.36599	2.78386	2.07433
C	6.02199	0.18892	-1.54314
H	1.50785	3.85434	2.06486
C	0.32470	2.22799	2.74918
H	5.37433	3.43576	-0.83045
C	6.15837	1.59086	-1.54251
H	1.13301	-1.07137	2.42325
H	-0.50781	0.39530	3.54394
H	-0.38190	2.85283	3.27962
H	4.93391	-1.48832	-0.84201
H	6.71194	-0.40817	-2.12175
H	6.95402	2.04589	-2.11424
H	-0.89923	-3.58979	0.57595

L30

Pd	-0.36318	-0.24165	0.60056
C	-3.53183	1.67566	0.50340
C	-4.37881	2.31854	1.38263
C	-2.26070	2.22039	0.19406
C	-1.89279	3.47822	0.74271
C	-3.94525	0.38362	-0.09989
C	-3.53640	-0.82053	0.53418
C	-4.74248	0.34421	-1.22166
C	-4.05540	-2.05932	0.07270
C	-5.18291	-0.90051	-1.71375
C	-2.41231	-1.98550	2.33651
C	-3.76771	-3.23603	0.79426
C	-2.97111	-3.19791	1.90546
H	-1.81794	-1.98267	3.24139
H	-4.19755	-4.16806	0.45556
H	-2.76950	-4.10114	2.46285
C	-2.67585	-0.80997	1.66545
H	-2.40044	0.15150	2.09850
C	-4.87281	-2.06609	-1.07693
H	-5.24861	-3.01118	-1.44234
C	-5.16130	1.58919	-1.94571
H	-6.24825	1.65405	-1.99095
H	-4.78944	1.56685	-2.97002
H	-4.77522	2.47995	-1.45709

H	-5.79921	-0.91316	-2.60179
C	-0.66231	4.05735	0.37326
C	0.18965	3.40584	-0.47519
C	-0.15676	2.15353	-1.00229
C	-1.36375	1.56111	-0.68950
C	-2.78328	4.11590	1.62955
H	-2.49776	5.06567	2.05929
C	-3.98286	3.54462	1.94594
H	-4.65240	4.03877	2.63604
H	-0.40933	5.03015	0.77121
H	1.13205	3.85249	-0.75724
H	0.50397	1.68727	-1.72212
H	-1.71135	0.68203	-1.24283
C	-5.71206	1.72743	1.73150
H	-5.61039	0.67536	1.99474
H	-6.16252	2.25841	2.56660
H	-6.38892	1.78863	0.87950
P	1.02068	-1.42177	-1.11516
C	2.45225	-2.13593	-0.24991
C	1.75424	-1.08643	-2.83541
C	0.38590	-3.17775	-1.29022
C	3.42434	-1.49710	0.51338
C	2.46310	-3.52946	-0.38547
C	2.48392	-2.28813	-3.43072
C	0.57380	-0.69907	-3.72811
C	2.73999	0.07633	-2.71316
O	1.45858	-4.09130	-1.09510
C	4.42630	-2.26966	1.09696
C	3.37561	-0.03340	0.71335
C	3.47474	-4.28780	0.18381
H	1.81578	-3.12825	-3.60247
H	2.91811	-2.00320	-4.38887
H	3.29261	-2.61773	-2.78103
H	-0.14542	-1.51512	-3.79394
H	0.05727	0.17356	-3.33168
H	0.91843	-0.46626	-4.73483
H	2.34704	0.87156	-2.08152
H	3.67416	-0.26218	-2.27185
H	2.95902	0.49297	-3.69567
C	4.45449	-3.64247	0.91986
H	5.17909	-1.78446	1.70090
C	4.39569	0.78273	0.19285
C	2.30417	0.52536	1.42383
H	3.47671	-5.35846	0.05598
H	5.24349	-4.22289	1.37476
C	4.31804	2.19808	0.36809
C	2.23516	1.94492	1.59298
C	3.23848	2.74323	1.05874
C	0.26931	0.30708	2.77278
H	3.18237	3.81572	1.18754
H	-0.04298	-3.38567	-2.27617
C	5.34488	3.01448	-0.16814
C	5.50044	0.25872	-0.52606
C	1.30754	-0.28729	2.06502
C	1.15423	2.50760	2.32625
C	6.46993	1.07611	-1.02467
H	1.12816	3.57968	2.45346
C	0.18680	1.72129	2.86880
H	5.27377	4.08384	-0.02586
C	6.39427	2.47105	-0.84361
H	1.50420	-1.35356	2.18333
H	-0.35008	-0.29620	3.43077
H	-0.62753	2.16271	3.42967
H	5.56748	-0.80991	-0.67249
H	7.30622	0.65922	-1.56709
H	7.17398	3.10018	-1.24771
C	-0.67577	-3.35701	-0.21298
H	-0.24523	-3.12627	0.76764
H	-1.49007	-2.65775	-0.41593
H	-1.06337	-4.37356	-0.19689

4.3 Ligand-Only Coordinates

L1

C	-2.48996	2.77195	-0.70889
C	-2.51412	1.37723	-0.66729
C	-1.32303	0.63455	-0.64028
C	-0.10050	1.32749	-0.63609
C	-0.10168	2.73025	-0.72189
C	-1.28256	3.46946	-0.74574
P	1.62207	0.67761	-0.57429
C	2.16699	2.42064	-1.05138
O	1.10872	3.36008	-0.77359
C	-1.39046	-0.85915	-0.65350
C	-1.90828	-1.57692	0.44172
C	-2.00407	-2.97458	0.41643
C	-1.58328	-3.65994	-0.72050
C	-1.07560	-2.98449	-1.82694
C	-0.98892	-1.58575	-1.79323
C	2.04319	0.63567	1.30238
C	3.57080	0.44685	1.38583
C	1.34718	-0.60176	1.89910
C	1.62197	1.88670	2.08912
O	-2.27801	-0.81720	1.51861

C	-2.82901	-1.46918	2.65146
O	-0.53824	-0.83580	-2.83924
C	-0.05633	-1.50159	-3.99467
H	-3.42590	3.32413	-0.73394
H	-3.46344	0.85096	-0.66594
H	-1.24543	4.55248	-0.80477
H	2.37474	2.44967	-2.12903
H	3.05928	2.75697	-0.51764
H	-2.39345	-3.52367	1.26483
H	-1.65356	-4.74419	-0.74478
H	-0.76264	-3.54099	-2.70167
H	4.11807	1.30528	0.97740
H	3.87685	0.33680	2.43489
H	3.89819	-0.44936	0.84582
H	0.25904	-0.48648	1.90017
H	1.66973	-0.74154	2.94042
H	1.59519	-1.51606	1.34739
H	1.89800	1.77029	3.14687
H	2.10800	2.79546	1.71952
H	0.53959	2.04236	2.04304
H	-3.06471	-0.67632	3.36374
H	-3.74772	-2.01361	2.39688
H	-2.11211	-2.16439	3.10755
H	0.26908	-0.71354	-4.67613
H	0.79548	-2.15220	-3.75899
H	-0.84396	-2.09470	-4.47817

L2

C	-2.42433	2.62726	-0.60616
C	-2.45858	1.23243	-0.56918
C	-1.27388	0.48291	-0.49721
C	-0.04824	1.16848	-0.43749
C	-0.03705	2.57100	-0.52350
C	-1.21223	3.31707	-0.59486
P	1.66501	0.51104	-0.31721
C	2.26119	2.25659	-0.76857
O	1.17735	3.19291	-0.53059
C	-1.34919	-1.01028	-0.52231
C	-1.91429	-1.73206	0.54657
C	-2.01654	-3.12901	0.50837
C	-1.55427	-3.80966	-0.61517
C	-0.99912	-3.13008	-1.69598
C	-0.90652	-1.73210	-1.64974
C	2.01365	0.45425	1.57402
C	3.53485	0.24969	1.71760
C	1.28326	-0.78063	2.13425
C	1.57198	1.70280	2.35375
O	-2.32304	-0.97710	1.61264
C	-2.91933	-1.63358	2.71961
O	-0.41043	-0.97846	-2.67270
C	0.11703	-1.64101	-3.80987
C	2.69275	2.36541	-2.22953
H	-3.35525	3.18524	-0.66628
H	-3.41035	0.71191	-0.60621
H	-1.16627	4.39989	-0.65205
H	3.07937	2.56833	-0.11263
H	-2.44264	-3.68123	1.33686
H	-1.62937	-4.89332	-0.64932
H	-0.65460	-3.68289	-2.56113
H	4.10695	1.10484	1.33735
H	3.79707	0.12957	2.77740
H	3.87452	-0.64651	1.18520
H	0.19704	-0.65499	2.09388
H	1.56388	-0.93037	3.18631
H	1.54390	-1.69344	1.58599
H	2.08115	2.61027	2.01341
H	0.49390	1.86921	2.26448
H	1.80301	1.57429	3.42086
H	-3.17821	-0.84412	3.42760
H	-3.83024	-2.17209	2.42712
H	-2.22336	-2.33508	3.19796
H	0.47570	-0.85150	-4.47274
H	0.95450	-2.29746	-3.54132
H	-0.65223	-2.22721	-4.33001
H	2.94117	3.40406	-2.47462
H	3.57225	1.73934	-2.41552
H	1.89101	2.03540	-2.89932

L3

C	0.26464	0.26647	3.45197
C	-0.81564	0.41281	2.58055
C	-0.63946	0.32341	1.19073
C	0.64775	0.05741	0.69282
C	1.72675	-0.03953	1.58730
C	1.55434	0.04906	2.96744
P	1.22312	-0.14039	-1.04250
C	2.99311	0.01728	-0.37285
O	2.96954	-0.23366	1.05754
C	-1.81025	0.54891	0.28827
C	-2.88759	-0.35686	0.24765
C	-4.00088	-0.12791	-0.57181
C	-4.03862	1.02382	-1.35371
C	-2.99751	1.94784	-1.33252
C	-1.89096	1.71187	-0.50491

C	1.09309	-2.03309	-1.36527
C	1.88210	-2.29643	-2.66311
C	-0.39437	-2.35224	-1.60547
C	1.62684	-2.92294	-0.23165
O	-2.75027	-1.46712	1.03637
C	-3.80507	-2.41512	1.06225
O	-0.84117	2.57520	-0.39097
C	-0.81954	3.73006	-1.21328
C	5.01311	1.56330	-0.08131
C	3.59423	1.40226	-0.63734
H	0.10345	0.34417	4.52414
H	-1.80783	0.60905	2.97459
H	2.40991	-0.03897	3.62941
H	3.64176	-0.75382	-0.80191
H	-4.82280	-0.83223	-0.60775
H	-4.89878	1.20545	-1.99266
H	-3.05551	2.83961	-1.94423
H	2.95298	-2.08883	-2.54797
H	1.78554	-3.35193	-2.95118
H	1.50819	-1.68717	-3.49437
H	-0.98348	-2.22186	-0.69270
H	-0.50098	-3.39818	-1.92592
H	-0.82752	-1.71776	-2.38750
H	2.69130	-2.75763	-0.03628
H	1.08324	-2.74921	0.70230
H	1.50016	-3.98141	-0.50032
H	-3.48858	-3.19102	1.76178
H	-4.74237	-1.96679	1.41686
H	-3.97097	-2.86408	0.07423
H	0.11216	4.24605	-0.97445
H	-0.82530	3.46624	-2.27851
H	-1.66614	4.39581	-0.99885
H	5.39965	2.56937	-0.27949
H	5.02879	1.39788	0.99999
H	5.70231	0.84379	-0.54118
H	3.59134	1.56750	-1.72276
H	2.93137	2.16410	-0.20585

L4

C	0.09330	0.18840	3.46673
C	-0.96075	0.39812	2.57655
C	-0.76757	0.29015	1.19022
C	0.50821	-0.05929	0.71428
C	1.56419	-0.21668	1.62756
C	1.37420	-0.11244	3.00453
P	1.10189	-0.29228	-1.00950
C	2.87269	-0.29935	-0.31493
O	2.80489	-0.47855	1.12464
C	-1.91169	0.58144	0.27179
C	-3.03472	-0.26662	0.21676
C	-4.12763	0.02356	-0.61045
C	-4.09928	1.18015	-1.38536
C	-3.01208	2.04882	-1.34957
C	-1.92553	1.75126	-0.51512
C	0.83144	-2.16840	-1.34890
C	1.63683	-2.49199	-2.62281
C	-0.66807	-2.36635	-1.63935
C	1.25234	-3.10115	-0.20238
O	-2.96251	-1.38590	1.00136
C	-4.06694	-2.27588	1.01668
O	-0.83423	2.56102	-0.38950
C	-0.77613	3.74563	-1.16753
C	3.70203	0.95628	-0.65328
C	5.15737	0.78490	-0.18843
C	3.09747	2.25341	-0.09600
H	-0.08043	0.28063	4.53578
H	-1.94525	0.65779	2.95276
H	2.21167	-0.25115	3.68080
H	3.40782	-1.17413	-0.69966
H	-4.98443	-0.63719	-0.65721
H	-4.94328	1.40989	-2.03032
H	-3.01914	2.94551	-1.95660
H	2.71721	-2.37398	-2.47517
H	1.46259	-3.53520	-2.91889
H	1.33970	-1.85203	-3.46200
H	-1.27319	-2.20110	-0.74319
H	-0.84601	-3.39693	-1.97740
H	-1.02511	-1.69078	-2.42531
H	2.31879	-3.02204	0.03290
H	0.68993	-2.88775	0.71204
H	1.05353	-4.14565	-0.48158
H	-3.79790	-3.06933	1.71637
H	-4.98142	-1.77861	1.36551
H	-4.24912	-2.71294	0.02620
H	0.17221	4.22140	-0.91197
H	-0.79204	3.52374	-2.24224
H	-1.60097	4.42862	-0.92515
H	3.69713	1.01962	-1.75191
H	5.62161	-0.09997	-0.64117
H	5.76026	1.65810	-0.46386
H	5.20396	0.66955	0.89973
H	2.06428	2.39870	-0.42859
H	3.68681	3.11738	-0.42482
H	3.09916	2.24574	0.99940

L5

C	-0.40761	-0.31352	3.52220
C	-1.36032	0.14493	2.61225
C	-1.09552	0.15879	1.23299
C	0.14626	-0.32262	0.78405
C	1.10256	-0.73942	1.72200
C	0.84254	-0.75656	3.09000
P	0.81580	-0.45069	-0.92078
C	2.51425	-0.83838	-0.14425
O	2.31324	-1.15942	1.24987
C	-2.12649	0.70889	0.29962
C	-3.36944	0.07026	0.12392
C	-4.34914	0.60294	-0.72456
C	-4.08419	1.79135	-1.40080
C	-2.87134	2.45673	-1.24598
C	-1.90078	1.91838	-0.38927
C	3.56141	0.29446	-0.27386
C	0.28509	-2.21724	-1.48433
C	1.09639	-2.52402	-2.75831
C	-1.20968	-2.14280	-1.84904
C	0.49368	-3.32827	-0.44288
O	-3.52830	-1.10386	0.81000
C	-4.75953	-1.79723	0.69177
O	-0.70100	2.51482	-0.14582
C	-0.33619	3.64988	-0.91100
C	4.81256	-0.06292	0.54787
C	3.92747	0.49314	-1.75444
O	2.89448	1.45300	0.24895
C	3.64825	2.63352	0.42976
H	-0.63496	-0.31023	4.58519
H	-2.31994	0.50668	2.96776
H	1.60419	-1.09547	3.78496
H	2.94016	-1.73405	-0.60761
H	-5.29954	0.10276	-0.86417
H	-4.84018	2.20788	-2.06130
H	-2.69285	3.38514	-1.77448
H	2.17316	-2.59470	-2.56083
H	0.78105	-3.48821	-3.17954
H	0.94523	-1.75752	-3.52758
H	-1.82976	-1.98705	-0.96142
H	-1.52452	-3.08736	-2.31472
H	-1.41797	-1.33436	-2.55947
H	1.54588	-3.45320	-0.16627
H	-0.07200	-3.12806	0.47245
H	0.14483	-4.28861	-0.84881
H	-4.66484	-2.67945	1.32770
H	-5.60262	-1.18684	1.04126
H	-4.94881	-2.11474	-0.34208
H	0.68305	3.89335	-0.60713
H	-0.35040	3.43163	-1.98641
H	-0.99261	4.50643	-0.70527
H	5.60244	0.68392	0.41549
H	4.56485	-0.13759	1.60904
H	5.21668	-1.02756	0.22138
H	4.68638	1.27447	-1.86746
H	3.04649	0.77880	-2.33635
H	4.34454	-0.42728	-2.17932
H	2.94148	3.39087	0.78020
H	4.43515	2.52022	1.18843
H	4.10989	2.99238	-0.50223

L6

C	-0.73004	-0.03944	3.52950
C	-1.57967	0.51976	2.57536
C	-1.36831	0.30888	1.20283
C	-0.28457	-0.49308	0.80333
C	0.57196	-1.02077	1.78393
C	0.36117	-0.81750	3.14542
P	0.31987	-0.93440	-0.87616
C	1.87580	-1.66992	-0.05053
O	1.62694	-1.77694	1.36726
C	-2.32493	0.93885	0.23738
C	-3.63416	0.43467	0.10515
C	-4.56880	1.03775	-0.74614
C	-4.19257	2.16814	-1.46639
C	-2.91477	2.70664	-1.34932
C	-1.98788	2.09704	-0.49055
C	3.18705	-0.87089	-0.28946
C	-0.61852	-2.56012	-1.32292
C	0.14306	-3.18985	-2.50621
C	-2.02627	-2.16058	-1.80257
C	-0.74579	-3.56969	-0.17111
O	-3.90792	-0.68321	0.84672
C	-5.21978	-1.22070	0.80467
O	-0.72829	2.58479	-0.28957
C	-0.37725	3.80776	-0.91786
C	4.31151	-1.51651	0.54671
C	3.53644	-0.93786	-1.78524
O	2.85681	0.43474	0.20203
C	3.54827	1.68185	-0.03051
C	5.06908	1.60792	0.17742
C	2.92987	2.58714	1.04755
C	3.21673	2.24585	-1.42493

H	-0.91343	0.14162	4.58554
H	-2.42052	1.13077	2.88902
H	1.04343	-1.24986	3.87029
H	2.04176	-2.69081	-0.40847
H	-5.56954	0.63741	-0.85160
H	-4.91086	2.64060	-2.13122
H	-2.65153	3.59109	-1.91556
H	1.15132	-3.52114	-2.23064
H	-0.39916	-4.07389	-2.86801
H	0.23584	-2.48963	-3.34501
H	-2.63137	-1.77773	-0.97734
H	-2.54056	-3.04089	-2.21361
H	-1.99032	-1.39744	-2.58859
H	0.22717	-3.91145	0.19748
H	-1.29201	-3.13888	0.67403
H	-1.29913	-4.45675	-0.51150
H	-5.21311	-2.07119	1.48888
H	-5.96640	-0.48943	1.14089
H	-5.48372	-1.56894	-0.20258
H	0.63298	4.03782	-0.57795
H	-0.37753	3.71629	-2.01180
H	-1.05462	4.61971	-0.62252
H	5.28045	-1.05737	0.33721
H	4.09079	-1.41383	1.61093
H	4.39436	-2.58508	0.31429
H	4.48990	-0.44420	-1.99383
H	2.75942	-0.47742	-2.40054
H	3.64192	-1.98538	-2.09235
H	5.56602	0.99946	-0.58460
H	5.49244	2.61728	0.11470
H	5.31146	1.20086	1.16389
H	1.84068	2.59709	0.94476
H	3.31117	3.61154	0.96521
H	3.16825	2.20399	2.04487
H	3.73534	1.71132	-2.22513
H	2.13990	2.17391	-1.60454
H	3.51500	3.29949	-1.48689

L7

C	-0.73004	-0.03944	3.52950
C	-1.57967	0.51976	2.57536
C	-1.36831	0.30888	1.20283
C	-0.28457	-0.49308	0.80333
C	0.57196	-1.02077	1.78393
C	0.36117	-0.81750	3.14542
P	0.31987	-0.93440	-0.87616
C	1.87580	-1.66992	-0.05053
O	1.62694	-1.77694	1.36726
C	-2.32493	0.93885	0.23738
C	-3.63416	0.43467	0.10515
C	-4.56880	1.03775	-0.74614
C	-4.19257	2.16814	-1.46639
C	-2.91477	2.70664	-1.34932
C	-1.98788	2.09704	-0.49055
C	3.18705	-0.87089	-0.28946
C	-0.61852	-2.56012	-1.32292
C	0.14306	-3.18985	-2.50621
C	-2.02627	-2.16058	-1.80257
C	-0.74579	-3.56969	-0.17111
O	-3.90792	-0.68321	0.84672
C	-5.21978	-1.22070	0.80467
O	-0.72829	2.58479	-0.28957
C	-0.37725	3.80776	-0.91786
C	4.31151	-1.51651	0.54671
C	3.53644	-0.93786	-1.78524
O	2.85681	0.43474	0.20203
C	3.54827	1.68185	-0.03051
C	5.06908	1.60792	0.17742
C	2.92987	2.58714	1.04755
C	3.21673	2.24585	-1.42493
H	-0.91343	0.14162	4.58554
H	-2.42052	1.13077	2.88902
H	1.04343	-1.24986	3.87029
H	2.04176	-2.69081	-0.40847
H	-5.56954	0.63741	-0.85160
H	-4.91086	2.64060	-2.13122
H	-2.65153	3.59109	-1.91556
H	1.15132	-3.52114	-2.23064
H	-0.39916	-4.07389	-2.86801
H	0.23584	-2.48963	-3.34501
H	-2.63137	-1.77773	-0.97734
H	-2.54056	-3.04089	-2.21361
H	-1.99032	-1.39744	-2.58859
H	0.22717	-3.91145	0.19748
H	-1.29201	-3.13888	0.67403
H	-1.29913	-4.45675	-0.51150
H	-5.21311	-2.07119	1.48888
H	-5.96640	-0.48943	1.14089
H	-5.48372	-1.56894	-0.20258
H	0.63298	4.03782	-0.57795
H	-0.37753	3.71629	-2.01180
H	-1.05462	4.61971	-0.62252
H	5.28045	-1.05737	0.33721
H	4.09079	-1.41383	1.61093

H	4.39436	-2.58508	0.31429
H	4.48990	-0.44420	-1.99383
H	2.75942	-0.47742	-2.40054
H	3.64192	-1.98538	-2.09235
H	5.56602	0.99946	-0.58460
H	5.49244	2.61728	0.11470
H	5.31146	1.20086	1.16389
H	1.84068	2.59709	0.94476
H	3.31117	3.61154	0.96521
H	3.16825	2.20399	2.04487
H	3.73534	1.71132	-2.22513
H	2.13990	2.17391	-1.60454
H	3.51500	3.29949	-1.48689

L8

C	0.14308	0.21172	3.49175
C	-0.90924	0.25213	2.57689
C	-0.66662	0.25515	1.19318
C	0.66404	0.18789	0.74444
C	1.70829	0.20886	1.68655
C	1.46926	0.20555	3.05904
P	1.33960	0.11628	-0.97104
C	3.00782	0.66213	-0.19221
O	2.98468	0.23677	1.20461
C	-1.82220	0.38135	0.25285
C	-2.80126	-0.62663	0.15557
C	-3.90255	-0.49330	-0.70013
C	-4.03129	0.66664	-1.45970
C	-3.09268	1.69156	-1.37963
C	-1.99723	1.55042	-0.51647
C	1.43354	-1.78033	-1.30551
C	2.22122	-1.97727	-2.61701
C	-0.02195	-2.23222	-1.54778
C	2.02941	-2.63712	-0.17631
O	-2.58109	-1.73466	0.92844
C	-3.52505	-2.79224	0.88300
O	-1.05116	2.51861	-0.34369
C	-1.12684	3.69035	-1.13868
C	4.30417	0.13178	-0.79336
C	3.01464	2.20139	-0.20662
H	-0.07147	0.21334	4.55738
H	-1.93416	0.29548	2.93142
H	2.30152	0.21419	3.75568
H	-4.64608	-1.27665	-0.78016
H	-4.88244	0.77478	-2.12675
H	-3.22182	2.58774	-1.97366
H	3.27814	-1.71276	-2.52041
H	2.17453	-3.03218	-2.92029
H	1.79664	-1.37906	-3.43201
H	-0.61960	-2.17823	-0.63368
H	-0.02495	-3.27778	-1.88690
H	-0.51631	-1.63059	-2.31929
H	1.98775	-3.69892	-0.45873
H	3.07327	-2.39344	0.03804
H	1.46295	-2.51872	0.75249
H	-3.15439	-3.54962	1.57618
H	-4.52014	-2.45991	1.20658
H	-3.59895	-3.22645	-0.12262
H	-0.26634	4.29808	-0.85325
H	-1.06580	3.45539	-2.20890
H	-2.04822	4.25437	-0.94180
H	5.15565	0.56759	-0.25665
H	4.38651	0.41709	-1.84682
H	4.38249	-0.95379	-0.71681
H	2.09967	2.60951	0.23408
H	3.87152	2.56804	0.37099
H	3.09514	2.57563	-1.23249

L9

C	0.89060	1.23169	3.23817
C	1.02032	0.20162	2.30481
C	0.33089	0.24573	1.08349
C	-0.47654	1.36184	0.80391
C	-0.62871	2.36155	1.77982
C	0.05524	2.32073	2.99401
P	-1.48015	1.75370	-0.68710
C	-2.31658	3.10486	0.35165
O	-1.47356	3.39342	1.49848
C	0.45278	-0.90304	0.13508
C	1.65805	-1.18726	-0.52394
C	1.79173	-2.27068	-1.39709
C	0.70025	-3.10956	-1.59766
C	-0.51158	-2.87742	-0.94774
C	-0.62815	-1.78111	-0.08996
C	-0.30178	2.78140	-1.80626
C	-1.20196	3.44987	-2.86469
C	0.64485	1.78942	-2.50796
C	0.52770	3.84405	-1.06825
O	2.68343	-0.27311	-0.35769
C	3.98300	-0.68990	-0.14768
C	4.30713	-1.86216	0.54182
C	5.64982	-2.16966	0.76793
C	6.66224	-1.31877	0.32159
C	6.32317	-0.14664	-0.35867

C	4.98732	0.17056	-0.59810
O	-1.76570	-1.52504	0.63981
C	-2.96987	-2.12014	0.29080
C	-3.62895	-1.75835	-0.88637
C	-4.87625	-2.31803	-1.16368
C	-5.46220	-3.22157	-0.27277
C	-4.79525	-3.56590	0.90419
C	-3.54284	-3.01839	1.18992
C	-3.69925	2.68763	0.84776
H	1.43237	1.17416	4.17855
H	1.65240	-0.65287	2.52628
H	-0.07981	3.11565	3.72034
H	-2.38649	4.03921	-0.21269
H	2.73337	-2.43930	-1.90775
H	0.79062	-3.95763	-2.27076
H	-1.35405	-3.54284	-1.09454
H	-1.89399	4.17790	-2.42455
H	-0.58173	3.99081	-3.59179
H	-1.79527	2.71115	-3.41653
H	1.34231	1.32779	-1.80312
H	1.24070	2.31987	-3.26372
H	0.09417	0.99127	-3.01952
H	1.17048	4.37521	-1.78442
H	-0.09841	4.59099	-0.56953
H	1.17625	3.39047	-0.31220
H	3.52157	-2.52248	0.89340
H	5.90114	-3.08107	1.30403
H	7.70435	-1.56527	0.50301
H	7.10175	0.52525	-0.70988
H	4.70393	1.07665	-1.12418
H	-3.16875	-1.03624	-1.55325
H	-5.39633	-2.03847	-2.07604
H	-6.43586	-3.65015	-0.49300
H	-5.24709	-4.26386	1.60383
H	-3.00733	-3.26903	2.10024
H	-4.10495	3.45125	1.52059
H	-4.38542	2.56209	0.00320
H	-3.65218	1.73765	1.39098

L10

C	0.48787	0.14332	3.35842
C	-0.46841	0.29287	2.35263
C	-0.11141	0.21393	0.99588
C	1.22931	-0.05124	0.67004
C	2.18194	-0.15168	1.69945
C	1.83018	-0.06999	3.04584
P	2.02724	-0.25244	-0.97674
C	3.69196	-0.08957	-0.07795
O	3.48304	-0.33738	1.33863
C	-1.15601	0.45471	-0.04756
C	-2.22276	-0.45630	-0.22906
C	-3.22074	-0.20069	-1.17729
C	-3.15768	0.95968	-1.94802
C	-2.11673	1.86834	-1.79379
C	-1.12478	1.62255	-0.83527
C	1.94613	-2.14757	-1.30314
C	2.91761	-2.41641	-2.46967
C	0.51054	-2.46636	-1.76129
C	2.30579	-3.03404	-0.10053
C	-3.26008	-2.44212	0.86899
C	-3.61860	-3.41017	-0.26318
C	-4.45356	-1.68245	1.45111
O	-2.14388	-1.58444	0.54208
O	-0.05554	2.47072	-0.67719
C	-0.23426	3.81390	-0.15659
C	-1.19867	3.86645	1.02648
C	-0.58455	4.81870	-1.25678
C	4.31196	1.29560	-0.24807
H	0.18676	0.21381	4.40060
H	-1.50495	0.48270	2.61463
H	2.59225	-0.16028	3.81326
H	4.39309	-0.85758	-0.41728
H	-4.02639	-0.90363	-1.33752
H	-3.92700	1.14358	-2.69343
H	-2.04999	2.74688	-2.42361
H	3.96089	-2.21435	-2.19800
H	2.85906	-3.47189	-2.76797
H	2.67376	-1.80674	-3.34763
H	-0.20975	-2.33220	-0.94876
H	0.45182	-3.51358	-2.09051
H	0.20112	-1.83481	-2.60224
H	3.32812	-2.86451	0.25279
H	1.62694	-2.86108	0.74048
H	2.22455	-4.09348	-0.38289
H	-2.82774	-3.04195	1.67746
H	-2.71054	-3.86885	-0.66651
H	-4.25802	-4.20835	0.13172
H	-4.15636	-2.93192	-1.08645
H	-4.12635	-1.02949	2.26665
H	-5.17437	-2.40058	1.85810
H	-4.97082	-1.06999	0.70823
H	0.77346	4.04747	0.20450
H	-0.90041	3.15479	1.80184
H	-1.19457	4.87350	1.45894

H	-2.22374	3.63611	0.71673
H	0.06812	4.67687	-2.12398
H	-0.44398	5.84035	-0.88374
H	-1.62625	4.72514	-1.58061
H	5.21901	1.38233	0.36061
H	4.57322	1.46965	-1.29749
H	3.61112	2.07877	0.06109

L11

C	0.21157	0.04852	3.33943
C	-0.72328	0.33736	2.34396
C	-0.40615	0.17744	0.98451
C	0.86554	-0.31479	0.64543
C	1.80652	-0.54870	1.66306
C	1.49514	-0.38783	3.01250
P	1.59308	-0.67089	-1.00643
C	3.27890	-0.82725	-0.13992
O	3.05726	-0.94203	1.29211
C	-1.41066	0.57658	-0.04989
C	-2.61280	-0.15067	-0.21179
C	-3.56522	0.25235	-1.15555
C	-3.32303	1.37896	-1.94059
C	-2.14843	2.11017	-1.80323
C	-1.20078	1.71827	-0.84862
C	1.16533	-2.52361	-1.31235
C	2.06427	-2.98014	-2.47858
C	-0.30616	-2.58012	-1.76358
C	1.36048	-3.44555	-0.09861
C	-3.95168	-1.91347	0.93844
C	-4.49788	-2.82539	-0.16490
C	-4.98225	-0.94646	1.52397
O	-2.71090	-1.26935	0.57113
O	-0.01227	2.39352	-0.70260
C	0.01670	3.75180	-0.18773
C	-0.92463	3.95553	0.99726
C	-0.18076	4.79196	-1.29289
C	4.25987	0.32583	-0.43365
C	5.63005	0.04744	0.20532
C	3.72587	1.70392	-0.01522
H	-0.05653	0.18430	4.38414
H	-1.70960	0.70113	2.61595
H	2.24476	-0.58907	3.77108
H	3.75656	-1.76541	-0.44185
H	-4.47468	-0.31394	-1.30115
H	-4.05900	1.67660	-2.68290
H	-1.94739	2.96112	-2.44228
H	3.12877	-2.97087	-2.21478
H	1.81026	-4.01016	-2.76303
H	1.93087	-2.34634	-3.36319
H	-0.98647	-2.31441	-0.94933
H	-0.55433	-3.60141	-2.08595
H	-0.50008	-1.90796	-2.60755
H	2.39674	-3.45917	0.25520
H	0.72367	-3.14256	0.73848
H	1.09069	-4.47673	-0.36781
H	-3.61233	-2.56379	1.75224
H	-3.69217	-3.44092	-0.57667
H	-5.25558	-3.49419	0.25996
H	-4.96505	-2.27614	-0.98707
H	-4.53002	-0.34558	2.31955
H	-5.80886	-1.51997	1.95832
H	-5.39953	-0.26709	0.77634
H	1.04920	3.83084	0.16936
H	-0.73456	3.21090	1.77566
H	-0.76573	4.95245	1.42396
H	-1.97399	3.88216	0.69197
H	0.44036	4.54852	-2.16073
H	0.11445	5.78231	-0.92587
H	-1.22552	4.85589	-1.61397
H	4.38419	0.32620	-1.52704
H	6.05000	-0.90398	-0.14402
H	6.34426	0.84103	-0.04347
H	5.54481	-0.00337	1.29602
H	2.76654	1.93121	-0.49240
H	4.43984	2.48665	-0.29725
H	3.58734	1.75516	1.07063

L12

C	0.28642	0.14558	3.37011
C	-0.65931	0.27004	2.35194
C	-0.28334	0.20467	0.99857
C	1.06817	-0.02508	0.68860
C	2.01043	-0.08725	1.73228
C	1.63963	-0.01877	3.07360
P	1.89801	-0.23536	-0.94816
C	3.53154	0.15147	-0.01650
O	3.32235	-0.21361	1.38336
C	-1.31990	0.43968	-0.05404
C	-2.39627	-0.46248	-0.23012
C	-3.38167	-0.21278	-1.19279
C	-3.29911	0.93360	-1.98257
C	-2.25380	1.83676	-1.82902
C	-1.27564	1.59862	-0.85446
C	1.81078	-2.14363	-1.21473

C	2.70557	-2.47870	-2.42576
C	0.34768	-2.43935	-1.60551
C	2.18051	-3.01637	-0.00419
C	-3.44706	-2.46118	0.83499
C	-3.73223	-3.42438	-0.32194
C	-4.68230	-1.73916	1.37784
O	-2.34008	-1.57221	0.56844
O	-0.20985	2.45203	-0.69283
C	-0.40277	3.79401	-0.17266
C	-1.36569	3.83702	1.01202
C	-0.76656	4.79443	-1.27243
C	4.81271	-0.54274	-0.46370
C	3.71611	1.67837	-0.07383
H	-0.03061	0.20384	4.40832
H	-1.70360	0.43284	2.60042
H	2.39559	-0.07821	3.85013
H	-4.19437	-0.90812	-1.34875
H	-4.05841	1.11156	-2.73960
H	-2.17314	2.70623	-2.46965
H	3.76990	-2.33405	-2.21987
H	2.56686	-3.53195	-2.70601
H	2.44401	-1.86670	-3.29715
H	-0.33632	-2.28045	-0.76754
H	0.26036	-3.49152	-1.91230
H	0.01398	-1.81945	-2.44574
H	2.05308	-4.07767	-0.26207
H	3.21504	-2.87757	0.32079
H	1.53192	-2.80318	0.85115
H	-3.03415	-3.06029	1.65404
H	-2.79713	-3.86210	-0.68467
H	-4.37425	-4.23804	0.03528
H	-4.23832	-2.94904	-1.16644
H	-4.40323	-1.08645	2.21128
H	-5.39809	-2.48041	1.75093
H	-5.18864	-1.13268	0.62281
H	0.60296	4.03950	0.18628
H	-1.05971	3.12867	1.78737
H	-1.37104	4.84433	1.44393
H	-2.38881	3.59644	0.70367
H	-0.11320	4.66094	-2.14047
H	-0.63808	5.81761	-0.89920
H	-1.80741	4.68791	-1.59480
H	5.65079	-0.17956	0.14363
H	5.03044	-0.31396	-1.51159
H	4.75986	-1.62605	-0.34452
H	2.81169	2.20371	0.24823
H	4.54328	1.97137	0.58374
H	3.94584	1.99889	-1.09510

L13

C	-0.69027	-0.80972	-3.32359
C	0.31159	-0.32205	-2.48379
C	0.06563	-0.06906	-1.12187
C	-1.22303	-0.33684	-0.60914
C	-2.22146	-0.79494	-1.48982
C	-1.97428	-1.04775	-2.83860
P	-1.92864	-0.10408	1.08675
C	-3.64078	-0.37439	0.31161
O	-3.47218	-0.99591	-0.98776
C	1.17891	0.49528	-0.29591
C	2.44005	-0.14452	-0.22389
C	3.50474	0.41777	0.50067
C	3.27302	1.60624	1.19404
C	2.05183	2.28105	1.15423
C	1.02798	1.74065	0.35648
C	-1.64494	-1.78217	1.98312
C	-2.63400	-1.81772	3.16594
C	-0.21381	-1.73369	2.55124
C	-1.82977	-3.03122	1.10843
C	2.12918	-2.54359	-0.60536
C	2.82126	-3.14704	0.61654
C	2.25255	-3.42869	-1.83927
O	2.72240	-1.27445	-0.96858
O	-0.17028	2.41518	0.23842
C	-0.18107	3.61968	-0.56786
C	-1.42308	4.39382	-0.14435
C	-0.16978	3.29295	-2.05979
C	-4.41708	0.92904	0.13576
C	4.86747	-0.23309	0.51537
C	1.84872	3.54175	1.96205
H	-0.47188	-0.98642	-4.37366
H	1.30146	-0.12776	-2.88128
H	-2.77550	-1.40563	-3.47707
H	-4.22833	-1.07814	0.90761
H	4.08109	2.02738	1.78959
H	-3.67632	-1.90460	2.83843
H	-2.42131	-2.68885	3.80009
H	-2.54911	-0.92175	3.79240
H	0.53663	-1.64594	1.76078
H	-0.00092	-2.65161	3.11635
H	-0.08098	-0.88290	3.22911
H	-2.84238	-3.09938	0.69709
H	-1.13111	-3.04187	0.26567
H	-1.65025	-3.93788	1.70366

H	1.06875	-2.37975	-0.38305
H	2.78288	-2.46583	1.47255
H	2.32609	-4.08082	0.90782
H	3.87080	-3.36862	0.39411
H	1.71343	-2.98842	-2.68250
H	1.83758	-4.42262	-1.63913
H	3.30582	-3.54353	-2.11929
H	0.71309	4.20902	-0.32781
H	-1.39669	4.61492	0.92710
H	-1.49148	5.33798	-0.69575
H	-2.32561	3.80782	-0.34979
H	0.72107	2.71953	-2.33226
H	-0.16915	4.22031	-2.64516
H	-1.05153	2.70575	-2.33562
H	-5.36020	0.74067	-0.38933
H	-4.63926	1.37534	1.11102
H	-3.83776	1.65474	-0.44563
H	4.87362	-1.16585	1.09116
H	5.19626	-0.48640	-0.49758
H	5.60766	0.43807	0.96243
H	2.47312	3.52154	2.86154
H	2.11887	4.44904	1.40524
H	0.80404	3.64458	2.26980

L14

C	-0.42620	-3.02067	0.49029
C	0.86630	-2.50532	0.52224
C	1.11768	-1.18917	0.10537
C	0.03665	-0.37829	-0.27407
C	-1.27932	-0.89798	-0.26341
C	-1.50111	-2.23644	0.07099
P	0.11998	1.29397	-1.05300
C	-1.65348	1.02746	-1.62205
N	-2.25844	0.00750	-0.74097
C	2.53489	-0.71629	0.02417
C	3.27582	-0.43456	1.18667
C	4.61373	-0.02489	1.11374
C	5.21521	0.09510	-0.13691
C	4.51787	-0.18964	-1.30791
C	3.18217	-0.60658	-1.22269
C	-0.06703	2.55715	0.38215
C	-0.29829	3.92371	-0.29365
C	1.27627	2.58574	1.13536
C	-1.19934	2.26895	1.37868
C	3.27892	-0.32549	3.58002
O	2.58998	-0.56857	2.36368
O	2.42769	-0.94653	-2.30576
C	2.97563	-0.76022	-3.60088
C	-5.62713	0.44352	0.68738
C	-4.50321	-0.60035	0.45513
C	-3.65639	-0.02497	-0.72363
C	-5.17624	-1.91407	-0.00471
C	-3.77355	-0.78044	1.80020
O	-4.24819	0.55047	-1.63232
H	-0.60105	-4.05904	0.75946
H	1.69826	-3.13365	0.82462
H	-2.48515	-2.67643	-0.02141
H	-1.64338	0.67253	-2.65991
H	-2.25559	1.93642	-1.59846
H	5.17909	0.20191	2.00921
H	6.25178	0.41551	-0.19968
H	5.01260	-0.09754	-2.26686
H	-1.24468	3.95657	-0.84673
H	-0.33772	4.71458	0.46750
H	0.50950	4.17121	-0.99205
H	1.45303	1.64861	1.67206
H	1.26803	3.39943	1.87402
H	2.12127	2.75719	0.45876
H	-1.19225	3.02382	2.17810
H	-2.18506	2.30521	0.90368
H	-1.07959	1.28691	1.84741
H	2.55324	-0.51180	4.37386
H	4.13326	-1.00392	3.70224
H	3.62963	0.71263	3.64621
H	2.19054	-1.05475	-4.29942
H	3.24297	0.28939	-3.77626
H	3.85833	-1.39331	-3.76154
H	-6.19028	0.62529	-0.22878
H	-6.30842	0.07710	1.46385
H	-5.21244	1.40010	1.02596
H	-5.90008	-2.24301	0.75019
H	-5.70985	-1.75675	-0.94741
H	-4.46311	-2.73171	-0.15286
H	-4.51648	-1.00712	2.57412
H	-3.26022	0.14014	2.09663
H	-3.04186	-1.58803	1.80541

L15

C	-0.13516	-1.00287	-3.04291
C	1.12193	-0.86694	-2.45647
C	1.25648	-0.28533	-1.18640
C	0.10016	0.11249	-0.49683
C	-1.17057	-0.01785	-1.10502
C	-1.28630	-0.56639	-2.38930

P	-0.00343	0.96660	1.13348
C	-1.77857	1.46778	0.67293
N	-2.23481	0.51734	-0.35917
C	2.62851	-0.07293	-0.62890
C	3.42503	-1.15862	-0.21841
C	4.72342	-0.96458	0.27147
C	5.23183	0.33006	0.34215
C	4.47944	1.42721	-0.06837
C	3.18409	1.22086	-0.56293
C	-0.25448	-0.44175	2.41666
C	-0.64835	0.25392	3.73508
C	1.11168	-1.13038	2.59923
C	-1.30736	-1.49707	2.04607
C	3.58164	-3.53269	0.06405
O	2.83232	-2.38897	-0.31304
O	2.38671	2.22686	-1.02416
C	2.84615	3.56326	-0.90851
C	-3.56654	0.06664	-0.39942
C	-3.89767	-1.23367	-0.83291
C	-5.22136	-1.66576	-0.84038
C	-6.25289	-0.83278	-0.40267
C	-5.93229	0.44727	0.04919
C	-4.61314	0.89698	0.04925
C	-1.77333	2.91859	0.16211
H	-0.22077	-1.42848	-4.03960
H	2.01132	-1.18837	-2.98936
H	-2.25235	-0.62899	-2.87793
H	-2.43300	1.37868	1.54499
H	5.32930	-1.80128	0.59672
H	6.23736	0.48682	0.72357
H	4.90234	2.42259	-0.01181
H	-1.61834	0.76077	3.66225
H	-0.72994	-0.48943	4.53960
H	0.09855	0.99637	4.03964
H	1.40737	-1.67747	1.69889
H	1.05254	-1.85229	3.42593
H	1.90391	-0.41186	2.83931
H	-1.33372	-2.28004	2.81760
H	-2.31502	-1.07574	1.97482
H	-1.07322	-1.98023	1.09197
H	2.92562	-4.38745	-0.11089
H	4.48847	-3.64029	-0.54539
H	3.86152	-3.50227	1.12519
H	2.04309	4.18795	-1.30376
H	3.03534	3.83453	0.13786
H	3.75714	3.73089	-1.49839
H	-3.11232	-1.91303	-1.14262
H	-5.44269	-2.67547	-1.17752
H	-7.28314	-1.17637	-0.40912
H	-6.71605	1.11461	0.39902
H	-4.39889	1.90352	0.38991
H	-2.76483	3.22718	-0.18727
H	-1.45564	3.60071	0.95841
H	-1.07520	3.02801	-0.67513

L16

C	-0.13516	-1.00287	-3.04291
C	1.12193	-0.86694	-2.45647
C	1.25648	-0.28533	-1.18640
C	0.10016	0.11249	-0.49683
C	-1.17057	-0.01785	-1.10502
C	-1.28630	-0.56639	-2.38930
P	-0.00343	0.96660	1.13348
C	-1.77857	1.46778	0.67293
N	-2.23481	0.51734	-0.35917
C	2.62851	-0.07293	-0.62890
C	3.42503	-1.15862	-0.21841
C	4.72342	-0.96458	0.27147
C	5.23183	0.33006	0.34215
C	4.47944	1.42721	-0.06837
C	3.18409	1.22086	-0.56293
C	-0.25448	-0.44175	2.41666
C	-0.64835	0.25392	3.73508
C	1.11168	-1.13038	2.59923
C	-1.30736	-1.49707	2.04607
C	3.58164	-3.53269	0.06405
O	2.83232	-2.38897	-0.31304
O	2.38671	2.22686	-1.02416
C	2.84615	3.56326	-0.90851
C	-3.56654	0.06664	-0.39942
C	-3.89767	-1.23367	-0.83291
C	-5.22136	-1.66576	-0.84038
C	-6.25289	-0.83278	-0.40267
C	-5.93229	0.44727	0.04919
C	-4.61314	0.89698	0.04925
C	-1.77333	2.91859	0.16211
H	-0.22077	-1.42848	-4.03960
H	2.01132	-1.18837	-2.98936
H	-2.25235	-0.62899	-2.87793
H	-2.43300	1.37868	1.54499
H	5.32930	-1.80128	0.59672
H	6.23736	0.48682	0.72357
H	4.90234	2.42259	-0.01181
H	-1.61834	0.76077	3.66225

H	-0.72994	-0.48943	4.53960
H	0.09855	0.99637	4.03964
H	1.40737	-1.67747	1.69889
H	1.05254	-1.85229	3.42593
H	1.90391	-0.41186	2.83931
H	-1.33372	-2.28004	2.81760
H	-2.31502	-1.07574	1.97482
H	-1.07322	-1.98023	1.09197
H	2.92562	-4.38745	-0.11089
H	4.48847	-3.64029	-0.54539
H	3.86152	-3.50227	1.12519
H	2.04309	4.18795	-1.30376
H	3.03534	3.83453	0.13786
H	3.75714	3.73089	-1.49839
H	-3.11232	-1.91303	-1.14262
H	-5.44269	-2.67547	-1.17752
H	-7.28314	-1.17637	-0.40912
H	-6.71605	1.11461	0.39902
H	-4.39889	1.90352	0.38991
H	-2.76483	3.22718	-0.18727
H	-1.45564	3.60071	0.95841
H	-1.07520	3.02801	-0.67513

L17

C	-0.32376	0.00538	-3.00261
C	0.87018	-0.03198	-2.28407
C	0.86839	0.11156	-0.88665
C	-0.36047	0.23743	-0.22105
C	-1.56710	0.28701	-0.96010
C	-1.54637	0.18410	-2.35768
P	-0.64835	0.49778	1.58302
C	-2.39766	1.09326	1.13721
N	-2.71549	0.52889	-0.18929
C	2.17930	0.16902	-0.16645
C	2.99606	-0.97994	-0.05932
C	4.24180	-0.90878	0.57603
C	4.67634	0.30624	1.10429
C	3.89154	1.45062	1.01382
C	2.65092	1.38518	0.36597
C	-0.94564	-1.26482	2.29139
C	-1.51747	-1.05807	3.70836
C	0.43608	-1.93781	2.40146
C	-1.88427	-2.16432	1.47368
C	3.18724	-3.28011	-1.00429
C	3.60622	-4.19067	0.15441
C	4.31860	-2.93452	-1.97408
O	2.43935	-2.12367	-0.56605
O	1.82300	2.47837	0.28362
C	2.19676	3.64797	-0.49011
C	2.82719	3.29079	-1.83451
C	3.03880	4.63543	0.32164
C	-4.01708	0.10096	-0.50826
C	-4.24916	-0.99298	-1.36714
C	-5.54619	-1.41460	-1.64809
C	-6.64928	-0.77989	-1.07384
C	-6.42842	0.28827	-0.20447
C	-5.13599	0.72887	0.07451
C	-2.41513	2.63129	1.13297
H	-0.30297	-0.07716	-4.08650
H	1.81635	-0.14954	-2.80387
H	-2.46304	0.26496	-2.93124
H	-3.12250	0.71080	1.86171
H	4.86001	-1.78932	0.68383
H	5.63721	0.35133	1.61027
H	4.21698	2.38148	1.46147
H	-2.50770	-0.58685	3.69149
H	-1.62826	-2.02843	4.21105
H	-0.85754	-0.43573	4.32402
H	0.86271	-2.14506	1.41552
H	0.33763	-2.89569	2.93183
H	1.14833	-1.31936	2.95990
H	-1.94157	-3.15918	1.93857
H	-2.90358	-1.76884	1.42325
H	-1.52070	-2.29820	0.44969
H	2.42045	-3.81798	-1.57281
H	2.76379	-4.34270	0.83618
H	3.90548	-5.16793	-0.24228
H	4.44750	-3.79571	0.73075
H	3.94341	-2.30211	-2.78515
H	4.71017	-3.85805	-2.41526
H	5.14848	-2.41396	-1.48937
H	1.22147	4.11167	-0.67538
H	2.18259	2.60668	-2.39421
H	2.96637	4.20128	-2.42846
H	3.80711	2.81929	-1.70387
H	2.60421	4.77834	1.31602
H	3.06213	5.60697	-0.18655
H	4.07374	4.29664	0.43476
H	-3.40920	-1.52694	-1.79565
H	-5.69088	-2.26350	-2.31188
H	-7.65846	-1.11471	-1.29519
H	-7.26976	0.79698	0.25959
H	-4.99827	1.57249	0.74105
H	-3.38284	3.02417	0.80182

H	-2.20685	3.01545	2.13742
H	-1.64560	3.02261	0.45862

L18

P	-0.14870	0.61007	-1.01645
C	0.32506	0.02844	0.66211
C	0.30144	2.48060	-1.01018
C	-1.94275	0.64400	-0.39015
C	1.53097	-0.53455	1.11408
C	-0.75230	0.15807	1.55410
C	-0.11359	3.24421	0.25722
C	1.82825	2.57989	-1.18805
C	-0.39188	3.08689	-2.24633
O	-1.92668	0.64736	1.05780
H	-2.44842	1.56599	-0.69165
C	1.64095	-0.88419	2.46915
C	2.67697	-0.80380	0.19221
C	-0.64600	-0.18837	2.89978
H	0.36460	2.82795	1.14940
H	0.19439	4.29611	0.17303
H	-1.19622	3.22948	0.41844
H	2.35878	2.20035	-0.30952
H	2.17492	2.02265	-2.06614
H	2.11623	3.63178	-1.32411
H	-0.09913	2.57087	-3.16831
H	-1.48537	3.04822	-2.17022
H	-0.11145	4.14367	-2.35033
C	0.57127	-0.70212	3.34702
H	2.57159	-1.31123	2.82932
C	2.58860	-1.81697	-0.78436
C	3.89063	-0.09954	0.30917
H	-1.49640	-0.06394	3.56255
H	0.68015	-0.98417	4.39109
C	3.66731	-2.09849	-1.63411
O	1.40570	-2.49514	-0.82239
C	4.97671	-0.37648	-0.53168
O	3.91368	0.87284	1.27215
C	4.84791	-1.37386	-1.49501
H	3.59627	-2.87440	-2.38623
C	1.22153	-3.48331	-1.82274
H	5.90523	0.17422	-0.44532
C	5.10983	1.61165	1.46118
H	5.68656	-1.59158	-2.15104
H	0.20972	-3.86619	-1.67798
H	1.30745	-3.05733	-2.83046
H	1.93862	-4.30778	-1.71399
H	4.90361	2.30452	2.27903
H	5.94750	0.95925	1.74030
H	5.38016	2.18183	0.56283
C	-2.74609	-0.57165	-0.89478
H	-2.68227	-0.57670	-1.98976
H	-2.24574	-1.48393	-0.54598
C	-4.19749	-0.56464	-0.46342
C	-5.18426	-0.02057	-1.29666
C	-4.58620	-1.07805	0.78147
C	-6.52187	0.01408	-0.89975
H	-4.90214	0.37510	-2.27061
C	-5.92211	-1.04665	1.18192
H	-3.83357	-1.50246	1.44015
C	-6.89519	-0.49972	0.34296
H	-7.27183	0.43689	-1.56353
H	-6.20406	-1.45189	2.15049
H	-7.93644	-0.47796	0.65380

L19

P	2.13334	-0.29421	-1.44216
C	2.04422	-0.52800	0.38672
C	2.50306	1.57872	-1.68503
C	3.87749	-1.00604	-1.31878
C	0.92990	-0.51394	1.24283
C	3.31363	-0.82326	0.91487
C	3.53283	2.16854	-0.70903
C	3.00657	1.71671	-3.13618
C	1.17010	2.33763	-1.54555
O	4.35506	-0.90776	0.04065
C	1.13492	-0.74790	2.61300
C	-0.46090	-0.32080	0.71975
C	3.52229	-1.03101	2.27798
H	4.50824	1.67684	-0.78378
H	3.68116	3.23586	-0.92610
H	3.19201	2.08550	0.32782
H	3.96697	1.21224	-3.29632
H	2.28664	1.30857	-3.85541
H	3.15449	2.77786	-3.37706
H	0.39397	1.91854	-2.19704
H	0.80167	2.32497	-0.51614
H	1.31417	3.38867	-1.83272
C	2.41458	-0.98506	3.12178
H	0.27837	-0.77127	3.28024
C	-1.14469	0.89652	0.88464
C	-1.10326	-1.36783	0.03150
H	4.51952	-1.24501	2.64856
H	2.54489	-1.16248	4.18618
C	-2.43210	1.09371	0.35398

C	-2.39527	-1.21573	-0.49661
C	-3.02368	0.02029	-0.31755
C	-0.52353	-3.50840	0.87247
H	-4.01591	0.16012	-0.74029
H	4.60082	-0.49800	-1.96034
C	-3.14239	2.43985	0.44637
C	-3.07233	-2.33570	-1.27713
C	-0.75213	2.18917	2.85746
O	-0.47281	1.95488	1.47868
O	-0.41748	-2.55113	-0.18071
H	-0.10162	-3.12222	1.80769
H	-1.56892	-3.80159	1.04077
H	0.04900	-4.38197	0.55138
H	-0.38417	1.36841	3.48308
H	-0.22369	3.10779	3.12417
H	-1.82628	2.32509	3.03618
C	-3.41194	3.03280	-0.95047
H	-4.09977	2.40557	-1.52983
H	-3.86393	4.02827	-0.86330
H	-2.48328	3.12862	-1.52299
H	-2.46614	3.13093	0.95805
H	-2.38814	-3.18929	-1.27228
C	-4.39362	-2.78111	-0.62269
H	-5.13350	-1.97148	-0.61723
H	-4.82999	-3.62377	-1.17241
H	-4.24014	-3.09612	0.41581
C	-3.29124	-1.93951	-2.75021
H	-2.34502	-1.66244	-3.22668
H	-3.72577	-2.77500	-3.31246
H	-3.97513	-1.08672	-2.83990
C	-4.44428	2.35136	1.26553
H	-4.90952	3.34002	1.35942
H	-5.17274	1.68601	0.78665
H	-4.25934	1.96545	2.27461
H	3.85341	-2.06859	-1.59286

L20

P	2.68167	-0.67447	-1.28421
C	2.61796	-0.30565	0.52181
C	2.99424	1.03201	-2.11537
C	4.44541	-1.26968	-0.97211
C	1.51427	-0.03098	1.34628
C	3.90015	-0.38487	1.09313
C	4.04455	1.91122	-1.41885
C	3.43874	0.71593	-3.55761
C	1.64756	1.77817	-2.15636
O	4.93386	-0.72882	0.27438
C	1.73917	0.20979	2.71163
C	0.11034	-0.04844	0.82476
C	4.12852	-0.12773	2.44447
H	5.03045	1.43636	-1.38406
H	4.15278	2.86033	-1.96263
H	3.74889	2.14931	-0.39222
H	4.40120	0.19112	-3.59174
H	2.69974	0.10027	-4.08369
H	3.55906	1.65010	-4.12226
H	0.85770	1.16926	-2.61184
H	1.32185	2.07565	-1.15521
H	1.75157	2.69258	-2.75712
C	3.02964	0.17634	3.24571
H	0.89143	0.40089	3.36289
C	-0.62708	1.13868	0.67592
C	-0.50244	-1.27659	0.51625
H	5.13452	-0.18852	2.84679
H	3.17680	0.36551	4.30584
C	-1.96008	1.11752	0.21737
C	-1.83621	-1.33938	0.07085
C	-2.53062	-0.13011	-0.06168
C	0.07063	-3.12337	1.88012
H	-3.54072	-0.15614	-0.45954
H	5.14661	-0.97764	-1.75720
C	-0.35707	3.04591	2.09135
O	0.02436	2.33298	0.91258
O	0.24284	-2.42996	0.64178
H	0.40203	-2.50475	2.72326
H	-0.97517	-3.41907	2.02712
H	0.69491	-4.01743	1.81838
H	-0.06136	2.49386	2.99139
H	0.18186	3.99529	2.05803
H	-1.43415	3.24121	2.11535
H	4.44818	-2.36423	-0.89094
C	-2.50984	-2.62061	-0.27628
C	-3.82815	-2.85913	0.14791
C	-1.87572	-3.59852	-1.06279
C	-4.49495	-4.03297	-0.20285
H	-4.32763	-2.12204	0.77128
C	-2.54408	-4.77049	-1.41347
H	-0.85918	-3.43139	-1.40058
C	-3.85496	-4.99401	-0.98627
H	-5.51286	-4.19745	0.14122
H	-2.03918	-5.51069	-2.02895
H	-4.37197	-5.90972	-1.26074
C	-2.75780	2.35277	-0.02198
C	-4.08589	2.43210	0.42998

C	-2.23276	3.44141	-0.73999
C	-4.86620	3.55958	0.17332
H	-4.50335	1.60547	0.99896
C	-3.01391	4.56714	-0.99680
H	-1.21072	3.39776	-1.10022
C	-4.33289	4.63238	-0.54203
H	-5.88974	3.60006	0.53694
H	-2.59156	5.39498	-1.56064
H	-4.93871	5.51188	-0.74315

L21

P	1.70412	0.17969	-1.35163
C	1.30541	-0.70016	0.22066
C	2.17849	1.96272	-0.80548
C	3.37450	-0.69603	-1.26836
C	0.06850	-0.88086	0.86224
C	2.45749	-1.29197	0.76834
C	3.06609	2.04430	0.44606
C	2.90625	2.58570	-2.01394
C	0.86918	2.73487	-0.55731
O	3.62404	-1.15605	0.07772
C	0.03634	-1.60794	2.06414
C	-1.20808	-0.36955	0.27007
C	2.43076	-1.99703	1.97110
H	4.02284	1.52855	0.31398
H	3.28520	3.09630	0.67760
H	2.56674	1.61020	1.31799
H	3.85784	2.08605	-2.23234
H	2.29032	2.54982	-2.92026
H	3.13433	3.63964	-1.80611
H	0.18650	2.66482	-1.41237
H	0.34640	2.37222	0.33220
H	1.09453	3.79862	-0.39753
C	1.20352	-2.14272	2.61504
H	-0.91731	-1.77671	2.55551
C	-1.87726	0.74030	0.81710
C	-1.76622	-0.99550	-0.85883
H	3.34339	-2.43013	2.36750
H	1.15090	-2.70148	3.54587
C	-3.05550	1.24467	0.24291
C	-2.94794	-0.52684	-1.45152
C	-3.56320	0.59073	-0.88357
C	-1.40833	-3.33870	-0.88992
H	-4.46523	0.98272	-1.34988
H	4.21307	-0.06019	-1.56105
C	-3.72634	2.48501	0.78388
C	-3.50339	-1.19269	-2.68713
C	-1.83145	1.20500	3.16152
O	-1.27743	1.41899	1.86578
O	-1.11135	-2.06022	-1.45030
H	-1.07925	-3.40339	0.15396
H	-2.48297	-3.55985	-0.94362
H	-0.85996	-4.07053	-1.48791
H	-2.89826	1.45678	3.19774
H	-1.69627	0.16590	3.48395
H	-1.28433	1.86435	3.83964
H	-2.98678	3.19585	1.16478
H	-2.72790	-1.31493	-3.45019
H	3.35489	-1.57336	-1.92758
H	-4.31091	2.97975	0.00167
H	-4.41691	2.25832	1.60750
H	-4.32089	-0.60186	-3.11199
H	-3.89682	-2.19449	-2.47219

L22

P	1.46169	1.97969	-0.58300
C	0.47841	1.46607	0.88819
C	0.21824	2.94617	-1.68567
C	2.15866	3.34767	0.51337
C	-0.24182	0.28265	1.12402
C	0.53093	2.45049	1.88984
C	-0.69732	3.91912	-0.92630
C	1.07715	3.71553	-2.70922
C	-0.64066	1.90680	-2.43000
O	1.29061	3.55723	1.64600
C	-0.94283	0.15076	2.33254
C	-0.25708	-0.84498	0.14323
C	-0.16700	2.32418	3.08957
H	-0.13430	4.69467	-0.39687
H	-1.37091	4.42282	-1.63392
H	-1.31784	3.39446	-0.19310
H	1.70392	4.48076	-2.23550
H	1.73395	3.04355	-3.27422
H	0.42621	4.22975	-3.42876
H	-0.02416	1.17065	-2.95924
H	-1.30712	1.36983	-1.74882
H	-1.26832	2.41305	-3.17649
C	-0.91239	1.16349	3.29282
H	-1.50484	-0.75844	2.52282
C	-1.42616	-1.20578	-0.54315
C	0.89472	-1.62576	-0.08892
H	-0.11025	3.10917	3.83669
H	-1.46105	1.03714	4.22237
C	-1.45913	-2.26995	-1.44890

C	0.87954	-2.70169	-0.97976
C	-0.30023	-3.01137	-1.65567
H	-0.31206	-3.84333	-2.35435
H	2.26727	4.30411	-0.00335
O	-2.52431	-0.38258	-0.36707
O	1.99720	-1.30090	0.66631
H	3.14275	3.03986	0.88964
H	-2.37662	-2.49893	-1.97961
H	1.77544	-3.29184	-1.13193
C	-3.78981	-0.90911	-0.19872
C	-4.85161	-0.12125	-0.64986
C	-4.03090	-2.12237	0.45290
C	-6.16170	-0.55266	-0.44934
H	-4.63272	0.81901	-1.14590
C	-5.34819	-2.54431	0.64017
H	-3.20120	-2.72574	0.80546
C	-6.41750	-1.76670	0.19263
H	-6.98529	0.06297	-0.80098
H	-5.53506	-3.48750	1.14683
H	-7.43940	-2.10216	0.34371
C	3.25109	-1.78283	0.31727
C	3.88389	-2.66273	1.19403
C	3.89382	-1.32606	-0.83556
C	5.18101	-3.09492	0.91009
H	3.35879	-2.98875	2.08636
C	5.18684	-1.77091	-1.11151
H	3.38452	-0.62059	-1.48436
C	5.83320	-2.65479	-0.24299
H	5.67950	-3.77805	1.59247
H	5.69445	-1.41673	-2.00477
H	6.84192	-2.99367	-0.46173

L23

P	2.04428	-0.45023	-1.07745
C	1.16531	-0.41457	0.54592
C	2.60513	1.36708	-1.37007
C	3.51464	-1.14564	-0.11937
C	-0.20341	-0.25788	0.82615
C	2.06450	-0.63204	1.60562
C	3.16663	2.08187	-0.13122
C	3.68053	1.29363	-2.47311
C	1.39082	2.14751	-1.90713
O	3.37030	-0.85285	1.28788
C	-0.61771	-0.26829	2.16875
C	-1.21460	-0.13556	-0.27120
C	1.65693	-0.62565	2.93938
H	4.04848	1.57836	0.27825
H	3.46264	3.10657	-0.39686
H	2.41740	2.14959	0.66393
H	4.58122	0.76066	-2.14525
H	3.30306	0.79629	-3.37432
H	3.99002	2.30852	-2.75618
H	0.93141	1.64570	-2.76681
H	0.62457	2.28703	-1.13955
H	1.71254	3.14516	-2.23737
C	0.30266	-0.43425	3.20704
H	-1.67463	-0.17090	2.39817
C	-1.83088	1.09528	-0.57516
C	-1.54993	-1.25542	-1.06338
H	2.38263	-0.78578	3.73014
H	-0.04518	-0.44041	4.23675
C	-2.71999	1.21038	-1.64943
C	-2.44664	-1.14013	-2.12892
C	-3.02486	0.09268	-2.42277
C	-1.45285	-3.29465	0.22847
H	-3.70760	0.18537	-3.26301
H	4.47552	-0.74026	-0.44386
C	-2.27875	2.68076	1.21632
O	-1.44850	2.22574	0.11810
O	-0.93992	-2.47045	-0.84918
H	-1.63809	-2.65088	1.09768
H	-2.64176	1.79781	1.75853
H	3.53856	-2.23678	-0.23674
C	-1.35771	3.49041	2.12051
H	-1.90840	3.87186	2.98744
H	-0.52903	2.87048	2.47384
H	-0.94410	4.34380	1.57157
C	-3.47474	3.49221	0.71917
H	-3.13428	4.35211	0.13126
H	-4.14105	2.88619	0.09834
H	-4.05644	3.86466	1.57054
C	-0.34336	-4.28369	0.55947
H	0.55979	-3.75283	0.87385
H	-0.65566	-4.95369	1.36803
H	-0.10155	-4.89082	-0.32009
C	-2.75432	-3.97926	-0.18644
H	-3.52215	-3.24474	-0.45007
H	-2.58564	-4.62972	-1.05232
H	-3.14054	-4.59204	0.63651
H	-3.12916	2.18522	-1.89042
H	-2.65637	-2.02118	-2.72637

L24

P	-0.78594	0.64366	-0.77315

C	-0.30433	-0.21890	0.78057
C	-0.77566	2.50927	-0.29683
C	-2.58661	0.17105	-0.38395
C	0.96777	-0.57487	1.26891
C	-1.44415	-0.56671	1.52438
C	-1.37589	2.82839	1.08103
C	0.69111	2.97756	-0.34191
C	-1.56308	3.24046	-1.40247
O	-2.66426	-0.26225	0.99985
H	-3.21869	1.06281	-0.45224
C	-3.17959	-0.91040	-1.31021
C	1.05400	-1.20539	2.52157
C	2.23356	-0.28912	0.53064
C	-1.35964	-1.19415	2.76771
H	-0.82278	2.33013	1.88355
H	-1.33062	3.91092	1.26586
H	-2.42499	2.52438	1.15977
H	1.29654	2.49473	0.43084
H	1.15602	2.76559	-1.31164
H	0.74030	4.06260	-0.17614
H	-1.15205	3.03166	-2.39731
H	-2.62471	2.96635	-1.41148
H	-1.51065	4.32535	-1.24025
C	-2.42505	-2.24665	-1.24641
C	-4.67603	-1.11037	-1.02032
C	-0.09346	-1.50141	3.26039
H	2.03148	-1.48481	2.90312
C	2.50786	-0.85939	-0.73476
C	3.21734	0.51421	1.11860
H	-2.26475	-1.43588	3.31551
H	-2.51366	-2.69843	-0.25240
H	-2.84440	-2.95313	-1.97214
H	-1.35889	-2.12593	-1.46603
H	-4.82671	-1.46865	0.00363
H	-5.23569	-0.17392	-1.13543
H	-5.11066	-1.84740	-1.70556
H	0.00071	-1.99569	4.22380
C	3.71393	-0.57762	-1.38740
O	1.55604	-1.69737	-1.23868
C	4.42597	0.79160	0.47629
C	4.66480	0.24750	-0.78214
H	3.92495	-1.00698	-2.35983
C	1.75513	-2.25158	-2.52900
H	5.16482	1.42824	0.95403
H	5.59597	0.45443	-1.30306
H	0.87140	-2.85909	-2.73090
H	1.83951	-1.46931	-3.29409
H	2.64814	-2.88985	-2.56061
H	-3.07758	-0.50688	-2.32889
H	3.01388	0.94011	2.09773

L25

P	1.59763	0.37014	-0.91381
C	1.06070	-0.01440	0.81994
C	1.70811	-1.35315	-1.76373
C	3.30090	0.72124	-0.14031
C	-0.22852	-0.08778	1.38647
C	2.18221	-0.20084	1.65267
C	2.39167	-2.45624	-0.94002
C	0.25918	-1.77637	-2.07555
C	2.44107	-1.14366	-3.10564
O	3.41209	-0.04219	1.08810
C	-0.34314	-0.40181	2.75302
C	-1.48137	0.19392	0.61253
C	2.06601	-0.52710	3.00258
H	1.87067	-2.62750	0.00749
H	2.37754	-3.40090	-1.50196
H	3.43454	-2.22260	-0.70779
H	-0.31228	-1.98731	-1.16829
H	-0.27836	-1.00852	-2.64377
H	0.26879	-2.69381	-2.67998
H	1.96779	-0.35525	-3.70266
H	3.49526	-0.88321	-2.97642
H	2.40252	-2.07172	-3.69189
C	0.78524	-0.63295	3.54156
H	-1.33238	-0.44957	3.19818
C	-1.77539	1.51896	0.20427
C	-2.40437	-0.84932	0.35133
H	2.95774	-0.66925	3.60451
H	0.66455	-0.87149	4.59496
C	-2.99265	1.78905	-0.52691
C	-3.62283	-0.56269	-0.37244
C	-3.87771	0.74285	-0.79997
C	-3.09396	-3.19314	0.48870
H	-4.79349	0.95204	-1.34935
H	3.21115	1.77812	0.16293
C	-3.27677	3.12786	-0.94243
C	-0.92802	2.63219	0.51253
C	-2.18286	-2.20556	0.76096
C	-4.54562	-1.62305	-0.63673
C	-1.24190	3.90268	0.10625
H	-5.45703	-1.38616	-1.18077
C	-4.29504	-2.90168	-0.21820
H	-4.19255	3.30899	-1.50034

C	-2.42766	4.15729	-0.63988
H	-1.27351	-2.44639	1.30014
H	-2.89840	-4.21163	0.81355
H	-5.00576	-3.69728	-0.42449
H	-0.02623	2.45783	1.08772
H	-0.58164	4.72826	0.35808
H	-2.65670	5.17064	-0.95832
C	4.58814	0.56555	-0.94064
H	4.73997	-0.48785	-1.19707
H	4.47067	1.11147	-1.88496
C	5.81702	1.09035	-0.18588
H	5.95670	0.55043	0.75527
H	6.72385	0.96859	-0.78854
H	5.71084	2.15637	0.04924

L26

P	-1.48846	-0.04189	-0.91699
C	-0.92843	-0.39439	0.80533
C	-1.28358	-1.70305	-1.86634
C	-3.27566	-0.18726	-0.27249
C	0.33993	-0.24636	1.40264
C	-2.01586	-0.79549	1.60224
C	-1.68311	-2.95405	-1.06902
C	-2.15191	-1.58142	-3.13525
C	0.19294	-1.80615	-2.29182
O	-3.23933	-0.86624	1.01113
C	0.48059	-0.56030	2.76573
C	1.53970	0.25395	0.65537
C	-1.87350	-1.11734	2.95171
H	-2.73371	-2.93392	-0.76115
H	-1.53461	-3.85172	-1.68549
H	-1.07247	-3.06622	-0.16728
H	-3.22334	-1.53423	-2.90904
H	-1.89004	-0.69279	-3.72161
H	-1.99433	-2.46044	-3.77419
H	0.51650	-0.92142	-2.85206
H	0.85932	-1.92465	-1.43318
H	0.33057	-2.68234	-2.94017
C	-0.60783	-0.99975	3.52221
H	1.45427	-0.44674	3.23276
C	2.60509	-0.62946	0.35353
C	1.63508	1.62443	0.31000
H	-2.73730	-1.43288	3.52787
H	-0.47088	-1.23294	4.57484
C	3.77062	-0.13352	-0.34376
C	2.80210	2.10534	-0.39423
C	3.83288	1.21466	-0.70583
C	0.74958	3.90238	0.30999
H	4.71007	1.58210	-1.23478
H	-3.87063	-0.82923	-0.93099
C	4.83829	-1.03495	-0.64894
C	2.58126	-2.02127	0.69751
C	0.62705	2.58190	0.65501
C	2.88550	3.48957	-0.74436
C	3.62676	-2.85197	0.38697
H	3.76709	3.83146	-1.28161
C	1.88975	4.36538	-0.40667
H	5.70731	-0.64280	-1.17220
C	4.77438	-2.35524	-0.29420
H	-0.24267	2.24661	1.20828
H	-0.02914	4.60755	0.58798
H	1.96683	5.41537	-0.67565
H	1.71510	-2.41559	1.21721
H	3.58108	-3.90249	0.66155
H	5.59351	-3.02861	-0.53144
C	-3.99269	1.17085	-0.12010
H	-3.35152	1.81840	0.49658
C	-5.34131	1.00557	0.59816
H	-5.21800	0.54168	1.57985
H	-5.82505	1.97988	0.73355
H	-6.02149	0.37556	0.00958
C	-4.18597	1.84272	-1.48762
H	-3.23305	2.01488	-1.99771
H	-4.81570	1.22480	-2.14165
H	-4.68628	2.81032	-1.36873

L27

P	-1.05117	-0.62740	-1.55118
C	-2.55107	-1.57206	-1.05220
C	-1.76563	0.82397	-2.59462
C	-0.79760	-1.96182	-2.86071
C	-3.36373	-1.43138	0.08605
C	-2.87048	-2.54660	-2.01265
C	-2.83120	0.42313	-3.62683
C	-2.36902	1.84064	-1.60709
C	-0.55591	1.46193	-3.30567
O	-2.03069	-2.67861	-3.07985
H	-0.46728	-1.56597	-3.82437
C	-4.51597	-2.22739	0.19150
C	-3.01970	-0.49088	1.18838
C	-4.00973	-3.34267	-1.90722
H	-3.70232	-0.03442	-3.14777
H	-3.17840	1.31574	-4.16640
H	-2.44703	-0.28247	-4.37059

H	-3.25867	1.43963	-1.11075
H	-1.64970	2.13582	-0.83415
H	-2.67000	2.74848	-2.14840
H	0.21325	1.77842	-2.59145
H	-0.08812	0.77507	-4.02190
H	-0.87685	2.34864	-3.86878
C	-4.83378	-3.16067	-0.79653
H	-5.15503	-2.11867	1.06197
C	-1.86359	-0.62320	1.97329
C	-3.84501	0.57172	1.58258
H	-4.23090	-4.07997	-2.67221
H	-5.72727	-3.77033	-0.69011
C	-1.53687	0.23025	3.03352
O	-0.93896	-1.60638	1.77224
C	-3.55620	1.44125	2.63924
O	-5.02266	0.86882	0.95034
C	-2.38788	1.27667	3.38047
C	-0.00242	-1.56315	2.87759
C	-5.47410	2.15181	1.45487
H	-2.14754	1.94511	4.20403
H	-0.04659	-2.67745	-2.50199
C	-4.70893	2.41167	2.77332
C	-0.19093	-0.19593	3.57635
H	-5.23787	2.90991	0.69822
H	-6.55861	2.09436	1.57187
H	-4.39750	3.45863	2.85826
H	-5.32917	2.18380	3.65132
H	0.99711	-1.71704	2.46535
H	-0.24182	-2.39590	3.55036
H	0.59963	0.51268	3.29395
H	-0.16638	-0.29367	4.66736

L28

P	-1.40322	1.17883	-0.53875
C	-3.02313	1.25878	0.34158
C	-1.87610	1.23080	-2.40310
C	-1.22930	3.01316	-0.13063
C	-3.87697	0.22348	0.76694
C	-3.38743	2.59323	0.58897
C	-2.99350	2.22269	-2.76087
C	-0.58261	1.59668	-3.15812
C	-2.30025	-0.19518	-2.80213
O	-2.51833	3.56929	0.19878
C	-5.09667	0.56724	1.37378
C	-3.55550	-1.22217	0.58067
C	-4.59762	2.93551	1.19184
H	-2.73141	3.25401	-2.50313
H	-3.18927	2.18937	-3.84196
H	-3.92784	1.97687	-2.24638
H	-0.24135	2.61364	-2.93034
H	0.23464	0.90509	-2.92150
H	-0.75770	1.54905	-4.24122
H	-1.52407	-0.93189	-2.56479
H	-3.21920	-0.50351	-2.29421
H	-2.48522	-0.23957	-3.88422
C	-5.45134	1.90306	1.57441
H	-5.75744	-0.22549	1.71157
C	-4.41085	-2.03908	-0.16712
C	-2.42874	-1.81968	1.19326
H	-4.84509	3.97895	1.35796
H	-6.39736	2.14096	2.05372
C	-4.15775	-3.40041	-0.35001
C	-2.16497	-3.18161	1.00585
C	-3.02726	-3.96418	0.23403
C	-0.48138	-1.52501	2.55157
H	-2.80803	-5.02022	0.09995
H	-0.81312	3.60256	-0.95111
H	-0.57632	3.12581	0.74457
O	-1.66619	-1.00224	1.97422
H	-5.28496	-1.58382	-0.62557
H	-4.83420	-4.00641	-0.94552
H	-1.29908	-3.64149	1.46782
H	-0.69966	-2.34258	3.25160
H	0.21739	-1.88037	1.78350
H	-0.02670	-0.69642	3.09719

L29

P	0.58055	-1.68847	-0.60668
C	1.98686	-2.27415	0.44294
C	1.20547	-1.83376	-2.41983
C	-0.17706	-3.39500	-0.32462
C	3.03085	-1.55037	1.05274
C	1.89347	-3.66086	0.66751
C	2.04640	-3.08552	-2.71286
C	-0.05956	-1.82601	-3.30225
C	2.02688	-0.56759	-2.72527
O	0.83901	-4.32106	0.11247
C	3.97086	-2.25271	1.82724
C	3.16631	-0.06204	0.93371
C	2.83353	-4.35835	1.42477
H	1.49328	-4.01125	-2.52307
H	2.35241	-3.09056	-3.76842
H	2.95587	-3.10881	-2.10393
H	-0.67636	-2.72090	-3.15894

H	-0.68586	-0.94772	-3.10604
H	0.22991	-1.79914	-4.36109
H	1.45777	0.34556	-2.51623
H	2.95222	-0.52966	-2.14377
H	2.30393	-0.55344	-3.78827
C	3.87739	-3.63530	1.99875
H	4.77417	-1.70063	2.30571
C	4.22443	0.49383	0.17350
C	2.27348	0.78607	1.63402
H	2.72875	-5.42911	1.56623
H	4.61626	-4.15197	2.60559
C	4.36503	1.93022	0.08599
C	2.42055	2.22031	1.53118
C	3.45642	2.75181	0.75827
C	0.38363	1.13155	3.14464
H	3.56373	3.83230	0.68559
H	-0.64554	-3.81439	-1.21754
C	5.43360	2.48175	-0.68833
C	5.17390	-0.30943	-0.54019
C	1.22954	0.28491	2.47730
C	1.51020	3.06824	2.23678
C	6.18459	0.25948	-1.27127
H	1.63113	4.14474	2.14065
C	0.51775	2.54372	3.01939
H	5.52410	3.56428	-0.74034
C	6.32119	1.67485	-1.34727
H	1.11930	-0.78751	2.58969
H	-0.39786	0.72351	3.78000
H	-0.16566	3.19889	3.55272
H	5.08500	-1.38908	-0.49219
H	6.89034	-0.37491	-1.80092
H	7.12905	2.10755	-1.93104
H	-0.93549	-3.32624	0.46597

L30

P	-1.78872	0.34841	-1.04760
C	-1.36315	0.05521	0.72539
C	-1.89404	-1.40083	-1.84094
C	-3.58016	0.61574	-0.47210
C	-0.10898	0.00689	1.36740
C	-2.53264	-0.05196	1.50166
C	-2.55861	-2.46887	-0.95893
C	-2.68719	-1.22433	-3.15212
C	-0.45814	-1.83322	-2.19137
O	-3.72872	0.06438	0.86323
C	-0.07329	-0.20141	2.75714
C	1.18755	0.19087	0.63698
C	-2.49555	-0.26894	2.87890
H	-3.59108	-2.21201	-0.69994
H	-2.57870	-3.43098	-1.48984
H	-2.00687	-2.61677	-0.02499
H	-3.73264	-0.94464	-2.97727
H	-2.23624	-0.46232	-3.79873
H	-2.69464	-2.17120	-3.70807
H	0.05199	-1.08486	-2.80869
H	0.14780	-2.00381	-1.29740
H	-0.48325	-2.77414	-2.75800
C	-1.24911	-0.34820	3.49614
H	0.88948	-0.23764	3.25796
C	2.06221	-0.90917	0.45956
C	1.56528	1.47809	0.18206
H	-3.42143	-0.35444	3.43839
H	-1.19215	-0.50521	4.57004
C	3.32281	-0.71866	-0.22260
C	2.82429	1.65289	-0.50609
C	3.66416	0.55183	-0.69364
C	1.15374	3.87580	-0.05179
H	4.61178	0.68914	-1.21076
H	-4.27613	0.05883	-1.10521
C	4.19644	-1.83689	-0.40138
C	1.75017	-2.23158	0.91772
C	0.76003	2.64286	0.39821
C	3.19274	2.95534	-0.96812
C	2.61643	-3.27688	0.72663
H	4.13957	3.06608	-1.49154
C	2.38312	4.03721	-0.75216
H	5.14095	-1.67326	-0.91511
C	3.85921	-3.08055	0.05977
H	-0.17413	2.53751	0.93779
H	0.52424	4.74296	0.12903
H	2.67603	5.02206	-1.10576
H	0.80829	-2.39822	1.42850
H	2.35342	-4.26817	1.08614
H	4.53323	-3.92096	-0.08194
C	-3.97279	2.09101	-0.44060
H	-3.27691	2.67122	0.17547
H	-3.95974	2.51011	-1.45240
H	-4.97947	2.20617	-0.02380

5.0 References

(1) Frisch, M. J.; Trucks, G. W.; Schlegel, H. B.; Scuseria, G. E.; Robb, M. A.; Cheeseman, J. R.; Scalmani, G.; Barone, V.; Petersson, G. A.; Nakatsuji, H.; Li, X.; Caricato, M.; Marenich, A. V.; Bloino, J.; Janesko, B. G.; Gomperts, R.; Mennucci, B.; Hratchian, H. P.; Ortiz, J. V.; Izmaylov, A. F.; Sonnenberg, J. L.; Williams-Young, D.; Ding, F.; Lipparini, F.; Egidi, F.; Goings, J.; Peng, B.; Petrone, A.; Henderson, T.; Ranasinghe, D.; Zakrzewski, V. G.; Gao, J.; Rega, N.; Zheng, G.; Liang, W.; Hada, M.; Ehara, M.; Toyota, K.; Fukuda, R.; Hasegawa, J.; Ishida, M.; Nakajima, T.; Honda, Y.; Kitao, O.; Nakai, H.; Vreven, T.; Throssell, K.; Montgomery, J. A., Jr.; Peralta, J. E.; Ogliaro, F.; Bearpark, M. J.; Heyd, J. J.; Brothers, E. N.; Kudin, K. N.; Staroverov, V. N.; Keith, T. A.; Kobayashi, R.; Normand, J.; Raghavachari, K.; Rendell, A. P.; Burant, J. C.; Iyengar, S. S.; Tomasi, J.; Cossi, M.; Millam, J. M.; Klene, M.; Adamo, C.; Cammi, R.; Ochterski, J. W.; Martin, R. L.; Morokuma, K.; Farkas, O.; Foresman, J. B.; fox, D. J. gaussian 16, Revision C.01 Gaussian, Inc., Wallingford CT, **2016**.

(2) (a) Lee, C.; Yang, W.; Parr, R. G. Development of the Colle-Salvetti conelation energy formula into a functional of the electron density. *Physical Review B*, **1988.** 37, 785- 792. (b) Becke, A. D. Density functional thermochemistry. III The role of exact exchange. *Chem. Phys*, **1993.** 93, 5648-5648. (c) Parr, R. G. and Yang, W. Density-functional theory of atoms and molecules. Oxford University Press, New York. **1989**

(3) Hay, P. J.; Wadt, W. R. Abinitio Effective Core Potentials for Molecular Calculations - Potentials for the Transition-Metal Atoms Sc to Hg. J Chem Phys **1985**, 82 (1), 270-283. DOI: Doi 10.1063/1.448799..

(4) Hehre, W. J.; Ditchfield, R.; Pople, J. A. Self-Consistent Molecular Orbital Methods. XII. Further Extensions of Gaussian-Type Basis Sets for Use in Molecular Orbital Studies of Organic Molecules. *J. Chem. Phys*. **1972**, 56, 2257–2261.

(5) Zhao, Y.; Truhlar, D.G. The M06 suite of density functionals for main group thermochemistry, thermochemical kinetics, noncovalent interactions, excited states, and transition elements: two new functionals and systematic testing of four M06-class functionals and 12 other functional. *Theor. Chem. Acc*., **2008.** 120, 215-41.

(6) (a) McLean, A. D.; Chandler, G. S. Contracted Gaussian-Basis Sets for Molecular Calculations.1. 2nd Row Atoms, Z=11-18. *J. Chem. Phys*. **1980**, 72, 5639–5648. (b) Krishnan, R.; Binkley, J. S.; Seeger, R.; Pople, J. A. Self-Consistent Molecular-Orbital Methods .20. Basis Set for Correlated Wave-Functions. J Chem Phys **1980**, 72 (1), 650-654. DOI: Doi 10.1063/1.438955.

(7) Grimme, S.; Ehrlich, S.; Goerigk, L. Effect of the Damping Function in Dispersion Corrected Density Functional Theory. J Comput Chem **2011**, 32 (7), 1456-1465. DOI: 10.1002/jcc.21759.

(8) (a) Weigend, F.; Ahlrichs, R. Balanced basis sets of split valence, triple zeta valence and quadruple zeta valence quality for H to Rn: Design and assessment of accuracy. Physical Chemistry Chemical Physics **2005**, 7 (18), 3297. DOI: 10.1039/b508541a. (b) Schafer, A.; Huber, C.; Ahlrichs, R. Fully Optimized Contracted Gaussian-Basis Sets of Triple Zeta Valence Quality for Atoms Li to Kr. J Chem Phys **1994**, 100 (8), 5829-5835. DOI: Doi 10.1063/1.467146.

(9) Grimme, S.; Antony, L.; Ehrlich, S.; Krieg, H.;, A consistent and accurate ab initio parameterization of density functional dispersion correction (DFT-D) for the 94 elements H-Pu. *J. Chem. Phys*., **2010**, 132, 154104.

(10) Chai, J.-D.; Head-Gordon, M. Long-range corrected hybrid density functionals with damped atom–atom dispersion corrections. Physical Chemistry Chemical Physics **2008**, 10 (44), 6615. DOI: 10.1039/b810189b.

(11) Marenich, A.V.; Cramer, C.J.; Truhlar, D.G. Universal solvation model based on solute electron density and a continuum model of the solvent defined by the bulk dielectric constant and atomic surface tensions. *J. Phys. Chem. B*, **2009**. 113, 6378-96.

(12) Glendening, E.D., Reed, A.E., Carpenter, J.E. and Weinhold, F. NBO Version 3.1. Gaussian Inc., Pittsburgh, PA. **2003**

(13) Lu, G.; Liu, R. Y.; Yang, Y.; Fang, C.; Lambrecht, D. S.; Buchwald, S. L.; Liu, P. Ligand–Substrate Dispersion Facilitates the Copper-Catalyzed Hydroamination of Unactivated Olefins. Journal of the American Chemical Society **2017**, 139 (46), 16548-16555.

(14) Varmuza, K. and Lohninger, H. 3 Principal Component Analysis of Chemical Data. In *Data Handling in Science and Technology*, Elsevier, **1990**; *5*. 43-64.

(15) (a) Sammon, J.W. A nonlinear mapping for data structure analysis. *IEEE Transactions on Computers*, **1969**. 18(5): 401–409. (b) Dzwinel, W. How to Make Sammon's Mapping Useful for Multidimensional Data Structures Analysis. *Pattern Recognition*. **1994.** *27*(7). 949-959.

(16) Bannwarth, C.; Ehlert, S.; Grimme, S. GFN2-xTB—An Accurate and Broadly Parametrized Self-Consistent Tight-Binding Quantum Chemical Method with Multipole Electrostatics and Density-Dependent Dispersion Contributions. *J. Am. Chem. Soc.* **2019**. 15(3), 1652-1671.

(17) Jouffroy, M.; Neufeld, K. Synthesis of Atropisomeric Biaryls via Chiral Suzuki–Miyaura/Enzymatic Kinetic Resolution. ACS Catal. **2022**, 12 (14), 8380– 8385.

(18) Handa, S.; Andersson, M. P.; Gallou, F.; Reilly, J.; Lipshutz, B. H. HandaPhos: A General Ligand Enabling Sustainable ppm Levels of Palladium-Catalyzed Cross-Couplings in Water at Room Temperature. Angew. Chem., Int. Ed. **2016**, 55, 4914– 4918.

(19) Patel, N. D.; Sieber, J. D.; Tcyrulnikov, S.; Simmons, B. J.; Rivalti, D.; Duvvuri, K.; Zhang, Y.; Gao, D. A.; Fandrick, K. R.; Haddad, N.; Lao, K. S.; Mangunuru, H. P. R.; Biswas, S.; Qu, B.; Grinberg, N.; Pennino, S.; Lee, H.; Song, J. J.; Gupton, B. F.; Garg, N. K.; Kozlowski, M. C.; Senanayake, C. H. Computationally Assisted Mechanistic Investigation and Development of Pd-Catalyzed Asymmetric Suzuki-Miyaura and Negishi Cross-Coupling Reactions for Tetra-ortho-Substituted Biaryl Synthesis. ACS Catal. **2018**, 8, 10190−10209.
